# Methane Catalytic Amidation via a Plausible Copper-Nitrene Intermediate

**DOI:** 10.1021/jacs.5c22747

**Published:** 2026-02-19

**Authors:** Jonathan Martínez-Laguna, Anna Cholewinska, Elena Borrego, Maria Besora, María Álvarez, Ana Caballero, Pedro J. Pérez

**Affiliations:** † Laboratorio de Catálisis Homogénea, Unidad Asociada al CSIC, CIQSO-Centro de Investigación en Química Sostenible and Departamento de Química, 16743 Universidad de Huelva, 21007 Huelva, Spain; ‡ Departament de Química Física i Inorgànica, 16777 Universitat Rovira i Virgili, 43007 Tarragona, Spain

## Abstract

The catalytic conversion of CH_4_ into CH_3_X compounds has been reported in a few cases, usually involving dehydrogenative processes in which the H atom is lost. Aiming at expanding this limited set of transformations, we have investigated the methane amidation reaction through metal-catalyzed nitrene transfer reactions, a transformation that remains unreported to date for the lightest hydrocarbon. Herein, we describe the use of copper-based catalysts for the direct, nondehydrogenative amidation reaction of methane via a metal-mediated formal nitrene insertion into the C–H bond, a reaction that is also extended to the series of gaseous alkanes. Mechanistic studies, supported by DFT calculations, a microkinetic model, and experimental evidence have led to the proposal of a metallonitrene intermediate responsible for this C–H amidation process via sequential hydrogen abstraction and rebound steps.

## Graphical abstract

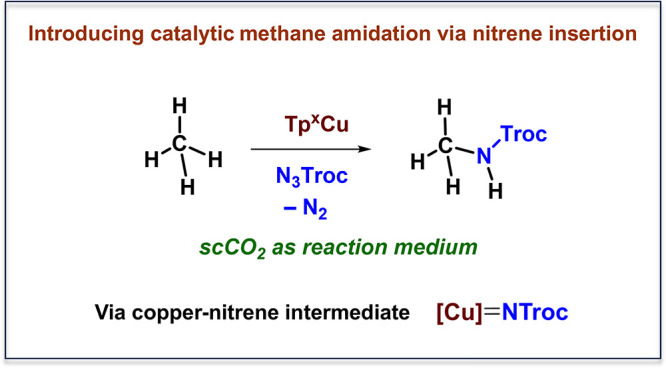

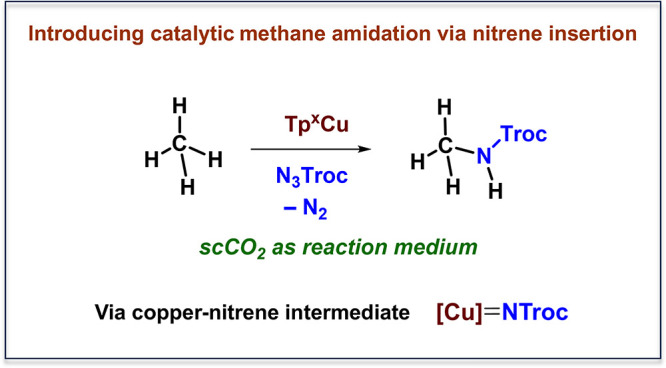

## Introduction

The direct functionalization of methane remains a major challenge in modern chemistry^1,2^ primarily due to the exceptional stability of its C–H bond, which possesses the highest bond dissociation energy among alkanes^3^ and a poor polarity.^4^ These factors lead to two major drawbacks: an inherent low reactivity and, once reacted, the appearance of selectivity issues, since very frequently the product contains C–H bonds more reactive than those of the parent methane. Despite these problems, there are a few examples in which the homogeneous metal-catalyzed functionalization of methane has been achieved upon generating C–O,^5,6^ C–B,^7,8^ C–Si,^9^ or C–C^10^ bonds. Of particular interest is the oxidation into methanol involving the intermediacy of metal-oxo species M=O (Scheme 1a),^11^ which display a very high electrophilicity that facilitates the reaction with the methane C–H bond, a rather poor nucleophile. Interestingly, the analogous methane amidation reaction via nitrene transfer remains unreported, a transformation that should take place via similar metal-nitrene intermediates M=NR (Scheme 1a).^12^

**Scheme 1:**
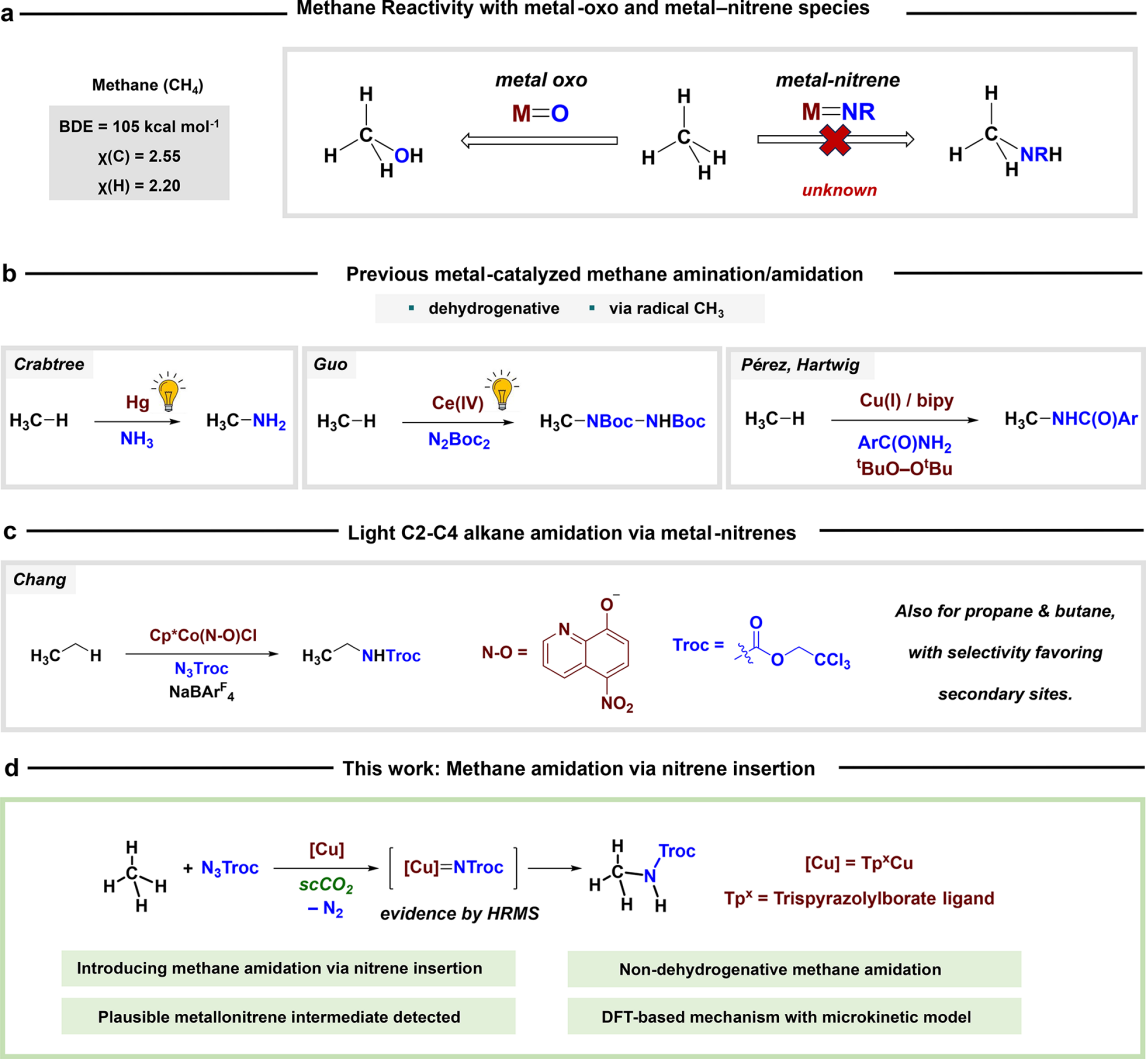
Methane Catalytic Amidation^a^

a (a) Metal-oxo and metal-nitrene reactive species for methane functionalization. (b) Previous examples for dehydrogenative methane amidation via methyl radicals. (c) Chang’s seminal work for gaseous C2–C4 alkanes C–H bond amidation by catalytic nitrene transfer. (d) This work: methane amidation via nitrene insertion.

To date, examples of metal-catalyzed methane amination/amidation have been reduced to a few (Scheme 1b). Crabtree first reported the formation of methyl radicals employing Hg/hν in the reaction of methane and ammonia, leading to methylamine.^13^ More recently, Guo described the amidation of methane using cerium-based photocatalysts and hydrazines as nitrogen sources.^14^ Later, in a thermal transformation, Hartwig and Pérez reported the copper-based methane amidation in the presence of peroxides.^15^ It is noted that these three transformations correspond to dehydrogenative processes, implying the formal loss of hydrogen from the substrates as well as the formation of methyl radicals upon hydrogen abstraction, either photochemically or peroxide-induced. Stoichiometric metal-driven methane amination is also known.^16^

The metal-catalyzed transfer of nitrene to C–H bonds constitutes an alternative route for the above goal. However, whereas this strategy has been successfully applied for condensed alkanes,^17–19^ it was not until the recent seminal work by Chang^20^ that light alkanes C2–C4 were amidated, employing a cobalt-based catalyst and an azide (N_3_Troc; Troc = −C­(O)­OCH_2_CCl_3_) as the nitrene source (Scheme 1c). Selectivity was preferred toward secondary sites using propane or butane. After this breakthrough, methane remains the only alkane that has not been amidated via nitrene-transfer reactions. Therefore, we have targeted this goal following our previous investigations on the use of coinage metal complexes bearing trispyrazolylborate ligands for the transfer of NTs (Ts = *p*-toluenesulfonate) units from PhI=NTs to saturated bonds.^17,21,22^ With the appropriate combination of the Tp^x^ ligand, the metal (copper), and the nitrene source (N_3_Troc), we herein describe the first example of the direct amidation of methane via formal nitrene insertion into the C–H bond of the lightest alkane (Scheme 1d). The reaction proceeds via a copper-nitrene intermediate that has been detected by mass spectrometry, a species predicted by DFT calculations. Expansion to C2–C4 alkanes has been explored as well, with high success.

## Results and Discussion

### Catalytic Amidation of Alkanes

We first evaluated the capabilities of several Cu- and Ag-based complexes to transfer the NTroc moiety to the C–H bond of cyclohexane as the probe reaction (Scheme 2a). Among the complexes tested, those containing copper and Tp^x^ ligands led to the best conversions into C_6_H_11_–N­(H)­Troc (**1**), the product derived from the insertion of the NTroc unit into the cyclohexane C–H bond (see SI for the optimization of reaction conditions). It is also noteworthy that the use of N_3_Ts or other azides proved to be ineffective (see SI), assessing the singular behavior of N_3_Troc. The mass balance is completed with NTroc-derived products due to decomposition pathways previously reported by Chang in his seminal contribution.^20^ The main byproduct is 2,2,2-trichloroethyl carbamate (>80% of byproducts), with very minor amounts (<20% of byproducts) of 2,2,2-trichloroethanol, methyl carbamate, methyl alkyl carbamates, alkyl isocyanate, and alkyl chlorides (see SI). The order of reactivity for the Cu-based catalysts is Tp* < Tp*^,Br^ < Tp^(CF3)2,Br^ < Tp^Ms^ < Tp^CF3^ < Tp^Br3^. We have previously found^18^ that for nitrene transfer reactions using Tp^x^M cores (M = Cu, Ag), an increase in electrophilicity provides better results, albeit an excess of electron deficiency results in a decrease of chemoselectivity, since decomposition pathways are also enhanced. This would explain why the Tp^(CF3)2,Br^-based catalyst is less effective than Tp^Br3^Cu.

**Scheme 2:**
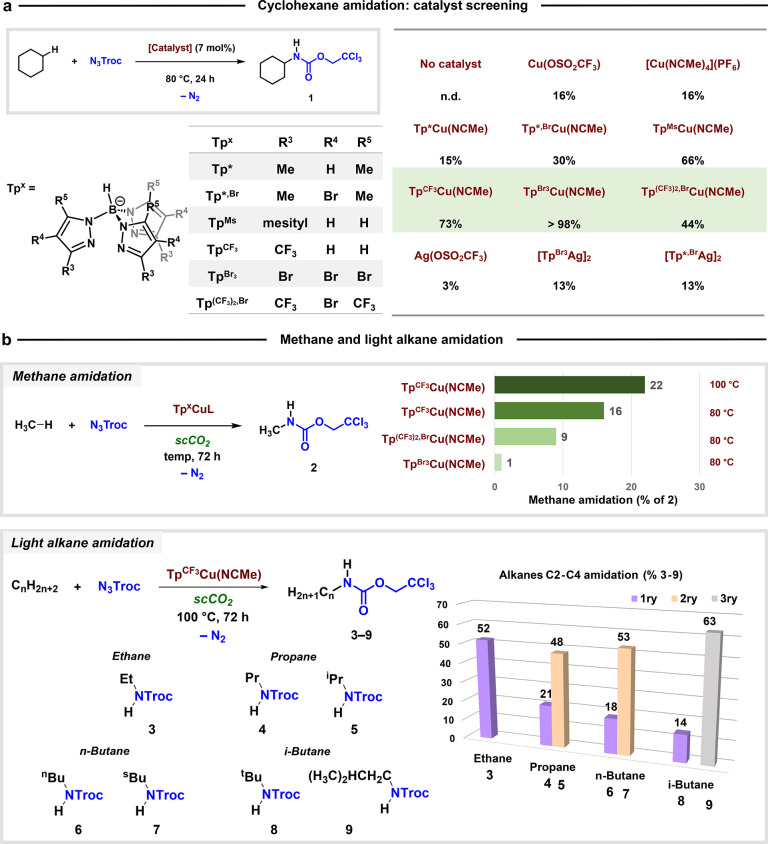
Alkane Amidation via Copper-Catalyzed Nitrene Transfer from Azide^a^

a (a) Cyclohexane probe reaction for catalyst screening. (b) Methane and light alkane amidation. All yields are referred to as the initial azide.

Once the viability of the Tp^x^Cu–N_3_Troc system was demonstrated with cyclohexane, we moved to light alkanes (Scheme 2b). Given the high bond dissociation energies of these hydrocarbons,^3^ the selection of the solvent is crucial: the highly reactive metal-nitrene intermediates might react with any nucleophile present in the reaction medium rather than with the targeted alkane. To address this issue, we have employed supercritical carbon dioxide, ensuring a one-phase medium with no other possible reaction site than the alkane C–H bond.^23^ The use of scCO_2_ as a solvent is becoming routine, and many homogeneous catalytic systems have already been described in such a medium.^24^ With methane as the reactant, we tested three Tp^x^Cu catalysts differing in their ancillary ligand. The best results were obtained with Tp^CF3^Cu­(NCMe), which leads to a 22% yield (azide-based) of CH_3_–N­(H)­CO_2_CH_2_CCl_3_ (**2**) in the first example of methane amidation by nitrene insertion. The poor performance of the Tp^Br3^-containing catalyst is explained due to its low solubility in the scCO_2_–CH_4_ medium, whereas both Tp^CF3^Cu and Tp^(CF3)2,Br^Cu complexes are soluble in such a mixture. The mass balance was completed with azide-derived decomposition products (see SI).

Alkanes C2–C4 were also evaluated in scCO_2_, with yields (based on initial azide) ranging from 52% (ethane) to 77% (iso-butane). The selectivities favor the secondary (propane, butane) or tertiary (iso-butane) sites, albeit the functionalization of the primary sites takes place to a higher degree compared to the cobalt system reported by Chang.^20^ Accordingly, the selectivities for primary (1ry) and secondary (2ry) sites for propane and butane with the copper catalyst are 1:7 and 1:4.4, respectively. These values are statistically corrected with the number of hydrogens of each type in the alkane. The cobalt catalyst provided 1:24 for propane and 1:16 for butane. We have also evaluated iso-butane, which generates a mixture of the two products derived from the functionalization of the primary and tertiary sites, with a corrected selectivity of 1:20. Overall, the reactivity observed follows the order C–H primary (1ry) < C–H secondary (2ry) < C–H tertiary (3ry).

Back to methane as the substrate, we first evaluated the effect of the pressure on the reaction outcome, carrying out additional experiments with 50 and 100 bar of CH_4_ in scCO_2_ as the solvent. Scheme 3a shows the variation of the yield of **2** with methane pressure, with a linear correlation being found. In another set of experiments, using 160 bar of methane, the reaction was monitored with time. Eight identical experiments were run and analyzed at 3, 6, 10, 16, 24, 48, 72, and 100 h, with the results shown in Scheme 3b. The yield increases up to ca. 60 h, and after 80 h, the reaction is finished. The average TOF, calculated at 80 h, is 5.5 × 10^–4^ mmol **2** h^–1^. These data will be employed to develop the microkinetic model (see below).

**Scheme 3:**
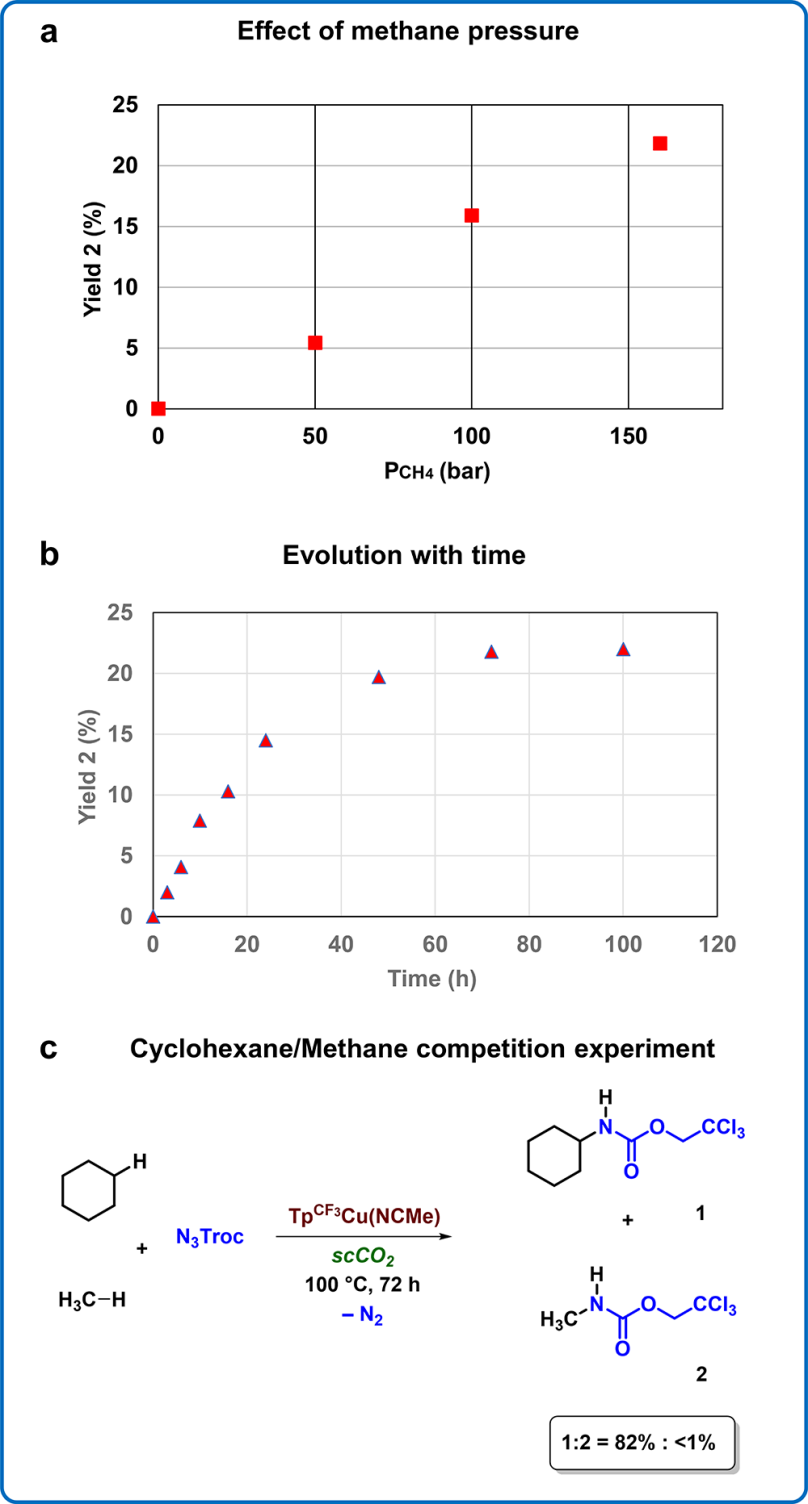
Methane Amidation Experiments^a^

a (a) Effect of methane pressure. (b) Monitoring of methane amidation with time. (c) Cyclohexane-methane competition experiment.

The observation of a decrease in yields from cyclohexane to methane can be explained by two factors. On one hand, there is a difference in their C–H bond dissociation energies, with cyclohexane being 6 kcal mol^–1^ lower than methane.^3^ On the other hand, the concentration of the alkane is much higher when using a liquid hydrocarbon than when using a scCO_2_–CH_4_ mixture. To compare the relative reactivity of methane and cyclohexane, an experiment in scCO_2_ using 92 mmol of cyclohexane and 260 mmol of methane was carried out. The main product was the cyclohexyl derivative (Scheme 3c), with <1% yield of **2** being quantified (see SI for details).

### DFT Calculations

We have performed DFT calculations (B3LYP-D3/def2TZVP&def2QZVP-SMD//B3LYP-D3/6–31g­(d)&LANL2DZ-SMD, see full details in SI) to elucidate the reaction mechanism for the methane amidation process. All computational results are available in the ioChem-BD repository^25^ and can be accessed via https://iochem-bd.urv.es/browse/handle/100/2265. Scheme 4 shows the reaction free energy profile. A microkinetic model has been built using the computational free energies and mechanism (see SI for details). The catalyst precursor Tp^CF3^Cu­(NCMe) (**Int0** ^**S**^) first dissociates MeCN, leading to intermediate **Int1** ^**S**^, which is 11.6 kcal mol^–1^ less stable than **Int0** ^**S**^. The azide N_3_Troc coordinates to **Int1** ^**S**^, a step that may form two species depending on the atom coordinated to copper: the internal nitrogen of azide (**Int2**_**N**_^S^) or the carbonyl oxygen (**Int2**_**O**_^S^). The lack of added acetonitrile and the relatively high concentration of N_3_Troc help their formation even though their energies are relatively high, −1.9 for **Int2**_**N**_^S^ and 0.9 kcal mol^–1^ for **Int2**_**O**_^S^ with respect to **Int1** ^**S**^. The transient concentrations during the first 24 h range between 3.0 × 10^–3^ and 2.6 × 10^–3^ mol L^–1^ for **Int0**, 7.2 × 10^–6^ and −2.4 × 10^–6^ mol L^–1^ for **Int1** ^**S**^, and 8.3 × 10^–8^ and 1.0 × 10^–8^ mol L^–1^ for **Int2**_**N**_^**S**^, according to the fitted microkinetic model (373.15 K) (see Figure S18 and details in the SI). Nitrogen extrusion from **Int2**_**N**_^S^ occurs through **TS2–3**_**N**_^S^ located at 17.6 kcal mol^–1^ above **Int1** ^**S**^ and results in the formation of molecular nitrogen and the copper nitrene intermediate **Int3**_**N**_^**S**^ in the singlet surface. The overall Gibbs free energy barrier for the formation of the copper nitrene intermediate **Int3**_**N**_^**T**^ is 29.2 kcal mol^–1^ (from **Int0** ^**S**^ to **TS2**_**N**_^**S**^). The microkinetic model shows that this barrier is accessible under the experimental conditions and that a 2 kcal mol^–1^ larger barrier would slightly better fit the experimental results (see SI). Consequently, when experimental concentrations are considered, the process is expected to be feasible at 80 °C, with the formation of the nitrene being thermodynamically favored and kinetically viable. The nitrene intermediate is more stable as a triplet, **Int3**_**N**_^**T**^, and the crossing from the singlet to triplet surface can be done through the minimum energy crossing point (MECP), **MECP3**_**N**_. **Int3**_**N**_^**T**^ and **MECP3**_**N**_ are located at −15.1 and −2.4 kcal mol^–1^ relative to **Int1** ^**S**^, respectively. The most stable copper nitrene **Int3**_**N**_^**T**^ can isomerize upon oxygen coordination into **Int3**_**ON**_^**T**^ and **Int3**_**ON**_^**S**^ (which are 6.6 and 3.2 kcal mol^–1^ less stable, respectively).

**Scheme 4:**
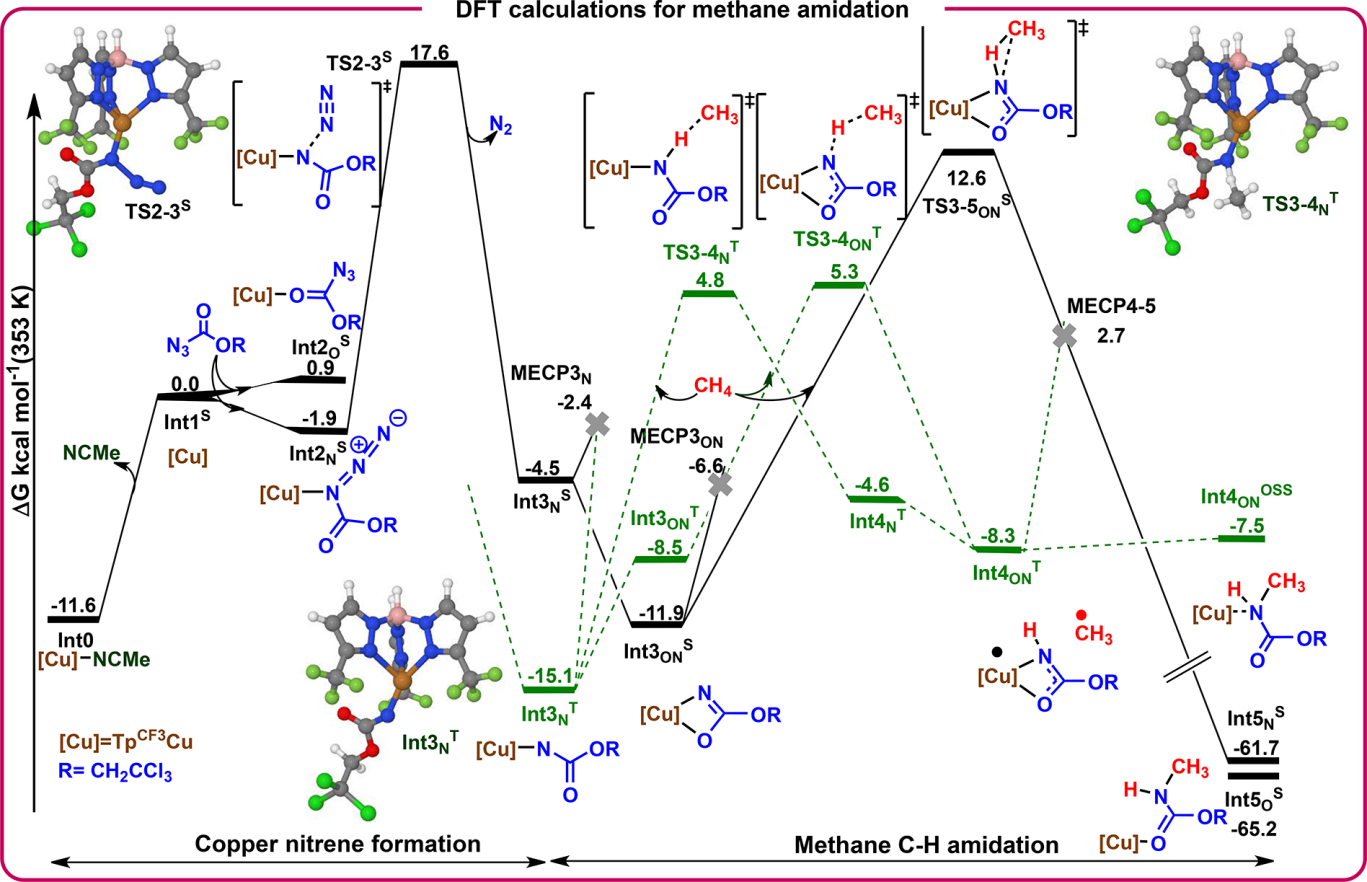
Reaction Free Energy Profile for Copper Nitrene Formation and Methane Amidation^a^

a Black solid lines represent the singlet surface, and green dashed lines represent the triplet surface.

The mechanism for the reaction of methane with the triplet copper nitrene **Int3**_**N**_^**T**^ has been explored in both the triplet and singlet surfaces (Scheme 4). Transition states **TS3–4**_**N**_^**T**^ and **TS3–4**_**ON**_^**T**^, located at 19.9 and 20.4 kcal mol^–1^ above **Int3**_**N**_^**T**^, activate and cleave the methane C–H bond in a homolytic manner, generating the new N–H bond. The resulting intermediates, **Int4**_**N**_^**T**^ and **Int4**_**ON**_^**T**^, contain CH_3_ and Tp^CF3^Cu–N­(H)­COOCH_2_CCl_3_ radicals. The reaction further evolves with the formation of the N–CH_3_ bond through **MECP4–5**, or via radical separation and rebound through the equivalent open shell singlet (**Int4**_**ON**_^**OSS**^) to form the singlet product. **MECP4–5** is located 11.0 kcal mol^–1^ above **Int4**_**ON**_^**T**^, 2 kcal mol^–1^ below **TS3–4**_**N**_^**T**^, and leads to **Int5**_**N**_^**S**^. The final product coordinates weakly to copper through oxygen, **Int5**_**O**_^**S**^. Overall, the barrier associated with the formal insertion of the triplet copper nitrene to the C–H bond of methane is computationally estimated as 19.9 kcal mol^–1^ (**Int3**_**N**_^**T**^ to **TS3–4**_**N**_^**T**^), whereas the reaction is thermodynamically favored by 61.4 kcal mol^–1^. We use the term formal insertion based on the composition of the final product, although the mechanism does not proceed following an insertion pathway such as that proposed for the C–H bond functionalization by carbene insertion.^26^

The singlet potential energy surface has also been evaluated, revealing a mechanistically simpler but more energy-demanding pathway. Transition state **TS3–5**_**ON**_^**S**^ forms the N–H and N–C bonds in an asymmetric concerted way, connecting singlet copper nitrene intermediate **Int3**_**ON**_^**S**^ with product **Int5**_**N**_^**S**^. **TS3–5**_**ON**_^**S**^ is 7.8 kcal mol^–1^ higher in energy than **TS3–4**_**N**_^**T**^, and hence, amidation is expected to take place via the triplet surface.

The mechanism for ethane, propane, and *iso*-butane functionalization has also been computationally studied (Scheme 5 and Figures S4–S9), revealing that the reaction proceeds via the same pathways found for methane. In the two latter cases, the terminal and internal C–H bonds compete and lead to different products **4**–**5** and **8**–**9**, respectively. Insertion of the nitrene into the tertiary C–H bond is more favored than secondary CH_2_, and both are more favored than the insertion into the primary CH_3_ bonds, in good qualitative agreement with the already mentioned experimental reactivity trend. The reaction barriers from **Int3**_**N**_^**T**^ to the corresponding lowest-energy triplet transition states (equivalent to **TS3–4**_**N**_^**T**^) are 19.9, 16.5, 17.2, and 14.9 kcal mol^–1^ for the primary C–H bonds of methane, ethane, propane, and *i*-butane, respectively, 14.5 kcal mol^–1^ for the internal C–H bond of propane (secondary), and 14.0 kcal mol^–1^ for the internal C–H of *iso*-butane (tertiary, see SI). For ethane and propane, the preferred reaction pathway is, similar to methane, the homolytic C–H cleavage and formation of the N–H bond on the triplet surface, followed by formation of the N–C bond when crossing to the singlet surface. The largest barrier (once the nitrene is formed) is the homolytic cleavage of the C–H bond (see Scheme 5, Figures S4–S7). For *iso*-butane, the same mechanism operates for both primary and tertiary C–H bonds, but, in this case, the singlet mechanism is competitive for tertiary C–H bonds (Scheme 5). The singlet transition state for the internal C–H bond of *iso*-butane is stabilized and more asynchronous than for methane (estimated, see SI for details), presumably due to the larger stability of the corresponding carbocation.

**Scheme 5:**
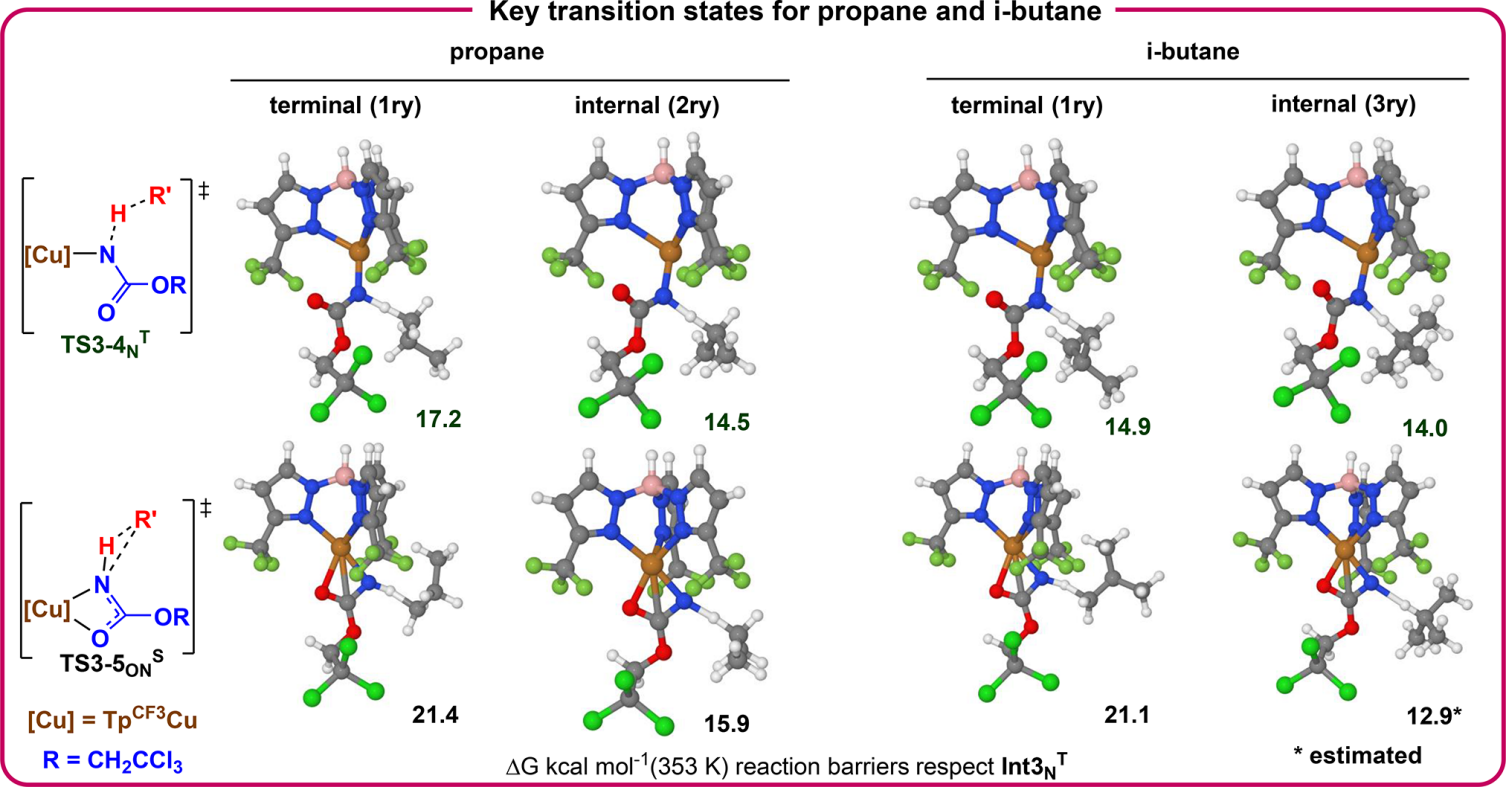
Schematic Comparison of the Key Transition States for Propane and i-Butane, Showing the Relative Barriers and Geometries for the Terminal and Internal C–H Bonds

The microkinetic models for ethane and methane as substrates are presented in detail in the SI. They are built using the above postulated computational mechanism for alkane amidation, converting computational energies to rate constants, testing different mechanistic proposals, and fitting the microkinetic model to the experimental yield for ethane and the kinetic curve of methane amidation (see Scheme 6). These models accurately reproduce the experimental data (Scheme 3b, Figure 1) and provide insight into the NTroc decomposition side reaction (SR). A good fit with experimental results is achieved when the side reaction mechanism consists of (i) a first selectivity-determining step that could involve water and have a barrier of 6–7 kcal mol^–1^ and (ii) a second energy-demanding step for catalyst recovery (around 30 kcal/mol).

**Scheme 6:**
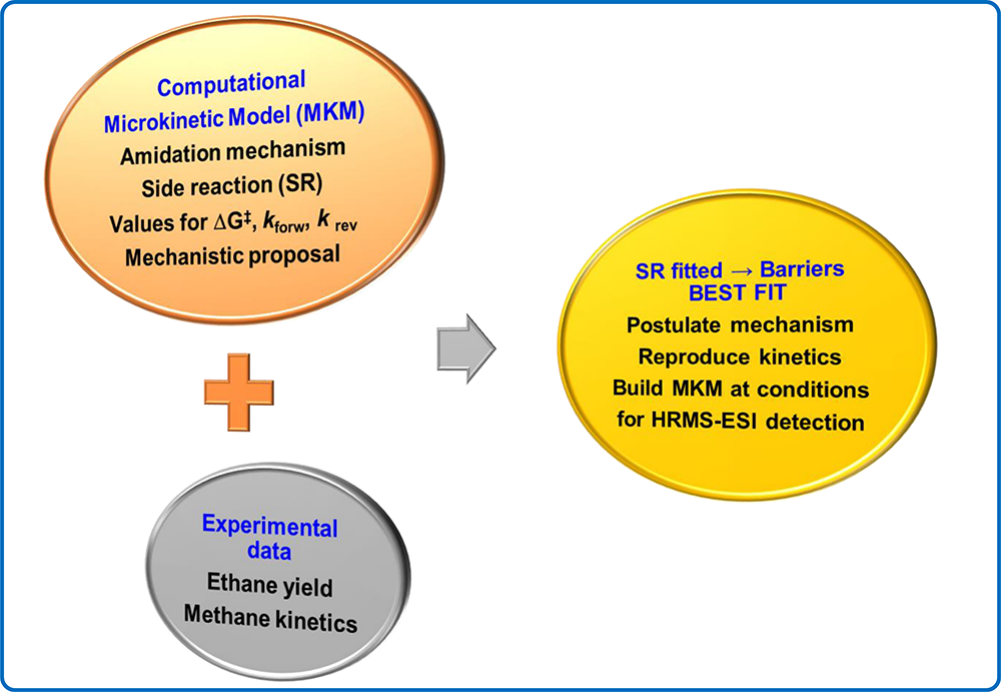
Construction of the Microkinetic Model for Alkane Amidation and Fitted NTroc Decomposition Side Reaction

**Figure 1:**
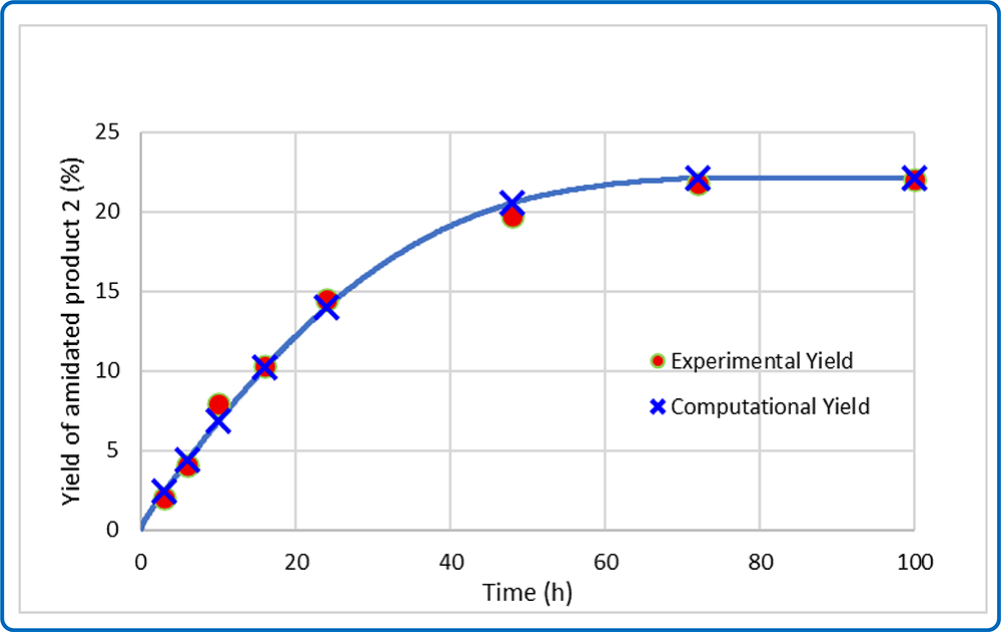
Fitted kinetic curve for methane amidation from the microkinetic model.

### HRMS-ESI Studies

To obtain experimental evidence supporting the calculations, we investigated the reaction of the catalyst Tp^CF3^Cu­(NCMe) with N_3_Troc using HRMS-ESI techniques, aiming at detecting short-lived intermediates. Thus, when a mixture of both compounds (with 0.1% MeOH added to ensure proper ionization of the sample) at room temperature was analyzed by HRMS-ESI (positive mode), two species with masses corresponding to two of the intermediates proposed from DFT calculations were detected (Scheme 7a). One of them matches that of the azide adduct [Tp^CF3^Cu­(N_3_Troc)]^+^ (**A** in Scheme 7b), whereas the other fits the mass expected for the metallonitrene [Tp^CF3^Cu­(NTroc)] (**B** in Scheme 7b and **Int3**_**N**_^**T**^ in calculations). It is noteworthy that when this HRMS-ESI experiment was carried out in a protic medium (MeOH), the species [Tp^CF3^Cu–N­(H)­Troc], resulting from the abstraction of a hydrogen atom from the medium, was detected as the result of the interception of the copper-nitrene, demonstrating the capabilities of such species to induce a HAT step in the C–H bond activation process. Calculations considering the experimental solvent and conditions for detection were performed (Figure S20) and used to build a new microkinetic model. The results show that after 10 min of reaction, the concentration of the intermediate would be of the order of 1 × 10^–9^ or 1 × 10^–10^ mol L^–1^, depending on the model used (see Figures S21–S24 and accompanying text), and the concentration of **Int2**_**N**_^**S**^ would be 1 × 10^–9^ mol L^–1^ for both models and hence detectable in HRMS-ESI.

**Scheme 7:**
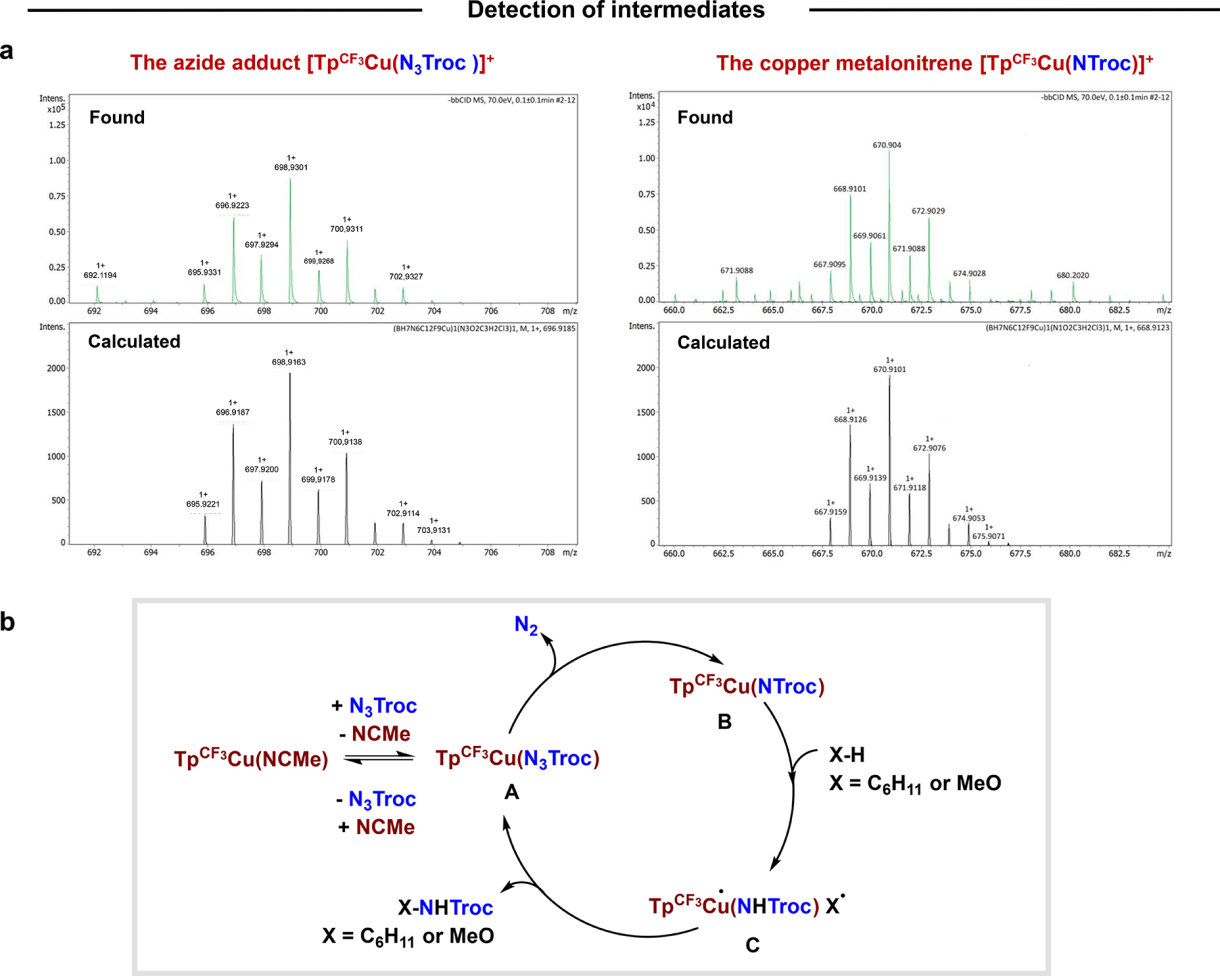
(a) Experiments for the Identification of Intermediates by HRMS-ESI; (b) Simplified Mechanism for Cyclohexane/Methanol Amidation Reactions

### KIE and Radical-Trap Studies

In the search for additional evidence to support the mechanistic picture, a competition experiment with cyclohexane and cyclohexane d-12 was carried out, leading to a value of KIE = 2.1 (Scheme 8a). This value is similar to that reported by Chang with a cobalt-based catalyst (2.2) and could be interpreted as the result of the homolytic C–H bond cleavage not being the rate-determining step. However, it is worth mentioning that the KIE values reported in the literature for amidation reactions via nitrene transfer are very sensible to the electronic nature of the N-substituent: the more electron-withdrawing the substituent, the lower the KIE.^27^ Therefore, comparison with other NR moieties may induce misinterpretation. The computational KIE has been estimated for secondary carbons of propane (similar to those of cyclohexane). A KIE of 4.5 was found in good qualitative agreement with experiments, which supports the postulated mechanism. Two supplementary experiments oriented to support the involvement of radical species complete this section (Scheme 8b). On one hand, the presence of a radical trap such as BHT (2,6-di-t-butyl-4-hydroxytoluene) completely inhibits the reaction. On the other hand, when CCl_4_ is added instead of BHT, the cyclohexyl radical is trapped in the form of C_6_H_11_Cl,^28^ assessing the homolytic cleavage of the cycloalkane C–H bond.

**Scheme 8:**
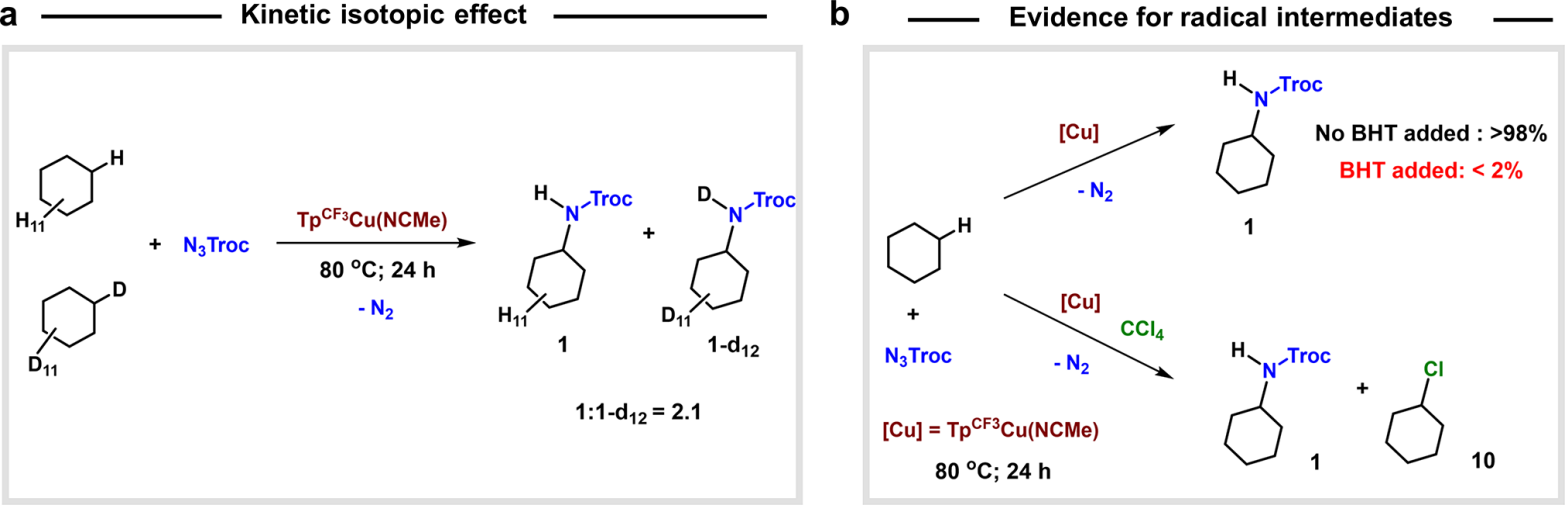
(a) Competition of Cyclohexane H_12_ and D_12_ to Calculate the KIE; (b) Experiments Assessing the Intermediacy of Radical Species: Inhibition by BHT (2,6-di-t-butyl-4-hydroxytoluene) and Cyclohexyl Radical Trapping with CCl_4_

## Conclusions

We have demonstrated access to catalytic methane amidation via nitrene transfer. The combination of tailored Tp^x^Cu-based catalysts, azide as a nitrene source, and supercritical CO_2_ as the reaction medium enabled the direct, nondehydrogenative functionalization of methane upon the formal insertion of a nitrene unit into the C–H bond, a strategy that can also be applied to the other gaseous alkanes. The detection of the key copper-nitrene intermediate provides experimental evidence of the DFT-calculated mechanism. In view of the similarities between oxo- and nitrene-transfer reactions from copper centers,^29,30^ we believe that these results constitute an excellent foundation for the related and more challenging direct oxidation of methane into methanol via oxo insertion into the C–H bond of methane and will inspire further research in this direction.

## Supplementary Material



## Notes

All procedures and characterization data for new compounds, catalytic experiments, and DFT calculations (PDF)
