# Supplementary material for: Methane Catalytic Amidation via a Plausible Copper-Nitrene Intermediate
Source: J Am Chem Soc. 2026 Feb 19;148(8):9057–65. doi: 10.1021/jacs.5c22747 (PMC12964415; doi:10.1021/jacs.5c22747)
Supplement: Supplementary file 1 [file ja5c22747_si_001.pdf]

Supporting Information for

Methane catalytic amidation via a plausible copper-nitrene intermediate

Jonathan Martínez-Laguna, ‡ Anna Cholewinska,§ Elena Borrego, ‡ Maria Besora, §* María Álvarez, ‡* Ana Caballero, ‡* Pedro J. Pérez‡*

*Corresponding authors: perez@dqcm.uhu.es (PJP), ana.caballero@dqcm.uhu.es (AC); maria.alvarez@dqcm.uhu.es (MA); maria.besora@urv.cat (MB)

Contents

1. General information. S3

2. General procedures S4

 2. a. General procedure for cyclohexane functionalization

 2. b. General procedure for gaseous alkanes

3. Synthesis and characterization of $Tp^{CF3}Cu(NCMe)$ S8

4. Synthesis and characterization of 2,2,2-trichloroethyl isobutylcarbamate (**9**)..............S11

5.Detection of intermediates by HRMS-ESI...S13

6. Kinetic isotopic effect (KIE) determination experiment....................................S18

7. Reaction with cyclohexane adding BHT as radical inhibitor.................................S19

8. Reaction in presence of CCl_4 as radical trap...S20

9. Kinetic study: reaction with methane in $scCO_2$ S21

10. Study of the effect of pressure in the reaction with methane in $scCO_2$ S22

11. Competition experiment: cyclohexane vs methane S23

12. NMR spectra for catalytic experiments S24

13. Computational details S30

14. Free energy Profiles for methane, ethane, propane and iso-butane amidation S32

 14. a. Methane amidation

 14. b. Ethane amidation

 14. c. Propane amidation

 14. d. iso-Butane amidation

15. Microkinetic model S40

15. a. Construction of the microkinetic model

15. b. Considered mechanisms for side reaction

15. c. Microkinetic model corrections and fitting

15. d. Fitting of the microkinetic model for ethane with experimental results

15. e. Fitting of the microkinetic model for methane with experimental results

15. f. Computational study of the hydrolysis first step's

15. g. Summary of the results for the side reaction mechanism

16. Computational prediction of the KIE for propane. S63

17. Computational insight on the nitrene intermediate detection conditions........ S64

18. Cartesian coordinates (in Ångström, Å) and potential energies (in Hartree) of the optimized structures S68

19. References S95

1. General information

All air– and moisture–sensitive manipulations were carried out with standard Schlenk techniques under nitrogen atmosphere or in a glovebox (MBRAUN UNILAB) under an atmosphere of purified nitrogen. All reactants were purchased and used without further purification. Solvents were purchased and dried using standard protocols. Gaseous alkanes were obtained from Air Liquide and used as received. 2,2,2-trichloroethylcarbonyl azide (N_3-Troc)[1] and $NaTp^{CF3}$ ligand[2] were prepared according to the literature method. The complexes $[Tp^{*,Br}Ag]_2$,[3] $[Tp^{Br3}Ag]_2$,[4] $[Tp^{Ms}Cu(THF)]$,[5] $[Tp^{*}Cu(NCMe)]$,[6] $[Tp^{*,Br}Cu(NCMe)]$,[7] $[Tp^{Br3}Cu(NCMe)]$[8] and $[Tp^{(CF3)2Br}Cu(NCMe)]$,[9] were prepared following the reported procedures. IR spectroscopy was performed using a Bruker Alpha FT-IR. Nuclear magnetic resonance (NMR) spectra was recorded at room temperature from solutions in $CDCl_3$ (unless otherwise noted) on Bruker spectrometer operating at 400 MHz (^1H NMR) and were referenced to residual solvent peak (chloroform: 7.26 ppm for ^1H NMR). Chemical shift values for ^1H, ^{13}C and ^{19}F are reported as δ (ppm) relative to the deuterated solvents. Gas chromatography studies were performed using an Agilent Technologies 7820A GC System. GC-MS spectrometry was performed employing an Agilent Technologies 7890B GC System coupled to an Agilent technologies 5977A MSD (EI ionization; Single Quadrupole). High-resolution mass spectra were obtained on a Bruker Compact Elite QTOF with an electrospray ionization source (ESI) by positive mode. CHN elemental analyses were performed at the elemental analysis services from the Instituto de Investigaciones Químicas of Sevilla (Spain) employing a LECO TruSpec CHN Elemental Analyzer. With gaseous alkanes, the experiments were performed in a PARR Micro Bench Top reactor with a teflon container connected to a commercial Iberfluid supercritical plant for the experiments using $scCO_2$.

2. General procedures

2. a. General procedure for cyclohexane functionalization

In a typical experiment, a 50 mL J-Young ampoule protected from light was charged with 0.009 mmol of the corresponding catalyst (7%). Subsequently, N_3-Troc (0.13 mmol) dissolved in 5 mL of cyclohexane was added. The ampoule was sealed, and the resulting mixture was heated under stirring for 24 h at 80 ºC. Then, the mixture was filtered off and an aliquot from filtrate was analyzed by GC, revealing full consumption of N_3-Troc. Reaction mixtures were filtered through silica to remove the catalyst (DCM as eluent). Then, volatiles were removed under vacuum and the resulting residue was analyzed by ^{1}H NMR. Yields were determined by ^{1}H NMR analysis employing mesitylene as internal standard and identified by its comparison with literature precedents.[10]

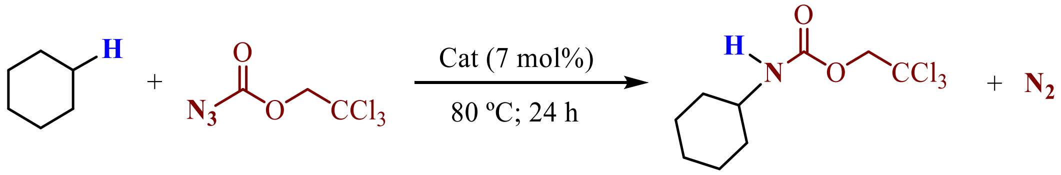

Cyclohexane **N_3-Troc** **1**

Partial decomposition of azide, as reported by Chang,[11] gave mainly 2,2,2-trichloroethyl carbamate (> 80% of byproducts), with minor amounts of 2,2,2-trichloroethanol, methyl carbamate, methyl alkyl-carbamates, alkyl isocyanate and alkyl chlorides (< 20% of byproducts). These compounds are detected by GC and GC-MS analysis, to complete the mass balance on initial azide.

Table S1. *Catalyst screening with cyclohexane.*

Entry	Catalyst	Yield (%)^a
**1**	none	n. d.
**2**	$Ag(OSO_2CF_3)$	3
**3**	$[Tp^{*,Br}Ag]_2$	13
**4**	$[Tp^{Br3}Ag]_2$	13
**5**	$Cu(OSO_2CF_3)$	16

Entry	Catalyst	Yield (%)[a]
**6**	$[Cu(NCMe)_4](PF_6)$	16
**7**	$Tp^{*}Cu(NCMe)$	15
**8**	$Tp^{Ms}Cu(THF)$	66
**9**	$Tp^{*,Br}Cu(NCMe)$	30
**10**	$Tp^{CF3}Cu(NCMe)$	73
**11[b]**	**$Tp^{CF3}Cu(NCMe)$**	**91**
**12**	**$Tp^{Br3}Cu(NCMe)$**	**>99**
**13**	$Tp^{(CF3)2,Br}Cu(NCMe)$	44

[a]Yields are referred to initial azide. n. d. = not detected. [b]The reaction was carried out employing 10 mol% of catalyst at 100 ºC.

2. b. General procedure for gaseous alkanes

The experiments were performed in a PARR Micro Bench Top reactor with a Teflon container. For a typical experiment, inside a glovebox, the reactor was charged with 0.018 mmol of the catalyst (10 mol% referred to azide) and N_3-Troc (0.183 mmol) in two separate compartments. The reactor was sealed, removed from the glove box and connected to a supercritical plant. The reactor was first pressurized with the desired alkane (isobutane 1.5 bar; n-butane 1 bar; propane 7 bar; ethane 30 bar; methane 160 bar; for experiments with isobutane, n-butane and propane, the reactor was cooled in an ice/water bath prior to the pressurization) under stirring at 40 ºC and then $scCO_2$ was incorporated adding 90 bar to the reactor pressure from the supercritical plant at 40 ºC. The temperature was set at 100 ºC, and the mixture was stirred for 72 h. Once the final reaction temperature is achieved, a final pressure of 415 bar (methane), 320 bar (ethane), 260 bar (propane) or 240 bar (isobutane or n-butane) is reached. Once the reaction was completed, the reactor was cooled in a water/ice bath and the system slowly depressurized. The resulting mixture was extracted with dichloromethane and filtered through silica to remove the catalyst (DCM as eluent). GC studies revealed no N_3-Troc remaining. Then, volatiles were removed under vacuum and the resulting residue was

analyzed by ^{1}H NMR. Yields were determined by ^{1}H NMR analysis using mesitylene as internal standard, the product being identified by comparison with literature precedents,[11-13] with the exception of compound **9** that has been synthetized (see below). By-products from azide decomposition were also detected, as mentioned above for the cyclohexane experiment.

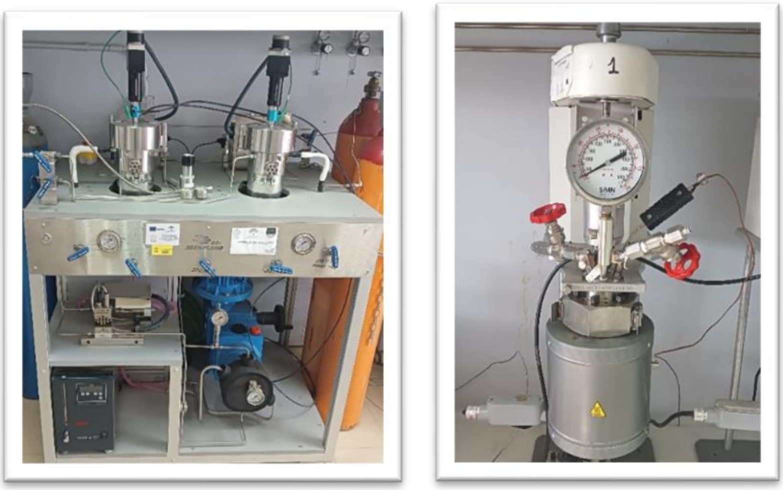

Figure S1. *Iberfluid supercritical plant for the experiments using $scCO_2$ (left) and PARR Micro Bench Top reactor with a teflon container inside (right).*

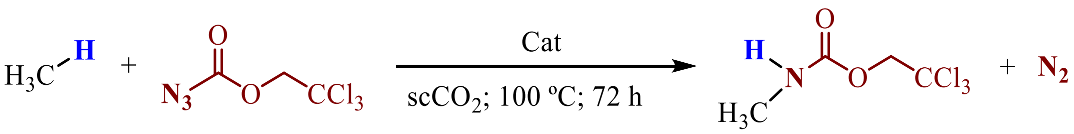

Methane **N_3-Troc** **2**

Table S2. *Methane amidation with different catalysts employing $scCO_2$ as reaction medium.*

Entry	Catalyst	T (ºC)	Yield (%)[a,b]
**1**	$Tp^{CF3}Cu(NCMe)$	80	16
**2**	$Tp^{Br3}Cu(NCMe)$	80	1
**3**	$Tp^{(CF3)2,Br}Cu(NCMe)$	80	9
**4**	**$Tp^{CF3}Cu(NCMe)$**	**100**	**22**

[a]Yields are referred to initial azide. [b]The remaining initial azide decomposes into a mixture of $TrocNH_2$ (80% of byproducts) and those mentioned in page S4 (< 20% of byproducts).

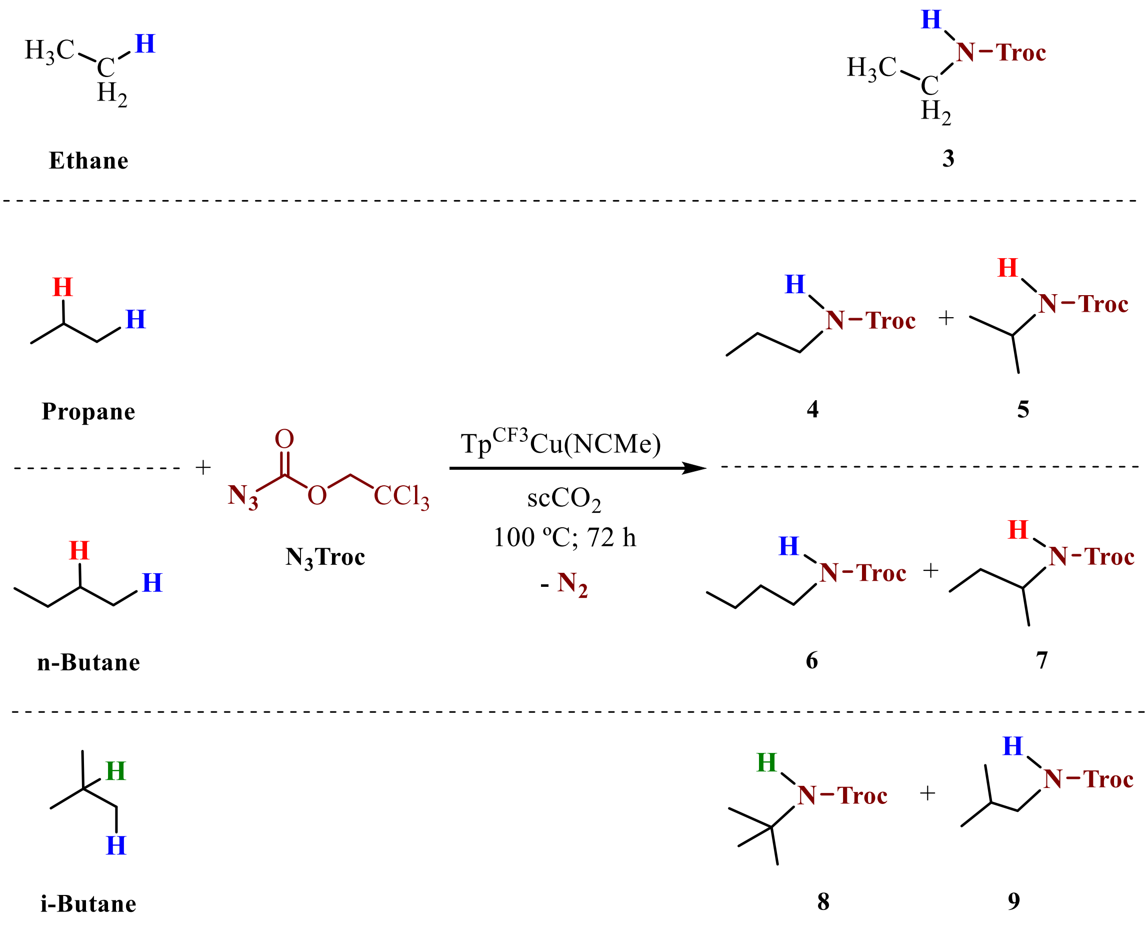

Table S3. *Results obtained in the functionalization of C2-C4 alkanes in scCO₂.*

**Entry**	**Substrate**	**Product distribution (C1 : C2 : C3)**	**Corrected selectivity[a] (C1 : C2 : C3)**	**Yield (%)[b]**
**1**	iso-Butane	18 : - : 82	1.0 : - : 40	77
**2**	n-Butane	25 : 75 : -	1.0 : 4.5 : -	70
**3**	Propane	30 : 70 : -	1.0 : 7.0 : -	69
**4**	Ethane	-	-	52

[a]Correction based on the number of H atoms of each type. [b]Yields are referred to initial azide.

3. Synthesis and characterization of $Tp^{CF3}Cu(NCMe)$

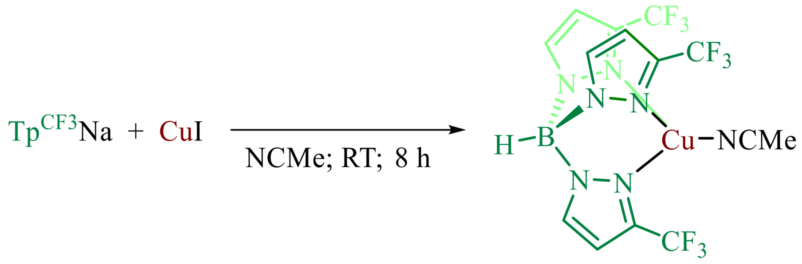

To a 50 mL Schlenk flask containing a solution of 191 mg of CuI (1.0 mmol) in 15 mL of dry acetonitrile, $NaTp^{CF3}$ was added (442 mg, 1.0 mmol). The resulting white suspension was stirred at rt for 8 h affording a colorless solution that was taken to dryness resulting in a light green solid. The solid was extracted with a mixture 1:10 of DCM-n-hexane (3 x 5 mL). The extracts were combined, and the solvent was removed under vacuum yielding $Tp^{CF3}Cu(NCMe)$ as a white solid (282 mg; 54%).

^{1}H NMR (400 MHz, $CDCl_3$) δ 7.63 (d, J = 2.3 Hz, 3H), 6.43 (d, J = 2.3 Hz, 3H), 4.54 (br s, 1H), 2.21 (s, 3H) ppm. **^{11}B{^{1}H} NMR** (128 MHz, $CDCl_3$) δ -3.15 (bs) ppm. **^{13}C{^{1}H} NMR** (101 MHz, $CDCl_3$) δ 142.6 (q, J = 37.4 Hz), 135.6 (s), 121.4 (q, J = 268.8 Hz), 113.5 (s), 103.6 (q, J = 2.2 Hz), 2.4 (s) ppm. **^{19}F NMR** (376 MHz, $CDCl_3$) δ -61.46 (s) ppm. **IR** (selected; cm^{-1}): ν (B–H) = 2515. **HRMS-ESI** m/z calculated for $C_{14}H_{10}BCuF_9N_7Na$ $[M+Na]^+$ 544.0138, found 544.0136 as a higher-intensity signal of a complex isotopic pattern. **Elemental analysis** found (calculated) for **$Tp^{CF3}Cu(NCMe)$ ($C_{14}H_{10}BCuF_9N_7$)**: C 34.8 (32.2) H 2.0 (1.9) N 18.8 (18.8).

^{1}H NMR (400 MHz, $CDCl_3$) spectrum of $Tp^{CF3}Cu(NCMe)$

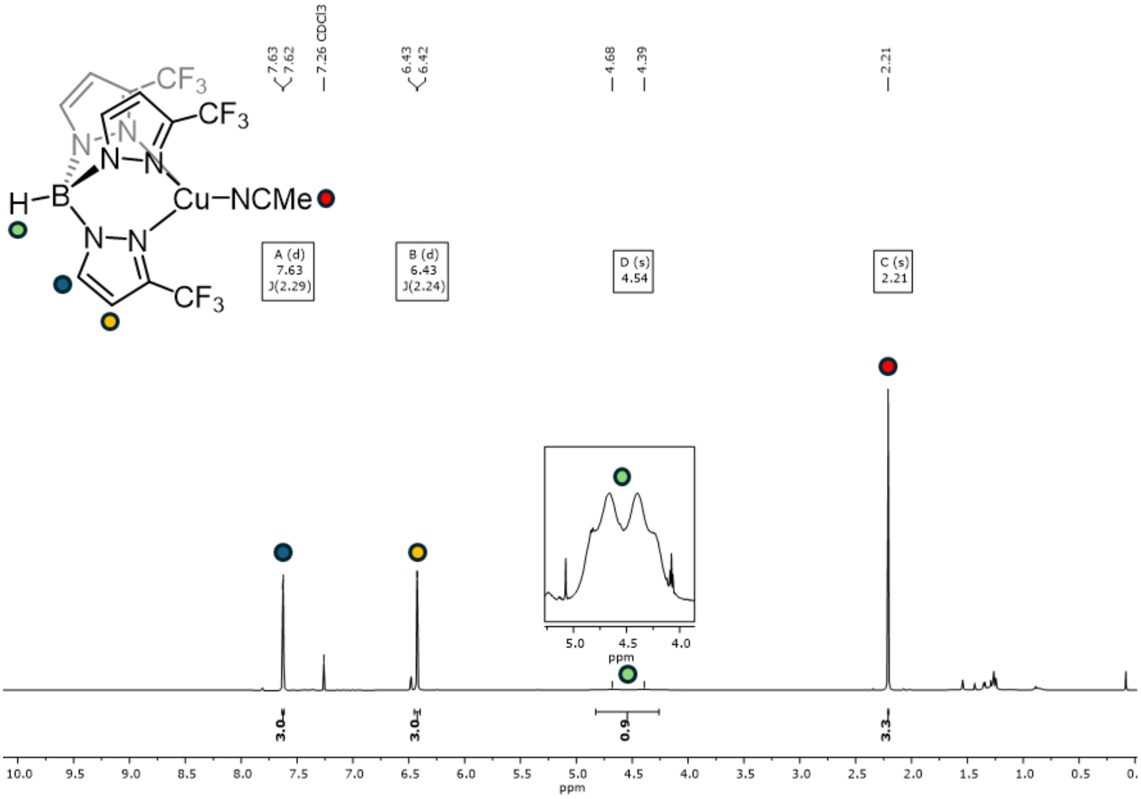

$^{11}B\{^{1}H\}$ NMR (128 MHz, $CDCl_3$) spectrum of $Tp^{CF3}Cu(NCMe)$

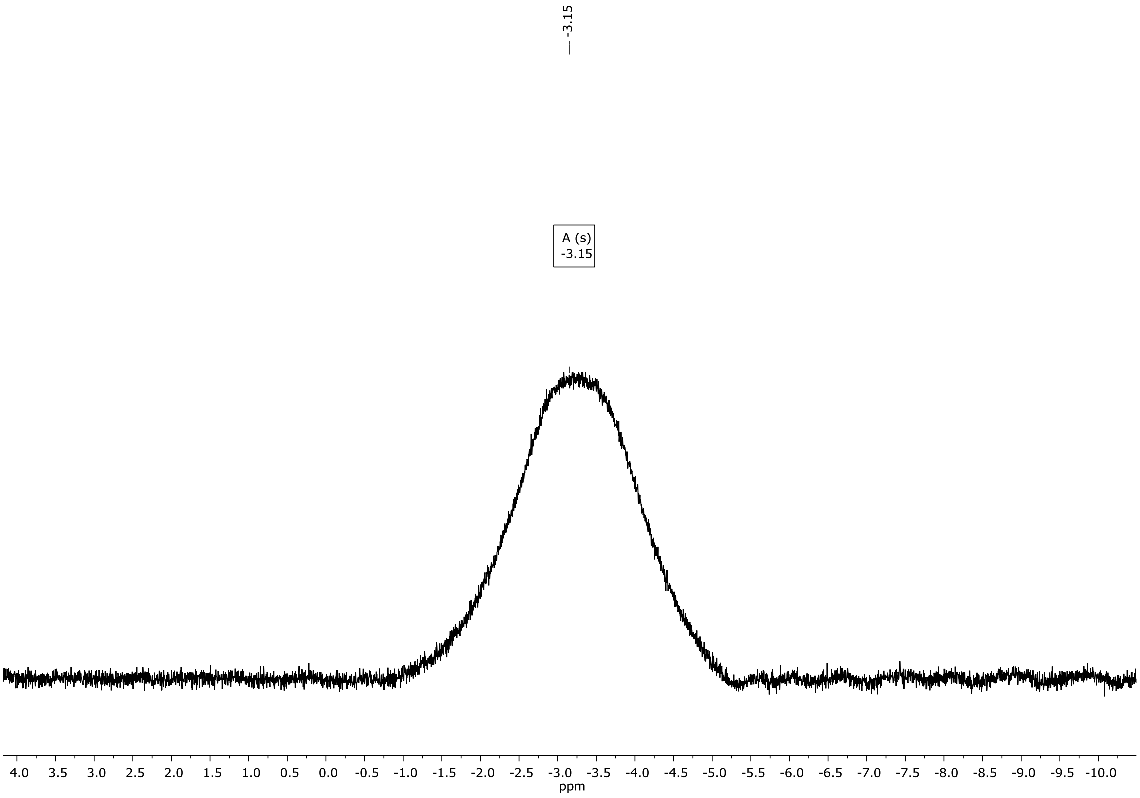

^{13}C {^{1}H} NMR (101 MHz, CDCl3) spectrum of Tp^{CF3}Cu(NCMe)

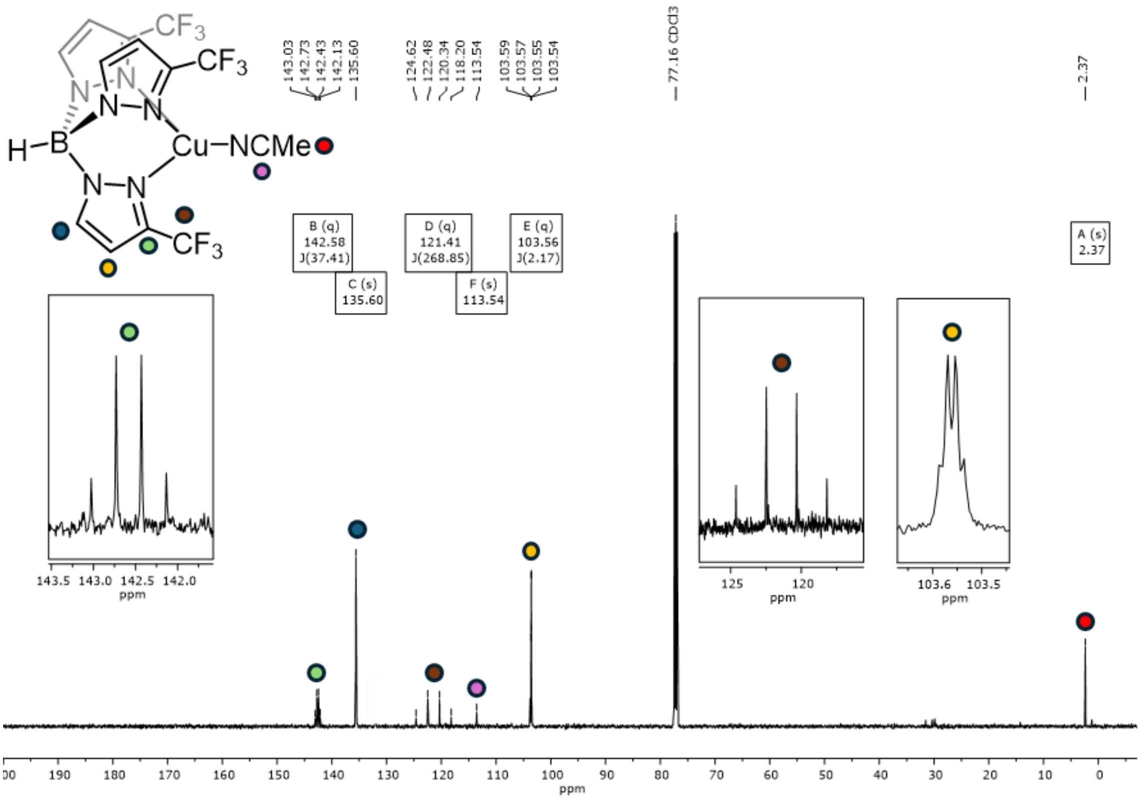

^{19}F NMR (376 MHz, CDCl3) spectrum of Tp^{CF3}Cu(NCMe)

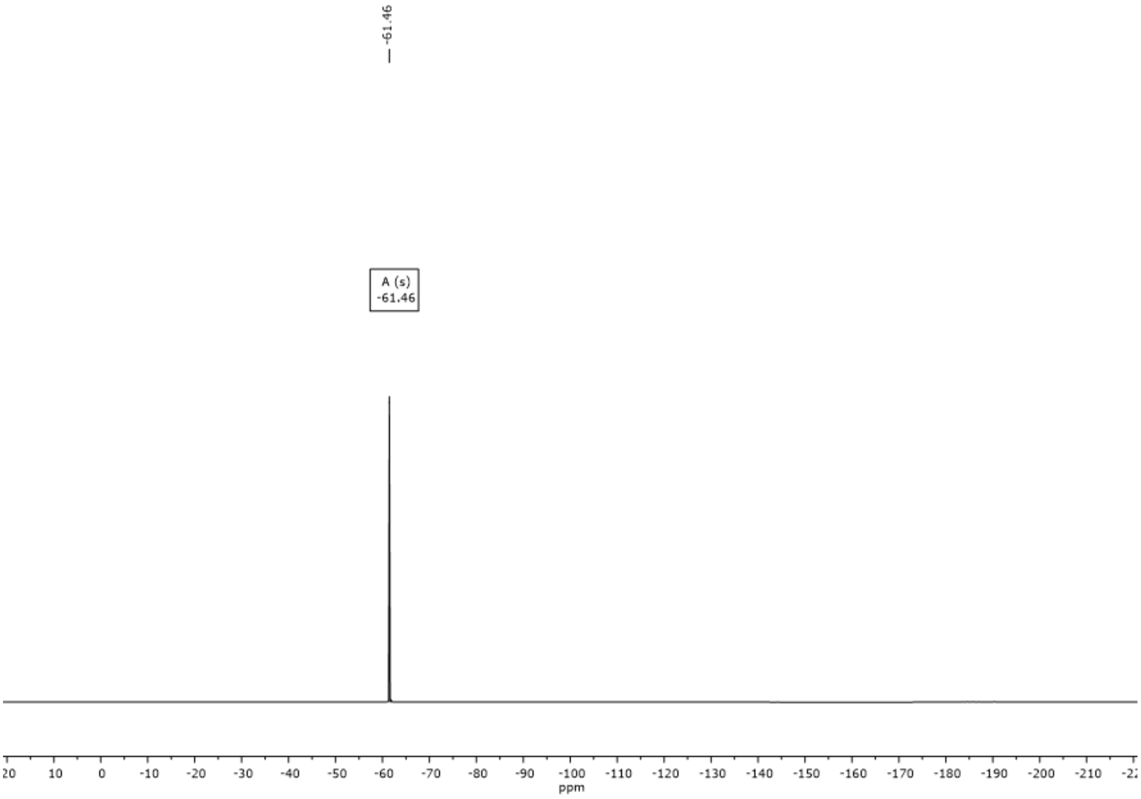

4. Synthesis and characterization of 2,2,2-trichloroethyl isobutylcarbamate (9)

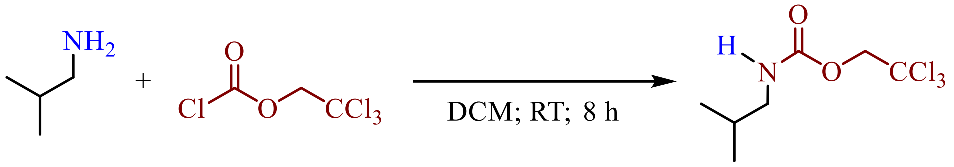

To a 50 mL Schlenk flask under N_2, DCM (25 mL) and 0.35 mL of 2,2,2-trichloroethyl chloroformate chloride (2.5 mmol) were added. The resulting colorless solution was placed in a ice-water bath and 0.65 mL of isobutylamine (6.0 mmol) were added dropwise. Then, the ice-water bath was removed, and the reaction was stirred at rt for 8 h. When the reaction was completed, the resulting white suspension was filtered off and volatiles were removed under vacuum leading to a light-yellow oil. The mixture was washed with a saturated $NaHCO_3$ solution (25 mL), 1 M HCl (25 mL), and brine (25 mL), dried with $MgSO_4$, and concentrated under vacuum to afford the product as a white solid (456 mg; 74%).

^{1}H NMR (400 MHz, $CDCl_3$) δ 5.01 (br s, 1H), 4.73 (s, 2H), 3.07 (t, J = 6.2 Hz, 2H), 1.80 (m, 1H), 0.93 (d, J = 6.6 Hz, 6H) ppm. **^{13}C{^{1}H} NMR** (101 MHz, $CDCl_3$) δ 154.8 (s), 95.8 (s), 74.4 (s), 113.5 (s), 48.7 (s), 28.7 (s), 19.9 (s) ppm. **HRMS-ESI** m/z calculated for $C_7H_{12}Cl_3NO_2Na$ $[M+Na]^+$ 269.9826, found 269.9824 as a higher-intensity signal of a complex isotopic pattern.

^{1}H NMR (400 MHz, $CDCl_3$) spectrum of **9**

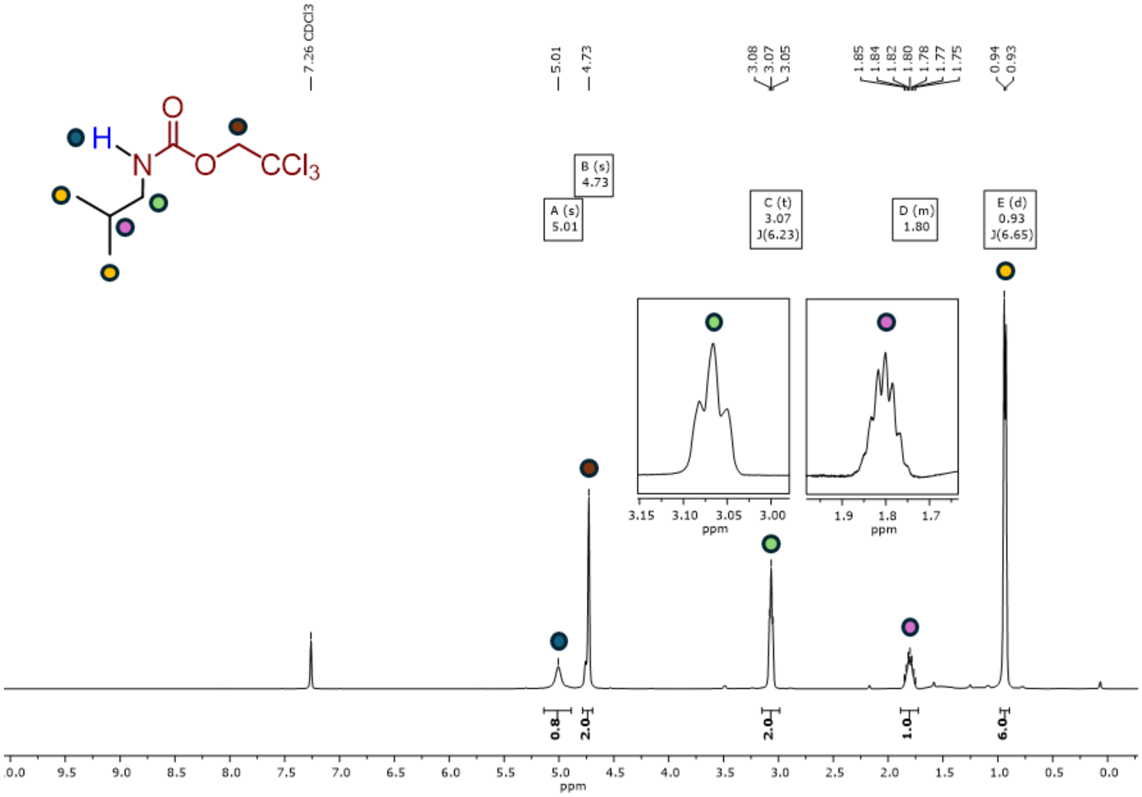

^{13}C {^{1}H} NMR (101 MHz, $CDCl_3$) spectrum of **9**

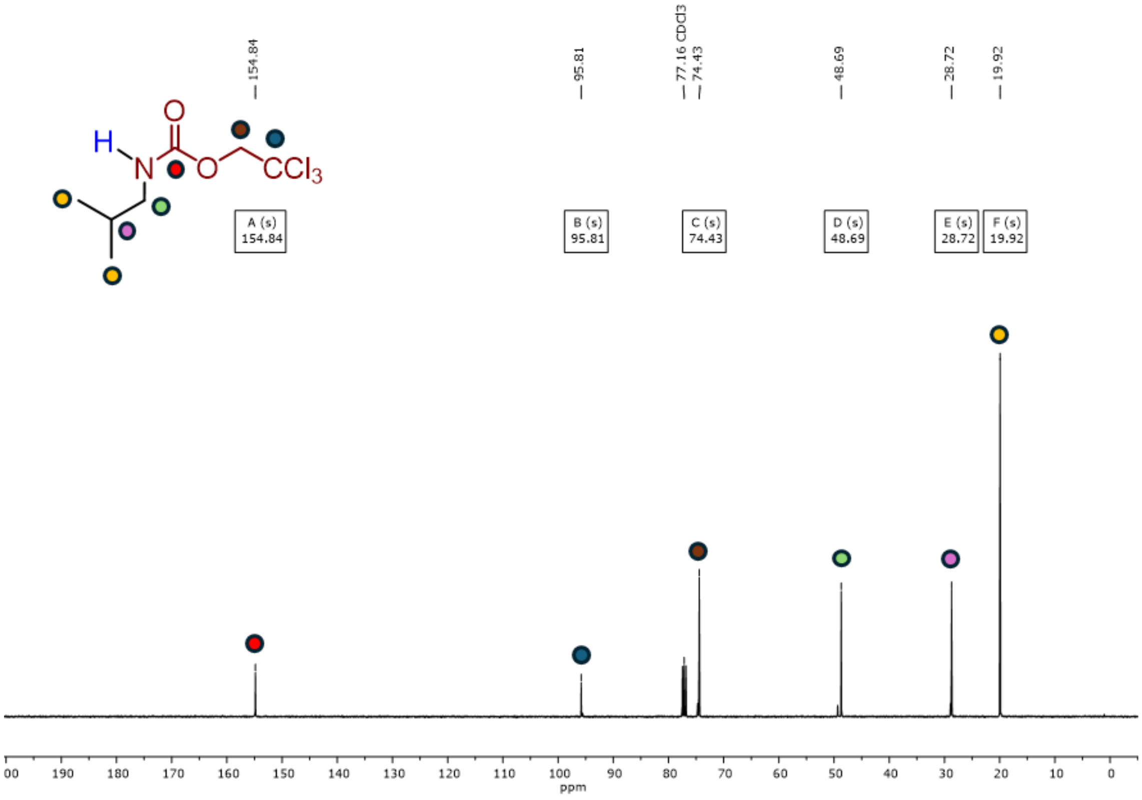

5. Detection of intermediates by HRMS-ESI

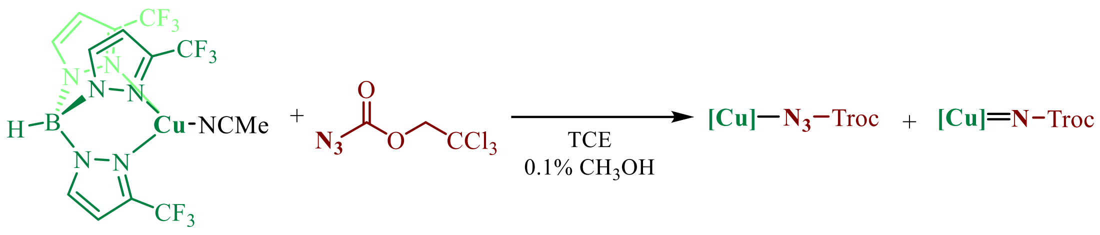

Tp^{CF3}Cu(NCMe) **N_3Troc** **Tp^{CF3}Cu-N_3Troc** **Tp^{CF3}Cu-NTroc**

A 10 mL vial was charged with 5 mL of tetrachloroethane (TCE) and 0.3 mg of Tp^{CF3}Cu(NCMe) (0.5 µmol) leading to a 0.1 mM colorless solution which is subjected to ultrasound at 30 ºC for 10 minutes. Following a similar procedure, a 10.0 mM solution of N_3Troc in TCE (11.0 mg of azide in 5 mL of solvent) is prepared. In a third 10 mL vial, 0.5 mL of the Tp^{CF3}Cu(NCMe) solution, 0.5 mL of the azide solution and 4.0 mL of TCE were added to a final concentration of 0.01 mM for Tp^{CF3}Cu(NCMe) and 1.0 mM for N_3Troc. Finally, 5 µL of MeOH were added and the resulting solution was subjected to an ultrasound bath at 30 ºC for 10 minutes and filtered obtaining a colorless solution which was analyzed by ESI-QTOF (positive mode; direct injection; broadband collision-induced dissociation (bbCID); 70 eV).

The species **Tp^{CF3}Cu-NTroc** and **Tp^{CF3}Cu-N_3Troc** were detected at 670.9040 and 698.9301 m/z respectively as a center of complex isotopic patterns.

HRMS-ESI m/z calculated for $C_{15}H_9BN_7O_2Cl_3F_9Cu$ [**Tp^{CF3}Cu-NTroc**]$^+$ 670.9101, found 670.9040 as a higher-intensity signal of a complex isotopic pattern.

HRMS-ESI m/z calculated for $C_{15}H_9BN_9O_2Cl_3F_9Cu$ [**Tp^{CF3}Cu-N_3Troc**]$^+$ 698.9163, found 698.9301 as a higher-intensity signal of a complex isotopic pattern.

HRMS-ESI spectrum of the mixture of Tp^{CF3}Cu(NCMe) with N_3-Troc (Top: found; Bottom: calculated for the targeted species).

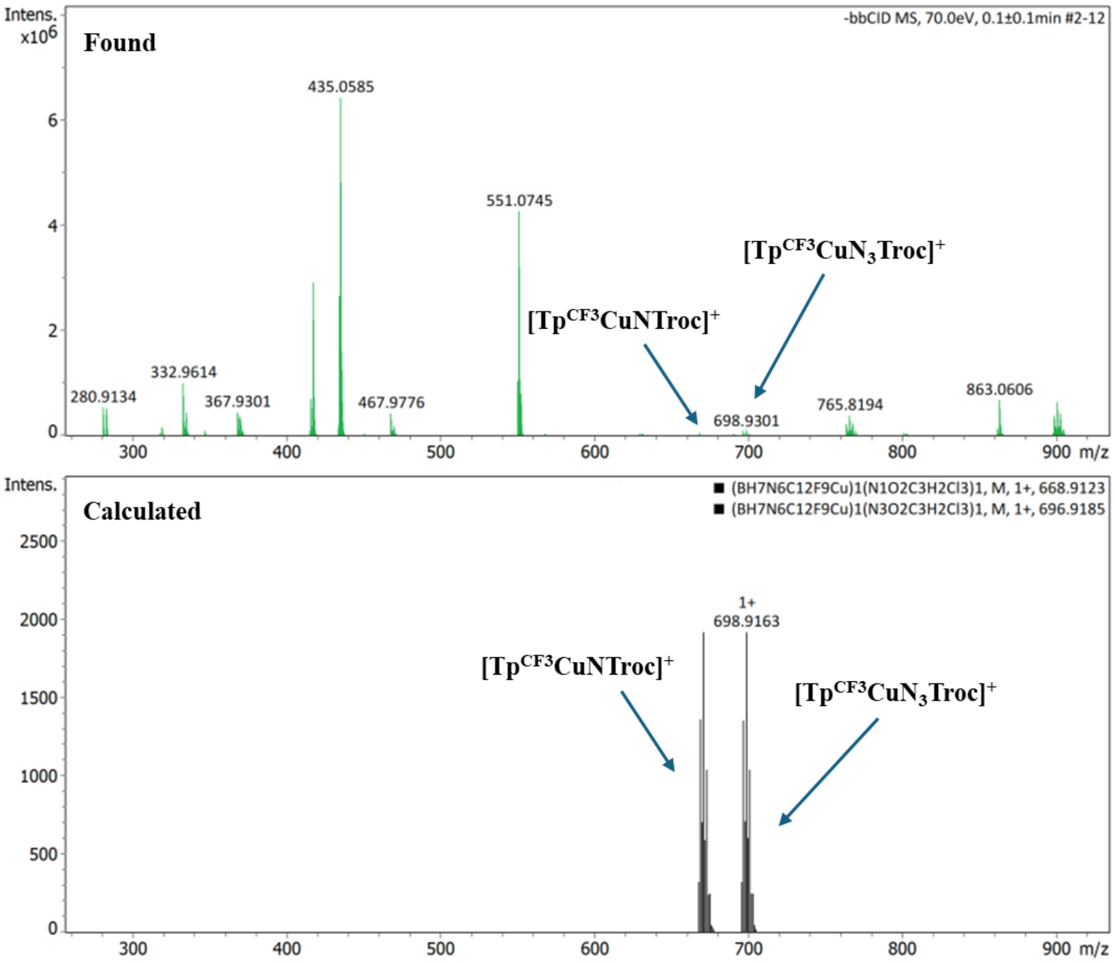

HRMS-ESI spectrum (expanded) of the mixture of $Tp^{CF3}Cu(NCMe)$ with N_3-Troc (Top: found; Bottom: calculated for the targeted species).

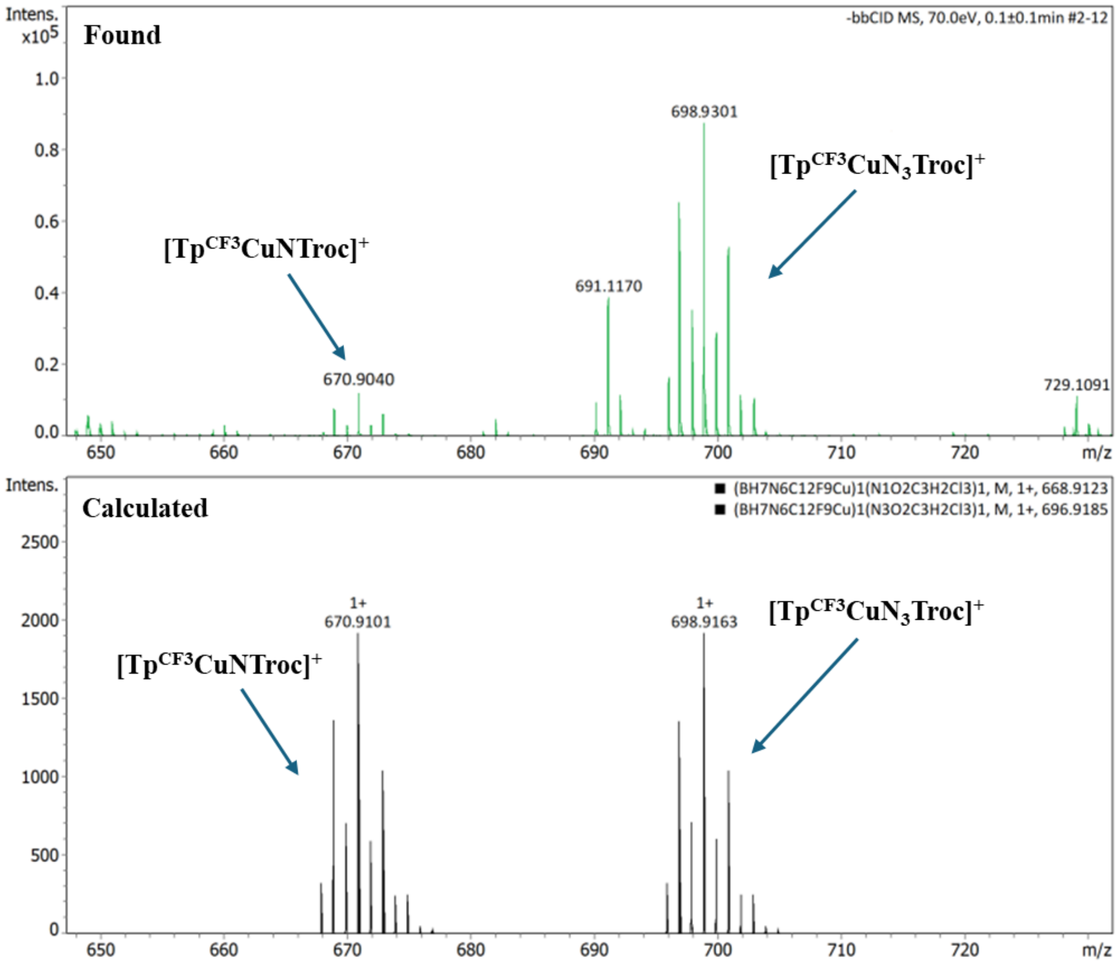

HRMS-ESI spectrum of the mixture of Tp^{CF3}Cu(NCMe) with N$_3$-Troc (Top: found; Bottom: calculated for the targeted species) amplified for [Tp^{CF3}Cu-NTroc]$^+$

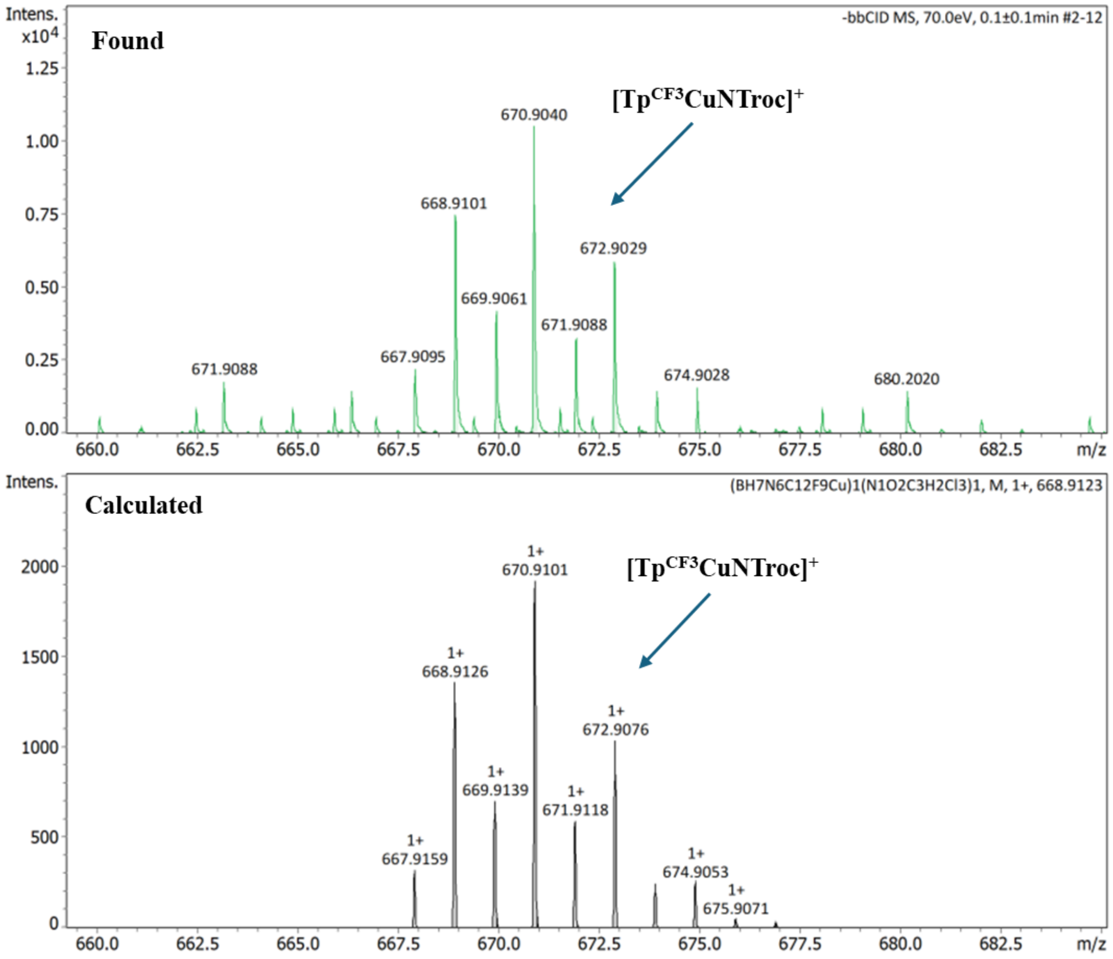

HRMS-ESI spectrum of the mixture of $Tp^{CF3}Cu(NCMe)$ with N_3-Troc (Top: found; Bottom: calculated for the targeted species) amplified for $[Tp^{CF3}Cu\text{-}N_3Troc]^+$

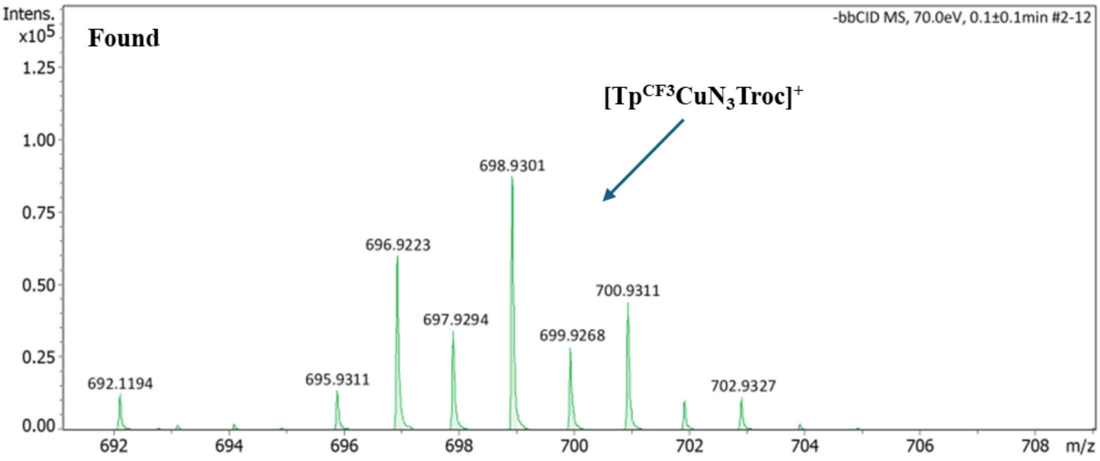

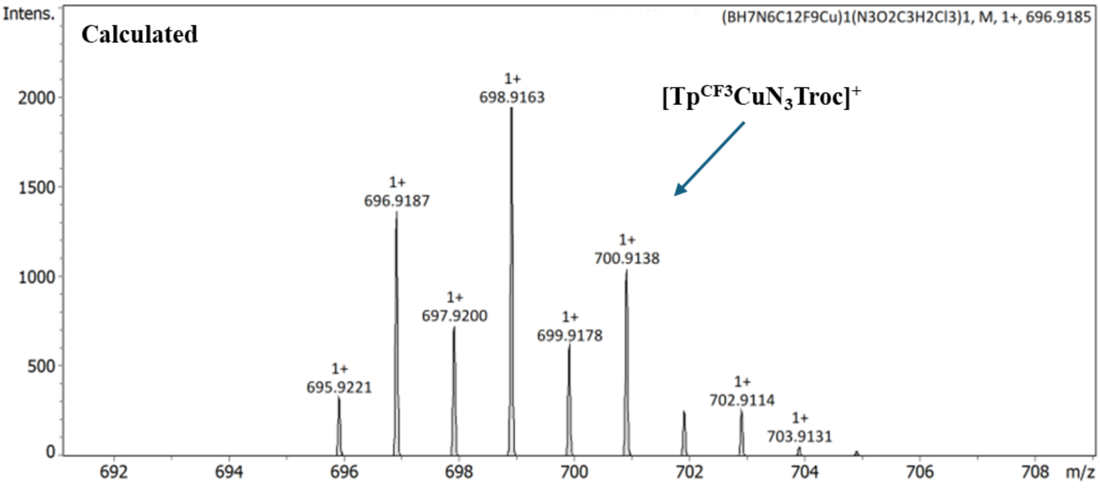

6. Kinetic isotopic effect (KIE) determination experiment

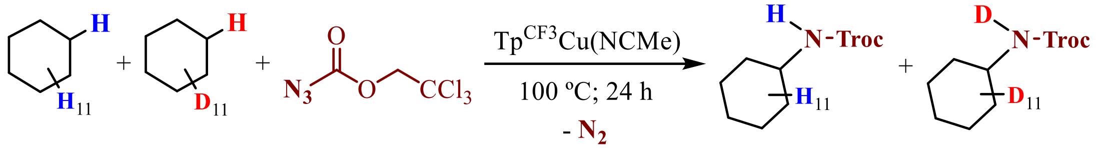

N$_3$-Troc

1 53.3%

1-d$_{12}$ 25.4%

KIE = 2.1

Following the general procedure for liquid alkanes, the experiment was carried out employing 0.006 mmol of Tp^{CF3}Cu(NCMe) (10%), 1 mL of dry cyclohexane, 1 mL of dry perdeuterated cyclohexane and 0.06 mmol of N$_3$-Troc at 100 ºC for 24 h. Yields were determined by GC and ^{1}H NMR analysis employing mesitylene as internal standard and identified by its comparison with literature precedents.

^{1}H NMR spectrum (400 MHz; CDCl$_3$) for the KIE determination experiment

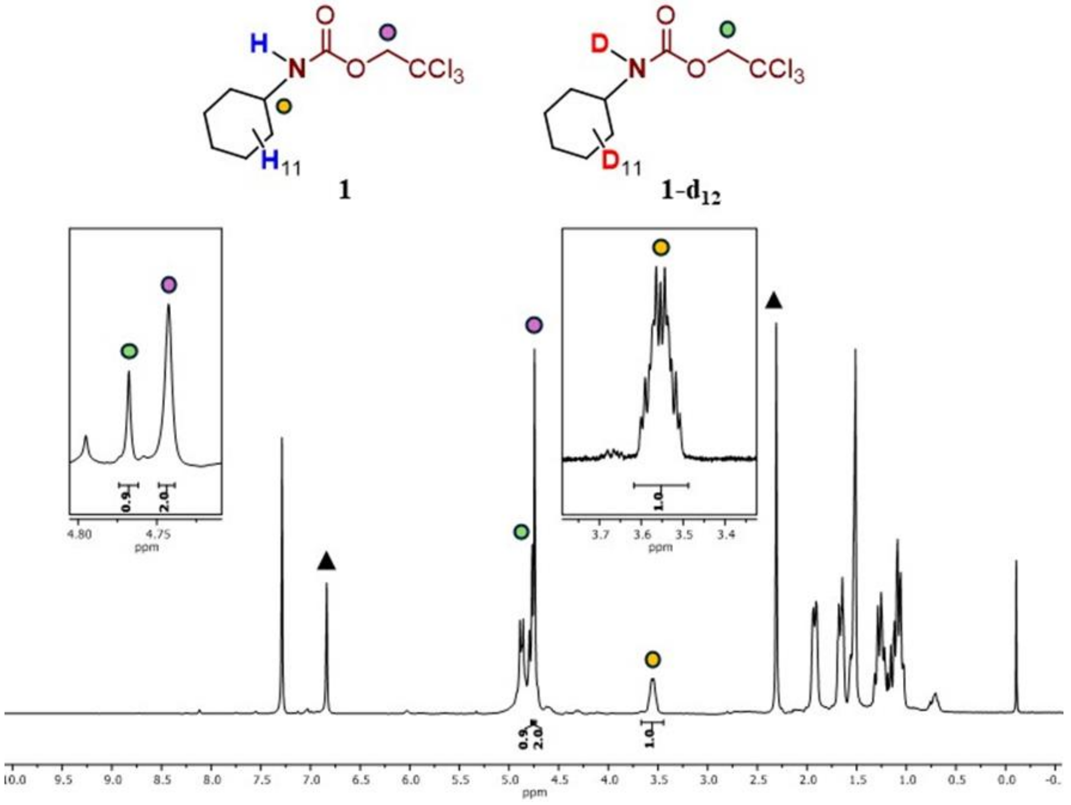

7. Reaction with cyclohexane adding BHT as radical inhibitor

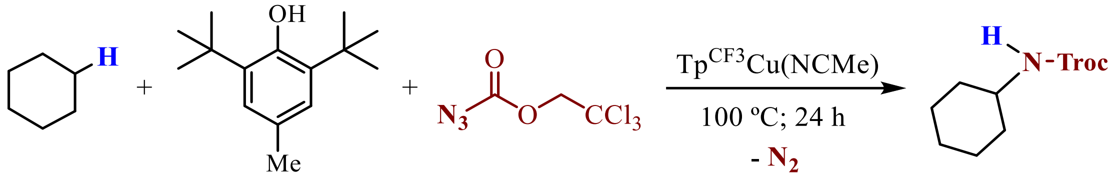

N3-Troc **1**

<1%

Following the general procedure for liquid alkanes, the experiment was carried out employing 0.013 mmol of Tp^{CF3}Cu(NCMe) (10%), 5 mL of dry cyclohexane, 0.13 mmol of 3,5-di-tert-4butylhydroxytoluene (BHT) and 0.13 mmol of N_3-Troc at 100 ºC for 24 h. Yields were determined by ^{1}H NMR analysis employing mesitylene as internal standard and identified by its comparison with literature precedents.

^{1}H NMR spectrum (400 MHz; $CDCl_3$) for the reaction with BHT

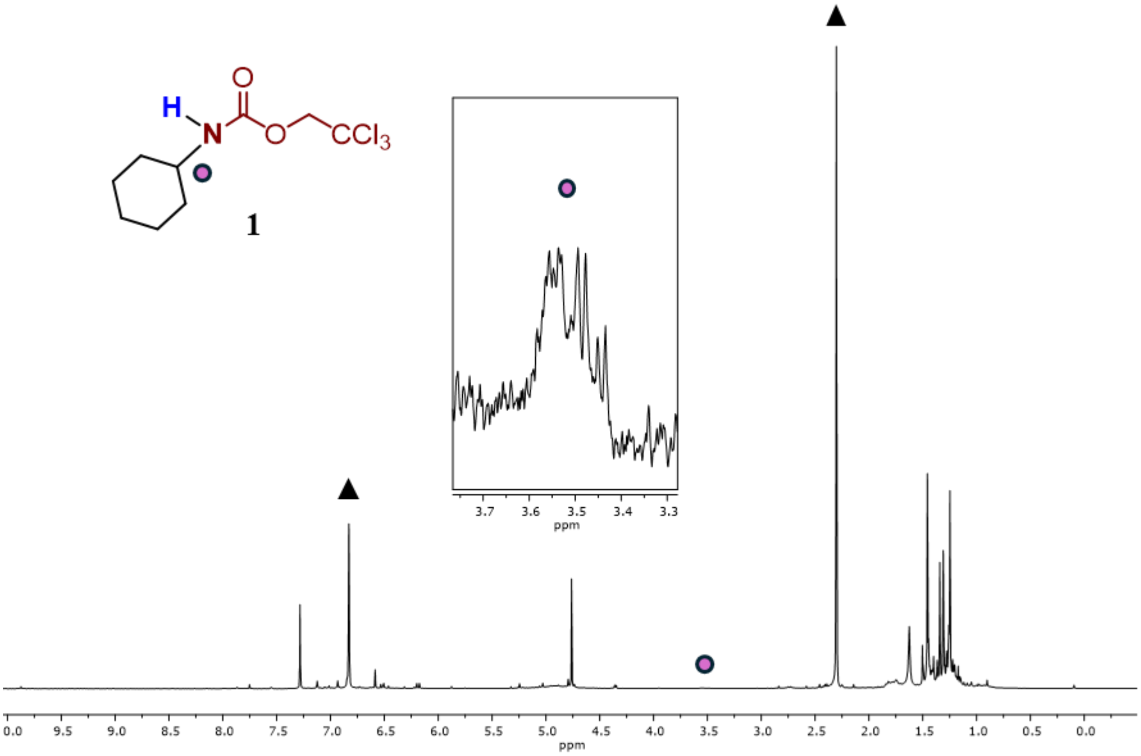

8. Reaction in presence of CCl_4 as radical trap

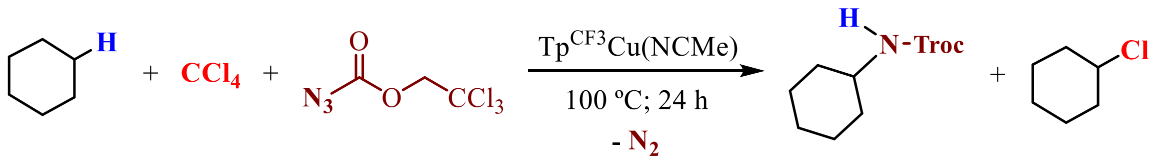

N$_3$-Troc | 1 | 10

41.1% | 12.8%

Following the general procedure for liquid alkanes, the experiment was carried out employing 0.0013 mmol of Tp^{CF3}Cu(NCMe) (10%), 4 mL of dry cyclohexane, 1 mL of dry carbon tetrachloride and 0.13 mmol of N_3-Troc at 100 ºC for 24 h. Yields were determined by ^{1}H NMR analysis employing mesitylene as internal standard and identified by its comparison with literature precedents.

^{1}H NMR spectrum (400 MHz; $CDCl_3$) for the reaction in presence of CCl_4

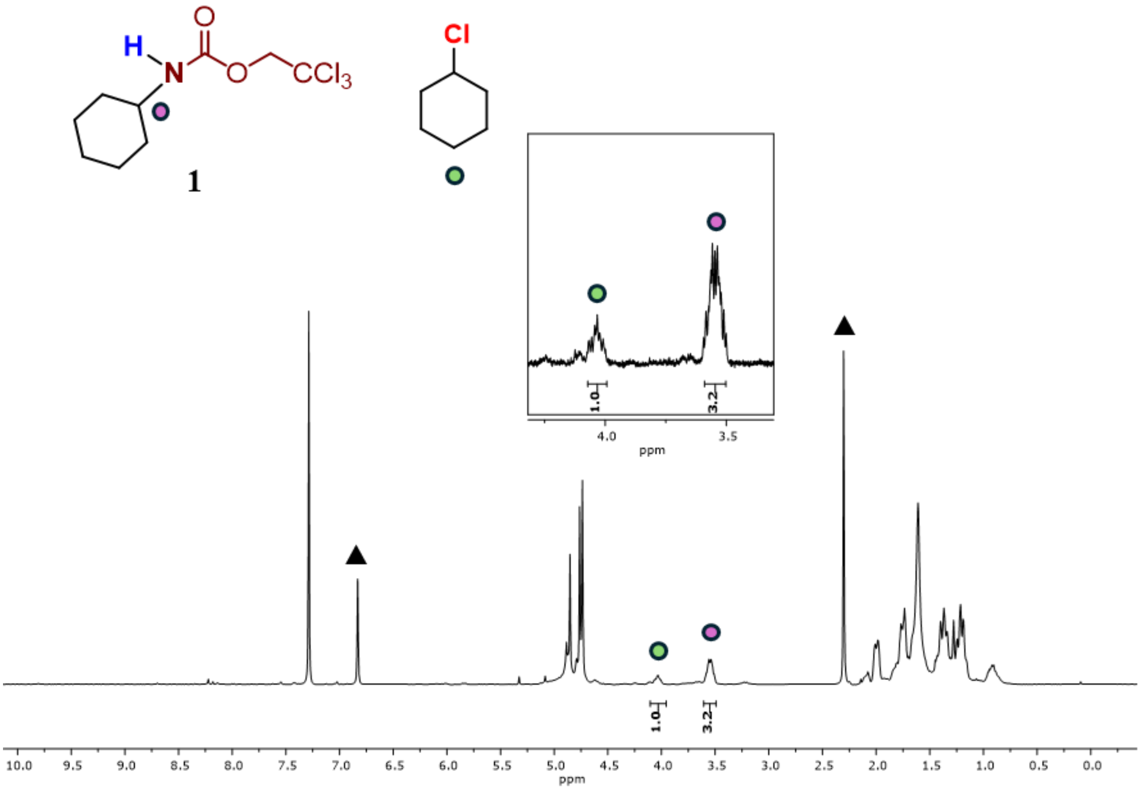

9. Kinetic study: reaction with methane in $scCO_2$

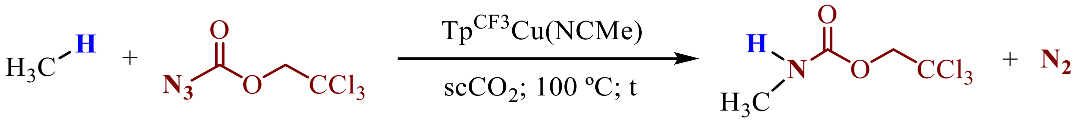

Methane **N_3-Troc** **2**

Following the general procedure for gaseous alkanes, the experiment was carried out employing 0.018 mmol of $Tp^{CF3}Cu(NCMe)$ (10%), 0.183 mmol of N_3-Troc and 160 bar of methane in $scCO_2$ at 100 ºC for the indicated time. Yields were determined by ^1H NMR analysis employing mesitylene as internal standard and identified by its comparison with literature precedents.

Table S4. *Results obtained in the functionalization of methane in $scCO_2$ at different times.*

Entry	Time (h)	Yield (%)[a]
**1**	3	2.0
**2**	6	4.1
**3**	10	7.9
**4**	16	10.3
**5**	24	14.5
**6**	48	19.7
**7**	72	21.8
**8**	100	22.0

[a]Yields are referred to initial azide.

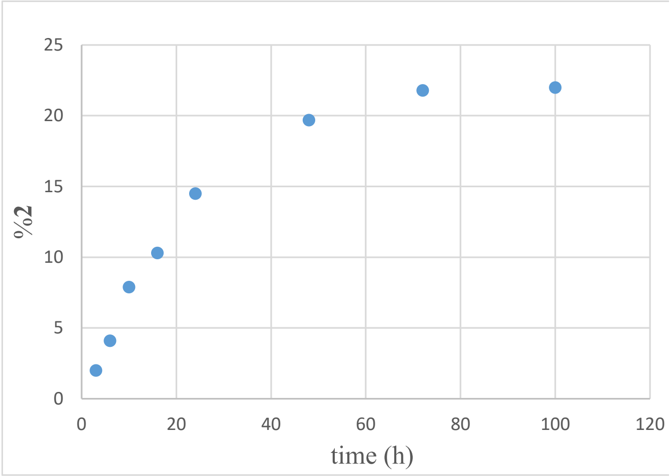

10. Study of the effect of pressure in the reaction with methane in $scCO_2$

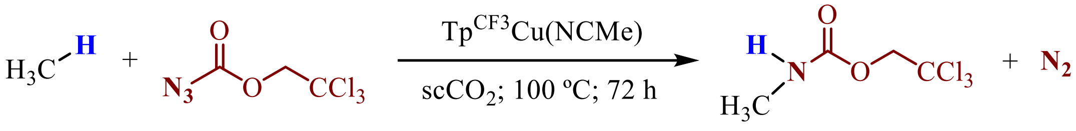

Methane
0-160 bar

N₃-Troc

2

Following the general procedure for gaseous alkanes, the experiment was carried out employing 0.018 mmol of $Tp^{CF3}Cu(NCMe)$ (10%), 0.183 mmol of N_3-Troc and the indicate pressure of methane in $scCO_2$ at 100 ºC for 72 hours. Yields were determined by ^{1}H NMR analysis employing mesitylene as internal standard and identified by its comparison with literature precedents.

Table S5. *Results obtained in the functionalization of methane in $scCO_2$ at different pressures.*

Entry	Pressure CH_4 (bar)	mmol CH_4	Total pressure at 100 ºC (bar)	Yield (%)[a]
**1**	0	0	220	n.d.
**2**	50	113	290	5.4
**3**	100	210	350	15.9
**4**	160	309	415	21.8

[a]Yields are referred to initial azide.

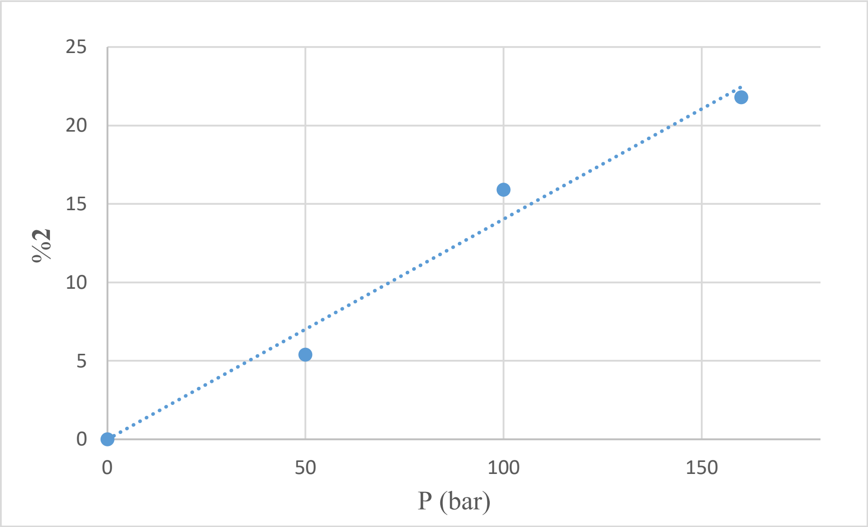

11. Competition experiment: cyclohexane vs methane

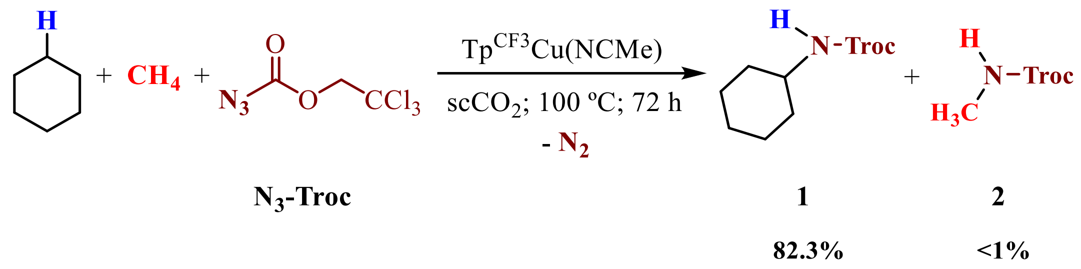

Following the general procedure for gaseous alkanes, the experiment was carried out employing 0.018 mmol of $Tp^{CF3}Cu(NCMe)$ (10%), 0.183 mmol of N_3-Troc in 10 mL of cyclohexane (92 mmol C_6H_{12}) and 160 bar of methane (260 mmol CH_4) in $scCO_2$ at 100 ºC for 72 hours. Yields were determined by ^1H NMR analysis employing mesitylene as internal standard and identified by its comparison with literature precedents.

^{1}H NMR spectrum (400 MHz; $CDCl_3$) for the competition reaction

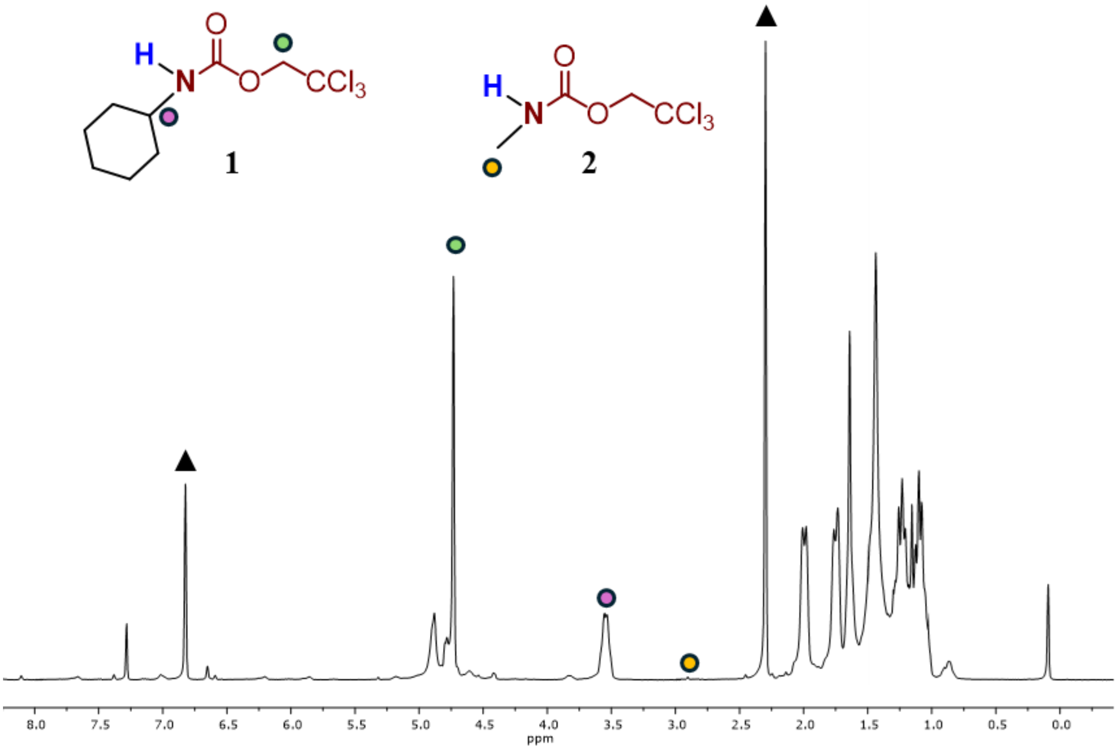

12. NMR spectra for catalytic experiments

12. a. ^{1}H NMR spectrum ($CDCl_3$; 400 MHz) for the functionalization of cyclohexane employing $Tp^{CF3}Cu(NCMe)$ as catalyst (mesitylene ▲ added as internal standard)

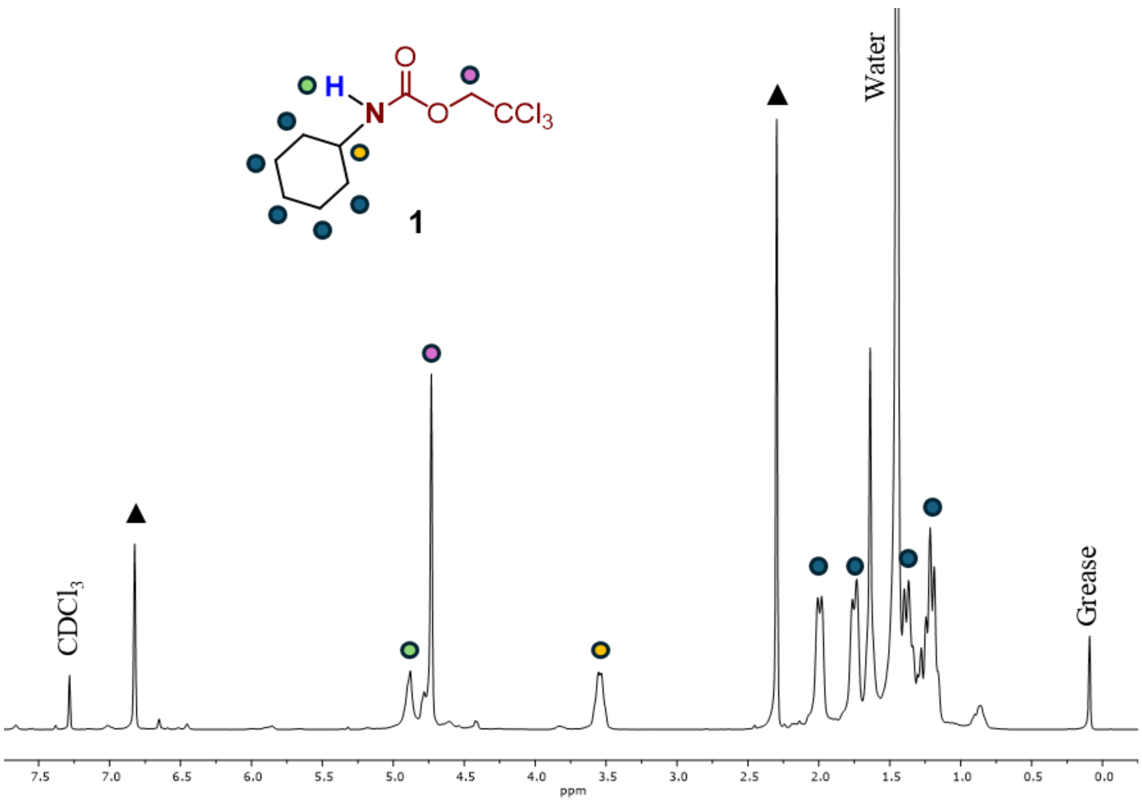

12. b. ^{1}H NMR spectrum (CDCl$_3$; 400 MHz) for the functionalization of methane (scCO$_2$ as solvent) employing Tp^{CF3}Cu(NCMe) as catalyst (mesitylene▲ added as internal standard)

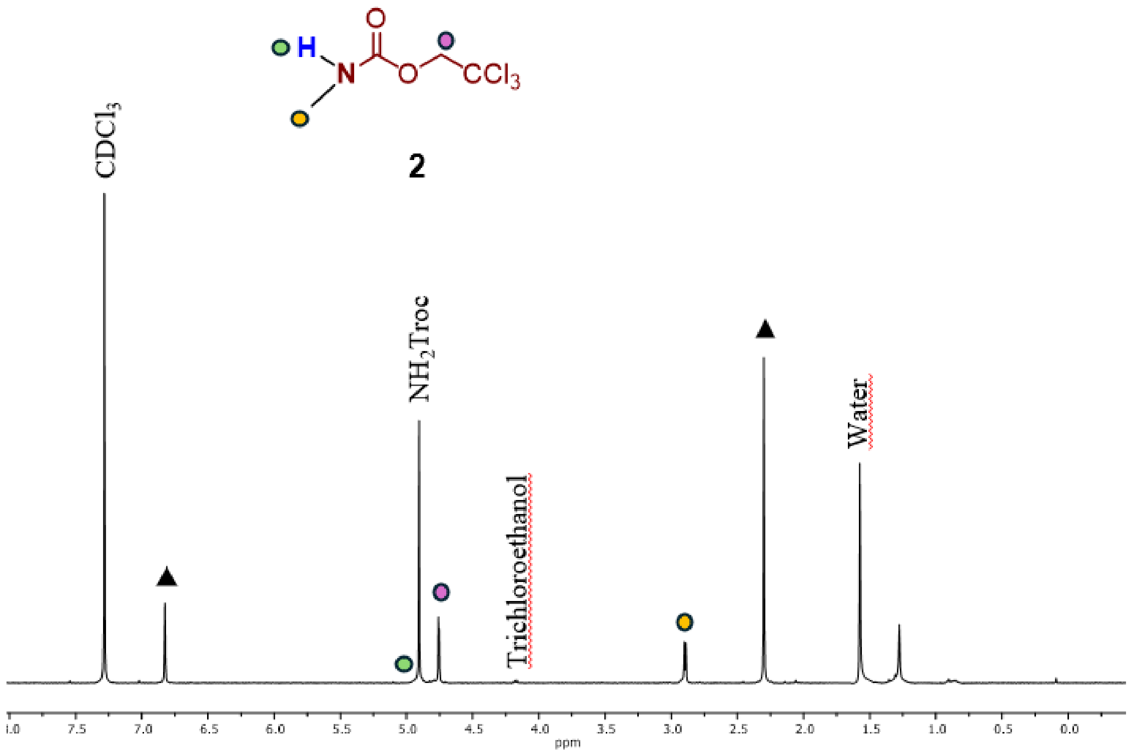

12. c. ¹H NMR spectrum ($CDCl_3$; 400 MHz) for the functionalization of ethane ($scCO_2$ as solvent) employing Tp^{CF3}Cu(NCMe) as catalyst (mesitylene▲ added as internal standard)

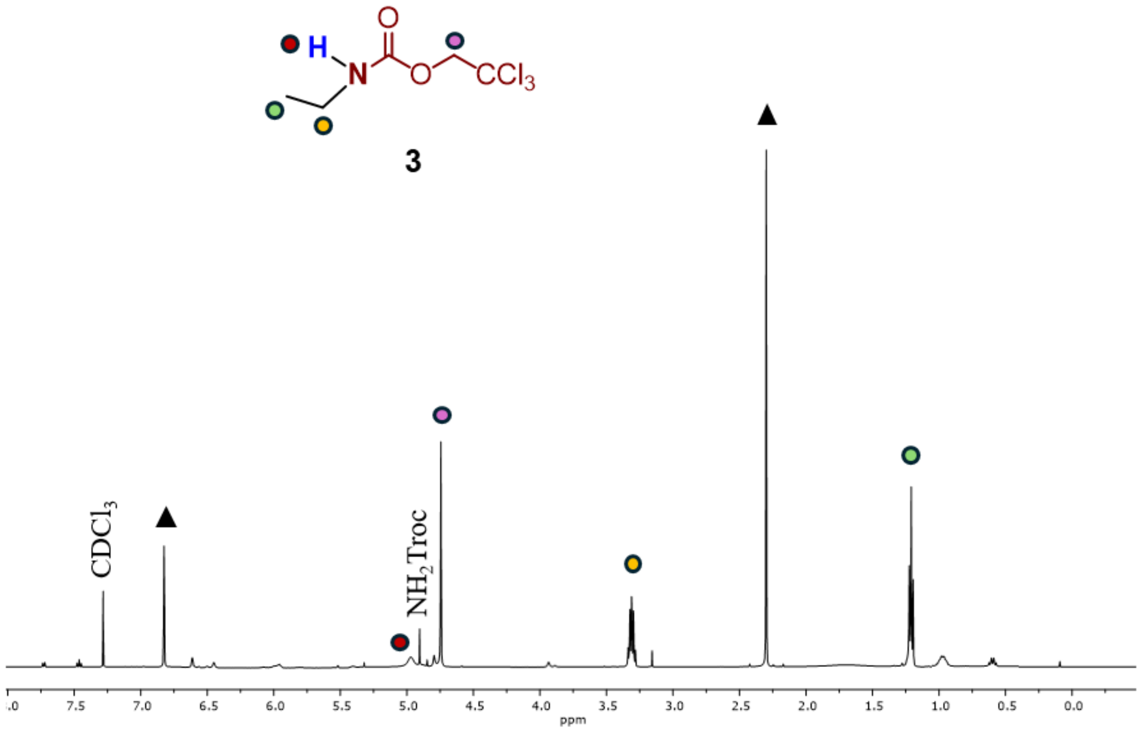

12. d. ^{1}H NMR spectrum ($CDCl_3$; 400 MHz) for the functionalization of propane ($scCO_2$ as solvent) employing Tp^{CF3}Cu(NCMe) as catalyst (mesitylene▲ added as internal standard)

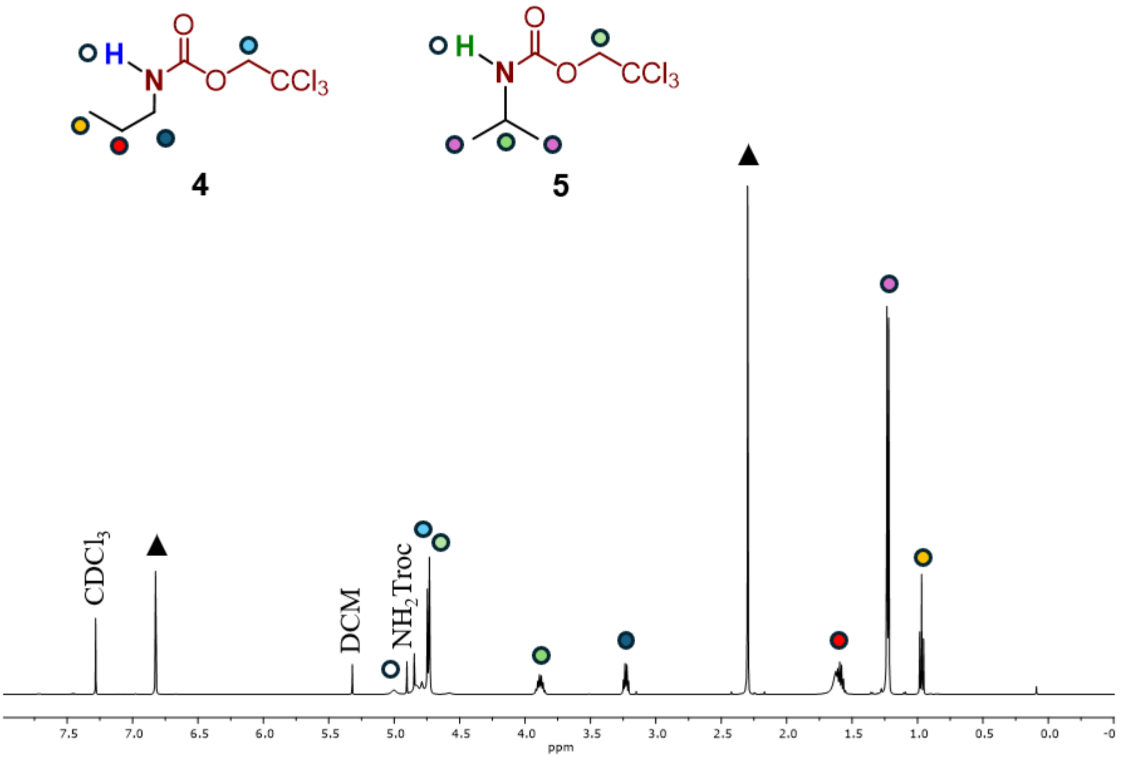

12. e. ^{1}H NMR spectrum ($CDCl_3$; 400 MHz) for the functionalization of n-butane ($scCO_2$ as solvent) employing $Tp^{CF3}Cu(NCMe)$ as catalyst (mesitylene▲ added as internal standard)

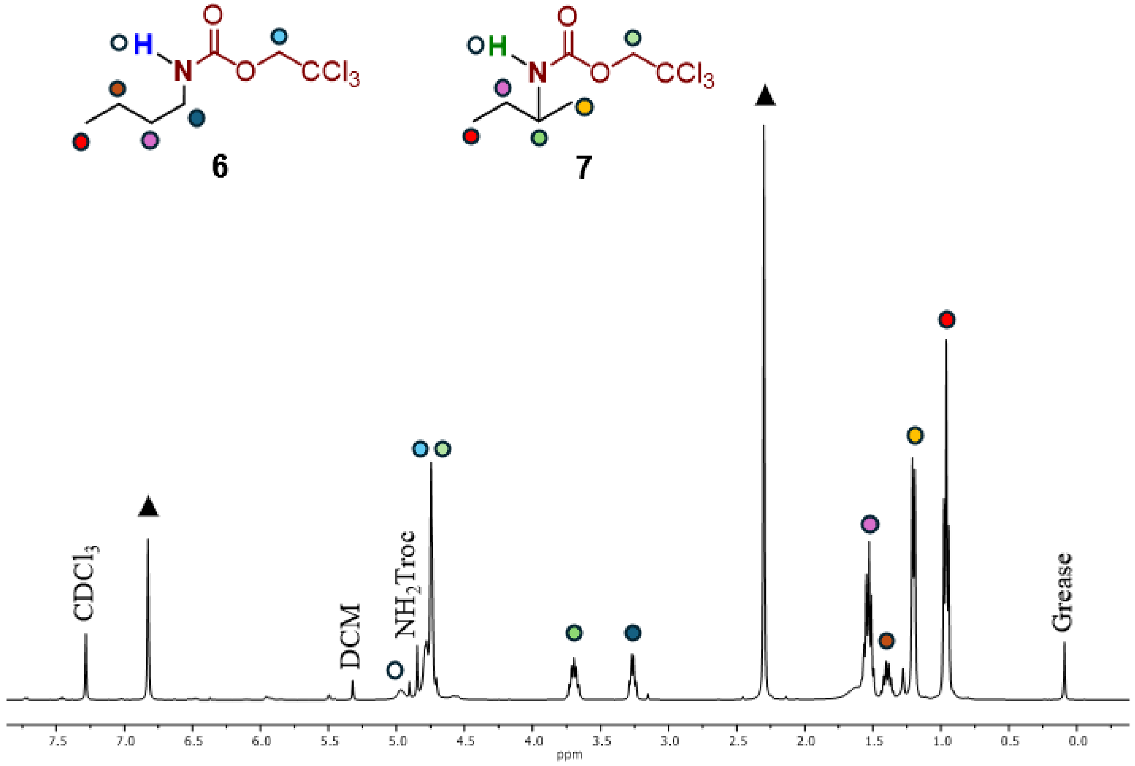

12. f. ^{1}H NMR spectrum ($CDCl_3$; 400 MHz) for the functionalization of isobutane ($scCO_2$ as solvent) employing Tp^{CF3}Cu(NCMe) as catalyst (mesitylene ▲ added as internal standard)

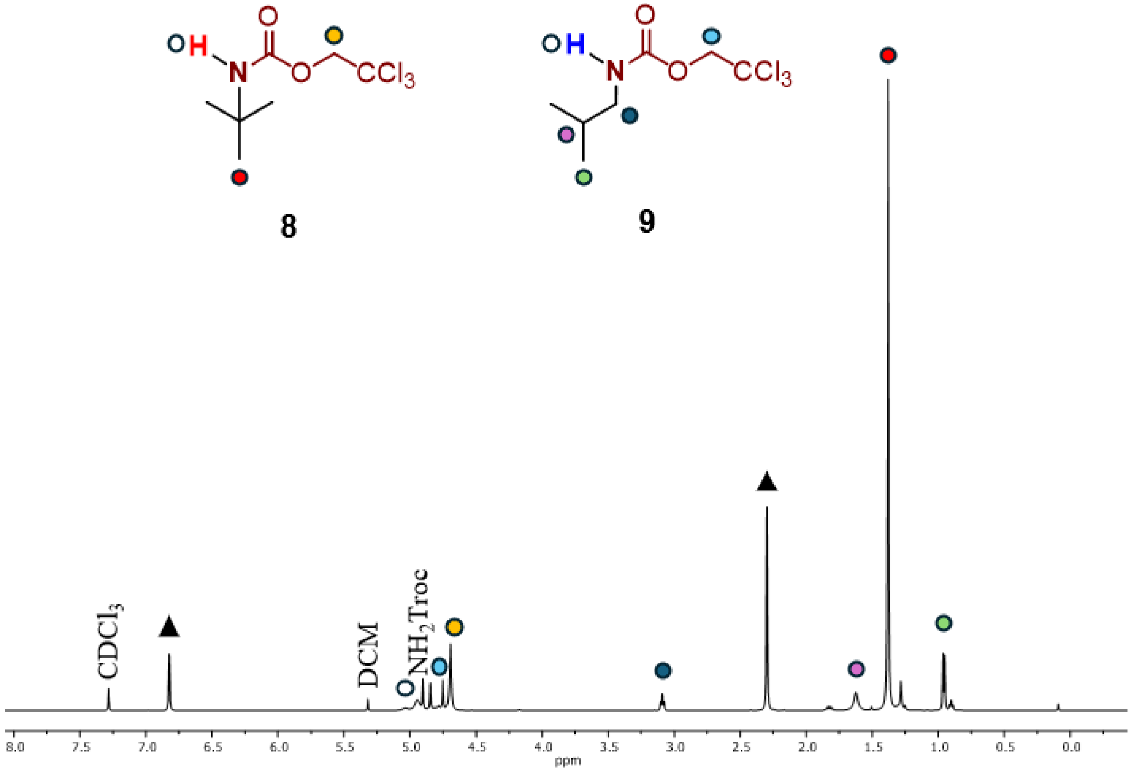

13. Computational details

Geometries presented along the computational study, were optimized using Gaussian 16[14] with the B3LYP-D3 functional[15,16] which includes Grimme's dispersion correction. The 6-31g(d)[17] basis sets were used for all atoms except for the heavier elements (F, Cl, and Cu) for which the LANL2DZ[18] basis sets were used instead, halogen atoms were supplemented with extra d and p functions[19] extracted from Basis Set Exchange.[20] All calculations included the implicit simulation of the solvent environment through a continuum model, namely SMD[21] with the default parameters for heptane as an approach for the simulation of supercritical CO_2. HRMS-ESI detection was done using Tetrachloroethane (TCE) as solvent, to reproduce these conditions some species where optimized considering TCE as solvent within SMD, using Gaussian default parameters for 1,1,2,- trichloroethane and changing the dielectric constant to $\varepsilon = 8.42$ through the *eps* keyword. The potential energies were further refined by performing single point (SP) calculations with the larger valence triple-zeta polarization basis sets def2-TZVP[22] for all light atoms and def2-QZVP[23] for F, Cl and Cu. Both singlet and triplet Potential Energy Surfaces (PESs) were carefully explored and Minimum Energy Crossing Points (MECPs) between singlet and triplet PESs searched with the program developed by Prof. J.N. Harvey[24] together with the easyMECP utility.[25] Gibbs free energies for MECPs were approximated to the average between singlet and triplet using projected frequencies. The program goodvibes[26] was used to perform the standard state correction (to change from the standard state in gas phase, 1 atm, to the standard state in solution, 1 M), to perform the Grimme's quasi-harmonic approximation for low frequencies,[27,28] and to obtain free energies at 303.15, 353.15 and 373.15 K from computed free energies at 298.15 K. For the evaluation of the Kinetic Isotope Effect, frequencies of substrates and key transition states were run using the Gaussian keyword (Iso=2) on key atoms. All energies presented correspond to Gibbs free energies in solution and in kcal·mol^{-1}. To explore the conformational complexity of the studied species conformational searches were performed both using the Conformer-Rotamer Ensemble Sampling Tool CREST developed by Grimme and co-workers[29] and performing manual explorations, in order of sampling a large number of conformers for the key transition states. A data set collection of computational results is available in the ioChem-BD repository[30] and can be accessed via https://iochem-bd.urv.es/browse/handle/100/2265 .

For details about the construction of the microkinetic model, please see Microkinetic Model section within this document.

As mentioned above, Minimum Energy Crossing Points (MECPs) were located at different points of the PES. They are represented in the Free Energy Profiles as grey crosses (Scheme 4 and Figures S3 to S9). These crosses represent the lowest energy point in which the singlet and triplet free energies meet in that region of the PES, they are points with same potential energy and geometry in the singlet and triplet surfaces. To clarify the simplified representation, Figure S2 shows in more detail the points where the free energy surfaces cross. **MECP3$_{N}$** and **MECP3$_{ON}$** (Figure S2, a and b), are MECPs that allow to cross between surfaces at a geometry close to minima where the singlet and triplet structures are similar. **MECP4-5** (Figure S2, c) is different than the other two as in this case the MECP connects minima that are not similar. Connects the triplet **Int4$_{ON}^{T}$**, with unpaired electrons on copper and carbon, with the singlet **Int5$_{N}^{S}$**, where the N-C bond is formed. This MECP has a role similar to a transition state but changing spin states.

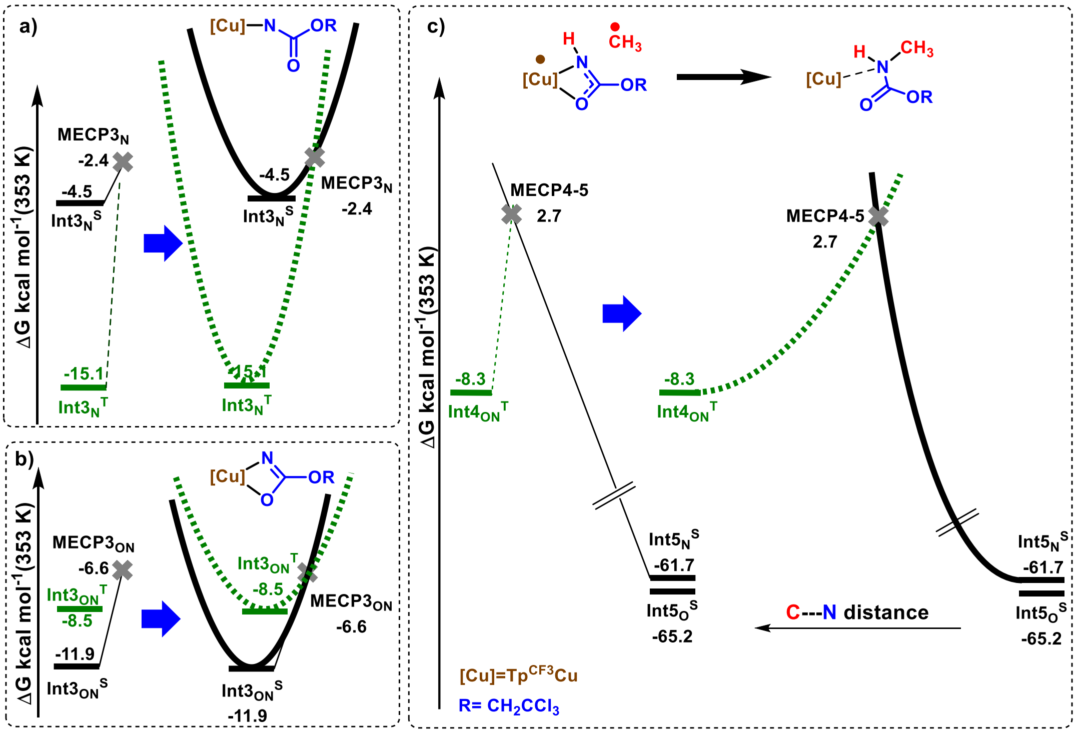

Figure S2. *Minimum Energy Crossing Points (MECPs) on the Free energy profile for methane amidation (353.15 K), in kcal·mol^{-1}. In black and grey solid lines the singlet surface is presented, the triplet surface is represented in dark and sea green dashed lines instead.*

14. Free energy profiles for methane, ethane, propane and iso-butane amidation

14. a. Methane amidation

The computationally postulated mechanism for the amidation of methane is presented in Scheme 4 of the main text at 353.15 K. [Cu] stands for the Tp^{CF3}Cu core along the calculations. The corresponding free energy profile at 373.15 K is presented in Figure S3.

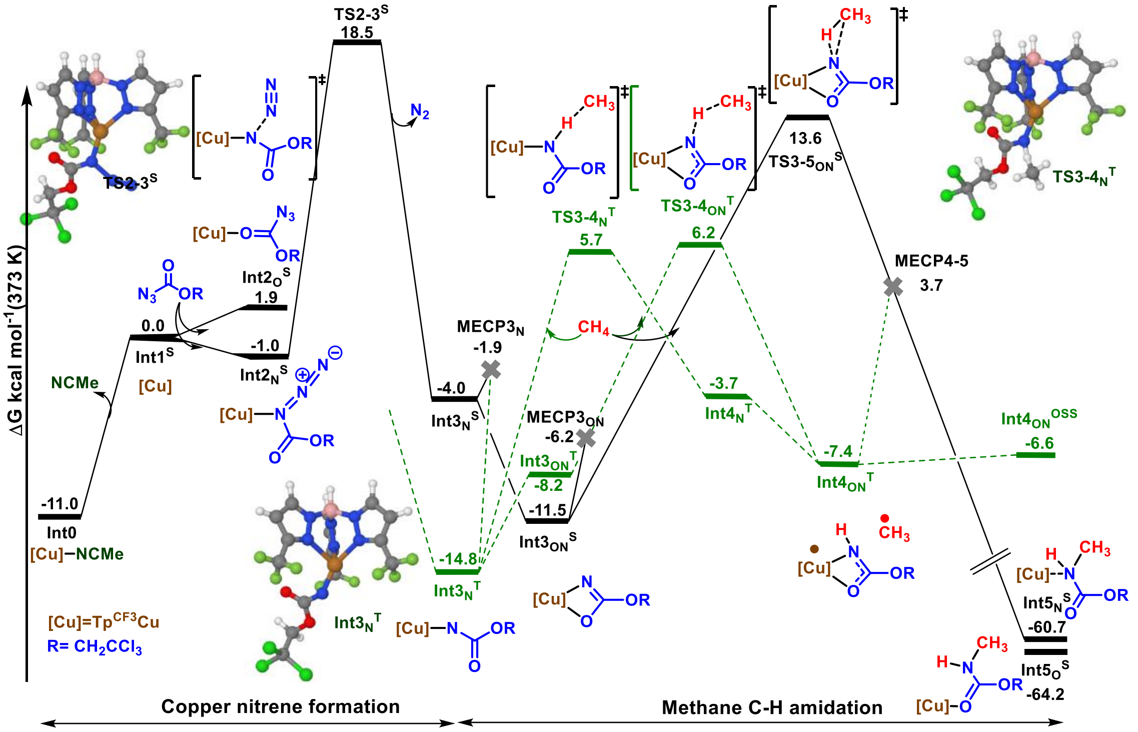

Figure S3. *Free energy profile for methane amidation (373.15 K), in kcal·mol*$^{-1}$*. In black and grey solid lines the singlet surface is presented, the triplet surface is represented in dark and sea green dashed lines instead. Grey crosses represent Minimum Energy Crossing Points (MECPs).*

14. b. Ethane amidation

The computationally postulated mechanism for the amination of ethane from the triplet nitrene intermediate **Int3$_N$^{T}** is presented in **Figure S4** (353.15 K) and **Figure S5** (373.15 K). [Cu] stands for the Tp^{CF3}Cu core along the calculations. Formation of the copper nitrene **Int3$_N$^{T}** is the same as for methane and is presented in Scheme 4 of the main text (left) and in Figure S3 (left).

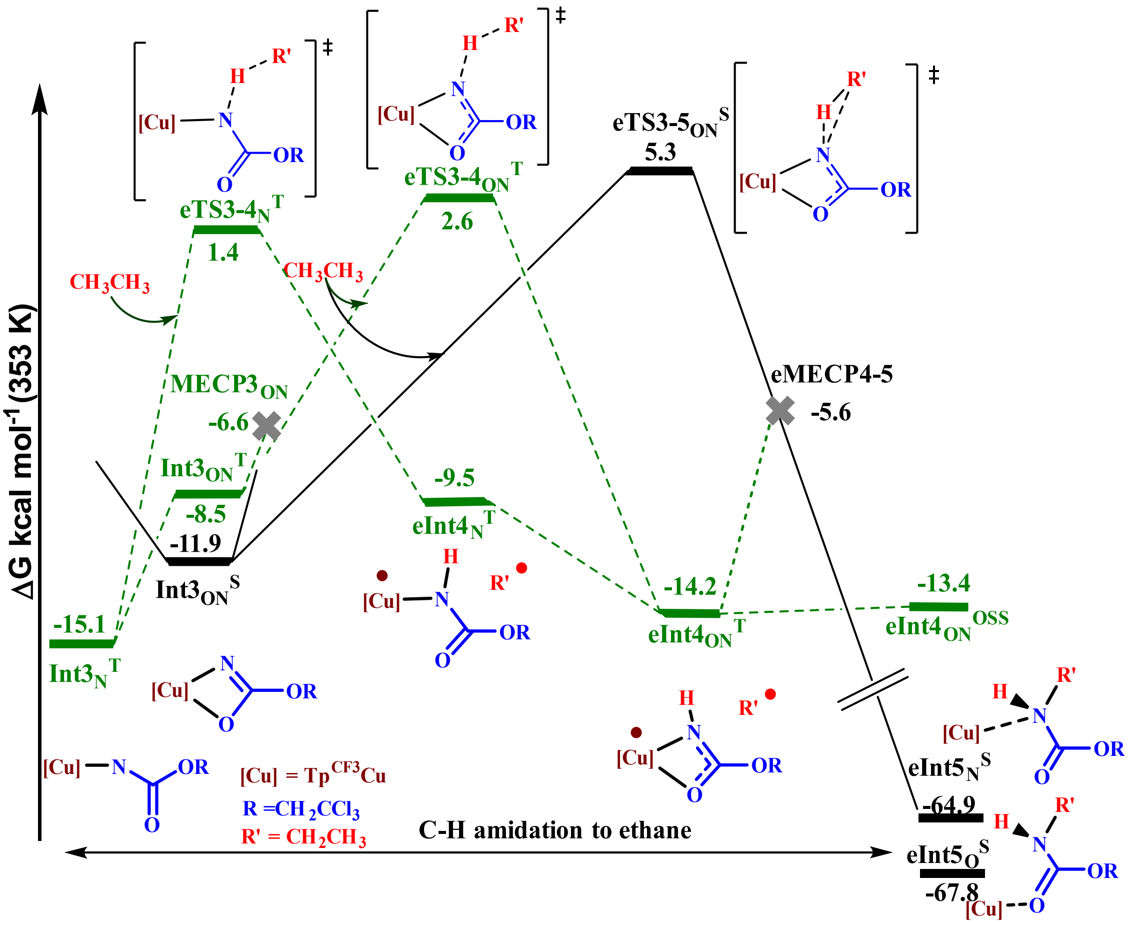

Figure S4. *Free energy profile for ethane amidation (353.15 K), in kcal·mol^{-1}. In black solid lines the singlet surface is presented, the triplet surface is represented in green dashed lines instead. Grey crosses represent Minimum Energy Crossing Points (MECPs).*

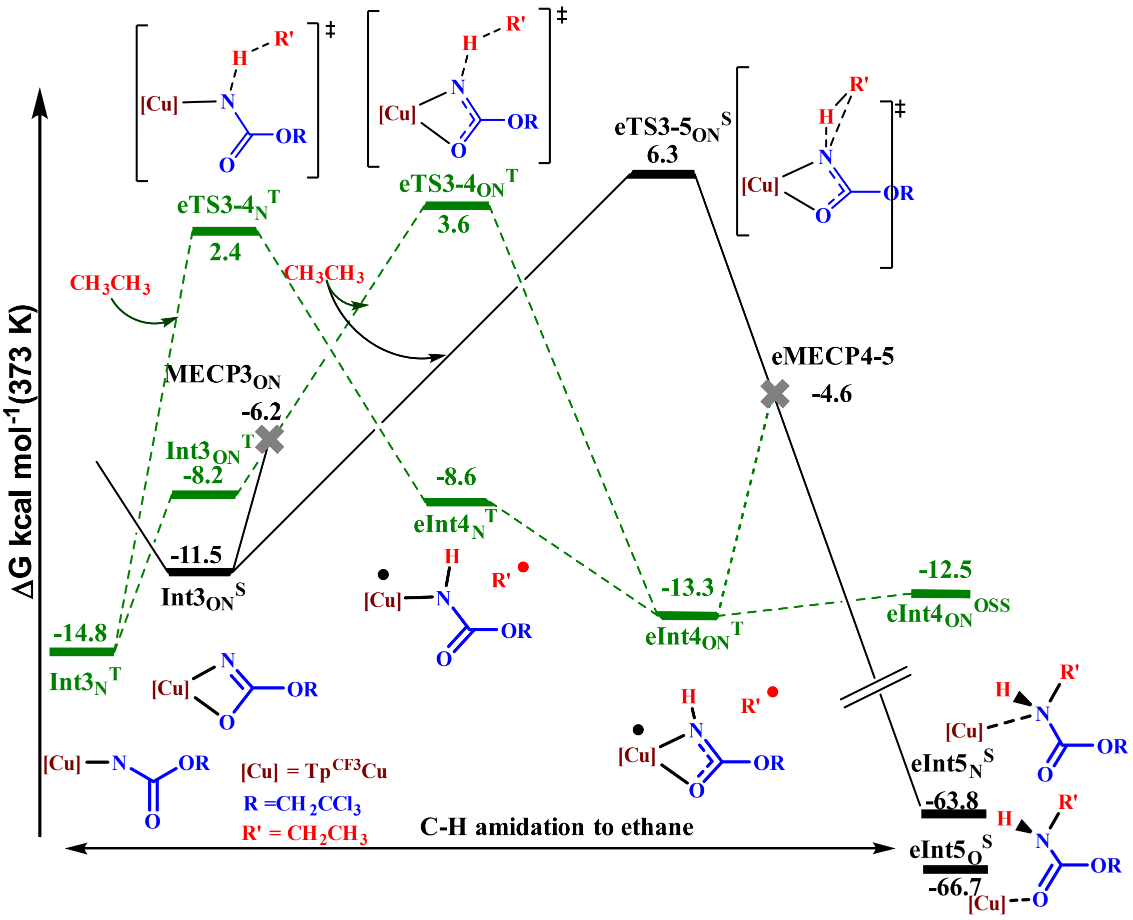

Figure S5. *Free energy profile for ethane amidation (373.15 K), in kcal·mol^{-1}. In black solid lines the singlet surface is presented, the triplet surface is represented in green dashed lines instead. Grey crosses represent Minimum Energy Crossing Points (MECPs).*

14. c. Propane amidation

The computationally postulated mechanism for the amidation of propane from the triplet nitrene intermediate **Int3$_N$^{T}** is presented in **Figure S6** (353.15 K) and **Figure S7** (373.15 K). Formation of the copper nitrene **Int3$_N$^{T}** is the same as for methane and is presented in Scheme 4 of the main text (left) and in Figure S3 (left). Propane presents to types of CH bonds: terminal (primary) and internal (secondary). Minima and transition states on the reaction path leading to the amidation of terminal (primary) CH bonds have the letters "tp" in front of the species name, standing for "terminal propane". Minima and transition states on the path leading to the amidation of the internal (secondary) CH bonds have letters "ip" in front of the species name, standing for "internal propane".

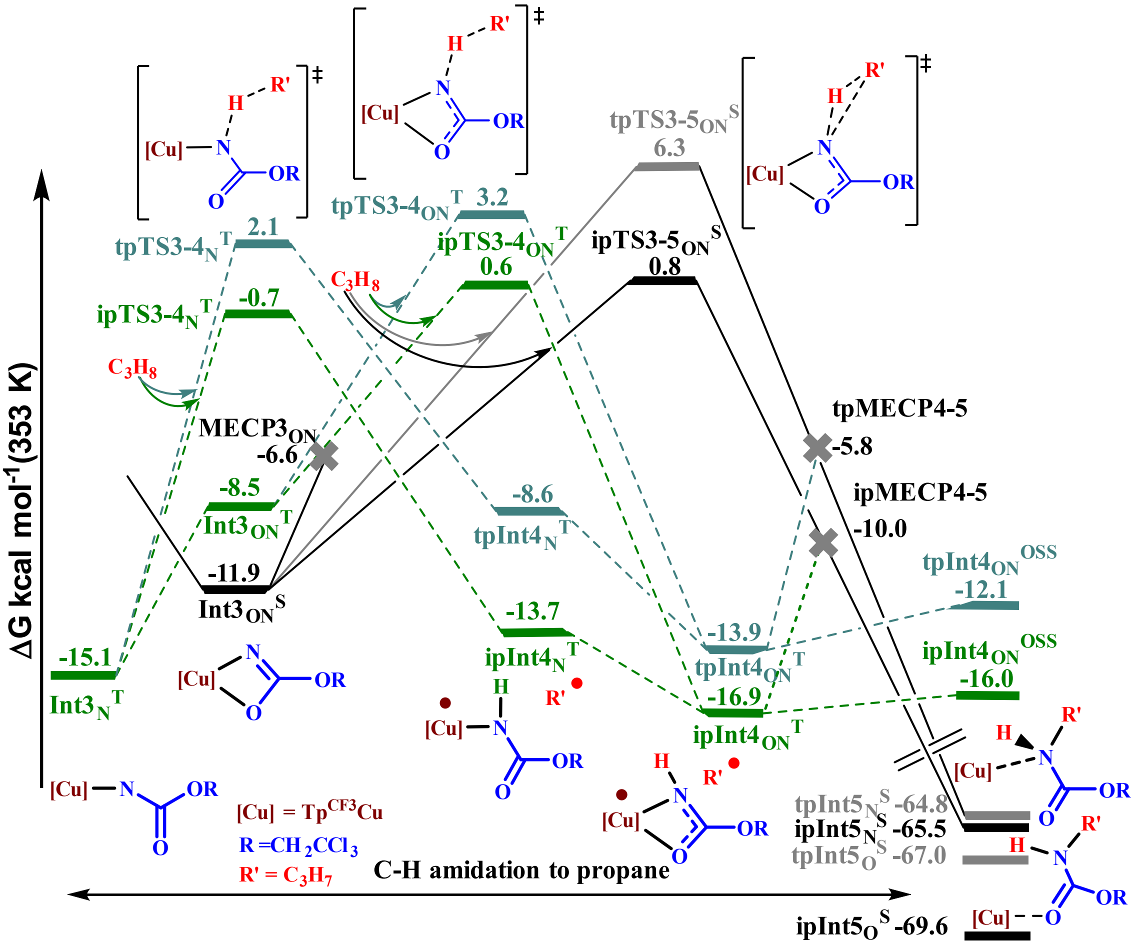

Figure S6. *Free energy profile for propane amidation (353.15 K), in kcal·mol^{-1}. In black and grey solid lines the singlet surface is presented, the triplet surface is represented in dark and sea green dashed lines instead. "tp" stands for terminal CH of propane and "ip" for internal CH of propane.*

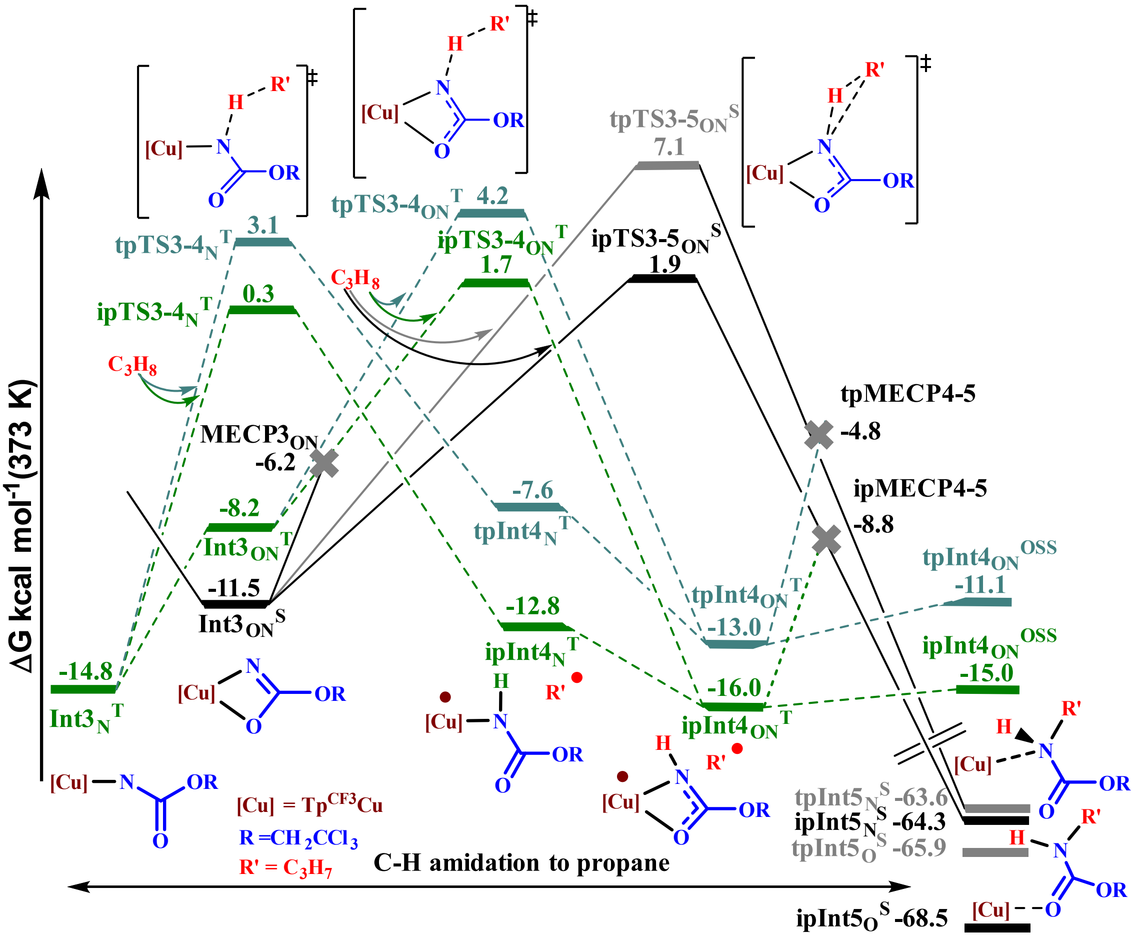

Figure S7. *Free energy profile for propane amidation (373.15 K), in kcal·mol^{-1}. In black and grey solid lines the singlet surface is presented, the triplet surface is represented in dark and sea green dashed lines instead. "tp" stands for terminal CH of propane and "ip" for internal CH of propane.*

14. d. iso-Butane amidation

The computationally postulated mechanism for the amidation of iso-butane from the triplet nitrene intermediate **$Int3_N^T$** is presented in **Figure S8** (353.15 K) and **Figure S9** (373.15 K). Formation of the copper nitrene **$Int3_N^T$** is the same as for methane and is presented in **Scheme 4** of the main text (left) and in **Figure S3** (left). *iso*-butane presents to types of CH bonds: terminal (primary) and internal (tertiary). Minima and transition states on the reaction path leading to the amidation of terminal (primary) CH bonds have the letters "tb" in front of the species name, standing for "terminal butane". Minima and transition states on the path leading to the amidation of the internal (secondary) CH bonds have letters "ib" in front of the species name, standing for "internal butane".

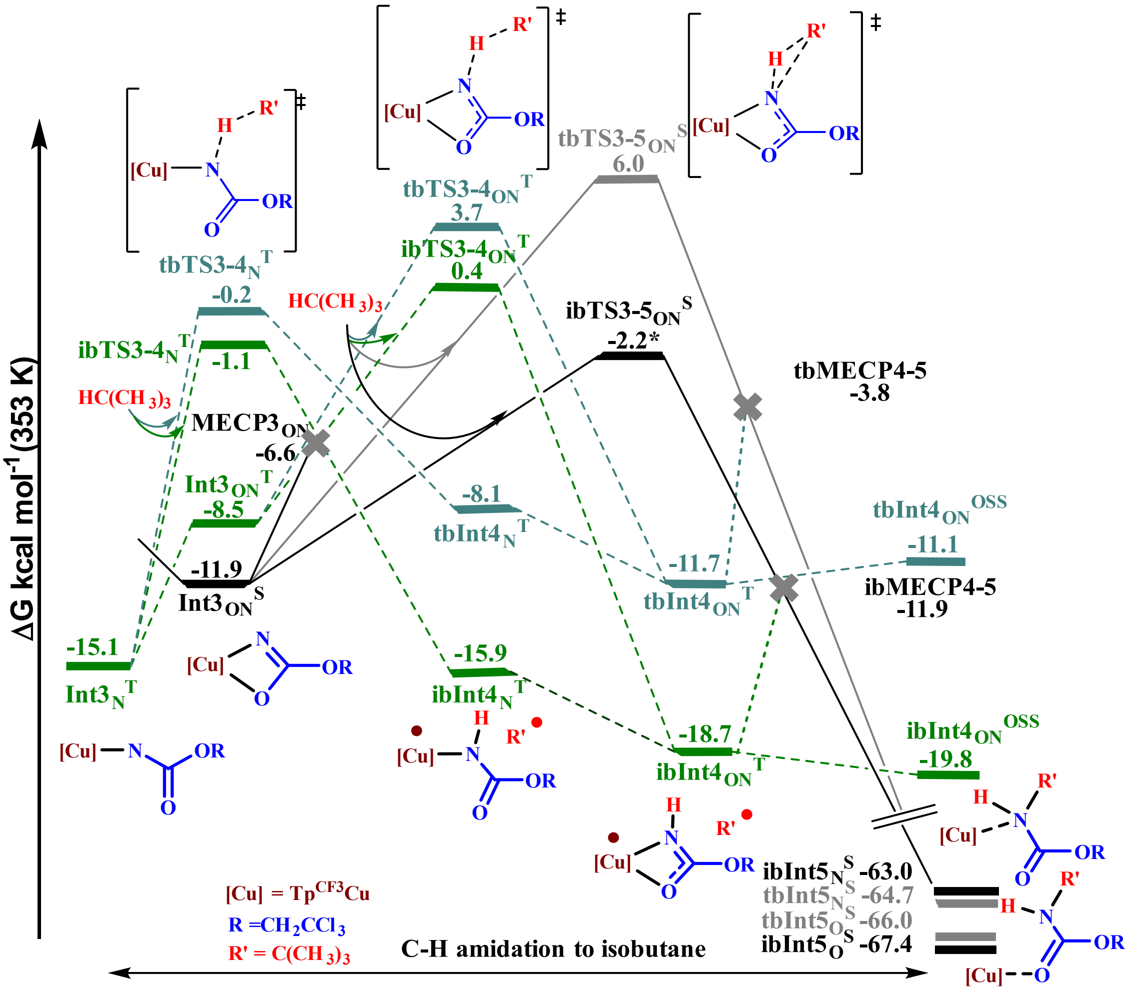

Figure S8. *Free energy profile for iso-butane amidation (353.15 K), in kcal·mol^{-1}. In black and grey solid lines the singlet surface is presented, the triplet surface is represented in dark and sea green dashed lines instead. "tb" stands for terminal CH of iso-butane and "ib" for internal CH of iso-butane. Grey crosses represent Minimum Energy Crossing Points (MECPs). *Not fully characterized, see text.*

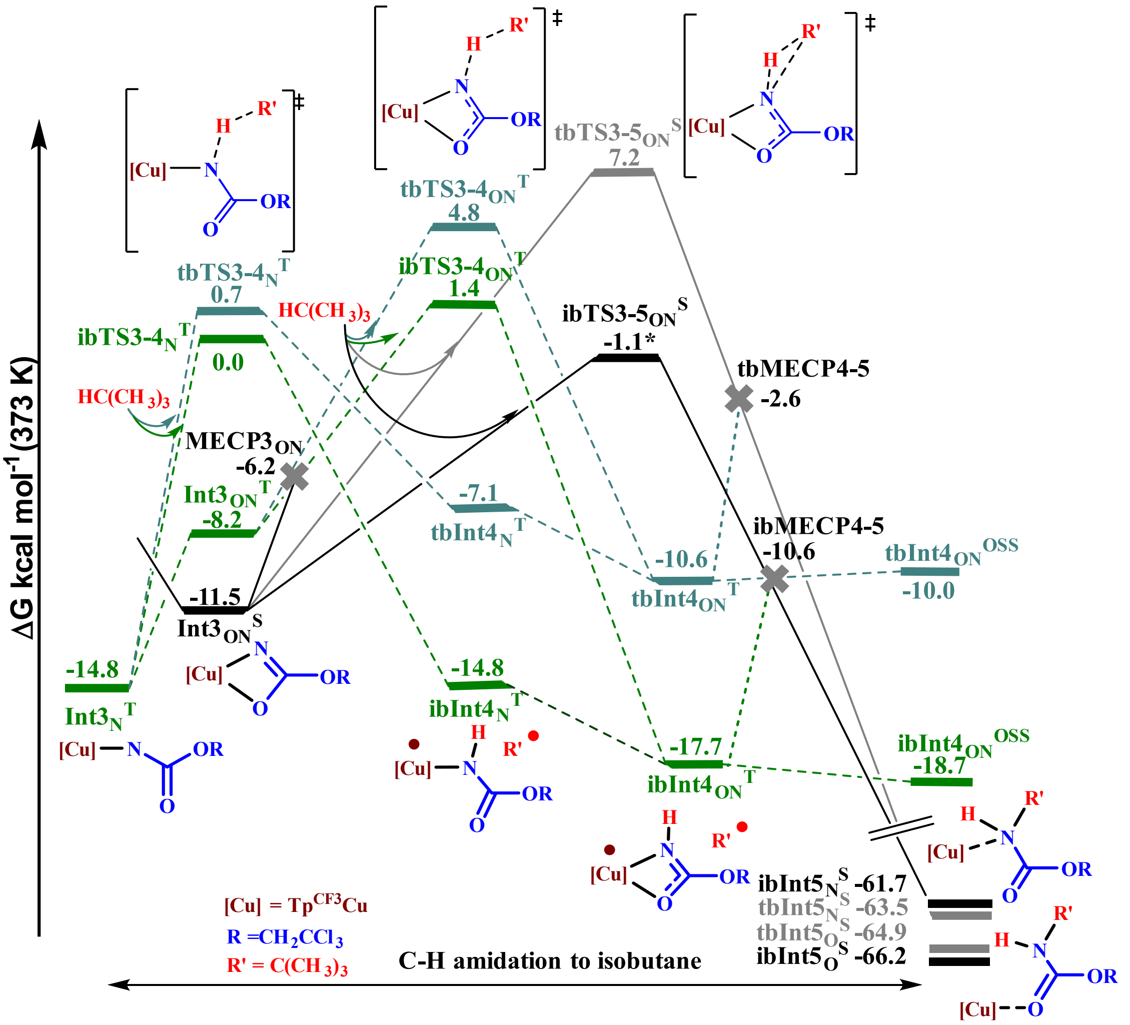

Figure S9. *Free energy profile for iso-butane amidation (373.15 K), in kcal·mol^{-1}. In black and grey solid lines the singlet surface is presented, the triplet surface is represented in dark and sea green dashed lines instead. "tb" stands for terminal CH of iso-butane and "ib" for internal CH of iso-butane. Grey crosses represent Minimum Energy Crossing Points (MECPs). *Not fully characterized, see text.*

It is relevant to note that for the amidation of the internal C-H bond of the *iso*-butane in the singlet potential energy surface, no transition state was found. Scans and transition state searches on the singlet potential energy surface led to no transition state. However, we have been able to locate several very shallow minima. Doing small displacements of the N-H distance, the scan of this distance leads to the amidation product. **Figure S10** shows the PES energy change when scanning the reaction coordinate corresponding to the N-H distance, from the lowest conformer of this shallow minima.

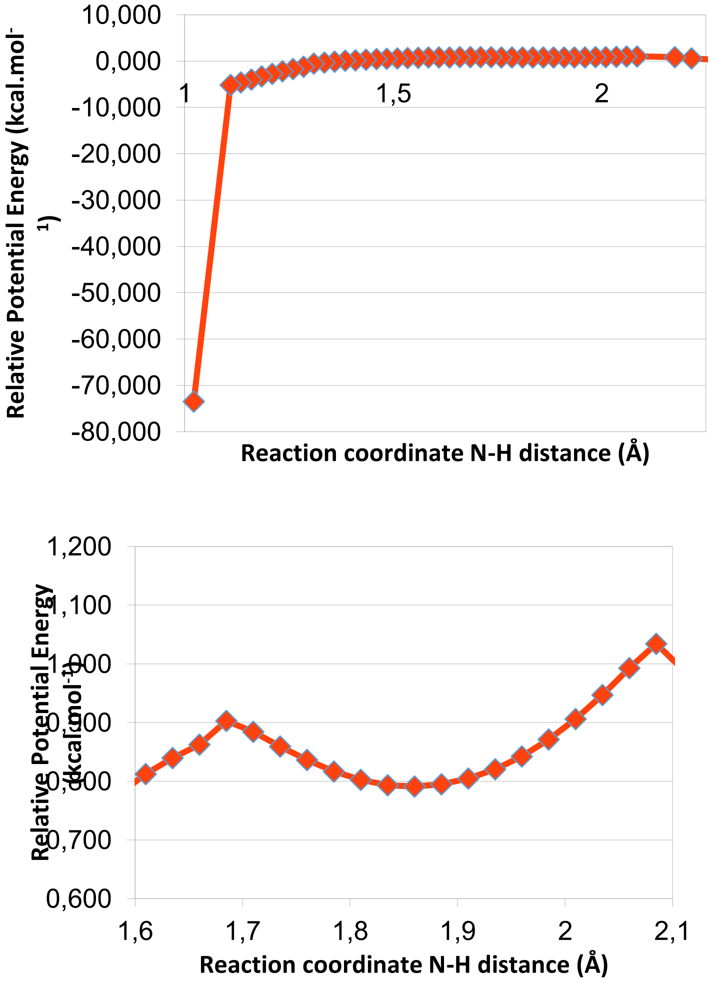

Figure S10. *Scan of the N-H distance (N of metallonitrene and H of substrate's CH bond, in Å) on the singlet Potential Energy Surface for the amidation of the internal (tertiary) C-H bond, relative potential energies in kcal·mol^{-1}. Top: Large view of the scan. Bottom: Zoom on the located shallow minima.*

Scans show a very flat region from 1.4 until 2.1 Å. The lowest energy minimum has an N-H distance of 1.860 Å and a relative energy of -1.6 kcal·mol^{-1}. This species is confirmed to be a minimum on the PES with no imaginary frequency. There are two very low energy maxima at each side of this minima in the PES. Free energies have been computed for these two maxima points. The point of higher energy at the N-H distance of 1.685 Å has a relative free energy of -4.2 kcal·mol^{-1}, and the one at 2.085 Å has a relative free energy of -5.4 kcal·mol^{-1}. Hence, both maxima on the PES have lower free energy than the minima, and hence the shallow minima (with an energy of -1.6 kcal·mol^{-1}) is higher in free energy and has been considered as an approximate measure of the higher energy point in the reaction path and has been labelled as **ibTS3-5$_{ON}$^{S}**.

15. Microkinetic model

15. a. Construction of the microkinetic model

Microkinetic models convert the computed energies reported in the free energy profiles to theoretical evolution of species (reactants, intermediates and products) over time, considering some specific reaction conditions (i.e. initial concentrations, temperature). These models take into account concentration effects, as a difference from free energy profiles that are computed at a specific standard state (1 atm or 1M, in this study 1M), data that can be directly compared to experimental data, and results usually allow a deeper understanding of the catalytic system.[31,32]

In order to build the model, it is necessary to define the elementary steps of the reaction and their rate laws (equations). For a microkinetic model of a catalytic reaction, all relevant in-cycle and all off-cycle reaction steps must be defined. The rate constant for each step can be obtained using computational free energies within Transition State Theory and Eyring–Polanyi equation, see eq 1,

$$k = \frac{k_B.T}{h} exp\left(-\frac{\Delta G\ddagger}{RT}\right) \qquad \text{eq(1)}$$

where k_B is the Boltzmann constant, h is Planck's constant, T is the absolute temperature, R is the gas constant, and $\Delta G^{\ddagger}$ is the activation free energy. For the few barrierless steps (low-energy steps for which a transition state could not be located), the barriers have been approximated as diffusion barriers and evaluated using the Stokes–Einstein equation and the Smoluchowski relation,[33] see eq(2),

$$k_D = \frac{8k_B.T}{3\eta} \qquad \text{eq(2)}$$

where η is the fluid viscosity. For the models presented herein, these small barriers have been assigned a diffusion constant of 3.974×10^{10} s^{-1} (equivalent to 3.91 kcal·mol^{-1}), see reference 32 for further details.

The full kinetic models were constructed using the reaction steps detailed in Table S6. The reaction rate constants were computed using equations 1 and 2. For the reactivity of alkanes using energies reported in free energy profiles in **Figures S3** and **S9** and at 373.15 K. While for the conditions of nitrene detection, energies reported in free energy profile in **Figure S20** and at 303.15 K. Rate laws were derived and numerically integrated using COPASI.[34,35]

Table S6. Reaction steps included in the microkinetic model.

	Reaction steps
**Reaction 1**	Int0 ⇌ Int1S + NCMe
**Reaction 2**	Int1S + N3R ⇌ Int2NS
**Reaction 3**	Int1S + N3R ⇌ Int2OS
**Reaction 4**	Int2NS ⇌ Int3NS + N2
**Reaction 5**	Int3NS ⇌ Int3NT
**Reaction 6**	Int3NS ⇌ Int3ONS
**Reaction 7**	Int3NT ⇌ Int3ONT
**Reaction 8**	Int3ONS ⇌ Int3ONT
**Reaction 9**	Int3NT + R ⇌ Int4NT
**Reaction 10**	Int3ONT + R ⇌ Int4ONT
**Reaction 11**	Int4NT ⇌ Int4ONT
**Reaction 12**	Int3ONS + R ⇌ Int5NS
**Reaction 13**	Int4ONT + R ⇌ Int4ONOSS
**Reaction 14**	Int4ONOSS + R ⇌ Int5NS
**Reaction 15**	Int4ONT ⇌ Int5NS
**Reaction 16**	Int5NS ⇌ Int5OS
**Reaction 17**	Int5OS ⇌ Int1S + Product
**MECHANISM A**	
**Reaction 18**	Int3ONT + H_2O ⇌ Int1S + subproduct
**MECHANISM B**	
**Reaction 18**	Int3ONT ⇌ Int1S + subproduct
**MECHANISM C**	
**Reaction 18**	Int3ONT + H_2O ⇌ Int6 + subproduct1
**Reaction 19**	Int6 + R ⇌ Int1S + subproduct2
**MECHANISM D**	
**Reaction 18**	Int3ONT + H_2O ⇌ Int6 + subproduct1
**Reaction 19**	Int6 ⇌ Int1S + subproduct2

In this study, we constructed models of three different chemical systems with different initial concentrations and temperatures to match the experiments.

1. Reaction of ethane as the substrate.

2. Reaction of methane as the substrate.

3. Conditions for nitrene intermediate detection (HMRS-ESI).

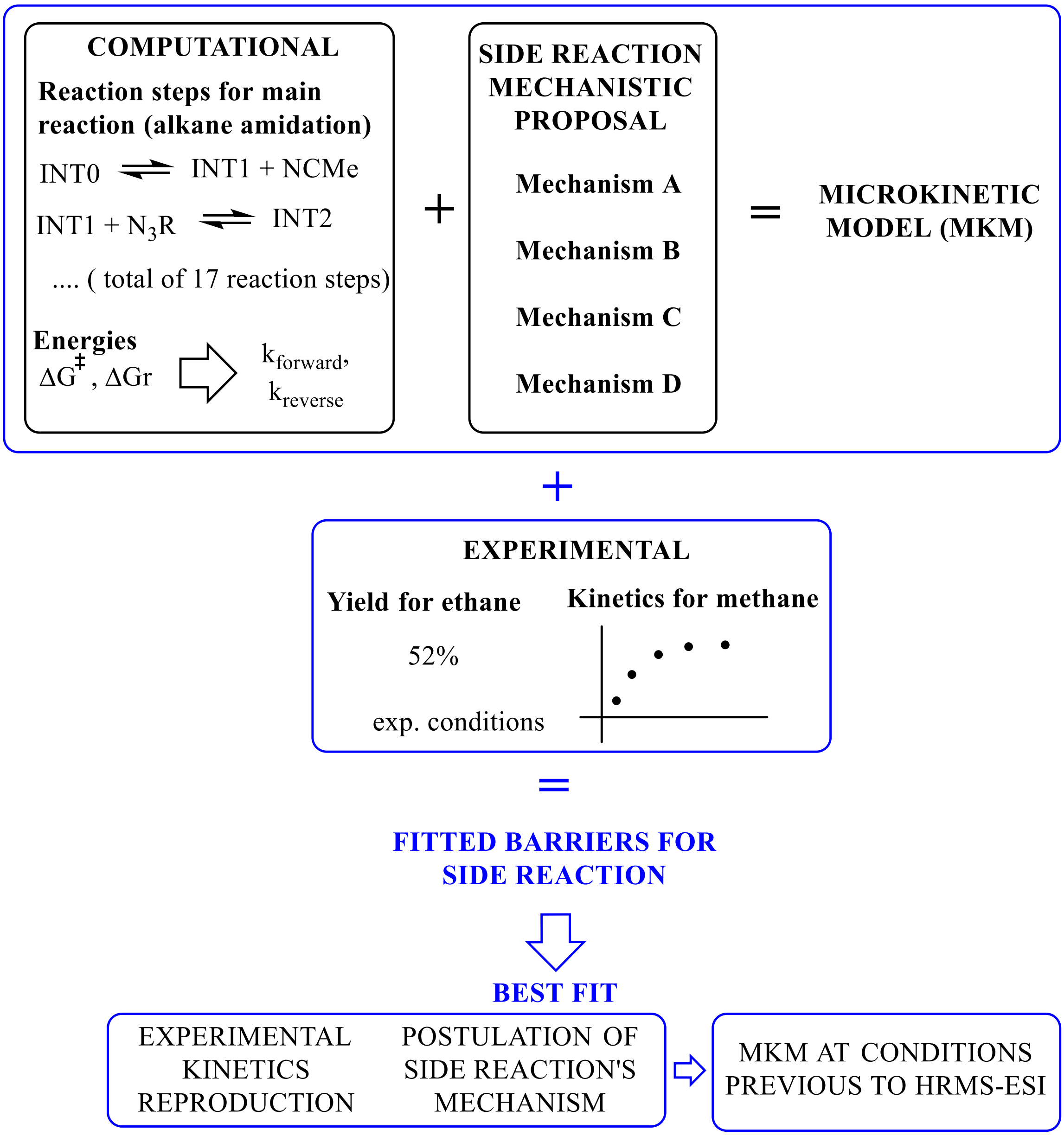

Scheme S1. *Summary of the steps followed for this microkinetic study.*

Ethane (product yield) and methane (kinetic evolution of the product over time; see **Table S4**) have been used to gain insight into the main amidation reaction, as well as into the side reaction involving nitrene decomposition, which leads to the formation of 2,2,2-trichloroethyl carbamate and other subproducts (see above). In order to explain the experimental yields and the kinetic evolution of the products over time, it is necessary to incorporate the side reactions into the microkinetic model. However, this side reaction has not been computationally studied for this catalytic system, as it is expected to be complex and is beyond the scope of the current study. Therefore, we used the available experimental information and computational free energy profiles to test four plausible, simplified mechanisms (A, B, C and D; see below). Using this information, we reproduced the experimental kinetic data and gained insight into the side reaction's mechanism. The scheme followed for this microkinetic study is summarized in Scheme S1.

15. b. Considered mechanisms for side reaction

The side reaction leading to the partial nitrene decomposition or hydrolysis to form mainly 2,2,2-trichloroethyl carbamate, along with other subproducts, has been experimentally observed (see above). The formation of these by-products was previously reported by Chang et al.[11] Also, De Bruin, Reek, Roithová, and co-workers experimentally and computationally studied the nitrene hydrolysis mechanism during aqueous styrene aziridination, which is mediated by the $[Co^{III}(TAML^{red})]^{-}$ catalyst.[36] In neutral/basic media, they postulated the formation of an oxo/oxyl radical cobalt complex as an intermediate, formed by the hydrolysis of the corresponding cobalt nitrene after undergoing proton transfers and hydroxy group coordination steps, see also reference [37].

However, the mechanism encountered for the aforementioned cobalt system in aqueous media is not expected to be followed exactly for the copper catalysts reported herein under *sc.* CO_2 and with only adventitious water. This is mainly because nitrene protonation is expected to be difficult under the experimental conditions. Instead, other mechanisms could occur, such as hydrolysis via insertion into the O–H bond of spurious water or reorganization of the NTroc, to name a few. Although the study of this side reaction is very interesting, it is not within the scope of the current work and will be considered in future studies. Nevertheless, in order to reproduce the experimental yield and reaction kinetics from the computational data, it is necessary to have a simplified, general

mechanism that depends on concentrations, as well as an approximate value for the side reaction barrier. To this end, we devised a method to gain insight and obtain useful data: i) use the results of the computational study (free energy profile) to compute reaction rate constants and build a microkinetic model for the amidation reaction (black cycle in Scheme S2); ii) test different reaction mechanisms for the side reaction and fit the barrier or barriers for each of them; iii) check which mechanism best fits the experimental results and is more effective in reproducing the kinetic curves obtained for methane.

Four simple mechanistic proposals were considered for the side reaction: i) hydrolysis as the rate-determining step of the side reaction or not and ii) selectivity and rate-determining step the same or different steps. The four possibilities tested are mechanisms A (dark green), B (light green), C (orange) and D (red), see Scheme S2.

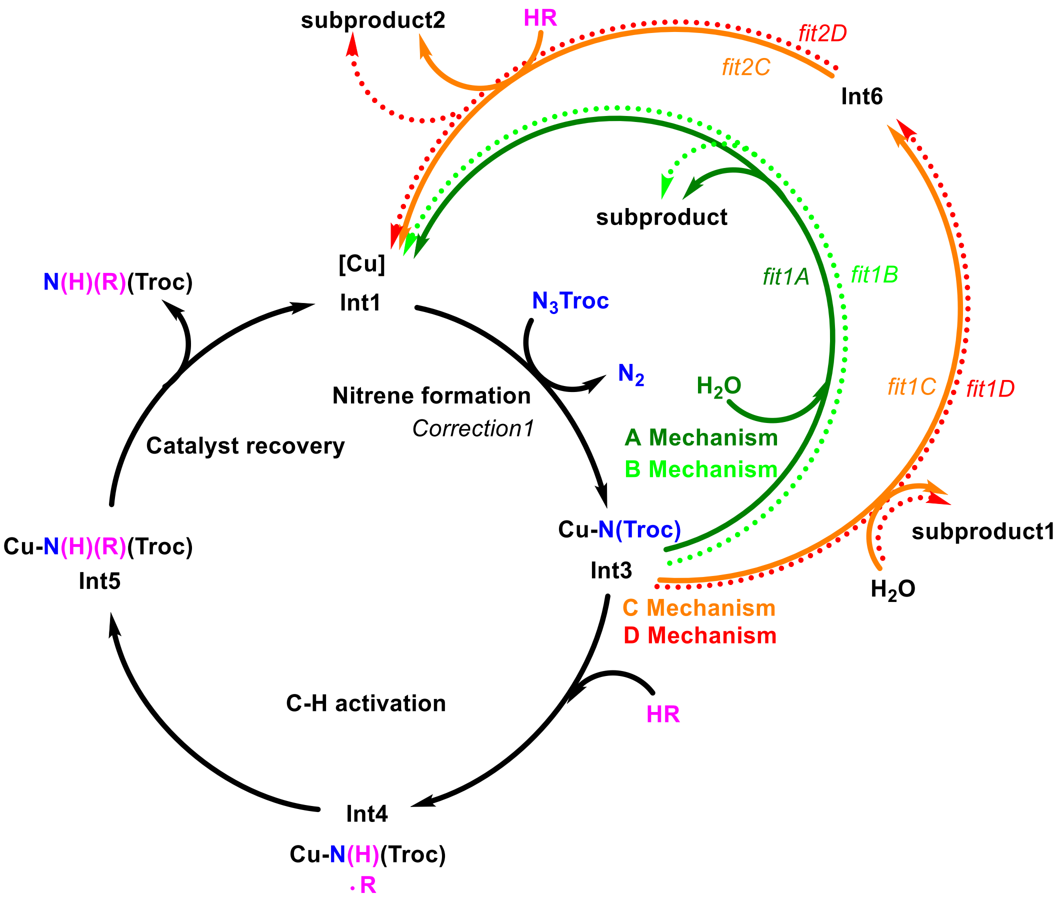

Scheme S2. *Simplified scheme of the catalytic reaction cycle for the alkane amidation via copper catalyst, including side reaction mechanisms A (solid dark green), B (dashed light green), C (solid orange) and D (dashed red).*

Mechanism A:

The side reaction could follow the simple kinetics of:

$\mathbf{Int3_N^T}$ + H_2O → subproduct + $\mathbf{Int1^S}$

This represents a mechanism that starts with $\mathbf{Int3_N^T}$, involves a single step with participation of water molecules, solid dark green in **Scheme S2** and **S3**. This is a plausible possibility, given that hydrolysis is both the slowest and the selectivity-determining step in the aforementioned mechanism by de Bruin and co-workers[36]. Indeed, the real mechanism could be much more complex than this single step, but this simplified mechanism would represent those with a selectivity step dependent on the concentration of water. As although no water is added as reagent or additive, adventitious water is present. It is also well known that the reaction yield benefits from the use of molecular thieves, which reduce side reaction yields. The fitted free energy barrier according to this mechanism will be named **fit1A**.

Mechanism B:

The side reaction could follow the simple kinetics of:

$\mathbf{Int3_N^T}$ → subproduct + $\mathbf{Int1^S}$

This is a simple step similar to mechanism **A**, but it does not depend on the concentration of water (dashed light green in **Scheme S2** and **S3**). This represents different mechanisms starting from $\mathbf{Int3_N^T}$, that don't involve the participation of water or any other species in low concentrations (for high concentrations, k[species] ≈ k'(apparent), see below). For instance, a plausible example would be a mechanism governed by an internal arrangement. Note that this does not prevent water or any other species from playing a role in the mechanism, but they should participate in a step that is not relevant for selectivity or velocity. The fitted free energy barrier according to this mechanism will be named **fit1B**.

Please note that in mechanism **A**, if the water concentration is much greater than that of the azide, the result would be similar to that of mechanism **B**, as $k[H_2O][\mathbf{Int3_N^T}] \approx k'(\text{apparent})[\mathbf{Int3_N^T}]$. However, as $k \neq k'(\text{apparent})$, the fitted ΔG values (**fit1A** and **fit1B**) will differ for mechanisms **A** and **B**, but be related by $[H_2O]$.

Mechanism C:

The side reaction could follow the simple kinetics of:

Int3$_N$^{T} + H_2O → subproduct1 + **Int6**

Int6 + alkane → subproduct2 + **Int1^S**

The real mechanism could be much more complex than the two steps shown in orange in **Scheme S2** and **S3**. This mechanism is similar to that found for the aforementioned cobalt complex by de Bruin et al.,[36] where **Int6** could be a proposed oxo/oxyl radical complex or another species. This intermediate would then react with the alkane to form **subproduct2**. Unlike de Bruin and co-workers,[36] this final step would have an important associated barrier. Please note that if the second step (**Int6** to **Int1^S**) has a low barrier, the mechanism behaves like Mechanism **A**. However, if the second step is similar to or higher than the main reaction-determining step (**Int0** to **Int3$_N$^{T}**), both steps of Mechanism **C** play a role: the first (**Int3$_N$^{T}** to **Int6**) defines the selectivity, and the second (**Int6** to **Int1^S**) partially controls the reaction speed (it does not control the main reaction in black in **Scheme S2**). According to this mechanism, the fitted free energy barriers will be named **fit1C** for the first, selectivity-determining step and **fit2C** for the second, rate-determining step.

Mechanism D:

The side reaction could follow the simple kinetics of:

Int3$_N$^{T} + H_2O → subproduct1 + **Int6**

Int6 → subproduct2 + **Int1^S**

This mechanism, shown in red and dashed in **Scheme S2** and **S3**, is similar to Mechanism C. However, in this case, the recovery of the catalytic species **Int1** is independent of the alkane concentration or any other species. Please note that, once again, if the alkane concentration is higher than the azide concentration, this mechanism would produce a similar result to Mechanism **C**, as k[alkane][**Int6**] ≈ $k'_{apparent}$[**Int6**]. According to this mechanism, the fitted free energy barriers will be named **fit1D** and **fit2D** for the first and second steps, respectively.

Mechanism A:

Int3$_N^T$ + H_2O → subproduct + **Int1^S**

Mechanism B:

Int3$_N^T$ → subproduct + **Int1^S**

Mechanism C:

Int3$_N^T$ + H_2O → subproduct + **Int6**

Int6 + alkane → subproduct2 + **Int1^S**

Mechanism D:

Int3$_N^T$ + H_2O → subproduct1 + **Int6**

Int6 → subproduct2 + **Int1^S**

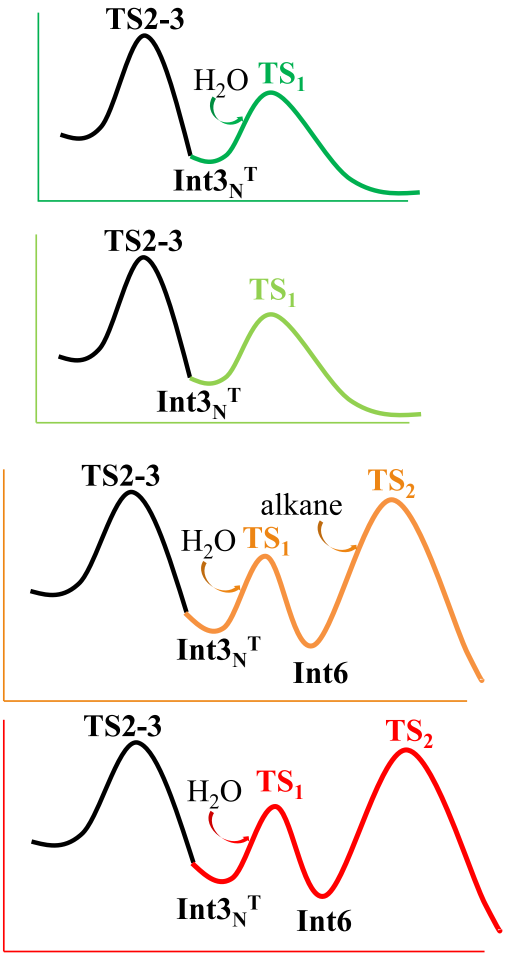

Scheme S3. *Reactions and simplified potential energy surface of the catalytic reaction cycle for the alkane amidation for side reaction mechanisms A (solid dark green), B (dashed light green), C (solid orange) and D (dashed red).*

15. c. Microkinetic model corrections and fitting

Microkinetic models are highly sensitive to small changes in the energetic barriers of rate- or selectivity-determining steps. For this reason, corrections are commonly applied to microkinetic models built from computational energies in order to adjust them to experimental results and evaluate the discrepancy between experiments and theory. In this study, we considered a single possible correction (**correction1**) to the barrier for nitrogen extrusion (from **Int2$_N^S$** to **Int3$_N^S$**).

As mentioned above, however, the side reaction or hydrolysis mechanism has not been investigated computationally. Instead, we fitted the barriers for mechanisms **A**, **B**, **C** and **D**, searching for the energy barrier value that would allow us to reproduce the

experimental results. This led to the following fitted energies: **fit1A**, **fit1B**, **fit1C**, **fit2C**, **fit1D** and **fit2D**. The fittings have been computed from **Int3$_{ON}$^{T}**. See details below.

15. d. Fitting of the microkinetic model for ethane with experimental results

The thermodynamic and kinetic data obtained from the computational free energies at 373.15 K (see **Figures S5** and **S3** for copper nitrene formation) were used to calculate the reaction rate constants required for the microkinetic modelling and testing of mechanisms **A**, **B**, **C** and **D**. As ethane has six equivalent C-H bonds, the rate constants for the steps involving ethane were multiplied by six. The results obtained are presented in **Table S7**. Please note that our fitting and final selection were based on: i) reproducing the experimental yield and ii) achieving completed reaction after 72 hours. The experimental conditions considered are: 373.15 K, 72 hours, [N_3R]= 0.003 M, [catalyst]= 0.0003 M, [ethane]= 1.065 M and [H_2O]= 0.0169 M.

The results of the microkinetic model for ethane, expressed as a percentage yield of the amidated product, and the agreement with the reaction time, are presented in **Table S7**. For mechanism **A**, the best fit for a yield of 52% is achieved with a side reaction barrier of **fit1A** = 6.2 kcal·mol^{-1} from **Int3$_{ON}$^{T}** (entry 2, **Table S7**), when the computational barriers are used (**correction1** = 0, see above). Lower and higher barriers lead to an under- and overestimation of the reaction yield (entries 1 and 3). However, the reaction proceeds too quickly and is completed within approximately one day. To fit the end of the reaction at around 72 hours, the barrier for nitrene formation must be increased; good results are achieved with **correction1** = 1.0 kcal·mol^{-1} (entry 5). With **correction1** = 0.5 kcal·mol^{-1} (entry 4), the reaction is still slightly too fast, while larger barriers lead to incomplete reactions within the experimental reaction times (entries 6 - 8). Therefore, the best fit for mechanism **A** was found with **correction1** = 1.0 kcal·mol^{-1}and **fit1A** = 6.2 kcal·mol^{-1} (entry 5, **Table S7**), shaded in light green in the table.

Mechanism **B** produces similar results to mechanism **A** because the water concentration is relatively high (0.0169 M) in front of the azide concentration. Therefore, k_{1A}[H_2O] must be similar to k_{1B}. Consequently, the barrier for the side reaction in mechanism **B** (which does not depend on water) must be larger than **fit1A**. Our results indicate that **fit1B** should be approximately 9.3 kcal·mol^{-1}. The best results are also achieved for **correction1** = 1.0 kcal·mol^{-1}and **fit1B** of 9.3 kcal·mol^{-1} (entry 10, **Table S7**).

Mechanism **C** performs well for several of the adjustments highlighted in pale green in **Table S7**. All solutions require the barrier for the selectivity step, **fit1C**, to be 6.2 kcal·mol^{-1}, smaller barrier for **fit1C** (entry 13, **Table S7**) leads to low proportion of products and larger to too high. For the other two parameters:

- A good adjustment involves not correcting the nitrene formation step (**correction1**= 0), fitting **fit2C** = 31 kcal·mol^{-1} (entry 15, **Table S7**), smaller or larger **fit2C** to too fast or too slow reactions (entries 14 and 16, **Table S7**).
- Another adjustment consists on **correction1** = 1 kcal·mol^{-1} and a small barrier for **fit2C** (entries 21 and 22, **Table S7**). This is the same solution as mechanism A, as the barrier for nitrene formation is high and if **fit2C** is relatively small, its influence on the mechanism is minimal. If the **fit2C** increases to 30 or 31 kcal·mol^{-1} (entries 23 and 24, **Table S7**) the yield is lower than expected as the reaction does not achieve full completion.
- Also, another adjustment is found between these two, with **correction1** = 0.5 kcal·mol^{-1} and **fit1C** and **fit2C** of 6.2 and 30.0 kcal·mol^{-1} (entry 19, **Table S7**). This is an intermediate point where the speed of the reaction is defined by the nitrene formation step and the second side reaction step (entries 14 and 23, and 18 and 20, **Table S7**).

Mechanism **D** produces similar results as mechanism **C**, best results are achieved for **correction1**, **fit1D** and **fit2D** of 0.5, 6.2 and 30 kcal·mol^{-1} (entry 27, **Table S7**). **fit2D** is similar to **fit2C**, as the concentration of ethane is 1.06 M. The reaction with **correction1** = 1.0 kcal·mol^{-1} gives similar results although is slightly slow and does not lead to full completion (entry 28, **Table S7**) while at lower values (**correction1**= 0.0 kcal·mol^{-1}) the reaction proceeds too fast (entry 25, **Table S7**).

The best fit/s found for each mechanism are highlighted in pale green in **Table S7**, and correspond to entries 5, 10, 15, 19, 21 and 27. They will be used as starting point to build a new microkinetic model for methane.

Table S7. *Computed and experimental yields % for **ethane** as substrate at **373.15 K** for Mechanism A, B, C and D, applying **correction1** (to energy barrier for nitrene formation) and fitted **fit1A**, **fit1B**, **fit1C/2C** or **fit1D/2D** for the side reaction barrier/s from* ***Int3$_{ON}$^{T}***.

**Entry**	**Correction1 kcal mol^{-1}**	**Mechanism**	**Fit 1A, 1B 1C/2C or 1D/2D kcal mol^{-1}**	**Prod [%] t= 72 h.**	**Exp.[%] t= 72 h.**	**Agreement[a]**
**1**	0	A	6	45	52	RC, F
**2**	0	A	6.2	52	52	RC, F
**3**	0	A	7	74	52	RC, F
**4**	0.5	A	6.2	52	52	RC, F
**5**	1	A	6.2	51	52	RC, G
**6**	1.5	A	6.2	48	52	RNC, G
**7**	2	A	6.5	47	52	RNC, G
**8**	2	A	7	55	52	RNC, G
**9**	0	B	9.2	50	52	RC, F
**10**	1	B	9.3	53	52	RC, G
**11**	1.5	B	9.3	49	52	RNC, G
**12**	1.5	B	10	66	52	RNC, G
**13**	0	C	6/31	46	52	RC, F
**14**	0	C	6.2/30	52	52	RC, F
**15**	0	C	6.2/31	52	52	RC, G
**16**	0	C	6.2/32	28	52	RNC, G
**17**	0	C	6.5/32	50	52	RNC, G
**18**	0.5	C	6.2/20	52	52	RC, F
**19**	0.5	C	6.2/30	52	52	RC, G
**20**	0.5	C	6.2/31	51	52	RC, G
**21**	1	C	6.2/20	51	52	RC, G
**22**	1	C	6.2/29	51	52	RC, G
**23**	1	C	6.2/30	50	52	RC, G
**24**	1	C	6.2/31	47	52	RNC, S
**25**	0	D	6.2/30	52	52	**RC, F**
**26**	0	D	6.2/31	27	52	RNC, S
**27**	0.5	D	6.2/30	52	52	**RC, G**
**28**	1	D	6.2/30	50	52	**RC, G**

[a] *Fittings have been computed from* ***Int3$_{ON}$^{T}***. [b] *RC = reaction completed, RNC = reaction not completed, F = too fast, S = too slow and G = good speed.*

15. e. Fitting of the microkinetic model for methane with experimental results

The thermodynamic and kinetic data required to obtain the reaction rate constants for building microkinetic models for methane amidation were obtained from the computational free energies shown in **Figure S3** at 373.15 K. Mechanisms **A**, **B**, **C** and **D** were tested. As methane has four equivalent C-H bonds, the reaction rate constants for steps involving methane were multiplied by four. The selected results are presented in **Tables S8** and **S9**. Please note that, in this case, the results of the computational microkinetic model are not compared with a single reference value (yield), but with several data points of the concentration of the amidation product over time. This allows for a better comparison between experimental and computational kinetic results. The search for solutions was based on: i) reproducing the curve obtained in the experimental kinetic study (**Table S4**) and ii) full azide consumption after 72 hours. The experimental conditions used are: 373.15 K, 72 hours, [N_3R]= 0.003 M, [catalyst]= 0.0003 M, [methane]= 4.99 M and [H_2O]= 0.0169 M.

For mechanism **A**, the ethane best for **fit1A** lead to a reaction that is too fast and has a too low product yield (see entry 1, **Table S8**). To achieve a product yield close to the experimentally observed 22%, a small increase in **fit1A** to 7.4 kcal·mol^{-1} is necessary. This small difference of 1.2 kcal·mol^{-1} could be due to small errors in the methane/ethane activation barriers or to the change in total pressure between the two experiments, among other factors. In order to reproduce the speed, **correction1** also needs to be increased by around 1.6 kcal·mol^{-1}, to approximately 2.8 kcal·mol^{-1} (entry 5, **Table S8**). However, when the long-time yield is reproduced (entries 2 and 3, **Table S8**), the short-time yield is not, and vice versa (entries 1–8, **Table S8**), so the fitting cannot achieve an accurate solution for mechanism **A**. To evaluate the agreement, we computed the mean absolute error (MAE) between the microkinetic model results and the experimental results in **Table S4**, see **Table S9**. The best solution for mechanism **A** (entry 5, **Table S8** and **S9**) has an associated MAE of 1.8 kcal·mol^{-1}. Please note that this is the lowest MAE found for finished reactions. If the reaction is not completed, it is possible to obtain a product formation curve with smaller errors (see **Table S9**, entries 7–9). However, this does not reproduce the experiments.

Table S8. *Computed and experimental yields % for* ***methane*** *as substrate at* ***373.15 K****, applying correction1 (correction to energy barrier for nitrene formation) and fitting side reaction barrier/s* ***(fit1A****,* ***fit1B****,* ***fit1C/2C*** *or* ***fit1D/2D****) for Mechanism* ***A****,* ***B****,* ***C*** *and* ***D****.*

Entry	Correction1 kcal mol^{-1}	Mechanism	Fit1A, 1B, 1C/2C and 1D/2D[a] kcal mol^{-1}	Prod [%] t= 24 hours	Prod [%] t= 72 hours	Agreement[b]
**Experim.**				14.5	22.0	
1	1	A	6.2	5.3	5.4	RC, F
2	2.2	A	7.4	21	22	RC, F
3	2.5	A	7.4	19	22	RC, F
4	2.6	A	7.4	18	22	RC, F
5	2.8	A	7.4	17	22	RC, G
6	3.0	A	7.4	15	20.8	RNC, G
7	3.2	A	7.4	13	19.7	RNC, S
8	3.5	A	7.4	11	19.0	RNC, S
9	1	B	9.3	5	6	RC, S
10	2.2	B	10.6	23	25	RC, F
11	2.8	B	9.3	5	5	RC, S
12	2.8	B	10.4	15	20	RC, G
13	2.8	B	10.5	17	22	RC, G
14	3.0	B	10.5	15	21	RNC, S
15	3.2	B	10.6	14	22	RNC, S
16	0	C	6.2/31	4	5	RC, S
17	0	C	7.4/31	19	22	RC, F
18	0	C	7.4/31.5	13	22	RC, G
19	0	C	7.4/32	9	20	RC, S
20	2.2	C	7.4/28	20	22	RC, F
21	2.2	C	7.4/30	14	22	RC, G
22	2.3	C	7.4/30	13	22	RC, G
23	2.4	C	7.4/30	13	22	RC, G
24	2.8	C	7.4/28	16	22	RC, G
25	2.8	C	7.4/30	10	20	RNC, S
26	3.0	C	7.4/30	15	21	RC, G
27	0	D	7.4/30	17	22	RC, F
28	0	D	7.4/30.5	11	23	RC, G
29	1	D	6.2/30	3	6	RC, S
30	2.2	D	7.4/29	13	22	RC, G
31	2.2	D	7.4/30	7	17	RNC, S
32	2.8	D	7.4/30	8	12	RNC, S

[a] *Fittings have been computed from* ***Int3$_{ON}$^{T}***. [b] *RC = reaction completed, RNC = reaction not completed, F = too fast, S = too slow and G = good speed.*

Table S9. *Selected results of the fitting with experimental results for methane as the substrate.*

Entry	Correction1 kcal mol^{-1}	Mecha nism	Fit 1A, 1B, 1C/2C and 1D/2D[a] kcal mol^{-1}	MAE[%] kcal mol^{-1} [b]
1	1	A	6.2	8.7
2	2.2	A	7.4	5.1
3	2.6	A	7.4	3.0
4	2.8	A	7.2	2.2
5	2.8	A	7.4	1.8
6	2.8	A	7.6	4.7
7	3.0	A	7.4	1.0 RNC
8	3.2	A	7.4	1.2 RNC
9	3.2	A	7.5	0.8 RNC
10	1	B	9.3	8.5
11	2.2	B	10.6	7.6
12	2.8	B	10.4	1.9
13	2.8	B	10.5	2.3
14	3.1	B	10.6	1.6 RNC
15	3.2	B	10.5	0.9 RNC
16	3.2	B	10.6	0.9 RNC
17	3.2	B	10.7	2.1 RNC
18	3.3	B	10.6	0.8 RNC
19	0	C	6.2/31	9.3
20	0	C	7.4/31	2.4
21	0	C	7.4/31.5	1.1
22	0	C	7.4/32	6.4
23	2.0	C	7.4/30	0.8
24	2.1	C	7.4/30	0.6
25	2.2	C	7.3/30	1.3
26	2.2	C	7.4/29	2.7
27	2.2	C	7.4/29.9	1.8

**28**	**2.2**	**C**	**7.4/30**	**0.5**
**29**	**2.2**	**C**	**7.4/30.1**	**1.3**
**30**	**2.2**	**C**	**7.4/31**	**3.3 RNC**
**31**	**2.2**	**C**	**7.5/30**	**1.6**
**32**	**2.3**	**C**	**7.4/30**	**0.5**
**33**	**2.4**	**C**	**7.4/30**	**0.8**
**34**	**3.2**	**C**	**7.4/30**	**7.4RNC**
**35**	**0**	**D**	**7.4/30**	**1.6**
**36**	**0**	**D**	**7.4/30.5**	**1.4**
**37**	**1**	**D**	**6.2/30**	**9.9**
**38**	**2.2**	**D**	**7.4/29**	**0.7**

[a] Fittings have been computed from $\mathbf{Int3_{ON}^{T}}$. [b] RNC = Reaction not completed.

As observed for ethane, Mechanism **B**, needs a **fit1B** higher than **fit1A**, a good **fit1B** for methane is found at 10.4 kcal·mol^{-1} and a **correction1** of 2.8 kcal·mol^{-1} to better reproduce the speed. None of these solutions gives a kinetic curve really close to the experimental one and the errors for completed reactions are large (see **Table S8** and **S9**).

Mechanism **C** gives good agreement between theory and experiment for **fit1C** = 7.4 kcal·mol^{-1}, and, as was seen for ethane, there are several possible adjustments:

- There is a good adjustment when no correction to the nitrene barrier is performed (**correction1** = 0) and a high barrier is given to the second step (**fit2C** = 31.5 kcal·mol^{-1}, entry 18, **Table S8**). The resulting curve reproduces the experimental curve well, with a relatively low MAE of 1.1 kcal·mol^{-1} (entry 21, **Table S9**).
- There is also an adjustment equivalent to mechanism **A** (entry 24, **Table S8**) with **correction1**, **fit1C** and **fit2C** of 2.8, 7.4 and 28.0 kcal·mol^{-1} respectively. As with mechanism **A**, the fitting is poor (MAE = 1.2 kcal·mol^{-1}).
- Using **correction1** = 2.2 or 2.3 kcal·mol^{-1} and **fit2C** = 30 kcal·mol^{-1} (entries 21 and 22, **Table S8**), the resulting curves closely match the experimental curves, with a low MAE of just 0.5 kcal·mol^{-1} (see **Table S9** entries 28 and 32).

Similar results can be found for mechanism **D**, being the best solution **correction1**, **fit1D** and **fit2D** of 2.2, 7.4 and 29.0 kcal·mol^{-1} with good agreement with the experimental

curve (entry 30, **Table S8**) with experimental curve and a small MAE of 0.7 kcal·mol^{-1} (entry 39, **Table S9**).

These results clearly show that mechanisms **A** and **B**, behave very similarly. **C** and **D**, are also very similar. It is evident that the kinetic curve for methane can be better reproduced by mechanisms **C** or **D** than by **A** or **B**. This is illustrated in **Figures S11 - S16**, where the plots of the experimental kinetic curve alongside the microkinetic model results are presented. To visualize the effect of small changes of 0.1 or 0.2 kcal·mol^{-1} in the fitting of the microkinetic models, the optimal values have been plotted alongside results obtained by slightly increasing or decreasing the fitted value.

The best fit for mechanism **A** was found for **correction1** = 2.6 kcal·mol^{-1} and **fit1A** = 7.4 kcal·mol^{-1} (entry 5, **Table S8**). **Figure S11** shows the results of increasing and decreasing **correction1** by 0.2 kcal·mol^{-1}, together with the match with the experimental results, which are plotted in green. Similarly, **Figure S12** shows the results of increasing and decreasing **fit1A** by 0.2 kcal·mol^{-1}. While the results are not completely incompatible, none of them accurately reproduce the experimental curve.

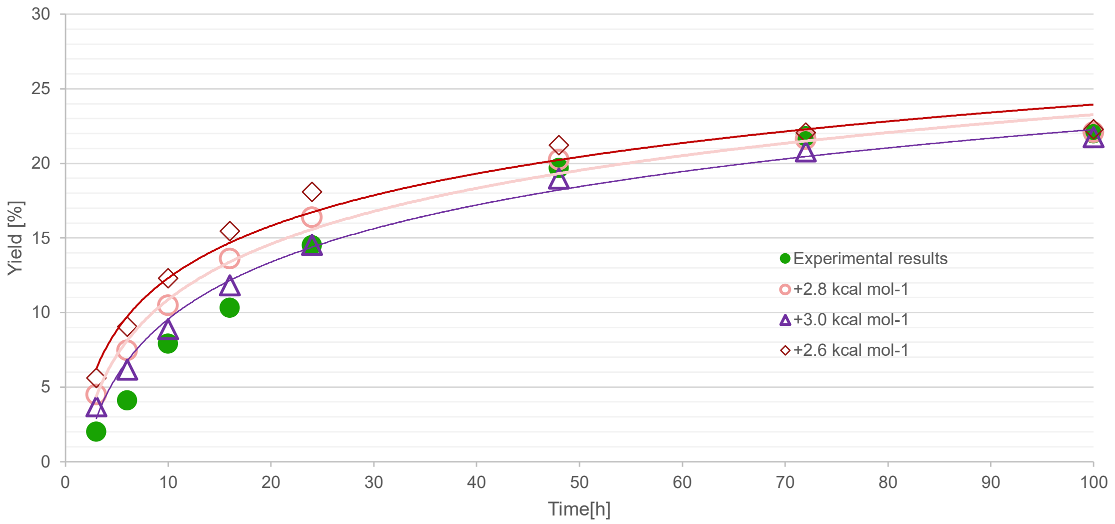

Figure S11. *Experimental and computed yield [%] of amidated methane at different reaction times (hours), considering* ***Mechanism A*** *at* ***373.15K****, with* ***correction1*** *= 2.6, 2.8 and 3.0 kcal.mol*$^{-1}$ *and* ***fit1A*** *= 7.4 kcal.mol*$^{-1}$*.*

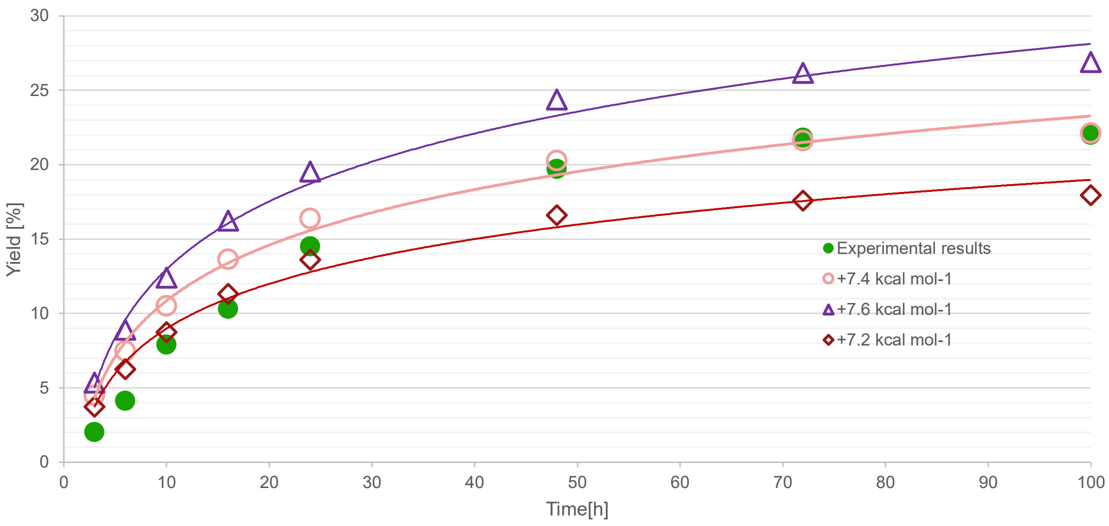

Figure S12. *Experimental and computed yield [%] of amidated methane at different reaction times (hours), considering* ***Mechanism A*** *at* ***373.15K****, with* ***correction1*** *= 2.8 kcal.mol*$^{-1}$ *and* ***fit1A*** *= 7.2, 7.4 and 7.6 kcal.mol*$^{-1}$.

However, when the same type of graph is produced for mechanism **C**, good agreement is achieved (see **Figures S13–S15**). In this case, we used the results leading to the lowest error (MAE): **correction1** of 2.2 kcal·mol^{-1} and **fit1C** and **fit2C** of 7.4 and 30 kcal·mol^{-1}, respectively (similar results were found for **correction1** = 2.3 kcal·mol^{-1}). We then increased/decreased **correction1** by 0.2 kcal·mol^{-1} (see **Figure S13**), **fit1C** by 0.1 kcal·mol^{-1} (see **Figure S14**), and **fit2C** by 0.2 kcal·mol^{-1} (see **Figure S15**). There is a very good agreement between the computational microkinetic model using mechanism **C** and the experimental results.

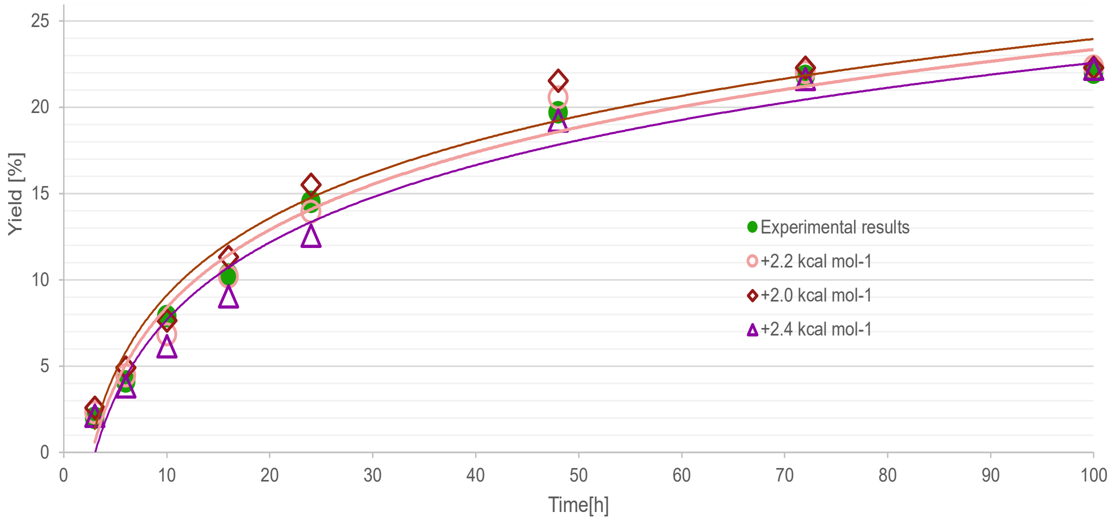

Figure S13. *Experimental and computed yield [%] of amidated methane at different reaction times (hours), considering* ***Mechanism C*** *at* ***373.15K****, with* ***correction1*** *= 2.0, 2.2 and 2.4 kcal.mol^{-1},* ***fit1C*** *= 7.4 kcal.mol^{-1},* ***fit2C*** *= 30 kcal.mol^{-1}.*

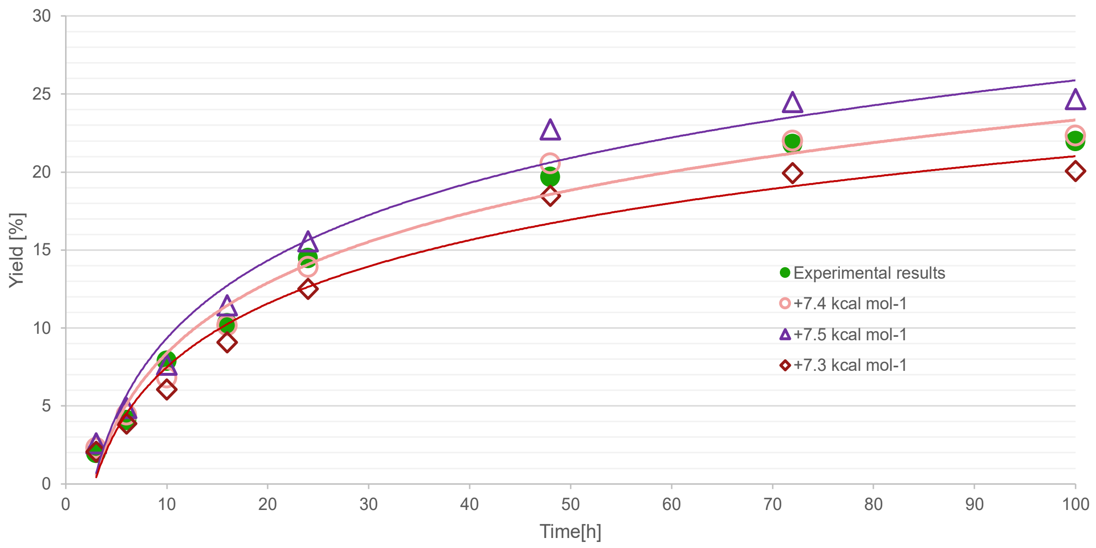

Figure S14. *Experimental and computed yield [%] of amidated methane at different reaction times (hours), considering* ***Mechanism C*** *at* ***373.15K****, with* ***correction1*** *= 2.2 kcal.mol^{-1},* ***fit1C*** *= 7.3, 7.4 and 7.5 kcal.mol^{-1},* ***fit2C*** *= 30.0 kcal.mol^{-1}.*

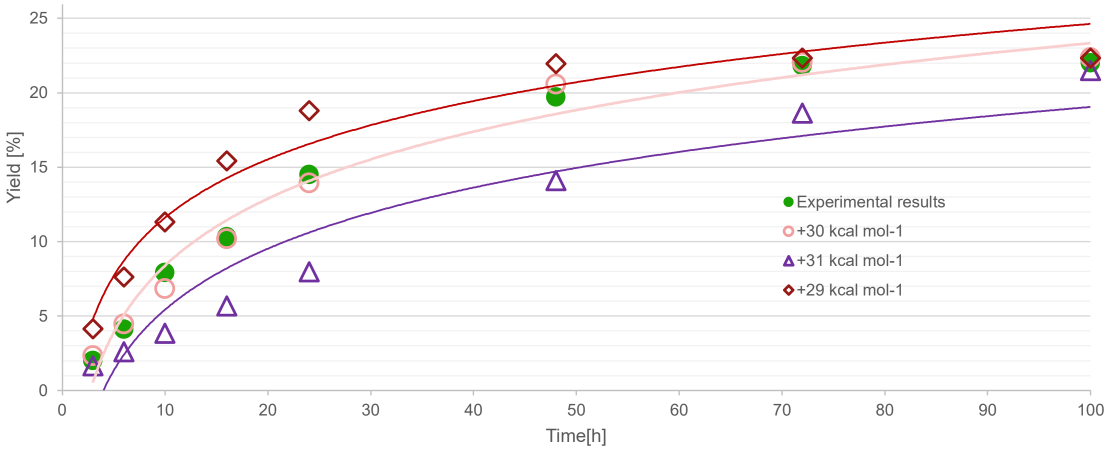

Figure S15. *Experimental and computed yield [%] of amidated methane at different reaction times (hours), considering* ***Mechanism C*** *at* ***373.15K****, with* ***correction1*** *= 2.2 kcal.mol^{-1},* ***fit1C*** *= 7.4 kcal.mol^{-1},* ***fit2C*** *= 29.0, 30.0 and 31.0 kcal.mol^{-1}.*

In **Figure S16** the results of two solutions for mechanism **C**: **correction1**, **fit1C**, **fit2C** =2.2, 7.4 and 30.0 and kcal·mol^{-1} and **correction1**, **fit1C** and **fit2C** = 0.0, 7.4 and 31.5 kcal·mol^{-1} are presented. Both solutions behave well, although the first leads to slightly better overall agreement, as evidenced by a lower MAE.

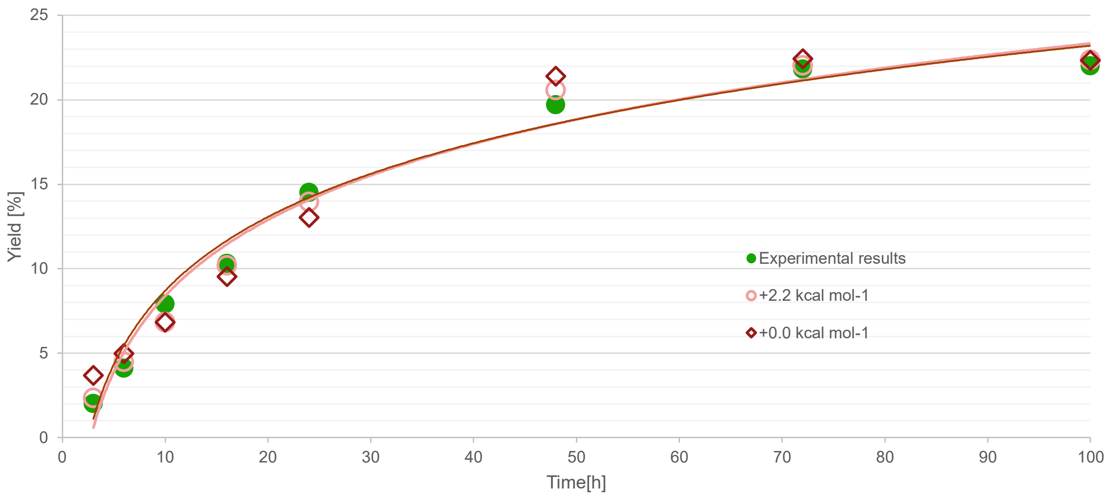

Figure S16. *Experimental and computed yield [%] of amidated methane at different reaction times (hours), considering* ***Mechanism C*** *at* ***373.15K****, for 2 adjustments: i) beige,* ***correction1*** *= 2.2 kcal·mol^{-1},* ***fit1C*** *= 7.4 kcal·mol^{-1},* ***fit2C*** *= 30.0 kcal·mol^{-1}; ii) brown,* ***correction1*** *= 0.0 kcal·mol^{-1},* ***fit1C*** *= 7.4 kcal·mol^{-1},* ***fit2C*** *= 31.5 kcal·mol^{-1}.*

15. f. Computational study of the hydrolysis first step's

In order to determine whether the reaction with water could be the key step in determining selectivity, we searched for the transition states for the insertion of the nitrene into the O-H bond of water. Both singlet and triplet transition states were considered. The triplet transition states $_{H2O}$**TS3-4**$_{ON}^{T}$ and $_{H2O}$**TS3-4**$_{N}^{T}$ (see **Figure S17**) were located at 4.6 and 6.5 kcal·mol^{-1} above **Int1**^{S} at 373.15K. The singlet transition state was found at higher energies (above 25 kcal·mol^{-1}).

According to the microkinetic model the barrier for the first step of the side reaction had a good fitting value of 7.4 kcal·mol^{-1} above **Int3**$_{ON}^{T}$, and -0.8 kcal·mol^{-1} relative to **Int1**. The transition states located for the reaction of water with the nitrene are approximately 5 kcal·mol^{-1} higher in energy than the value expected according to the **fit1C** value. This could be due to an oversimplification of the role of water, whereby the participation of additional water molecules could stabilize the formed radical, or because the transition state governing the process is not the located one. Further investigations would be necessary to reveal the details of the mechanism, as the current calculations do not allow us to hypothesise or rule out the involvement of water in this step.

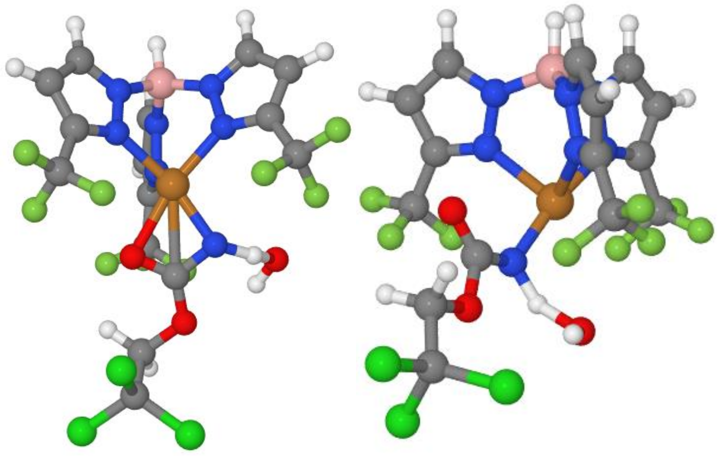

$_{H2O}$**TS3-4**$_{ON}^{T}$ $_{H2O}$**TS3-4**$_{N}^{T}$

Figure S17. *Ball and stick representation of the triplet transition states for the reaction of the copper nitrene with water.*

15. g. Summary of the results for the side reaction mechanism

The combination of results from experiments (yields and kinetic studies) and theory (mechanisms and free energy profiles) has enabled the computational reproduction of the experimental kinetic curves for methane through the construction of microkinetic models.

The experimental results can be explained by a two-step side reaction mechanism, such as mechanism **C**. Simpler mechanisms, such as mechanisms **A** and **B**, produce larger errors and cannot be well reproduced by the microkinetic models.

The results are consistent with a side reaction mechanism that begins with the nitrene intermediate reacting to form a side reaction intermediate (like **Int6** in **Scheme S2** and **S3**) via a low-barrier step that defines the selectivity. This step could include a water molecule, which would initiate hydrolysis. If water is included, the barrier for this first step should be close 6 – 7 kcal·mol^{-1}, whereas if water is not involved, the barrier should be close to 9 -10 kcal·mol^{-1}. This first step could be:

$\mathbf{Int3_N^T}$ + H_2O → subproduct + **Int6**, or

$\mathbf{Int3_N^T}$→ subproduct + **Int6**

The nitrene decomposition reaction would then proceed to the second step, which involves recovering the catalyst. According to our results, this second step would have associated a relatively large barrier, with values around 30 kcal·mol^{-1}, and would proceed as follows:

Int6 + alkane → subproduct2 + $\mathbf{Int1^S}$, or

Int6 → subproduct2 + $\mathbf{Int1^S}$

Similar results are obtained for mechanisms **C** and **D**, and our results agree with the idea of a low-energy step to define the selectivity, followed by an energy demanding step. The effect of the methane pressure in the observed experimental yield (see Scheme 3a in the main text) agree better with mechanism **C** than **D**.

It is also interesting to note the agreement between the computations and the experiments, which supports the idea that the energy barrier of the rate-limiting step is well captured. According to the results, the computed nitrene formation barrier probably has a small associated error of 2.2 kcal·mol^{-1} or less. Two solutions of the microkinetic model support mechanisms with corrections between 0 to 2.2 kcal·mol^{-1}.

Figure S18 and **S19** show how the reactants, products and intermediates evolve for the best microkinetic model of methane amidation (**correction1**= 2.2 kcal·mol^{-1}, **fit1C** =7.4 kcal·mol^{-1} and **fit2C** = 30 kcal·mol^{-1}). Please note that the concentrations of methane and water are not shown on the graph, as these remain approximately constant over time and are much higher than the other concentrations.

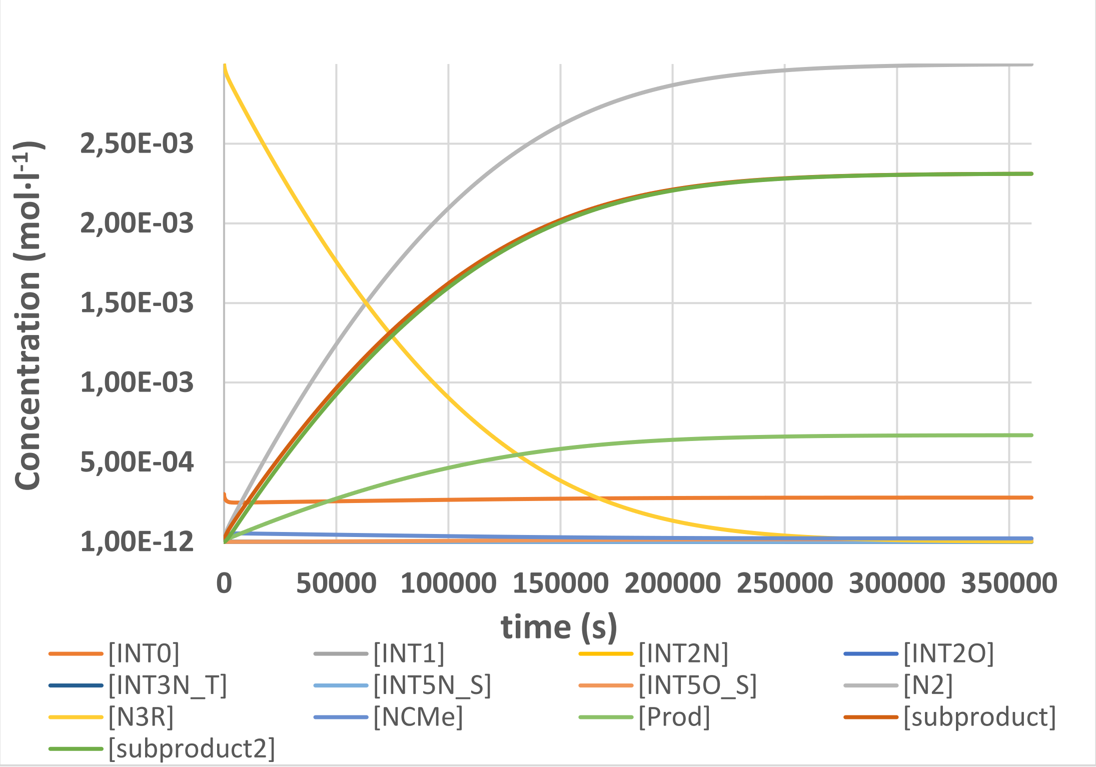

Figure 18. *Evolution of concentration (mol l^{-1}) of reactants, products and intermediates with time (s), using* **correction1**= 2.2 kcal·mol^{-1}, and mechanism **C** for side reaction: **fit1C** =7.4 kcal·mol^{-1} and **fit2C** = 30 kcal·mol^{-1}. *Concentrations of methane and water are not added for clarity.*

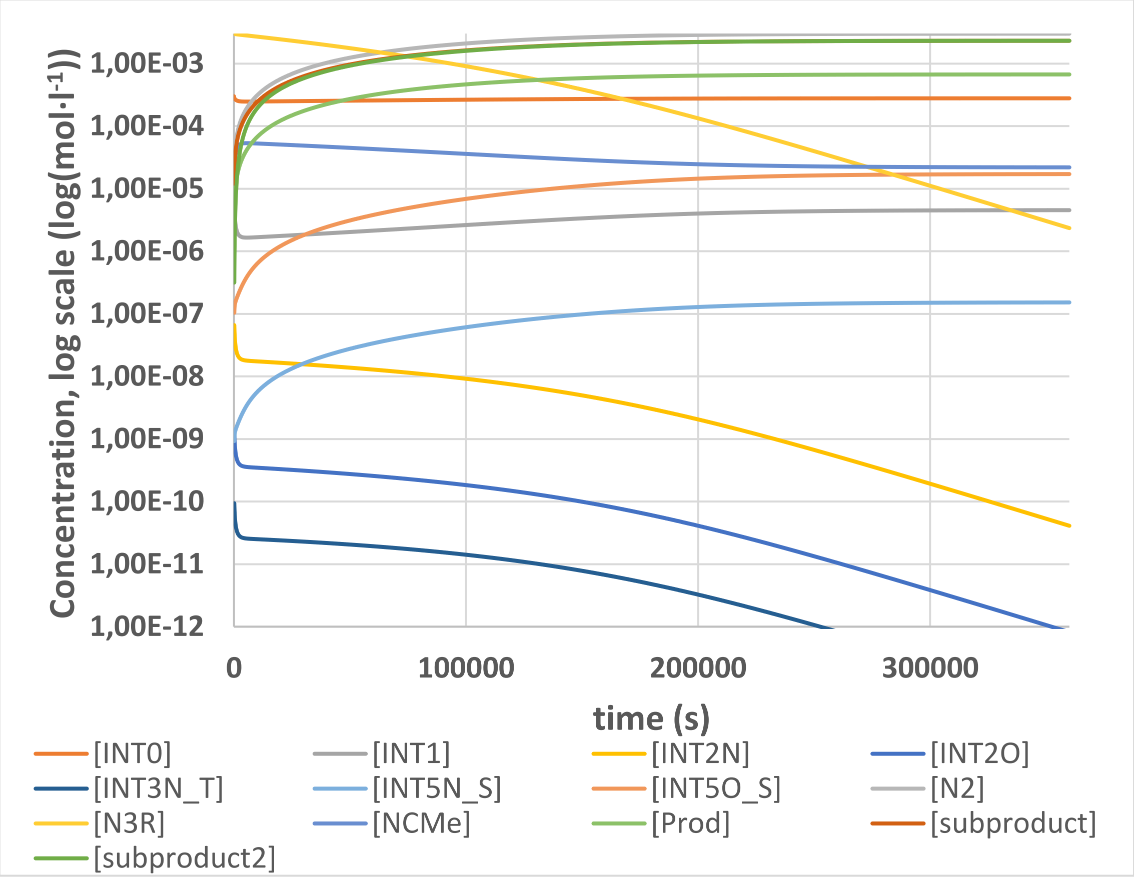

Figure 19. *Evolution of concentration (mol l^{-1}) of reactants, products and intermediates with time (s) in logarithmic scale, using* **correction1**= 2.2 kcal·mol^{-1}, and mechanism **C** for side reaction: **fit1C** =7.4 kcal·mol^{-1} and **fit2C** = 30 kcal·mol^{-1}. *For clarity concentrations of methane and water are not included (higher concentration than axis scale), as well as those of intermediates* ***Int3$_N^S$***, ***Int3$_{ON}^T$***, ***Int3$_{ON}^S$***, ***Int4$_N^T$***, ***Int4$_{ON}^T$*** *and* ***Int4$_{ON}^{OSS}$*** *(lower than axis scale).*

16. Computational prediction of the KIE for propane

The KIE has been experimentally measured for cyclohexane; however, this substrate has not yet been studied computationally. Instead, the secondary carbons of propane have been used to estimate the KIE, as it is expected that they will exhibit very similar behaviour when only the propane secondary carbons are considered. For propane, we found that the key transition states on the triplet and singlet surfaces had similar energies. (see **Table S10**). Therefore, we studied the KIE for the lowest-energy conformers of **ipTS3-4$_N$^{T}**, **ipTS3-4$_{ON}$^{T}** and **ipTS3-4$_{ON}$^{S}** at 353.15 K.

Please note that, according to our mechanistic study, the experimentally observed KIE is not associated with the rate-determining step (N_2 extrusion and copper nitrene formation), but with the selectivity-determining step (nitrene insertion into the C–H bond). Please also note that the level of theory used does not account for tunnelling effects, and that the main differences between hydrogen and deuterium are related to the entropic (and enthalpic) term, due to the change in mass.

Table S10. *Free energies barriers in kcal.mol^{-1} at 353.15 K and respect* ***Int3$_N$^{T}***.

	**ipTS3-4$_N$^{T}**	**ipTS3-4$_{ON}$^{T}**	**ipTS3-4$_{ON}$^{S}**
Hydrogen	14.5	15.8	15.9
Deuterium	15.6	16.9	16.7

The results show similar values for the barriers to nitrene insertion into C-H and C-D bonds, with slightly higher barriers for deuterium as expected. The experimental KIE is 2.1 for the two competing products. Computational modelling estimates this competition to be slightly larger, at KIE = 4.5, which shows good qualitative agreement and further supports the postulated mechanism.

17. Computational insight on the nitrene intermediate detection conditions

The key species of the free energy profile were optimized considering Tetrachloroethane (TCE) as the solvent (see Computational Details) and methanol as the substrate. The simplified free energy profile is presented in **Figure S20**. For the formation of the nitrene the energy cost is 26.9 kcal·mol^{-1}, similar to the one computed in *sc*CO_2. This barrier is difficult to overcome at 303.15 K and hence the formation of the nitrene is expected to be slow in these conditions. As no alkane is added, the reactivity could be with the added methanol.

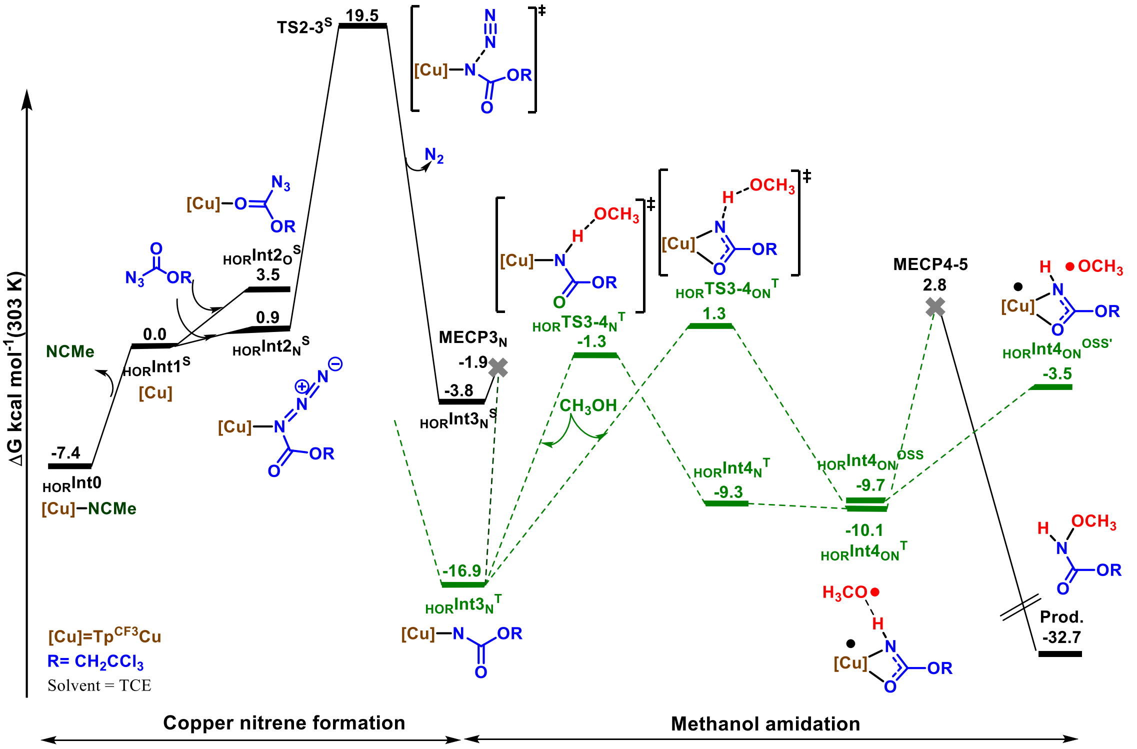

Figure S20. *Free energy profile for methanol amidation (303.15 K), in kcal mol^{-1}. In black solid lines the singlet surface is presented, the triplet surface is represented in dark and sea green dashed lines instead.*

The triplet nitrene intermediate computed in TCE, $_{\mathrm{HOR}}$**Int3**$_{\mathrm{N}}^{\mathrm{T}}$, has barriers of more than 15 kcal·mol^{-1} for the insertion of the nitrene in the triplet potential energy surface. The reactivity on the singlet PES requires high energies and has not been included for simplicity. Interestingly, the located $_{\mathrm{HOR}}$**Int4**$_{\mathrm{ON}}^{\mathrm{T}}$ and $_{\mathrm{HOR}}$**Int4**$_{\mathrm{ON}}^{\mathrm{OSS}}$ intermediates form a strong interaction between the recently added nitrogen and the oxygen of the formed radical, [Cu]-N(R)--·OMe with a hydrogen – oxygen distances of 1.970 Å and 1.974 Å respectively. For the radical rebound, this interaction has to be broken. The corresponding open shell singlet $_{\mathrm{HOR}}$**Int4**$_{\mathrm{ON}}^{\mathrm{OSS}}$ is located at higher energies. Different subproducts could

be obtained from the reactivity with methanol. But as they were not experimentally detected in significant amounts the different possibilities have not been explored in this occasion. As far as the product formed is significantly thermodynamically favored vs. nitrene, and shares the firsts steps with the reported mechanism, the microkinetic model would also reproduce its reactivity. Also, the amidation of tetrachloroethane C-H bonds has not been explored as it is not experimentally observed and is expected to require higher energies than that of methanol.

A microkinetic model was built under the experimental conditions of 303.15 K, using computational free energies (**Figure S20**), with initial concentrations of [N_3Troc] = 0.001 mol·l^{-1}, [Int0] = 1.0×10^{-5} mol·l^{-1}, [water]= 5.55×10^{-5} mol·l^{-1} and [methanol] = 0.0247 mol·l^{-1}. Note that we have included the nitrene decomposition side reaction in the microkinetic model considering, water is present from the 0.1% methanol and with the best fitted values for the side reaction, following mechanism **C** (see above). We used the best-fitting parameters to reproduce the experimental curve for methane (**correction1** = 2.2 kcal·mol^{-1}, **fit1C** = 7.4 kcal·mol^{-1} and **fit2C** = 30 kcal·mol^{-1}), as well as the best-fitting parameters to reproduce the experimental yield for ethane considering mechanism **C** (**correction1** = 0.0 kcal·mol^{-1}, **fit1C** = 6.2 kcal·mol^{-1} and **fit2C** = 31 kcal·mol^{-1}).

The microkinetic model results show that, after 10 minutes of reaction, the concentrations of the intermediates **Int3$_N^T$** and **Int2$_N^S$** according to our calculations are of the order of 1×10^{-9} mol·l^{-1} for both considering ethane fitted parameters (see **Figure S21** and **S22**), and of 1×10^{-10} and 1×10^{-9} mol·l^{-1} respectively when the methane fitted parameters are used instead (see **Figure S23** and **S24**). In both cases, these concentrations would therefore be detectable in HMRS-ESI.

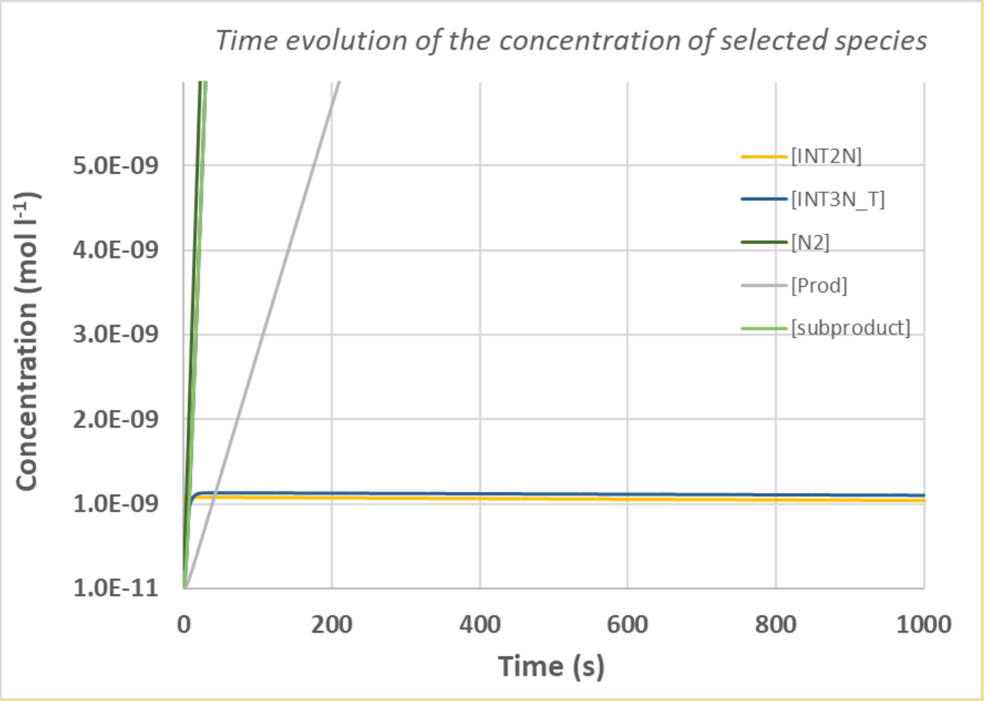

Figure 21. *Evolution of concentration (mol·l^{-1}) of selected species with time (s) for* ***correction1****= 0.0 kcal·mol^{-1},* ***fit1C*** *=6.2 kcal·mol^{-1} and* ***fit2C*** *= 31 kcal·mol^{-1}. For clarity only species with concentrations between 6.0×10^{-9} and 1.0×10^{-11}mol·l^{-1} have been included.*

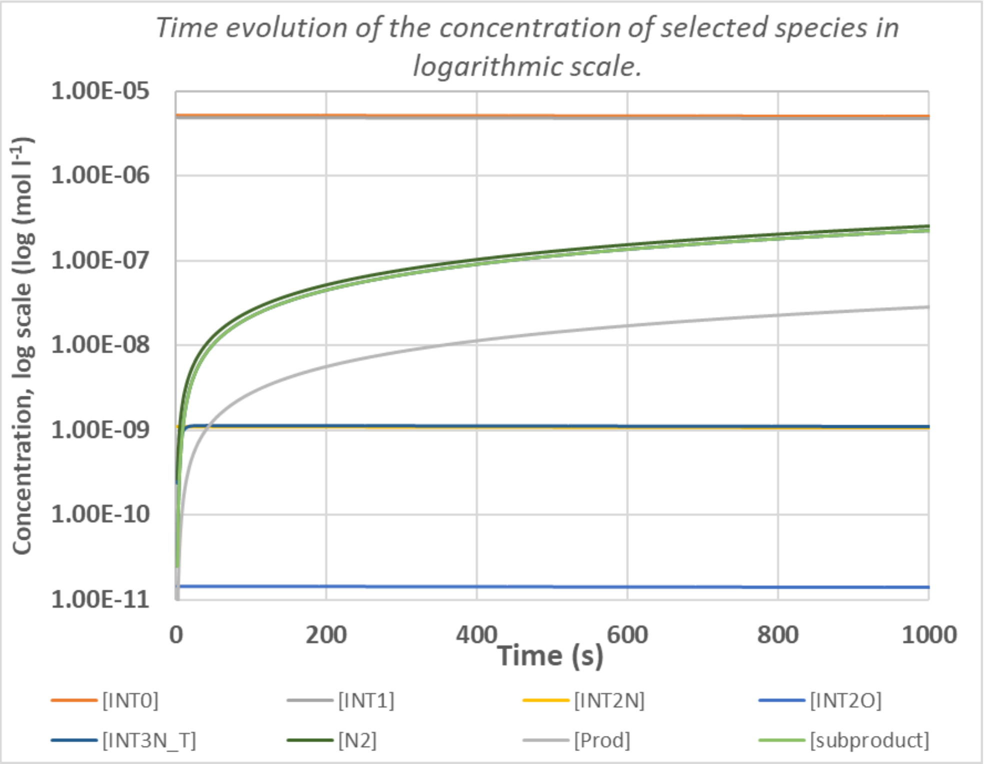

Figure S22. *Evolution of concentration (mol·l^{-1}) of selected species with time (s) in logarithmic scale, for* ***correction1****= 0.0 kcal·mol^{-1},* ***fit1C*** *=6.2 kcal·mol^{-1} and* ***fit2C*** *= 31 kcal·mol^{-1}. For clarity only species with concentrations between 1.0×10^{5} and 1.0×10^{-11}mol·l^{-1} have been included. Please note evolution of* ***Int2$_N$*** *and* ***Int3$_N^T$*** *are overlapped in this time scale.*

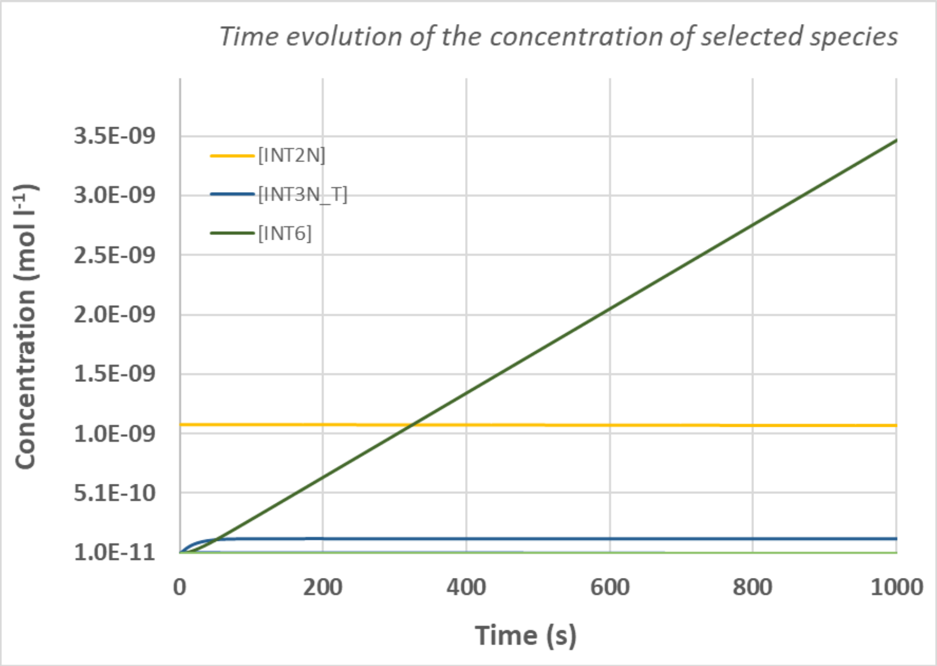

Figure S23. *Evolution of concentration (mol·l^{-1}) of selected species with time (s) for* ***correction1****= 2.2 kcal·mol^{-1},* ***fit1C*** *=7.4 kcal·mol^{-1} and* ***fit2C*** *= 30 kcal·mol^{-1}. For clarity only species with concentrations between 4.0×10^{-9} and 1.0×10^{-11}mol·l^{-1} have been included.*

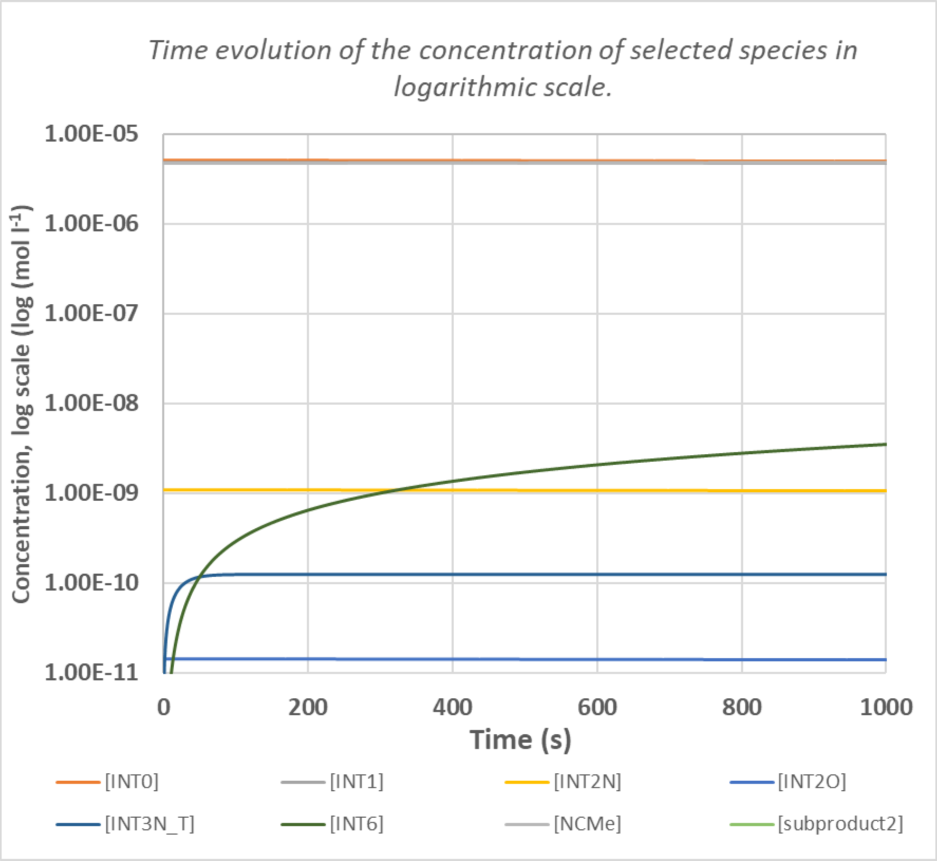

Figure S24. *Evolution of concentration (mol·l^{-1}) of selected species with time (s) in logarithmic scale, for* ***correction1****= 2.2 kcal·mol^{-1},* ***fit1C*** *=7.4 kcal·mol^{-1} and* ***fit2C*** *= 30 kcal·mol^{-1}. For clarity only species with concentrations between 1.0×10^{-5} and 1.0×10^{-11}mol·l^{-1} have been included.*

18. Cartesian coordinates (in Ångström, Å) and potential energies (in Hartree) of the optimized structures

NCMe
E = -132.756598490 Hartree
Atom X Y Z
1 N -3.3328 0.0039 0.0961
2 C -3.4733 0.1036 -1.0512
3 C -3.6502 0.2295 -2.4952
4 H -2.6938 0.0811 -3.0074
5 H -4.3623 -0.5200 -2.8558
6 H -4.0316 1.2254 -2.7439

N3Troc
E = -475.037954112 Hartree
Atom X Y Z
1 N -2.8664 -0.3246 -1.5077
2 C -3.9873 0.3767 -1.0345
3 O -3.9434 1.0731 -0.0549
4 N -3.0427 -1.0596 -2.5061
5 N -3.0466 -1.7615 -3.3928
6 O -5.0785 0.1779 -1.8308
7 C -6.3159 0.7255 -1.3905
8 C -7.2695 -0.4057 -0.9704
9 H -6.1647 1.3989 -0.5445
10 H -6.7609 1.2606 -2.2319
11 Cl -6.5815 -1.3212 0.4103
12 Cl -8.8216 0.3588 -0.4833
13 Cl -7.5572 -1.5287 -2.3426

N_2
E = -109.515658220 Hartree
Atom X Y Z
1 N -3.0732 -1.0932 -2.5021
2 N -3.1186 -1.7954 -3.3541

Int0
E = -2042.73675944 Hartree
Atom X Y Z
1 C 0.8635 0.3369 2.9266
2 N 0.7068 0.3610 1.5802
3 N -0.4748 -0.1906 1.2336
4 C -1.0594 -0.5612 2.3742
5 C -0.2591 -0.2535 3.4867
6 Cu -0.9502 -0.2258 -0.8653
7 N -0.2090 1.7982 -0.9432
8 N 0.9419 2.0485 -0.2853
9 C 1.3022 3.3465 -0.4385
10 C 0.3506 3.9736 -1.2283
11 C -0.5688 2.9503 -1.5118
12 B 1.6645 0.9200 0.4960
13 N 2.0350 -0.2202 -0.4883
14 N 1.0759 -0.8601 -1.1892
15 C 1.7055 -1.7731 -1.9309
16 C 3.0937 -1.7388 -1.7206
17 C 3.2566 -0.7266 -0.7870
18 C 0.9059 -2.6534 -2.8266
19 H 3.8508 -2.3561 -2.1794
20 H 4.1459 -0.3329 -0.3167
21 C -1.8174 2.9991 -2.3212
22 H 0.3197 5.0029 -1.5509
23 H 2.2023 3.7264 0.0224
24 C -2.3990 -1.2086 2.3226
25 H -0.4714 -0.4358 4.5290
26 H 1.7556 0.7385 3.3847
27 H 2.6573 1.3528 1.0120
28 F -2.8402 -1.5280 3.5709
29 F -3.3512 -0.4089 1.7557
30 F -2.4007 -2.3621 1.5906
31 F -2.0270 4.2455 -2.8288
32 F -1.7954 2.1357 -3.3801
33 F -2.9298 2.6706 -1.5988
34 F 1.7053 -3.5242 -3.5029
35 F -0.0150 -3.4008 -2.1484
36 F 0.1976 -1.9528 -3.7618
37 N -2.5593 -0.8810 -1.7087
38 C -3.5278 -1.2640 -2.2143
39 C -4.7453 -1.7442 -2.8504
40 H -5.6115 -1.2082 -2.4486
41 H -4.6913 -1.5772 -3.9312
42 H -4.8676 -2.8155 -2.6593

Nitrene formation---

Int1_singlet
E = -1909.93813070 Hartree
Atom X Y Z
1 C 0.9110 0.3364 2.9525
2 N 0.6986 0.3590 1.6136
3 N -0.5321 -0.1247 1.3275
4 C -1.0865 -0.4475 2.4979
5 C -0.2176 -0.1808 3.5680
6 Cu -0.9389 -0.0264 -0.7405
7 N -0.2163 1.9490 -0.8579
8 N 0.9771 2.0792 -0.2338
9 C 1.4498 3.3406 -0.3855
10 C 0.5363 4.0656 -1.1340
11 C -0.4832 3.1384 -1.4021
12 B 1.6453 0.8810 0.4983
13 N 1.9349 -0.2548 -0.5228
14 N 0.9391 -0.8460 -1.2220
15 C 1.5247 -1.7404 -2.0213
16 C 2.9182 -1.7465 -1.8489
17 C 3.1331 -0.7778 -0.8816
18 C 0.6861 -2.6087 -2.8941
19 H 3.6477 -2.3587 -2.3569
20 H 4.0464 -0.4213 -0.4280
21 C -1.7690 3.3447 -2.1244
22 H 0.5942 5.0974 -1.4455
23 H 2.3984 3.6274 0.0446
24 C -2.4412 -1.0659 2.5230
25 H -0.3939 -0.3373 4.6213
26 H 1.8430 0.6917 3.3672
27 H 2.6766 1.2435 0.9902
28 F -3.0768 -0.8271 3.7016
29 F -3.2411 -0.5961 1.5221
30 F -2.4047 -2.4244 2.3706
31 F -1.6658 4.3331 -3.0533
32 F -2.1847 2.2163 -2.7691
33 F -2.7921 3.7022 -1.2904
34 F 1.3625 -2.9975 -4.0087
35 F 0.2829 -3.7551 -2.2671
36 F -0.4540 -1.9813 -3.3037

Int2$_O$_singlet
E = -2385.01050323 Hartree
Atom X Y Z
1 C 0.9348 -0.1345 3.5196
2 N 0.7311 0.2461 2.2337
3 N -0.5872 0.2506 1.9457
4 C -1.2106 -0.1308 3.0622
5 C -0.2981 -0.3896 4.0992
6 Cu -1.0413 0.7534 -0.1201
7 O -3.0208 0.6566 -0.4602
8 C -3.6858 0.8226 -1.4685
9 N -3.1649 1.4599 -2.5829
10 N -3.9032 1.5211 -3.5959
11 N -4.4371 1.6540 -4.5816
12 B 1.7807 0.6063 1.1499
13 N 1.5045 2.0357 0.6089
14 N 0.3481 2.3266 -0.0254
15 C 0.4202 3.6171 -0.3587
16 C 1.6349 4.1865 0.0564
17 C 2.2954 3.1348 0.6727
18 C -0.6984 4.2383 -1.1205
19 H 1.9701 5.2042 -0.0732
20 H 3.2635 3.0894 1.1498
21 C -2.6962 -0.2305 3.0736
22 H -0.5118 -0.7099 5.1074
23 H 1.9344 -0.1957 3.9247
24 N 0.5267 -0.5721 -0.7081
25 N 1.6787 -0.4097 -0.0210
26 C 2.6470 -1.2145 -0.5243
27 C 2.1130 -1.9332 -1.5816
28 C 0.7835 -1.4843 -1.6488
29 C -0.3020 -1.9404 -2.5594
30 H 2.6007 -2.6622 -2.2107
31 H 3.6397 -1.2140 -0.0982
32 O -4.9695 0.4389 -1.5915
33 C -5.5239 -0.3555 -0.5332
34 C -5.6441 -1.8204 -0.9846
35 Cl -6.7067 -1.9424 -2.4325
36 Cl -4.0337 -2.4909 -1.3703
37 Cl -6.3822 -2.7260 0.3804
38 H 2.8815 0.5631 1.6245
39 H -4.8981 -0.3058 0.3591
40 H -6.5204 0.0390 -0.3313
41 F -3.1532 -0.6190 4.2956
42 F -3.3128 0.9516 2.7750
43 F -3.1780 -1.1349 2.1655
44 F -0.5812 5.5948 -1.1481
45 F -0.7484 3.8228 -2.4226
46 F -1.9223 3.9482 -0.5905
47 F 0.2002 -2.4330 -3.7245
48 F -1.0625 -2.9450 -2.0166
49 F -1.1755 -0.9436 -2.8805

Int2$_N$_singlet
E = -2385.01380909 Hartree
Atom X Y Z
1 C 0.8601 0.2284 2.9532
2 N 0.6588 0.3697 1.6195
3 N -0.5716 -0.0710 1.2784
4 C -1.1391 -0.4939 2.4102
5 C -0.2804 -0.3268 3.5100
6 Cu -0.9933 0.0346 -0.8372
7 N -2.8294 -0.3953 -1.5885
8 N -3.0450 -1.3517 -2.3852
9 N -3.1043 -2.2310 -3.0893
10 B 1.6406 0.9078 0.5473
11 N 1.0098 2.1256 -0.1763
12 N -0.1551 2.0140 -0.8525
13 C -0.3918 3.2174 -1.3799
14 C 0.6199 4.1337 -1.0456
15 C 1.4959 3.3877 -0.2749
16 C -1.6270 3.4705 -2.1738
17 H 0.6962 5.1719 -1.3311
18 H 2.4255 3.6586 0.2040
19 C -2.5341 -1.0171 2.4023
20 H -0.4668 -0.5798 4.5426

21 H 1.7954 0.5267 3.4039
22 N 0.9191 -0.7491 -1.2164
23 N 1.9240 -0.2138 -0.4926
24 C 3.1018 -0.7977 -0.8215
25 C 2.8613 -1.7530 -1.7985
26 C 1.4751 -1.6735 -2.0025
27 C 0.5960 -2.4482 -2.9184
28 H 3.5694 -2.4042 -2.2878
29 H 4.0199 -0.4938 -0.3401
30 C -3.9561 0.3677 -1.1909
31 O -5.0755 -0.0464 -1.8295
32 C -6.3098 0.5541 -1.4363
33 C -7.1556 -0.4554 -0.6415
34 Cl -7.4922 -1.9117 -1.6408
35 O -3.8580 1.2648 -0.3997
36 Cl -6.3041 -0.9401 0.8539
37 Cl -8.7042 0.3633 -0.2331
38 H 2.6685 1.2374 1.0702
39 H -6.1299 1.4372 -0.8206
40 H -6.8447 0.8226 -2.3485
41 F -2.7295 -1.9163 3.4086
42 F -3.4743 -0.0415 2.5762
43 F -2.8512 -1.6454 1.2328
44 F -1.4069 4.4134 -3.1358
45 F -2.0847 2.3513 -2.8066
46 F -2.6651 3.9350 -1.4197
47 F 1.3053 -3.3122 -3.6864
48 F -0.3450 -3.1830 -2.2459
49 F -0.1179 -1.6417 -3.7653

TS2-3_singlet
E = -2384.97583714 Hartree
Atom X Y Z
1 C 1.0612 0.5539 2.8835
2 N 0.7699 0.4324 1.5668
3 N -0.3389 -0.3257 1.3996
4 C -0.7343 -0.6828 2.6256
5 C 0.1152 -0.1511 3.6080
6 Cu -0.9614 -0.6043 -0.5909
7 N -2.7663 -0.9226 -0.9422
8 N -3.1885 -1.6276 -2.5030
9 N -2.8666 -2.3144 -3.3150
10 B 1.5403 1.0305 0.3633
11 N 0.6363 2.0276 -0.4001
12 N -0.5193 1.6140 -0.9606
13 C -1.0144 2.6745 -1.6024
14 C -0.1850 3.8000 -1.4580
15 C 0.8629 3.3354 -0.6802
16 C -2.3007 2.5653 -2.3454
17 H -0.3300 4.7882 -1.8670
18 H 1.7486 3.8335 -0.3131
19 C -1.9405 -1.5340 2.8509
20 H 0.0478 -0.2766 4.6780
21 H 1.9194 1.1255 3.2054
22 N 0.9888 -0.9024 -1.1652
23 N 1.9357 -0.1206 -0.6061
24 C 3.1601 -0.4789 -1.0571
25 C 3.0117 -1.5348 -1.9459
26 C 1.6277 -1.7554 -1.9711
27 C 0.8315 -2.7165 -2.7845
28 H 3.7786 -2.0653 -2.4892
29 H 4.0443 0.0391 -0.7154
30 C -3.8271 -0.1750 -0.5314
31 O -4.9227 -0.1500 -1.3163
32 C -5.9600 0.7090 -0.8470
33 C -7.0216 0.7938 -1.9471
34 Cl -6.3287 1.4829 -3.4497
35 O -3.7122 0.4015 0.5416
36 Cl -7.7094 -0.8298 -2.2940
37 Cl -8.3258 1.8744 -1.3352
38 H 2.5276 1.5876 0.7521
39 H -6.4185 0.3043 0.0592
40 H -5.5712 1.7102 -0.6495
41 F 1.6163 -3.6747 -3.3364
42 F -0.1337 -3.3453 -2.0475
43 F 0.1662 -2.1024 -3.8110
44 F -1.7064 -2.4418 3.8476
45 F -3.0286 -0.8177 3.2459
46 F -2.3023 -2.2371 1.7473
47 F -2.4546 3.5952 -3.2199
48 F -2.3951 1.4063 -3.0600
49 F -3.3989 2.5880 -1.5257

Int3$_N$_singlet
E = -2275.46852555 Hartree
Atom X Y Z
1 C -0.9439 -1.9817 -2.2707
2 N -0.8788 -1.1640 -1.1957
3 N 0.1565 -1.5225 -0.4056
4 C 0.7376 -2.5775 -0.9865
5 C 0.0817 -2.9109 -2.1790
6 Cu 0.4761 -0.3442 1.2681
7 N 2.2542 -0.2876 1.4780
8 C 3.1738 0.2962 0.7325
9 O 4.2698 0.7364 1.3918
10 C 5.2634 1.3883 0.6102
11 C 6.5173 0.5049 0.4859
12 Cl 7.2078 0.1549 2.1072
13 B -1.8235 -0.0010 -0.7955
14 N -1.0461 1.3326 -0.7835
15 N -0.0451 1.5326 0.1007
16 C 0.3759 2.7841 -0.0969
17 C -0.3475 3.4114 -1.1257
18 C -1.2483 2.4430 -1.5348
19 C 1.4758 3.3691 0.7237
20 H -0.2339 4.4190 -1.4958
21 H -2.0183 2.4641 -2.2922
22 C 1.9047 -3.2644 -0.3532
23 H 0.3174 -3.7176 -2.8557
24 H -1.7161 -1.8492 -3.0143
25 N -1.4922 -0.4323 1.6819
26 N -2.3469 -0.2779 0.6452
27 C -3.6216 -0.3715 1.0827
28 C -3.6057 -0.5961 2.4529
29 C -2.2435 -0.6250 2.7719
30 C -1.5854 -0.8259 4.0955
31 H -4.4429 -0.7225 3.1215
32 H -4.4458 -0.2731 0.3916
33 O 2.9661 0.3908 -0.4846
34 Cl 6.1283 -1.0331 -0.3399
35 Cl 7.7223 1.4332 -0.4857
36 H -2.7420 0.0581 -1.5609
37 H 4.8945 1.6150 -0.3915
38 H 5.5362 2.3095 1.1286
39 F -2.5014 -1.0663 5.0665
40 F -0.7149 -1.8767 4.0812
41 F -0.8576 0.2621 4.4805
42 F 1.2716 4.7006 0.9333
43 F 1.5878 2.7758 1.9411
44 F 2.7066 3.2685 0.1320
45 F 2.0566 -4.5140 -0.8760
46 F 3.0834 -2.6155 -0.5437
47 F 1.7444 -3.4029 0.9912

Int3$_N$_triplet
E = -2275.48546156 Hartree
Atom X Y Z
1 C 0.9584 0.3591 2.9062
2 N 0.7207 0.3524 1.5719
3 N -0.3959 -0.3600 1.2921
4 C -0.8514 -0.8042 2.4662
5 C -0.0352 -0.3771 3.5272
6 Cu -0.9222 -0.3855 -0.8835
7 N -2.7295 -0.6180 -1.1101
8 C -3.8289 0.0579 -0.6625
9 O -4.7120 0.2910 -1.6692
10 C -5.8656 1.0577 -1.3548
11 C -7.1239 0.1725 -1.3826
12 Cl -7.3429 -0.5634 -3.0066
13 B 1.5722 0.9689 0.4426
14 N 0.7346 2.0179 -0.3359
15 N -0.3890 1.6665 -0.9995
16 C -0.8535 2.7854 -1.5686
17 C -0.0330 3.8819 -1.2717
18 C 0.9705 3.3414 -0.4820
19 C -2.1112 2.7736 -2.3692
20 H -0.1546 4.9043 -1.5948
21 H 1.8319 3.8000 -0.0190
22 C -2.0730 -1.6588 2.5662
23 H -0.1538 -0.5884 4.5791
24 H 1.8156 0.8779 3.3103
25 N 1.0363 -0.8739 -1.1977
26 N 1.9790 -0.1596 -0.5449
27 C 3.2106 -0.6036 -0.8786
28 C 3.0741 -1.6473 -1.7843
29 C 1.6894 -1.7708 -1.9457
30 C 0.9164 -2.7212 -2.7964
31 H 3.8490 -2.2302 -2.2575
32 H 4.0918 -0.1489 -0.4504
33 O -3.9802 0.3839 0.5038
34 Cl -7.0167 -1.1239 -0.1522
35 Cl -8.5313 1.2407 -1.0183
36 H 2.5505 1.4893 0.8959
37 H -5.7805 1.5123 -0.3660
38 H -5.9683 1.8279 -2.1220
39 F 1.7432 -3.5402 -3.4932
40 F 0.0820 -3.5105 -2.0605
41 F 0.1236 -2.0735 -3.7000
42 F -1.8588 -2.7001 3.4290
43 F -3.1496 -0.9860 3.0545
44 F -2.4338 -2.1964 1.3744
45 F -2.1779 3.8545 -3.1900
46 F -2.2273 1.6607 -3.1415
47 F -3.2337 2.8147 -1.5838

Int3$_{ON}$_singlet
E = -2275.47922213 Hartree
Atom X Y Z
1 C 1.0778 0.0770 3.2876
2 N 0.7735 0.2259 1.9798
3 N -0.4729 -0.2356 1.7391
4 C -0.9478 -0.6833 2.9080
5 C -0.0059 -0.5064 3.9289
6 Cu -1.1980 -0.1333 -0.1678
7 O -3.0784 0.1897 0.0815
8 C -3.2291 0.1857 -1.2122
9 O -4.3894 0.4319 -1.8174
10 C -5.5207 0.6987 -0.9878
11 C -6.4706 -0.5128 -0.9591
12 Cl -7.0352 -0.9076 -2.6164
13 B 1.6384 0.8225 0.8361
14 N 0.9717 2.1041 0.3050
15 N -0.2814 2.0551 -0.1939
16 C -0.5629 3.2986 -0.5883
17 C 0.5117 4.1737 -0.3483
18 C 1.4746 3.3616 0.2264
19 C -1.8635 3.6149 -1.2461
20 H 0.5649 5.2305 -0.5601
21 H 2.4698 3.5826 0.5840
22 C -2.3165 -1.2750 3.0041
23 H -0.1029 -0.7765 4.9690
24 H 2.0396 0.3907 3.6658
25 N 0.5920 -0.7782 -0.8403

26 N 1.7081 -0.2337 -0.3039
27 C 2.8034 -0.7644 -0.8895
28 C 2.3936 -1.6907 -1.8371
29 C 0.9959 -1.6616 -1.7616
30 C 0.0259 -2.4905 -2.5432
31 H 3.0038 -2.3100 -2.4761
32 H 3.7916 -0.4518 -0.5854
33 N -2.1185 -0.0517 -1.8361
34 Cl -5.6577 -1.9397 -0.2488
35 Cl -7.8821 -0.0455 0.0600
36 H 2.7418 1.0571 1.2371
37 H -5.2229 0.9377 0.0345
38 H -6.0469 1.5440 -1.4341
39 F -2.4497 -1.9842 4.1581
40 F -3.3024 -0.3369 3.0033
41 F -2.5786 -2.1232 1.9715
42 F -2.0876 4.9594 -1.2540
43 F -1.9187 3.2032 -2.5446
44 F -2.9298 3.0355 -0.6196
45 F 0.6449 -3.6152 -3.0068
46 F -1.0280 -2.8948 -1.7849
47 F -0.4777 -1.8519 -3.6292

Int3$_{ON}$_triplet
E = -2275.47696476 Hartree
Atom X Y Z
1 C 1.1028 0.0241 3.2557
2 N 0.7652 0.3451 1.9876
3 N -0.5691 0.2030 1.8111
4 C -1.0617 -0.2167 2.9835
5 C -0.0467 -0.3450 3.9390
6 Cu -1.3198 0.6071 -0.0672
7 O -3.5407 0.9905 0.1911
8 C -3.5214 0.8228 -1.0526
9 O -4.5816 0.4613 -1.7868
10 C -5.7795 0.0848 -1.1054
11 C -6.0094 -1.4310 -1.2335
12 Cl -6.1081 -1.9134 -2.9586
13 B 1.6947 0.7958 0.8268
14 N 1.2722 2.2059 0.3645
15 N 0.0057 2.4343 -0.0441
16 C -0.0472 3.7249 -0.3783
17 C 1.1953 4.3540 -0.1914
18 C 2.0097 3.3406 0.2867
19 C -1.3058 4.3187 -0.9164
20 H 1.4474 5.3881 -0.3703
21 H 3.0494 3.3443 0.5801
22 C -2.5210 -0.4847 3.1481
23 H -0.1434 -0.6667 4.9643
24 H 2.1321 0.0765 3.5785
25 N 0.3065 -0.4633 -0.8716
26 N 1.5245 -0.2071 -0.3465
27 C 2.4702 -0.9240 -0.9960
28 C 1.8508 -1.6786 -1.9812
29 C 0.4940 -1.3494 -1.8542
30 C -0.6595 -1.8380 -2.6664
31 H 2.3003 -2.3688 -2.6785
32 H 3.5087 -0.8497 -0.7084
33 N -2.3509 1.0458 -1.6968
34 Cl -4.6895 -2.3330 -0.4332
35 Cl -7.5756 -1.7926 -0.4176
36 H 2.8341 0.8011 1.1955
37 H -5.7339 0.3484 -0.0473
38 H -6.6047 0.6059 -1.5944
39 F -0.3805 -3.0478 -3.2242
40 F -1.7877 -1.9861 -1.9135
41 F -0.9832 -1.0007 -3.6889
42 F -2.7558 -1.1672 4.3019
43 F -3.2675 0.6515 3.2015
44 F -3.0199 -1.2334 2.1243
45 F -1.3476 5.6620 -0.6802
46 F -1.4328 4.1619 -2.2651
47 F -2.4192 3.7732 -0.3561

Int4$_{N}$_triplet
E = -2315.99931204 Hartree
Atom X Y Z
1 C 1.5927 -3.0640 -2.8408
2 N 1.6786 -2.3503 -1.7124
3 N 2.8148 -2.7401 -1.0949
4 C 3.4383 -3.6872 -1.8299
5 C 2.6856 -3.9296 -2.9708
6 Cu 0.4985 -0.9553 -0.7917
7 N 2.5273 0.1621 -0.5888
8 N 3.4714 -0.5896 0.0230
9 C 4.5740 0.1530 0.2953
10 C 4.3490 1.4397 -0.1591
11 C 3.0535 1.3820 -0.7041
12 B 3.2488 -2.0952 0.2534
13 N 2.1034 -2.3273 1.2782
14 N 0.8339 -1.9652 0.9972
15 C 0.0875 -2.3461 2.0399
16 C 0.8765 -2.9573 3.0226
17 C 2.1577 -2.9230 2.4905
18 C -1.3869 -2.1206 2.0395
19 F -1.9913 -2.8700 2.9990
20 C 2.3317 2.4890 -1.3978
21 F 2.0289 3.5305 -0.5742
22 C 0.4601 -2.8351 -3.7832
23 F 0.2254 -3.9301 -4.5503
24 N -0.9108 0.2581 -0.5940
25 C -0.7894 1.3953 0.1452
26 O -1.9365 2.1632 0.0549
27 C -1.9256 3.4093 0.7202
28 C -2.9498 3.4205 1.8678
29 Cl -4.6097 3.1117 1.2420
30 O 0.1917 1.7381 0.7819
31 Cl -2.5444 2.1821 3.0941
32 Cl -2.8994 5.0602 2.6198
33 F -1.7335 -0.8243 2.2744
34 F -1.9541 -2.4565 0.8428
35 F 3.1105 3.0233 -2.3932
36 F 1.1764 2.0834 -1.9824
37 F -0.6976 -2.5370 -3.1216
38 F 0.6887 -1.7959 -4.6364
39 C -4.0937 -0.3718 -0.4200
40 H -1.8904 -0.0182 -0.7214
41 H 5.0139 2.2893 -0.1193
42 H 5.4318 -0.2896 0.7805
43 H 0.5538 -3.3683 3.9666
44 H 3.0973 -3.2834 2.8824
45 H 2.8864 -4.6262 -3.7701
46 H 4.3670 -4.1213 -1.4898
47 H 4.2524 -2.6127 0.6520
48 H -0.9371 3.6222 1.1333
49 H -2.2136 4.1849 0.0054
50 H -4.1702 -1.3797 -0.8130
51 H -4.3191 0.4746 -1.0586
52 H -3.9700 -0.2186 0.6450

MECP3$_{ON}$_triplet
E = -2275.47162341 Hartree
Atom X Y Z
1 C 1.0958 -0.0081 3.2628
2 N 0.7483 0.3282 2.0019
3 N -0.5956 0.2632 1.8548
4 C -1.0860 -0.1157 3.0430
5 C -0.0578 -0.3040 3.9737
6 Cu -1.3577 0.6966 -0.0104
7 O -3.4117 0.5576 0.1710
8 C -3.4637 0.6676 -1.0999
9 O -4.5636 0.4468 -1.8211
10 C -5.7408 0.0176 -1.1311
11 C -5.9736 -1.4950 -1.3086
12 Cl -6.0952 -1.9151 -3.0485
13 B 1.6730 0.7506 0.8285
14 N 1.2627 2.1669 0.3606
15 N -0.0044 2.4134 -0.0351
16 C -0.0480 3.7050 -0.3695
17 C 1.2040 4.3164 -0.1967
18 C 2.0111 3.2922 0.2726
19 C -1.3094 4.3163 -0.8821
20 H 1.4677 5.3474 -0.3759
21 H 3.0546 3.2839 0.5517
22 C -2.5533 -0.2950 3.2615
23 H -0.1509 -0.6109 5.0036
24 H 2.1331 -0.0121 3.5629
25 N 0.2510 -0.4529 -0.8677
26 N 1.4769 -0.2463 -0.3391
27 C 2.4060 -0.9654 -1.0110
28 C 1.7670 -1.6697 -2.0191
29 C 0.4182 -1.3090 -1.8803
30 C -0.7357 -1.7358 -2.7265
31 H 2.1989 -2.3471 -2.7395
32 H 3.4465 -0.9273 -0.7232
33 N -2.3270 1.0345 -1.6726
34 Cl -4.6604 -2.4409 -0.5545
35 Cl -7.5351 -1.8726 -0.4860
36 H 2.8157 0.7475 1.1866
37 H -5.6803 0.2388 -0.0644
38 H -6.5772 0.5523 -1.5842
39 F -0.4693 -2.9229 -3.3416
40 F -1.8746 -1.9046 -1.9991
41 F -1.0283 -0.8454 -3.7136
42 F -2.7984 -0.6544 4.5526
43 F -3.2624 0.8408 3.0228
44 F -3.0852 -1.2668 2.4697
45 F -1.3285 5.6588 -0.6404
46 F -1.4658 4.1658 -2.2267
47 F -2.4171 3.7830 -0.2963

MECP3$_{ON}$_singlet
E = -2275.47164293 Hartree
Atom X Y Z
1 C 1.0958 -0.0081 3.2628
2 N 0.7483 0.3282 2.0019
3 N -0.5956 0.2632 1.8548
4 C -1.0860 -0.1157 3.0430
5 C -0.0578 -0.3040 3.9737
6 Cu -1.3577 0.6966 -0.0104
7 O -3.4117 0.5576 0.1710
8 C -3.4637 0.6676 -1.0999
9 O -4.5636 0.4468 -1.8211
10 C -5.7408 0.0176 -1.1311
11 C -5.9736 -1.4950 -1.3086
12 Cl -6.0952 -1.9151 -3.0485
13 B 1.6730 0.7506 0.8285
14 N 1.2627 2.1669 0.3606
15 N -0.0044 2.4134 -0.0351
16 C -0.0480 3.7050 -0.3695
17 C 1.2040 4.3164 -0.1967
18 C 2.0111 3.2922 0.2726
19 C -1.3094 4.3163 -0.8821
20 H 1.4677 5.3474 -0.3759
21 H 3.0546 3.2839 0.5517
22 C -2.5533 -0.2950 3.2615
23 H -0.1509 -0.6109 5.0036
24 H 2.1331 -0.0121 3.5629
25 N 0.2510 -0.4529 -0.8677
26 N 1.4769 -0.2463 -0.3391
27 C 2.4060 -0.9654 -1.0110
28 C 1.7670 -1.6697 -2.0191

29 C 0.4182 -1.3090 -1.8803
30 C -0.7357 -1.7358 -2.7265
31 H 2.1989 -2.3471 -2.7395
32 H 3.4465 -0.9273 -0.7232
33 N -2.3270 1.0345 -1.6726
34 Cl -4.6604 -2.4409 -0.5545
35 Cl -7.5351 -1.8726 -0.4860
36 H 2.8157 0.7475 1.1866
37 H -5.6803 0.2388 -0.0644
38 H -6.5772 0.5523 -1.5842
39 F -0.4693 -2.9229 -3.3416
40 F -1.8746 -1.9046 -1.9991
41 F -1.0283 -0.8454 -3.7136
42 F -2.7984 -0.6544 4.5526
43 F -3.2624 0.8408 3.0228
44 F -3.0852 -1.2668 2.4697
45 F -1.3285 5.6588 -0.6404
46 F -1.4658 4.1658 -2.2267
47 F -2.4171 3.7830 -0.2963

MECP3$_N$_singlet
E = -2275.46631366 Hartree
Atom X Y Z
1 C -0.9568 -1.9581 -2.2824
2 N -0.8889 -1.1392 -1.2090
3 N 0.1618 -1.4854 -0.4329
4 C 0.7507 -2.5321 -1.0221
5 C 0.0834 -2.8725 -2.2056
6 Cu 0.4910 -0.3474 1.2328
7 N 2.2502 -0.2739 1.4877
8 C 3.1806 0.3449 0.8314
9 O 4.2765 0.7967 1.4613
10 C 5.3103 1.3093 0.6239
11 C 6.5295 0.3709 0.6621
12 Cl 7.1742 0.2443 2.3337
13 B -1.8506 0.0101 -0.8010
14 N -1.1062 1.3588 -0.8119
15 N -0.0741 1.5702 0.0327
16 C 0.3059 2.8339 -0.1652
17 C -0.4779 3.4593 -1.1520
18 C -1.3699 2.4744 -1.5380
19 C 1.4352 3.4298 0.6055
20 H -0.4058 4.4746 -1.5116
21 H -2.1702 2.4849 -2.2638
22 C 1.9303 -3.2144 -0.4055
23 H 0.3197 -3.6780 -2.8833
24 H -1.7413 -1.8372 -3.0150
25 N -1.4604 -0.4227 1.6662
26 N -2.3400 -0.2726 0.6514
27 C -3.6021 -0.3898 1.1181
28 C -3.5509 -0.6284 2.4853
29 C -2.1811 -0.6410 2.7720
30 C -1.4828 -0.8424 4.0752
31 H -4.3712 -0.7771 3.1704
32 H -4.4444 -0.2988 0.4481
33 O 2.9092 0.4473 -0.3843
34 Cl 6.0814 -1.2559 0.0683
35 Cl 7.7885 1.0902 -0.4104
36 H -2.7854 0.0384 -1.5489
37 H 4.9731 1.4077 -0.4098
38 H 5.5986 2.2865 1.0147
39 F -2.3549 -1.1958 5.0515
40 F -0.5309 -1.8178 3.9999
41 F -0.8378 0.2844 4.4937
42 F 1.1889 4.7393 0.9016
43 F 1.6619 2.7847 1.7793
44 F 2.6205 3.4193 -0.0775
45 F 2.0994 -4.4473 -0.9623
46 F 3.0980 -2.5440 -0.5804
47 F 1.7730 -3.3915 0.9346

MECP3$_N$_triplet
E = -2275.46631495 Hartree
Atom X Y Z
1 C -0.9568 -1.9581 -2.2824
2 N -0.8889 -1.1392 -1.2090
3 N 0.1618 -1.4854 -0.4329
4 C 0.7507 -2.5321 -1.0221
5 C 0.0834 -2.8725 -2.2056
6 Cu 0.4910 -0.3474 1.2328
7 N 2.2502 -0.2739 1.4877
8 C 3.1806 0.3449 0.8314
9 O 4.2765 0.7967 1.4613
10 C 5.3103 1.3093 0.6239
11 C 6.5295 0.3709 0.6621
12 Cl 7.1742 0.2443 2.3337
13 B -1.8506 0.0101 -0.8010
14 N -1.1062 1.3588 -0.8119
15 N -0.0741 1.5702 0.0327
16 C 0.3059 2.8339 -0.1652
17 C -0.4779 3.4593 -1.1520
18 C -1.3699 2.4744 -1.5380
19 C 1.4352 3.4298 0.6055
20 H -0.4058 4.4746 -1.5116
21 H -2.1702 2.4849 -2.2638
22 C 1.9303 -3.2144 -0.4055
23 H 0.3197 -3.6780 -2.8833
24 H -1.7413 -1.8372 -3.0150
25 N -1.4604 -0.4227 1.6662
26 N -2.3400 -0.2726 0.6514
27 C -3.6021 -0.3898 1.1181
28 C -3.5509 -0.6284 2.4853
29 C -2.1811 -0.6410 2.7720
30 C -1.4828 -0.8424 4.0752
31 H -4.3712 -0.7771 3.1704
32 H -4.4444 -0.2988 0.4481
33 O 2.9092 0.4473 -0.3843
34 Cl 6.0814 -1.2559 0.0683
35 Cl 7.7885 1.0902 -0.4104
36 H -2.7854 0.0384 -1.5489
37 H 4.9731 1.4077 -0.4098
38 H 5.5986 2.2865 1.0147
39 F -2.3549 -1.1958 5.0515
40 F -0.5309 -1.8178 3.9999
41 F -0.8378 0.2844 4.4937
42 F 1.1889 4.7393 0.9016
43 F 1.6619 2.7847 1.7793
44 F 2.6205 3.4193 -0.0775
45 F 2.0994 -4.4473 -0.9623
46 F 3.0980 -2.5440 -0.5804
47 F 1.7730 -3.3915 0.9346

Methane Amidation---

CH_4
E = -40.5175777784 Hartree
Atom X Y Z
1 C -3.6444 -2.1043 -3.1065
2 H -2.9657 -2.8694 -3.4960
3 H -3.2304 -1.6851 -2.1841
4 H -3.7614 -1.3085 -3.8485
5 H -4.6197 -2.5544 -2.8972

Int4$_{ON}$_triplet
E = -2316.00632562 Hartree
Atom X Y Z
1 C 1.1196 0.2786 3.1280
2 N 0.7355 0.2293 1.8334
3 N -0.4861 -0.3413 1.7350
4 C -0.8627 -0.6569 2.9799
5 C 0.1181 -0.2824 3.9069
6 Cu -1.3139 -0.5766 -0.1131
7 O -3.3132 -0.0938 0.2783
8 C -3.5336 -0.4424 -0.9271
9 O -4.7069 -0.2000 -1.5461
10 C -5.7248 0.4497 -0.7954
11 C -6.8486 -0.5404 -0.4429
12 Cl -7.5738 -1.2324 -1.9367
13 B 1.4932 0.7266 0.5719
14 N 0.6809 1.8607 -0.0857
15 N -0.5910 1.6490 -0.4887
16 C -1.0267 2.8283 -0.9415
17 C -0.0371 3.8209 -0.8384
18 C 1.0412 3.1532 -0.2826
19 C -2.4211 2.9862 -1.4455
20 H -0.1068 4.8576 -1.1300
21 H 2.0294 3.4984 -0.0151
22 C -2.1808 -1.3100 3.2483
23 H 0.0972 -0.4154 4.9774
24 H 2.0747 0.7032 3.4001
25 N 0.5491 -1.1954 -0.7841
26 N 1.6270 -0.4714 -0.4078
27 C 2.7471 -0.9871 -0.9607
28 C 2.3941 -2.0909 -1.7236
29 C 1.0045 -2.1774 -1.5715
30 C 0.0763 -3.1930 -2.1547
31 H 3.0363 -2.7452 -2.2926
32 H 3.7106 -0.5389 -0.7668
33 N -2.5487 -1.0319 -1.5643
34 Cl -6.2272 -1.8675 0.5846
35 Cl -8.1097 0.3852 0.4539
36 C -0.5004 0.3872 -3.8852
37 H 2.5816 1.1211 0.8784
38 H -5.3251 0.8720 0.1276
39 H -6.1446 1.2377 -1.4240
40 H -0.2730 0.4189 -2.8281
41 H -2.5526 -1.0769 -2.5768
42 H -1.1928 1.1054 -4.3077
43 H -0.0191 -0.3421 -4.5258
44 F -2.1567 -1.9449 4.4545
45 F -3.2123 -0.4264 3.2942
46 F -2.4930 -2.2404 2.3055
47 F 0.7819 -4.2337 -2.6772
48 F -0.7789 -3.7031 -1.2300
49 F -0.6917 -2.6957 -3.1665
50 F -2.5758 4.1854 -2.0727
51 F -2.7758 2.0185 -2.3394
52 F -3.3600 2.9408 -0.4541

Int4$_{ON}$_oss
E = -2316.0063497 Hartree
Atom X Y Z
1 C 1.1146 0.2710 3.1306
2 N 0.7329 0.2238 1.8353
3 N -0.4888 -0.3463 1.7338
4 C -0.8677 -0.6638 2.9775
5 C 0.1115 -0.2910 3.9069
6 Cu -1.3141 -0.5771 -0.1160
7 O -3.3123 -0.0923 0.2748
8 C -3.5331 -0.4408 -0.9306
9 O -4.7062 -0.1970 -1.5495
10 C -5.7225 0.4558 -0.7994
11 C -6.8484 -0.5313 -0.4451
12 Cl -7.5754 -1.2241 -1.9376
13 B 1.4925 0.7239 0.5760
14 N 0.6814 1.8596 -0.0800
15 N -0.5895 1.6486 -0.4863
16 C -1.0247 2.8289 -0.9369
17 C -0.0357 3.8216 -0.8289
18 C 1.0417 3.1528 -0.2726
19 C -2.4182 2.9878 -1.4431
20 H -0.1051 4.8590 -1.1180

21 H 2.0291 3.4975 -0.0021
22 C -2.1864 -1.3170 3.2424
23 H 0.0888 -0.4258 4.9772
24 H 2.0694 0.6947 3.4052
25 N 0.5496 -1.1946 -0.7861
26 N 1.6272 -0.4720 -0.4063
27 C 2.7483 -0.9882 -0.9566
28 C 2.3962 -2.0912 -1.7214
29 C 1.0061 -2.1764 -1.5729
30 C 0.0785 -3.1913 -2.1585
31 H 3.0392 -2.7454 -2.2895
32 H 3.7117 -0.5412 -0.7599
33 N -2.5490 -1.0314 -1.5678
34 Cl -6.2295 -1.8582 0.5843
35 Cl -8.1073 0.3983 0.4506
36 C -0.4972 0.3902 -3.8879
37 H 2.5805 1.1172 0.8850
38 H -5.3216 0.8786 0.1230
39 H -6.1408 1.2438 -1.4290
40 H -0.2737 0.4189 -2.8299
41 H -2.5540 -1.0777 -2.5801
42 H -1.1746 1.1203 -4.3143
43 H -0.0265 -0.3486 -4.5255
44 F -2.1643 -1.9548 4.4472
45 F -3.2177 -0.4332 3.2891
46 F -2.4976 -2.2450 2.2970
47 F 0.7847 -4.2306 -2.6827
48 F -0.7767 -3.7035 -1.2349
49 F -0.6896 -2.6924 -3.1694
50 F -2.5712 4.1872 -2.0702
51 F -2.7718 2.0205 -2.3380
52 F -3.3588 2.9426 -0.4533

TS3-4$_{ON}$_triplet
E = -2315.98593100 Hartree
Atom X Y Z
1 C 1.2007 0.2534 3.2348
2 N 0.8396 0.2326 1.9326
3 N -0.3608 -0.3717 1.7935
4 C -0.7482 -0.7371 3.0211
5 C 0.2039 -0.3618 3.9780
6 Cu -1.1521 -0.5757 -0.0903
7 O -3.2278 -0.1503 0.2629
8 C -3.3835 -0.5229 -0.9357
9 O -4.4920 -0.2521 -1.6485
10 C -5.4897 0.5613 -1.0392
11 C -6.7295 -0.2782 -0.6886
12 Cl -7.4208 -1.0323 -2.1673
13 B 1.6017 0.7861 0.6985
14 N 0.7626 1.9097 0.0558
15 N -0.4990 1.6792 -0.3693
16 C -0.9574 2.8631 -0.7877
17 C 0.0051 3.8761 -0.6403
18 C 1.0926 3.2162 -0.0942
19 C -2.3565 3.0131 -1.2741
20 H -0.0878 4.9202 -0.8967
21 H 2.0686 3.5759 0.1972
22 C -2.0521 -1.4333 3.2418
23 H 0.1688 -0.5284 5.0435
24 H 2.1378 0.6974 3.5368
25 N 0.7631 -1.1157 -0.7568
26 N 1.8035 -0.3769 -0.3069
27 C 2.9670 -0.8639 -0.7932
28 C 2.6848 -1.9643 -1.5879
29 C 1.2899 -2.0786 -1.5235
30 C 0.4337 -3.1201 -2.1669
31 H 3.3730 -2.6004 -2.1225
32 H 3.9069 -0.3993 -0.5339
33 N -2.3836 -1.2077 -1.5025
34 Cl -6.3186 -1.5562 0.4938
35 Cl -7.9429 0.8477 0.0272
36 C -2.0506 -0.3448 -3.9094
37 H 2.6678 1.2136 1.0382
38 H -5.1138 1.0333 -0.1308
39 H -5.7805 1.3194 -1.7690
40 H -1.0858 0.1565 -3.8294
41 H -2.2313 -0.8825 -2.7314
42 H -2.8920 0.3314 -4.0507
43 H -2.0497 -1.2091 -4.5735
44 F -2.0339 -2.1145 4.4223
45 F -3.1071 -0.5768 3.3030
46 F -2.3268 -2.3318 2.2576
47 F -2.5244 4.1970 -1.9240
48 F -2.7247 2.0240 -2.1404
49 F -3.2817 2.9879 -0.2678
50 F 1.2083 -4.1354 -2.6450
51 F -0.4591 -3.6649 -1.3017
52 F -0.2784 -2.6438 -3.2262

TS3-5$_{ON}$_singlet
E = -2315.98196011 Hartree
Atom X Y Z
1 C 1.3614 0.0838 3.3854
2 N 0.9495 0.2142 2.1023
3 N -0.3386 -0.1772 1.9799
4 C -0.7288 -0.5573 3.2005
5 C 0.3068 -0.4122 4.1357
6 Cu -1.1980 -0.0451 0.0600
7 O -3.3512 -0.1602 0.2584
8 C -3.4544 0.0434 -0.9723
9 O -4.6355 0.2640 -1.5947
10 C -5.8151 0.0839 -0.8206
11 C -6.5557 -1.1835 -1.2807
12 Cl -7.0196 -1.0453 -3.0157
13 B 1.7611 0.7094 0.8793
14 N 1.0896 1.9756 0.2899
15 N -0.1902 1.9448 -0.1350
16 C -0.4462 3.1635 -0.6172
17 C 0.6737 4.0059 -0.5107
18 C 1.6341 3.1992 0.0777
19 C -1.7715 3.4724 -1.2204
20 H 0.7576 5.0396 -0.8098
21 H 2.6557 3.4050 0.3622
22 C -2.1197 -1.0463 3.4291
23 H 0.2826 -0.6414 5.1898
24 H 2.3699 0.3482 3.6682
25 N 0.6366 -0.8928 -0.7201
26 N 1.7830 -0.4050 -0.1999
27 C 2.8548 -1.0157 -0.7607
28 C 2.3958 -1.9411 -1.6844
29 C 0.9984 -1.8188 -1.6118
30 C -0.0308 -2.5474 -2.4059
31 H 2.9711 -2.6086 -2.3075
32 H 3.8584 -0.7518 -0.4605
33 N -2.3095 0.0630 -1.6599
34 Cl -5.5225 -2.6292 -1.0586
35 Cl -8.0423 -1.3319 -0.2793
36 C -2.9432 0.0495 -3.8850
37 H 2.8831 0.9668 1.2157
38 H -5.5773 -0.0089 0.2411
39 H -6.4618 0.9467 -0.9920
40 H -3.3767 -0.9337 -3.7485
41 H -3.6427 0.8670 -4.0428
42 H -2.3501 0.5409 -2.6610
43 H -2.0032 0.0794 -4.4320
44 F -2.2430 -1.5710 4.6825
45 F -3.0528 -0.0580 3.3254
46 F -2.4830 -2.0181 2.5487
47 F -1.9765 4.8159 -1.2955
48 F -1.8991 2.9969 -2.5037
49 F -2.8130 2.9409 -0.5222
50 F 0.4909 -3.6845 -2.9481
51 F -1.1132 -2.9096 -1.6685
52 F -0.5103 -1.8110 -3.4580

Int5$_{N}$_singlet
E = -2316.11924494 Hartree
Atom X Y Z
1 C -1.4452 -1.2561 -2.5059
2 N -1.1075 -0.6379 -1.3483
3 N 0.2292 -0.4430 -1.2862
4 C 0.7226 -0.9452 -2.4230
5 C -0.2899 -1.4786 -3.2361
6 Cu 0.9080 0.6358 0.4196
7 N 2.7303 0.8649 1.4933
8 C 2.6714 2.1279 2.2672
9 B -2.0576 -0.1417 -0.2278
10 N -1.9336 1.3975 -0.1003
11 N -0.7527 1.9926 0.1811
12 C -0.9968 3.3072 0.1690
13 C -2.3437 3.5818 -0.1151
14 C -2.9037 2.3261 -0.2822
15 C 0.0919 4.2739 0.4751
16 H -2.8219 4.5462 -0.1920
17 H -3.9140 2.0270 -0.5205
18 C 2.1878 -0.9520 -2.6743
19 H -0.1868 -1.9446 -4.2041
20 H -2.4775 -1.4862 -2.7253
21 N -0.4617 -0.6245 1.6646
22 N -1.6794 -0.8097 1.1158
23 C -2.4392 -1.6180 1.8960
24 C -1.6917 -1.9806 3.0051
25 C -0.4653 -1.3275 2.8008
26 C 0.7435 -1.3218 3.6633
27 H -1.9789 -2.6135 3.8306
28 H -3.4482 -1.8737 1.6072
29 C 3.8336 0.7539 0.6258
30 O 4.0864 -0.5678 0.4148
31 C 5.2140 -0.8956 -0.3817
32 C 6.3086 -1.5230 0.4973
33 Cl 6.8682 -0.3546 1.7426
34 O 4.4381 1.6781 0.1411
35 Cl 5.6949 -3.0018 1.3111
36 Cl 7.6804 -1.9571 -0.5827
37 H -3.1873 -0.4274 -0.5118
38 H 4.8972 -1.6330 -1.1190
39 H 5.6156 -0.0081 -0.8745
40 H 2.6905 2.9628 1.5713
41 H 2.6814 0.0418 2.0937
42 H 3.5183 2.2054 2.9581
43 H 1.7347 2.1368 2.8232
44 F -0.2382 5.5317 0.0779
45 F 0.3729 4.3507 1.8141
46 F 1.2684 3.9488 -0.1350
47 F 0.5983 -2.1218 4.7457
48 F 1.8699 -1.7443 2.9962
49 F 1.0615 -0.0696 4.1256
50 F 2.4640 -1.0032 -4.0054
51 F 2.8188 0.1458 -2.1698
52 F 2.8152 -2.0368 -2.1138

product_methane_amination
E = -406.138950282 Hartree
Atom X Y Z
1 C -3.2254 -0.2241 0.3393
2 N -3.6053 0.0393 -1.0423
3 C -4.8536 0.3282 -1.4730
4 O -5.7476 0.2124 -0.4259
5 C -7.0719 0.6277 -0.6797
6 C -7.3794 1.9322 0.0783
7 Cl -9.0908 2.3729 -0.2682
8 O -5.1685 0.6233 -2.6114
9 Cl -7.1756 1.6990 1.8515

10 Cl -6.2937 3.2516 -0.4688
11 H -7.2309 0.7981 -1.7470
12 H -7.7528 -0.1438 -0.3115
13 H -3.4577 0.6273 0.9882
14 H -3.7403 -1.1082 0.7280
15 H -2.8908 0.1157 -1.7513
16 H -2.1487 -0.4057 0.3664

Int5$_O$_singlet
E = -2316.12064088 Hartree
Atom X Y Z
1 C -0.8758 -1.2928 2.7840
2 N -0.7716 -0.6262 1.6315
3 N -2.0031 -0.6395 1.0796
4 C -2.8711 -1.3100 1.8765
5 C -2.1851 -1.7532 2.9969
6 Cu 0.7348 0.3363 0.4808
7 N -0.6426 1.9562 0.2068
8 N -1.9034 1.5859 -0.1068
9 C -2.7091 2.6731 -0.1932
10 C -1.9511 3.8016 0.0742
11 C -0.6673 3.2866 0.3205
12 B -2.2832 0.0884 -0.2652
13 N -1.4329 -0.5456 -1.3928
14 C -1.8469 -1.0899 -2.5644
15 C -0.7285 -1.4966 -3.2744
16 C 0.3424 -1.1482 -2.4336
17 N -0.0865 -0.5784 -1.3058
18 C 1.8038 -1.3367 -2.6525
19 F 2.3875 -2.1247 -1.7025
20 C 0.5846 4.0277 0.6348
21 F 1.3806 4.2226 -0.4620
22 C 0.3235 -1.4241 3.6516
23 F 0.7987 -0.1983 4.0737
24 N 3.2414 1.0399 2.6381
25 C 3.5354 0.6752 1.3883
26 O 2.7230 0.2648 0.5495
27 C 4.1993 1.5494 3.6120
28 F 0.3140 5.2645 1.1357
29 F 1.3624 3.3805 1.5521
30 F 2.0447 -1.9251 -3.8577
31 F 2.5008 -0.1608 -2.6398
32 F 0.0563 -2.1408 4.7721
33 F 1.3789 -2.0191 3.0278
34 O 4.8600 0.7731 1.1121
35 C 5.2537 0.5847 -0.2423
36 C 5.5939 1.9374 -0.8929
37 Cl 6.1124 1.5974 -2.5821
38 Cl 4.1675 3.0131 -0.8947
39 Cl 6.9441 2.7392 -0.0068
40 H -2.2686 4.8329 0.0951
41 H -3.7568 2.5641 -0.4328
42 H -0.6851 -1.9696 -4.2435
43 H -2.8986 -1.1468 -2.8048
44 H -2.5636 -2.3173 3.8355
45 H -3.9072 -1.4205 1.5913
46 H -3.4484 -0.0048 -0.5353
47 H 6.1530 -0.0338 -0.2373
48 H 4.4607 0.1061 -0.8198
49 H 4.9431 0.7893 3.8718
50 H 2.2712 0.9296 2.9029
51 H 3.6462 1.8270 4.5109
52 H 4.7144 2.4323 3.2227

MECP4-5_singlet
E = -2315.99477200 Hartree
Atom X Y Z
1 N -2.6865 2.5421 -1.5234
2 N -2.5933 1.3660 -0.8644
3 C -3.7877 1.0384 -0.3213
4 C -4.7011 2.0343 -0.6337
5 C -3.9543 2.9483 -1.3888
6 B -1.2815 0.5398 -0.8877
7 N -0.1258 1.4253 -0.3596
8 N 0.1780 2.6075 -0.9414
9 C 1.2065 3.1128 -0.2482
10 C 1.5826 2.2566 0.7940
11 C 0.7025 1.1907 0.6821
12 Cu -0.9801 3.2698 -2.5133
13 N -1.1192 4.5097 -3.9205
14 C -1.2148 5.8572 -3.7058
15 O -1.4279 6.5368 -4.8893
16 C -1.6079 7.9369 -4.8040
17 C -3.0580 8.3125 -5.1598
18 Cl -4.2035 7.5640 -4.0097
19 C 1.7670 4.4553 -0.5812
20 F 1.9591 4.6184 -1.9210
21 C -4.4105 4.2235 -2.0154
22 F -3.7192 5.3040 -1.5660
23 N -0.7927 1.0555 -3.3010
24 C -0.6131 0.3747 -4.4365
25 C -0.6802 -1.0133 -4.2282
26 C -0.9196 -1.1269 -2.8689
27 N -0.9810 0.1218 -2.3439
28 C -0.3318 1.0950 -5.7085
29 F -1.1229 2.1974 -5.8763
30 F -4.2723 4.2130 -3.3734
31 F -5.7268 4.4466 -1.7512
32 F 0.9613 5.4744 -0.1787
33 F 2.9715 4.6371 0.0266
34 F 0.9573 1.5447 -5.7933
35 F -0.5325 0.2893 -6.7850
36 O -1.0872 6.4082 -2.6280
37 Cl -3.1858 10.1100 -5.0673
38 Cl -3.4591 7.7774 -6.8319
39 C 1.3812 4.5585 -4.9565
40 H 1.5148 5.6006 -4.6947
41 H 2.3752 2.4000 1.5120
42 H 0.6070 0.2855 1.2638
43 H -5.7434 2.0980 -0.3618
44 H -3.9005 0.1248 0.2442
45 H -0.5749 -1.7998 -4.9596
46 H -1.0534 -1.9969 -2.2424
47 H -1.3991 -0.4305 -0.1947
48 H -1.3918 8.2970 -3.7955
49 H -0.9457 8.4161 -5.5300
50 H 1.7494 3.7920 -4.2889
51 H -1.3543 4.2470 -4.8752
52 H 1.0370 4.2913 -5.9480

TS3-4$_N$_triplet
E = -2315.98465349 Hartree
Atom X Y Z
1 C 2.6852 -33.1817 2.5470
2 N 2.9654 -32.5354 3.6836
3 N 4.1033 -33.0818 4.1632
4 C 4.5359 -34.0589 3.3348
5 C 3.6493 -34.1603 2.2716
6 Cu 2.0182 -31.0768 4.8230
7 N 4.1363 -30.1789 4.9396
8 N 5.0841 -31.0701 5.3093
9 C 6.3003 -30.4742 5.3838
10 C 6.1489 -29.1410 5.0473
11 C 4.7746 -29.0182 4.7753
12 B 4.7347 -32.5612 5.4847
13 N 3.6888 -32.7288 6.6198
14 N 2.4518 -32.1976 6.5135
15 C 1.8009 -32.5244 7.6359
16 C 2.6187 -33.2737 8.4912
17 C 3.8163 -33.3786 7.7984
18 C 0.3835 -32.1058 7.8366
19 F -0.1432 -32.6961 8.9423
20 C 4.0580 -27.7844 4.3364
21 F 3.8068 -26.9179 5.3566
22 C 1.4806 -32.7953 1.7585
23 F 1.2408 -33.6823 0.7591
24 N 0.6009 -29.8824 4.8570
25 C 0.5017 -28.6897 5.5245
26 O -0.6489 -28.0207 5.1847
27 C -0.9184 -26.8065 5.8604
28 C -2.1335 -26.9781 6.7891
29 Cl -3.5889 -27.4537 5.8429
30 O 1.3385 -28.2549 6.2958
31 Cl -1.8036 -28.2254 8.0302
32 Cl -2.4360 -25.3894 7.5840
33 F 0.2405 -30.7614 7.9950
34 F -0.4080 -32.4506 6.7776
35 F 4.8143 -27.0864 3.4331
36 F 2.8701 -28.0532 3.7353
37 F 0.3552 -32.7385 2.5339
38 F 1.6014 -31.5643 1.1839
39 C -1.7632 -30.8758 4.4702
40 H -0.5492 -30.3562 4.6401
41 H 6.9061 -28.3736 4.9921
42 H 7.1771 -31.0418 5.6604
43 H 2.3659 -33.6782 9.4590
44 H 4.7431 -33.8695 8.0565
45 H 3.6856 -34.8350 1.4302
46 H 5.4376 -34.6095 3.5598
47 H 5.7181 -33.1933 5.7463
48 H -0.0610 -26.4888 6.4577
49 H -1.1646 -26.0469 5.1144
50 H -1.5489 -31.8983 4.1601
51 H -2.1935 -30.2439 3.6934
52 H -2.2410 -30.7852 5.4442

Ethane amidation--

CH_3CH_3
E = -79.8328451458 Hartree
Atom X Y Z
1 C -3.6693 -2.0762 -3.0921
2 C -2.7211 -3.1453 -3.6365
3 H -3.2833 -1.6344 -2.1653
4 H -3.8089 -1.2615 -3.8132
5 H -4.6588 -2.4950 -2.8713
6 H -3.1071 -3.5871 -4.5633
7 H -1.7316 -2.7265 -3.8573
8 H -2.5815 -3.9599 -2.9154

eTS3-4$_{ON}$_triplet
E = -2355.31029073 Hartree
Atom X Y Z
1 C 1.2229 -2.1372 -1.5127
2 N 0.7164 -1.1539 -0.7590
3 N 1.7627 -0.3869 -0.3776
4 C 2.9117 -0.8735 -0.8987
5 C 2.6121 -2.0038 -1.6437
6 Cu -1.1803 -0.5891 -0.0804
7 N -2.3860 -1.2254 -1.5111
8 C -3.3974 -0.5451 -0.9598
9 O -4.4963 -0.2833 -1.6925
10 C -5.5089 0.5247 -1.1018
11 C -6.7487 -0.3219 -0.7686
12 Cl -7.9799 0.7973 -0.0724
13 B 1.5899 0.7775 0.6324
14 N 0.7318 1.9068 0.0246
15 N -0.5467 1.6837 -0.3509
16 C -1.0269 2.8783 -0.7130
17 C -0.0606 3.8899 -0.5800
18 C 1.0514 3.2186 -0.1014

19 C -2.4441 3.0365 -1.1425
20 F -3.3317 2.9764 -0.1062
21 C 0.3557 -3.2169 -2.0760
22 F -0.3697 -2.8150 -3.1569
23 N -0.3530 -0.3571 1.7887
24 N 0.8625 0.2253 1.8888
25 C 1.2622 0.2454 3.1800
26 C 0.2767 -0.3476 3.9555
27 C -0.7102 -0.7087 3.0289
28 C -2.0243 -1.3711 3.2879
29 F -2.3325 -2.2907 2.3331
30 O -3.2654 -0.1668 0.2402
31 Cl -7.4133 -1.0819 -2.2566
32 Cl -6.3488 -1.5963 0.4214
33 F -2.6387 4.2387 -1.7505
34 F -2.8397 2.0732 -2.0275
35 F -2.0042 -2.0183 4.4870
36 F -3.0598 -0.4899 3.3370
37 F 1.1244 -4.2630 -2.4952
38 F -0.5246 -3.7009 -1.1639
39 C -1.9369 -0.2547 -3.8759
40 H -0.1671 4.9405 -0.8025
41 H 2.0381 3.5737 0.1583
42 H 0.2692 -0.5075 5.0227
43 H 2.2153 0.6746 3.4522
44 H 3.2870 -2.6490 -2.1845
45 H 3.8554 -0.3901 -0.6926
46 H 2.6664 1.2034 0.9402
47 H -5.1512 1.0021 -0.1886
48 H -5.7938 1.2787 -1.8381
49 C -0.5631 0.3651 -3.8073
50 H -2.1699 -0.8311 -2.7530
51 H -2.7611 0.4563 -3.9593
52 H -2.0408 -1.0895 -4.5737
53 H -0.3551 0.9380 -4.7241
54 H -0.4789 1.0542 -2.9612
55 H 0.2155 -0.3967 -3.7087

eMECP4-5_singlet
E = -2355.32276979 Hartree
Atom X Y Z
1 C -2.7049 2.5208 -1.5272
2 N -2.6127 1.3585 -0.8759
3 N -3.8420 1.1299 -0.3656
4 C -4.6949 2.1319 -0.6939
5 C -4.0010 3.0593 -1.4528
6 Cu -1.0174 -0.2750 -0.2377
7 N -2.4951 -1.6242 -0.7081
8 N -3.7514 -1.3894 -0.2653
9 C -4.5726 -2.3971 -0.6332
10 C -3.8327 -3.3319 -1.3418
11 C -2.5395 -2.7950 -1.3569
12 B -4.1057 -0.1043 0.5239
13 N -3.1931 -0.0242 1.7755
14 C -3.5394 0.1404 3.0719
15 C -2.3802 0.2299 3.8298
16 C -1.3524 0.1081 2.8858
17 N -1.8483 -0.0443 1.6526
18 C 0.1243 0.1464 3.0993
19 F 0.7141 1.1797 2.4307
20 C -1.3051 -3.3969 -1.9413
21 F -0.4761 -3.9172 -0.9972
22 C -1.5294 3.0634 -2.2619
23 F -1.3247 2.4445 -3.4642
24 N 0.7418 0.2404 -0.6249
25 C 1.8354 -0.5402 -0.3913
26 O 1.8125 -1.7159 -0.0703
27 O 3.0050 0.1640 -0.6134
28 C 4.2202 -0.5156 -0.3735
29 C 4.9618 0.1160 0.8190
30 Cl 6.4993 -0.8013 1.0430
31 Cl 5.3520 1.8414 0.4821
32 Cl 3.9735 0.0125 2.3059
33 F -1.6225 -4.4094 -2.7941
34 F -0.5710 -2.4891 -2.6458
35 F 0.4186 0.3032 4.4186
36 F 0.7433 -0.9884 2.6778
37 F -1.6816 4.3879 -2.5288
38 F -0.3620 2.9229 -1.5650
39 C 0.9030 0.1576 -3.5370
40 H -4.1648 -4.2587 -1.7832
41 H -5.6194 -2.3733 -0.3677
42 H -2.2840 0.3590 4.8970
43 H -4.5796 0.1852 3.3600
44 H -4.3632 3.9806 -1.8830
45 H -5.7225 2.1131 -0.3611
46 H -5.2555 -0.1385 0.8589
47 H 4.0388 -1.5699 -0.1525
48 H 4.8549 -0.4154 -1.2580
49 H -0.1319 -0.1584 -3.4721
50 H 0.9891 1.1988 -0.8643
51 C 1.9887 -0.8469 -3.6585
52 H 1.0975 1.2196 -3.6434
53 H 2.0098 -1.2985 -4.6676
54 H 2.9768 -0.4067 -3.4840
55 H 1.8442 -1.6825 -2.9603

eMECP4-5_triplet
E = -2355.32274189 Hartree
Atom X Y Z
1 C -2.7049 2.5208 -1.5272
2 N -2.6127 1.3585 -0.8759
3 N -3.8420 1.1299 -0.3656
4 C -4.6949 2.1319 -0.6939
5 C -4.0010 3.0593 -1.4528
6 Cu -1.0174 -0.2750 -0.2377
7 N -2.4951 -1.6242 -0.7081
8 N -3.7514 -1.3894 -0.2653
9 C -4.5726 -2.3971 -0.6332
10 C -3.8327 -3.3319 -1.3418
11 C -2.5395 -2.7950 -1.3569
12 B -4.1057 -0.1043 0.5239
13 N -3.1931 -0.0242 1.7755
14 C -3.5394 0.1404 3.0719
15 C -2.3802 0.2299 3.8298
16 C -1.3524 0.1081 2.8858
17 N -1.8483 -0.0443 1.6526
18 C 0.1243 0.1464 3.0993
19 F 0.7141 1.1797 2.4307
20 C -1.3051 -3.3969 -1.9413
21 F -0.4761 -3.9172 -0.9972
22 C -1.5294 3.0634 -2.2619
23 F -1.3247 2.4445 -3.4642
24 N 0.7418 0.2404 -0.6249
25 C 1.8354 -0.5402 -0.3913
26 O 1.8125 -1.7159 -0.0703
27 O 3.0050 0.1640 -0.6134
28 C 4.2202 -0.5156 -0.3735
29 C 4.9618 0.1160 0.8190
30 Cl 6.4993 -0.8013 1.0430
31 Cl 5.3520 1.8414 0.4821
32 Cl 3.9735 0.0125 2.3059
33 F -1.6225 -4.4094 -2.7941
34 F -0.5710 -2.4891 -2.6458
35 F 0.4186 0.3032 4.4186
36 F 0.7433 -0.9884 2.6778
37 F -1.6816 4.3879 -2.5288
38 F -0.3620 2.9229 -1.5650
39 C 0.9030 0.1576 -3.5370
40 H -4.1648 -4.2587 -1.7832
41 H -5.6194 -2.3733 -0.3677
42 H -2.2840 0.3590 4.8970
43 H -4.5796 0.1852 3.3600
44 H -4.3632 3.9806 -1.8830
45 H -5.7225 2.1131 -0.3611
46 H -5.2555 -0.1385 0.8589
47 H 4.0388 -1.5699 -0.1525
48 H 4.8549 -0.4154 -1.2580
49 H -0.1319 -0.1584 -3.4721
50 H 0.9891 1.1988 -0.8643
51 C 1.9887 -0.8469 -3.6585
52 H 1.0975 1.2196 -3.6434
53 H 2.0098 -1.2985 -4.6676
54 H 2.9768 -0.4067 -3.4840
55 H 1.8442 -1.6825 -2.9603

eInt5$_O$_singlet
E = -2355.44245309 Hartree
Atom X Y Z
1 N -0.0760 -0.5831 -1.3140
2 N -1.4221 -0.5242 -1.3926
3 C -1.8533 -1.0529 -2.5651
4 C -0.7471 -1.4761 -3.2846
5 C 0.3352 -1.1532 -2.4482
6 B -2.2529 0.1202 -0.2568
7 N -1.8394 1.6084 -0.0902
8 N -0.5703 1.9485 0.2238
9 C -0.5671 3.2775 0.3545
10 C -1.8404 3.8224 0.1186
11 C -2.6225 2.7132 -0.1605
12 Cu 0.7736 0.2964 0.4791
13 O 2.7587 0.1847 0.5258
14 C 3.5930 0.5875 1.3467
15 O 4.9117 0.6699 1.0354
16 C 5.2704 0.4436 -0.3230
17 C 5.6301 1.7733 -1.0088
18 Cl 7.0215 2.5575 -0.1714
19 C 0.7000 3.9862 0.6811
20 F 1.4561 3.3117 1.5988
21 C 1.7911 -1.3714 -2.6753
22 F 2.5179 -0.2153 -2.6194
23 N -0.7472 -0.6370 1.6271
24 C -0.8596 -1.2992 2.7810
25 C -2.1783 -1.7279 3.0027
26 C -2.8606 -1.2696 1.8859
27 N -1.9819 -0.6210 1.0827
28 C 0.3424 -1.4539 3.6419
29 F 1.3796 -2.0749 3.0134
30 F 2.3548 -2.2090 -1.7555
31 F 2.0168 -1.9219 -3.9012
32 F 1.5084 4.1704 -0.4076
33 F 0.4572 5.2243 1.1922
34 F 0.8454 -0.2380 4.0565
35 F 0.0657 -2.1617 4.7664
36 N 3.3258 0.9394 2.6075
37 C 4.2627 1.5679 3.5435
38 Cl 6.0997 1.3862 -2.7021
39 Cl 4.2321 2.8856 -0.9995
40 H -2.1364 4.8597 0.1535
41 H -3.6730 2.6290 -0.3979
42 H -0.7184 -1.9429 -4.2572
43 H -2.9072 -1.0876 -2.7999
44 H -2.5650 -2.2819 3.8443
45 H -3.9009 -1.3555 1.6076
46 H -3.4212 0.0544 -0.5215
47 H 6.1536 -0.1977 -0.3254
48 H 4.4530 -0.0245 -0.8741
49 H 5.2307 1.0711 3.4433
50 H 2.3436 0.9080 2.8523
51 H 3.8860 1.3531 4.5478
52 C 4.3966 3.0740 3.3168
53 H 5.0933 3.5047 4.0454
54 H 4.7787 3.2788 2.3117

55 H 3.4285 3.5745 3.4232

eInt5$_N$_singlet
E = -2355.44164914 Hartree
Atom X Y Z
1 C 3.3936 -3.9535 0.0080
2 N 3.1334 -3.0065 0.9147
3 N 4.2532 -2.8970 1.6639
4 C 5.2030 -3.7612 1.2301
5 C 4.6918 -4.4678 0.1540
6 Cu 1.4871 -1.7601 1.5627
7 N -0.1494 -1.0441 0.4350
8 C -1.3403 -1.6481 0.8809
9 O -1.7914 -0.9628 1.9694
10 C -3.0144 -1.3906 2.5468
11 C -4.1083 -0.3401 2.2930
12 Cl -5.6116 -0.9288 3.0875
13 B 4.3248 -1.9313 2.8730
14 N 4.1108 -0.4744 2.3953
15 N 2.9799 -0.1057 1.7590
16 C 3.1203 1.1975 1.4970
17 C 4.3507 1.6917 1.9603
18 C 4.9513 0.5813 2.5306
19 C 2.0485 1.9223 0.7666
20 F 0.8102 1.7582 1.3421
21 C 2.3730 -4.3097 -1.0146
22 F 2.3109 -3.4022 -2.0403
23 N 1.9132 -2.3106 3.5626
24 N 3.2236 -2.3168 3.8954
25 C 3.3878 -2.7426 5.1712
26 C 2.1404 -3.0282 5.7014
27 C 1.2599 -2.7413 4.6467
28 C -0.2245 -2.8219 4.6457
29 F -0.7284 -3.2905 3.4670
30 O -1.8606 -2.6211 0.3929
31 Cl -3.6387 1.2435 2.9992
32 Cl -4.3974 -0.1373 0.5320
33 F 2.2874 3.2548 0.7139
34 F 1.8967 1.4917 -0.5261
35 F -0.6772 -3.6410 5.6328
36 F -0.8241 -1.6058 4.8512
37 F 2.6441 -5.5148 -1.5842
38 F 1.1136 -4.3753 -0.4972
39 C 0.1437 -1.2072 -1.0192
40 H 4.7322 2.6987 1.8881
41 H 5.9072 0.4654 3.0201
42 H 1.8976 -3.3925 6.6878
43 H 4.3750 -2.8161 5.6034
44 H 5.1700 -5.2379 -0.4316
45 H 6.1646 -3.8130 1.7194
46 H 5.4002 -2.0245 3.3959
47 H -3.3318 -2.3460 2.1251
48 H -2.8634 -1.4763 3.6228
49 H 0.0543 -2.2699 -1.2378
50 C -0.7798 -0.3690 -1.8958
51 H -0.1338 -0.0627 0.7162
52 H 1.1872 -0.9185 -1.1557
53 H -0.5138 -0.5023 -2.9502
54 H -1.8250 -0.6711 -1.7685
55 H -0.6891 0.6971 -1.6564

eInt4$_{ON}$_oss
E = -2355.3303324 Hartree
Atom X Y Z
1 C 1.4698 -1.7836 -1.4727
2 N 0.9272 -0.9253 -0.6017
3 N 1.9560 -0.2963 0.0083
4 C 3.1329 -0.7515 -0.4771
5 C 2.8690 -1.7157 -1.4388
6 Cu -1.0156 -0.3757 -0.0603
7 N -2.0709 -0.5443 -1.6979
8 C -3.1530 -0.1740 -1.0630
9 O -4.2917 0.0989 -1.7430
10 C -5.4280 0.4860 -0.9863
11 C -6.4860 -0.6307 -1.0024
12 Cl -7.9035 -0.0413 -0.0583
13 B 1.7069 0.8131 1.0684
14 N 0.9976 2.0030 0.3900
15 N -0.1943 1.8249 -0.2177
16 C -0.5077 3.0058 -0.7554
17 C 0.4829 3.9703 -0.4991
18 C 1.4297 3.2798 0.2391
19 C -1.7553 3.1657 -1.5563
20 F -2.8761 2.7660 -0.8887
21 C 0.6117 -2.6482 -2.3359
22 F 0.0064 -1.9536 -3.3445
23 N -0.3956 -0.3410 1.8813
24 N 0.7958 0.2252 2.1774
25 C 1.0246 0.1555 3.5072
26 C -0.0512 -0.4824 4.1065
27 C -0.9093 -0.7754 3.0390
28 C -2.2304 -1.4741 3.0955
29 F -2.4096 -2.3197 2.0456
30 O -3.0734 -0.0574 0.2039
31 Cl -7.0060 -0.9836 -2.6897
32 Cl -5.8400 -2.1248 -0.2573
33 F -1.9435 4.4692 -1.9049
34 F -1.7337 2.4477 -2.7188
35 F -2.3188 -2.2194 4.2351
36 F -3.2872 -0.6201 3.1095
37 F 1.3577 -3.6209 -2.9287
38 F -0.3799 -3.2641 -1.6395
39 C -2.7747 -0.7371 -4.6695
40 H 0.4957 5.0045 -0.8070
41 H 2.3704 3.5988 0.6639
42 H -0.1956 -0.7162 5.1499
43 H 1.9346 0.5568 3.9282
44 H 3.5714 -2.2914 -2.0215
45 H 4.0681 -0.3612 -0.1035
46 H 2.7497 1.1642 1.5420
47 H -5.1583 0.6986 0.0496
48 H -5.8570 1.3730 -1.4576
49 H -1.8017 -0.6623 -5.1483
50 H -2.0281 -0.4896 -2.7183
51 H -3.3780 0.1656 -4.6270
52 C -3.3851 -2.0682 -4.4020
53 H -3.8514 -2.4865 -5.3128
54 H -2.6362 -2.7960 -4.0676
55 H -4.1715 -2.0029 -3.6425

eTS3-4$_N$_triplet
E = -2355.30843578 Hartree
Atom X Y Z
1 N 3.7254 -33.1920 7.7617
2 N 3.6853 -32.7748 6.4790
3 C 2.4627 -32.2715 6.2814
4 C 1.6854 -32.3633 7.4436
5 C 2.5373 -32.9584 8.3642
6 Cu 5.4168 -32.9056 5.3348
7 N 6.1292 -32.6304 3.6496
8 C 7.1968 -33.2429 3.0519
9 O 7.3062 -32.8774 1.7297
10 C 8.4116 -33.3849 1.0060
11 C 9.3912 -32.2469 0.6680
12 Cl 10.0228 -31.4959 2.1649
13 C 2.1046 -31.7214 4.9429
14 F 3.0358 -30.8268 4.4898
15 B 5.0183 -33.8321 8.3412
16 N 5.3458 -35.1026 7.5319
17 N 5.5064 -35.0424 6.1907
18 C 5.6781 -36.3035 5.7887
19 C 5.6406 -37.2005 6.8712
20 C 5.4237 -36.3848 7.9675
21 C 5.8680 -36.6455 4.3482
22 F 5.1943 -37.7956 4.0348
23 N 6.1747 -32.8057 8.1978
24 C 6.9921 -32.3148 9.1565
25 C 7.8748 -31.4210 8.5676
26 C 7.5108 -31.4324 7.2147
27 N 6.4901 -32.2697 6.9988
28 C 8.1021 -30.6545 6.0875
29 F 8.7826 -31.4316 5.2015
30 O 7.9420 -34.0370 3.5989
31 F 5.4084 -35.6764 3.5144
32 F 7.1689 -36.8760 4.0178
33 F 7.1505 -29.9945 5.3624
34 F 8.9752 -29.7202 6.5509
35 F 2.0169 -32.6854 3.9817
36 F 0.9089 -31.0795 4.9776
37 Cl 10.7484 -32.9628 -0.2772
38 Cl 8.5768 -30.9877 -0.3289
39 C 5.7254 -30.2785 2.6117
40 H 5.2783 -29.7384 3.4492
41 C 4.8141 -30.5485 1.4432
42 H 5.9554 -31.4384 3.1435
43 H 5.7447 -38.2745 6.8454
44 H 5.3117 -36.6192 9.0162
45 H 8.6565 -30.8434 9.0361
46 H 6.8860 -32.6329 10.1833
47 H 0.6656 -32.0404 7.5856
48 H 2.3837 -33.2286 9.3987
49 H 4.8590 -34.1020 9.4974
50 H 8.9429 -34.1460 1.5816
51 H 8.0422 -33.8066 0.0679
52 H 3.8790 -31.0179 1.7686
53 H 4.5531 -29.6070 0.9353
54 H 5.2965 -31.2012 0.7083
55 H 6.7255 -29.9159 2.3657

eInt4$_{ON}$_triplet
E = -2355.33267536 Hartree
Atom X Y Z
1 C 1.1655 -2.1272 -1.5004
2 N 0.6733 -1.1677 -0.7070
3 N 1.7280 -0.4226 -0.3062
4 C 2.8683 -0.9012 -0.8509
5 C 2.5542 -2.0030 -1.6331
6 Cu -1.2135 -0.6101 -0.0417
7 N -2.4348 -1.1096 -1.4900
8 C -3.4232 -0.4934 -0.8824
9 O -4.5800 -0.2491 -1.5316
10 C -5.5868 0.4729 -0.8340
11 C -6.7594 -0.4536 -0.4698
12 Cl -7.9988 0.5602 0.3598
13 B 1.5568 0.7642 0.6803
14 N 0.7311 1.8857 0.0191
15 N -0.5343 1.6558 -0.3932
16 C -0.9808 2.8284 -0.8527
17 C -0.0043 3.8340 -0.7467
18 C 1.0776 3.1819 -0.1800
19 C -2.3719 2.9690 -1.3685
20 F -3.3188 2.9353 -0.3841
21 C 0.2760 -3.1570 -2.1163
22 F -0.4907 -2.6600 -3.1294
23 N -0.4092 -0.3560 1.8141
24 N 0.7958 0.2452 1.9301
25 C 1.1583 0.3069 3.2304
26 C 0.1592 -0.2776 3.9950
27 C -0.7971 -0.6786 3.0536
28 C -2.1024 -1.3644 3.3011
29 F -2.3814 -2.2931 2.3460
30 O -3.2226 -0.1204 0.3185
31 Cl -7.4810 -1.1740 -1.9518

32 Cl -6.2179 -1.7626 0.6237
33 F -2.5303 4.1554 -2.0185
34 F -2.7132 1.9828 -2.2477
35 F -2.0779 -2.0099 4.5016
36 F -3.1551 -0.5058 3.3422
37 F 1.0191 -4.1658 -2.6497
38 F -0.5766 -3.7114 -1.2155
39 C -1.6121 -0.0096 -4.4792
40 H -0.0850 4.8688 -1.0422
41 H 2.0590 3.5402 0.0947
42 H 0.1255 -0.4094 5.0654
43 H 2.0983 0.7556 3.5162
44 H 3.2198 -2.6333 -2.2021
45 H 3.8170 -0.4312 -0.6373
46 H 2.6335 1.1787 1.0018
47 H -5.1913 0.9166 0.0807
48 H -5.9571 1.2509 -1.5046
49 C -0.3441 0.4601 -3.8543
50 H -2.4228 -1.1543 -2.5054
51 H -2.5219 0.5740 -4.3792
52 H -1.6518 -0.9170 -5.0729
53 H -0.1378 1.5139 -4.0986
54 H -0.3886 0.4107 -2.7557
55 H 0.5155 -0.1375 -4.1757

eInt4$_N$_triplet
E = -2355.32444527 Hartree
Atom X Y Z
1 N -2.4638 -0.3504 3.2092
2 N -2.5298 0.1131 1.9438
3 C -3.7581 0.6168 1.7834
4 C -4.5135 0.4776 2.9546
5 C -3.6423 -0.1455 3.8387
6 Cu -0.8424 0.0273 0.7661
7 N -0.0229 0.3596 -0.8726
8 C 0.9838 -0.3765 -1.4244
9 O 1.2321 0.0462 -2.7183
10 C 2.2905 -0.5817 -3.4114
11 C 3.3969 0.4419 -3.7183
12 Cl 4.0567 1.1271 -2.2027
13 C -4.1402 1.2075 0.4684
14 F -3.2023 2.0959 0.0162
15 B -1.1528 -1.0003 3.7397
16 N -0.8410 -2.2418 2.8823
17 N -0.7768 -2.1490 1.5339
18 C -0.6140 -3.3967 1.0908
19 C -0.5584 -4.3181 2.1522
20 C -0.7096 -3.5322 3.2803
21 C -0.5646 -3.7121 -0.3680
22 F -1.3753 -4.7812 -0.6521
23 N -0.0134 0.0467 3.5996
24 C 0.8138 0.5321 4.5520
25 C 1.6571 1.4672 3.9689
26 C 1.2610 1.4870 2.6256
27 N 0.2588 0.6269 2.4108
28 C 1.8123 2.3098 1.5088
29 F 2.5624 1.5848 0.6347
30 O 1.5773 -1.2979 -0.8957
31 F -0.9896 -2.6815 -1.1426
32 F 0.6753 -4.0665 -0.8010
33 F 0.8286 2.9033 0.7706
34 F 2.6103 3.3002 1.9897
35 F -4.2656 0.2684 -0.5135
36 F -5.3226 1.8670 0.5473
37 Cl 4.6995 -0.4195 -4.6195
38 Cl 2.7569 1.7785 -4.7429
39 C -0.8052 3.0566 -2.2827
40 H -1.2696 3.5324 -1.4233
41 C -1.6131 2.2197 -3.2144
42 H -0.1138 1.2862 -1.3081
43 H -0.4405 -5.3896 2.0937
44 H -0.7431 -3.7927 4.3283
45 H 2.4339 2.0524 4.4362
46 H 0.7416 0.1818 5.5712
47 H -5.5325 0.7889 3.1256
48 H -3.7767 -0.4535 4.8652
49 H -1.2809 -1.3048 4.8907
50 H 2.7178 -1.3945 -2.8197
51 H 1.9098 -0.9651 -4.3620
52 H -2.3995 1.6687 -2.6847
53 H -2.1172 2.8377 -3.9796
54 H -0.9865 1.4980 -3.7523
55 H 0.1856 3.4007 -2.5680

eTS3-5$_{ON}$_singlet
E = -2355.31107931 Hartree
Atom X Y Z
1 C 5.6043 -2.1284 -8.6367
2 N 5.8067 -1.0710 -7.8478
3 N 7.1361 -1.0199 -7.6283
4 C 7.7666 -2.0317 -8.2749
5 C 6.8120 -2.7785 -8.9460
6 Cu 4.6050 0.5563 -6.8881
7 N 3.3188 0.9605 -8.3954
8 C 2.2498 0.8796 -7.6151
9 O 1.0207 1.1789 -8.1187
10 C -0.0982 0.9086 -7.2878
11 C -0.9135 -0.2596 -7.8688
12 Cl -2.3221 -0.5296 -6.7815
13 B 7.7441 0.0796 -6.7235
14 N 7.4713 1.4739 -7.3455
15 N 6.2113 1.9291 -7.4988
16 C 6.3200 3.1406 -8.0501
17 C 7.6646 3.4860 -8.2678
18 C 8.3654 2.3865 -7.7988
19 C 5.1040 3.9359 -8.3759
20 F 4.1416 3.8553 -7.4203
21 C 4.2288 -2.4794 -9.0885
22 F 3.8083 -1.7360 -10.1634
23 N 5.7509 0.1868 -5.1585
24 N 7.0820 0.0121 -5.3232
25 C 7.6792 -0.2060 -4.1278
26 C 6.7080 -0.1727 -3.1396
27 C 5.5223 0.0779 -3.8462
28 C 4.1382 0.2225 -3.3084
29 F 3.2987 -0.7612 -3.7336
30 O 2.4412 0.5526 -6.4197
31 Cl -1.5122 0.1507 -9.5201
32 Cl 0.0883 -1.7377 -7.9554
33 F 5.4123 5.2537 -8.5350
34 F 4.5060 3.5460 -9.5508
35 F 4.1472 0.1732 -1.9446
36 F 3.5620 1.4053 -3.6607
37 F 4.1647 -3.7818 -9.4884
38 F 3.2939 -2.3054 -8.1192
39 C 2.4973 1.1915 -10.6796
40 H 8.0550 4.4003 -8.6879
41 H 9.4265 2.1901 -7.7491
42 H 6.8281 -0.3050 -2.0755
43 H 8.7456 -0.3668 -4.0681
44 H 6.9548 -3.6609 -9.5509
45 H 8.8382 -2.1486 -8.2043
46 H 8.9271 -0.0896 -6.6202
47 H 0.2192 0.6486 -6.2756
48 H -0.7323 1.7979 -7.2692
49 H 1.9533 0.2648 -10.5266
50 H 1.8862 2.0924 -10.6361
51 H 3.1660 1.4133 -9.3894
52 C 3.6902 1.1644 -11.5676
53 H 3.3430 0.9849 -12.6008
54 H 4.2394 2.1092 -11.5573
55 H 4.3599 0.3390 -11.3083

Propane amidation---------------------------------------

$CH_3CH_2CH_3$
E = -119.149680404 Hartree
Atom X Y Z
1 C -3.6592 -2.0437 -3.0404
2 C -2.7362 -3.1206 -3.6178
3 H -3.2526 -1.6172 -2.1152
4 H -3.8006 -1.2208 -3.7532
5 H -4.6509 -2.4547 -2.8108
6 C -3.2812 -3.7329 -4.9114
7 H -1.7441 -2.6884 -3.8076
8 H -2.5884 -3.9137 -2.8719
9 H -2.6072 -4.5013 -5.3096
10 H -4.2601 -4.2013 -4.7451
11 H -3.4100 -2.9672 -5.6874

tpTS3-5$_{ON}$_singlet
E = -2394.63034554 Hartree
Atom X Y Z
1 C -0.6021 3.2299 -0.2786
2 N -0.3412 3.1161 -1.5852
3 N 0.9472 2.7162 -1.6758
4 C 1.4860 2.5771 -0.4419
5 C 0.5188 2.8987 0.4971
6 Cu -1.3833 3.4718 -3.3878
7 O -3.5495 3.6317 -2.9165
8 C -3.7038 4.0317 -4.0956
9 O -4.8961 4.4750 -4.5809
10 C -6.0388 4.2909 -3.7597
11 C -6.9906 3.2736 -4.4117
12 Cl -6.1852 1.6886 -4.5887
13 B 1.6301 2.4891 -3.0499
14 N 1.6061 3.8177 -3.8460
15 N 0.4495 4.4699 -4.0859
16 C 0.7883 5.5990 -4.7157
17 C 2.1777 5.6867 -4.9017
18 C 2.6595 4.5229 -4.3252
19 C -0.2489 6.5795 -5.1381
20 F -0.8929 6.2127 -6.2953
21 C -1.9510 3.6602 0.1920
22 F -2.3566 4.8274 -0.3805
23 N -0.4464 1.5190 -4.1068
24 C -0.8129 0.3590 -4.6585
25 C 0.2647 -0.5395 -4.7428
26 C 1.3195 0.1636 -4.1870
27 N 0.8658 1.3871 -3.8168
28 C -2.2085 0.1430 -5.1284
29 F -3.1215 0.8508 -4.4191
30 N -2.6265 4.0465 -4.8655
31 Cl -8.4329 3.1200 -3.3467
32 Cl -7.5159 3.8583 -6.0365
33 F -1.2212 6.7641 -4.2078
34 F 0.3085 7.7968 -5.3916
35 F -2.3852 0.4928 -6.4445
36 F -2.5568 -1.1738 -5.0469
37 F -2.9222 2.7412 -0.0630
38 F -1.9435 3.8596 1.5426
39 C -3.3384 4.5243 -7.1989
40 C -2.7406 3.3395 -7.8694
41 H 2.7342 6.4836 -5.3707
42 H 3.6675 4.1508 -4.2139
43 H 0.6016 2.9003 1.5729
44 H 2.5126 2.2627 -0.3232
45 H 0.2615 -1.5445 -5.1366
46 H 2.3497 -0.1158 -4.0204
47 H 2.7671 2.1463 -2.8806
48 H -5.7564 3.9278 -2.7689

49 H -6.5596 5.2479 -3.6778
50 H -4.4001 4.5149 -6.9666
51 H -2.9157 5.5077 -7.4098
52 H -2.7353 4.5092 -5.8693
53 H -3.0393 3.4181 -8.9333
54 C -1.2113 3.2529 -7.7733
55 H -3.2042 2.4249 -7.4899
56 H -0.8429 2.4010 -8.3535
57 H -0.7351 4.1635 -8.1541
58 H -0.9013 3.1147 -6.7327

ipMECP4-5_singlet
E = -2394.64911823 Hartree
Atom X Y Z
1 N -1.4383 -1.9563 -0.9560
2 N -2.7279 -2.3136 -1.1469
3 C -2.7964 -3.5723 -1.6329
4 C -1.5058 -4.0626 -1.7684
5 C -0.6977 -3.0081 -1.3262
6 B -3.8878 -1.3791 -0.7186
7 N -3.7122 -0.0132 -1.4286
8 N -2.5840 0.7172 -1.2784
9 C -2.7728 1.8485 -1.9694
10 C -4.0316 1.8593 -2.5826
11 C -4.5955 0.6487 -2.2078
12 Cu -1.0363 -0.0728 -0.1819
13 N 0.5610 0.4409 0.6449
14 C 1.6654 0.8380 -0.0524
15 O 2.7542 0.9996 0.7826
16 C 3.9812 1.3486 0.1759
17 C 4.9655 0.1664 0.2303
18 Cl 4.3193 -1.2374 -0.6677
19 C -1.7056 2.8906 -2.0387
20 F -1.1770 3.1640 -0.8118
21 C 0.7907 -2.9629 -1.2234
22 F 1.3432 -2.0054 -2.0157
23 N -2.7109 -0.6148 1.3611
24 C -2.9306 -0.6029 2.6776
25 C -4.1829 -1.1538 2.9997
26 C -4.7101 -1.5059 1.7676
27 N -3.8104 -1.1701 0.8102
28 C -1.9184 0.0058 3.5840
29 F -0.6492 -0.4357 3.3271
30 F 1.2123 -2.7048 0.0475
31 F 1.3332 -4.1548 -1.5932
32 F -0.6670 2.5314 -2.8382
33 F -2.2053 4.0554 -2.5347
34 F -1.8668 1.3683 3.4730
35 F -2.1839 -0.2724 4.8866
36 O 1.7019 1.0514 -1.2513
37 Cl 6.5076 0.7171 -0.5274
38 Cl 5.2826 -0.3124 1.9359
39 C 0.6168 3.3037 1.9653
40 C 1.4552 4.0306 0.9701
41 H -4.4557 2.6353 -3.2005
42 H -5.5569 0.2131 -2.4368
43 H -1.1897 -5.0294 -2.1283
44 H -3.7532 -4.0260 -1.8446
45 H -4.6248 -1.2773 3.9766
46 H -5.6494 -1.9693 1.5025
47 H -4.9388 -1.8668 -1.0226
48 H 3.8346 1.6301 -0.8691
49 H 4.4191 2.1781 0.7359
50 H -0.4321 3.1377 1.7357
51 H 0.7469 0.3289 1.6400
52 C 1.1292 2.9939 3.3299
53 H 1.3341 5.1271 1.0494
54 H 2.5246 3.8280 1.1179
55 H 1.1904 3.7621 -0.0595
56 H 1.2803 3.9078 3.9348
57 H 0.4460 2.3469 3.8899
58 H 2.1122 2.4991 3.2843

ipInt4$_{ON}$_oss
E = -2394.4826995 Hartree
Atom X Y Z
1 C -0.7834 -0.6937 3.1329
2 N -0.3216 -0.3455 1.9265
3 N 0.8827 0.2342 2.1270
4 C 1.1716 0.2572 3.4472
5 C 0.1228 -0.3315 4.1382
6 Cu -1.0254 -0.5289 0.0119
7 O -3.0533 -0.0521 0.2709
8 C -3.1857 -0.3862 -0.9516
9 O -4.3298 -0.1637 -1.6384
10 C -5.3934 0.4784 -0.9524
11 C -6.5308 -0.5177 -0.6669
12 Cl -5.9654 -1.8442 0.3918
13 B 1.7242 0.7688 0.9362
14 N 0.9561 1.9199 0.2555
15 N -0.2883 1.7219 -0.2287
16 C -0.6937 2.9130 -0.6776
17 C 0.2896 3.9007 -0.4899
18 C 1.3306 3.2161 0.1135
19 C -2.0619 3.0957 -1.2398
20 F -2.3891 2.1484 -2.1677
21 C -2.1094 -1.3667 3.2874
22 F -3.1517 -0.4944 3.2967
23 N 0.8942 -1.1119 -0.5485
24 C 1.4167 -2.0668 -1.3271
25 C 2.8144 -1.9745 -1.3644
26 C 3.0997 -0.8946 -0.5420
27 N 1.9358 -0.3988 -0.0656
28 C 0.5467 -3.0738 -2.0046
29 F -0.3859 -3.5996 -1.1688
30 N -2.1517 -0.9335 -1.5410
31 Cl -7.8487 0.3992 0.1547
32 Cl -7.1634 -1.2104 -2.2036
33 F -3.0452 3.0450 -0.2943
34 F -2.1752 4.3073 -1.8533
35 F -0.1306 -2.5631 -3.0743
36 F 1.2980 -4.1055 -2.4801
37 F -2.3484 -2.2635 2.2923
38 F -2.1604 -2.0472 4.4683
39 C -2.1211 -0.5691 -4.6408
40 C -0.8618 0.2287 -4.5522
41 C -3.4399 0.1113 -4.7986
42 H 0.2383 4.9452 -0.7564
43 H 2.3007 3.5514 0.4505
44 H 0.0258 -0.4898 5.2012
45 H 2.0999 0.6838 3.7977
46 H 3.5027 -2.6112 -1.8983
47 H 4.0423 -0.4508 -0.2570
48 H 2.7864 1.1581 1.3305
49 H -5.0543 0.9041 -0.0067
50 H -5.7814 1.2627 -1.6062
51 H -2.0471 -1.6227 -4.9043
52 H -2.1228 -1.0023 -2.5624
53 H -0.9503 1.0284 -3.8056
54 H -0.0006 -0.3944 -4.2927
55 H -0.6344 0.7238 -5.5144
56 H -3.5251 0.9772 -4.1311
57 H -3.5694 0.4908 -5.8294
58 H -4.2784 -0.5632 -4.5943

ipInt5$_{O}$_singlet
E = -2394.76308269 Hartree
Atom X Y Z
1 N -1.8303 -0.7202 1.1516
2 N -0.5616 -0.7113 1.6122
3 C -0.5702 -1.4408 2.7310
4 C -1.8531 -1.9368 3.0140
5 C -2.6265 -1.4492 1.9718
6 Cu 0.8453 0.3133 0.3741
7 O 2.8332 0.2847 0.3370
8 C 3.6617 0.6444 1.1849
9 N 3.3767 0.9946 2.4399
10 C 4.3317 1.3702 3.4963
11 H 5.1387 1.9246 3.0092
12 C 0.6866 -1.5815 3.5115
13 F 0.5380 -2.4353 4.5554
14 B -2.2192 0.0555 -0.1385
15 N -1.8393 1.5500 0.0450
16 N -0.5600 1.9201 0.2714
17 C -0.5824 3.2456 0.4313
18 C -1.8841 3.7582 0.3039
19 C -2.6550 2.6331 0.0584
20 C 0.6885 3.9798 0.6798
21 F 0.4493 5.2346 1.1502
22 N -1.4523 -0.5295 -1.3497
23 N -0.1031 -0.5602 -1.3591
24 C 0.2465 -1.0856 -2.5346
25 C -0.8803 -1.4070 -3.3104
26 C -1.9467 -1.0315 -2.5087
27 C 1.6899 -1.2626 -2.8585
28 F 1.8493 -1.7840 -4.1074
29 O 4.9879 0.7037 0.8971
30 C 5.3594 0.5435 -0.4664
31 C 5.7089 1.9052 -1.0936
32 Cl 4.2977 3.0005 -1.0592
33 Cl 7.0797 2.6707 -0.2068
34 Cl 6.2049 1.5919 -2.7952
35 F 1.4871 3.3511 1.5936
36 F 1.4517 4.1259 -0.4461
37 F 2.3912 -0.0910 -2.8243
38 F 2.3292 -2.1045 -1.9933
39 F 1.7384 -2.0236 2.7662
40 F 1.1053 -0.3793 4.0465
41 H -2.2042 4.7858 0.3838
42 H -3.7175 2.5240 -0.1034
43 H -0.9044 -1.8430 -4.2973
44 H -3.0126 -1.0837 -2.6771
45 H -2.1600 -2.5519 3.8460
46 H -3.6785 -1.5663 1.7563
47 H -3.4009 -0.0399 -0.3218
48 H 6.2511 -0.0855 -0.4873
49 H 4.5530 0.0873 -1.0434
50 C 4.9046 0.1187 4.1699
51 H 2.4055 0.8765 2.7024
52 C 3.6098 2.2898 4.4824
53 H 5.6279 0.3971 4.9449
54 H 4.1035 -0.4658 4.6372
55 H 5.4130 -0.5165 3.4370
56 H 4.3015 2.6183 5.2648
57 H 3.2119 3.1746 3.9744
58 H 2.7780 1.7637 4.9680

ipTS3-4$_{N}$_triplet
E = -2394.63137302 Hartree
Atom X Y Z
1 C 3.8418 -33.3980 7.7633
2 N 3.6742 -32.7354 6.5963
3 N 2.4206 -32.2396 6.5192
4 C 1.7995 -32.6022 7.6467
5 C 2.6536 -33.3394 8.4774
6 B 4.6942 -32.5222 5.4447
7 N 5.0106 -31.0209 5.2939
8 N 4.0402 -30.1415 4.9575
9 C 4.6510 -28.9621 4.8248
10 C 6.0301 -29.0614 5.0833
11 C 6.2140 -30.4002 5.3785
12 Cu 1.9319 -31.0978 4.8526
13 N 0.5468 -29.8838 4.9861

14 C 0.5167 -28.7284 5.7155
15 O 1.3543 -28.4110 6.5432
16 C 3.9046 -27.7312 4.4320
17 F 2.7084 -28.0038 3.8476
18 C 0.3663 -32.2504 7.8623
19 F -0.4197 -32.6588 6.8190
20 N 2.8888 -32.5062 3.6781
21 C 2.6041 -33.1332 2.5323
22 C 3.5885 -34.0785 2.2156
23 C 4.4933 -33.9759 3.2635
24 N 4.0502 -33.0309 4.1235
25 C 1.3538 -32.7836 1.8007
26 F 1.3113 -31.4700 1.4335
27 F -0.1158 -32.8476 8.9845
28 F 0.1490 -30.9139 7.9956
29 F 3.6552 -26.8953 5.4795
30 F 4.6293 -26.9946 3.5339
31 F 1.2173 -33.5250 0.6725
32 F 0.2351 -32.9931 2.5614
33 O -0.5522 -27.9344 5.3682
34 C -0.6329 -26.6690 6.0018
35 C -1.8628 -26.5993 6.9218
36 Cl -1.8641 -24.9710 7.6956
37 Cl -3.3797 -26.7934 5.9706
38 Cl -1.7835 -27.8694 8.1800
39 C -1.7971 -30.9166 4.4559
40 C -2.1066 -30.2728 3.1228
41 C -2.7816 -30.6797 5.5763
42 H -0.6938 -30.3828 4.7922
43 H 6.7695 -28.2757 5.0462
44 H 7.1051 -30.9557 5.6328
45 H 2.4294 -33.7665 9.4426
46 H 4.7872 -33.8642 7.9988
47 H 3.6274 -34.7317 1.3575
48 H 5.4154 -34.5044 3.4566
49 H 5.6957 -33.1382 5.6749
50 H 0.2602 -26.4769 6.6000
51 H -0.7435 -25.9048 5.2283
52 H -3.7452 -31.1561 5.3357
53 H -2.4289 -31.1038 6.5208
54 H -2.9695 -29.6119 5.7204
55 H -1.4739 -31.9600 4.3693
56 H -2.2768 -29.1960 3.2362
57 H -1.2916 -30.4232 2.4066
58 H -3.0186 -30.7127 2.6908

ipInt5$_N$_singlet
E = -2394.76216591 Hartree
Atom X Y Z
1 N 1.8724 -0.1922 -0.3220
2 N 0.8737 -0.7588 -1.0360
3 C 1.4452 -1.7434 -1.7388
4 C 2.8262 -1.8186 -1.4975
5 C 3.0527 -0.8070 -0.5799
6 Cu -1.0960 0.0720 -0.6396
7 N -2.7752 0.0308 -1.9527
8 C -3.7499 -0.7803 -1.3416
9 O -4.4532 -0.0218 -0.4519
10 C -5.5335 -0.6457 0.2236
11 C -6.8671 -0.0670 -0.2773
12 Cl -6.9306 1.7068 0.0012
13 C 0.6336 -2.6005 -2.6455
14 F 1.2518 -3.7902 -2.8796
15 B 1.5917 0.8849 0.7527
16 N 0.9885 2.1620 0.1248
17 N -0.1823 2.1431 -0.5501
18 C -0.4548 3.4239 -0.8260
19 C 0.5418 4.2869 -0.3397
20 C 1.4450 3.4313 0.2654
21 C -1.6666 3.8135 -1.5949
22 F -2.0623 5.0768 -1.2899
23 N -0.6491 -0.0836 1.4396
24 C -1.2113 -0.5585 2.5562
25 C -0.3377 -0.4766 3.6516
26 C 0.8089 0.0828 3.1122
27 N 0.5959 0.3068 1.7932
28 C -2.6182 -1.0364 2.5447
29 F -2.8817 -1.8309 3.6165
30 O -3.9249 -1.9541 -1.5594
31 Cl -7.0845 -0.3953 -2.0297
32 Cl -8.1815 -0.8681 0.6547
33 F -1.4783 3.7900 -2.9516
34 F -2.7352 2.9898 -1.3562
35 F -3.5269 -0.0091 2.5954
36 F -2.9275 -1.7502 1.4242
37 F -0.6005 -2.8733 -2.1375
38 F 0.4192 -2.0329 -3.8748
39 C -2.4235 -0.3488 -3.3664
40 C -1.2855 0.5450 -3.8533
41 C -3.6506 -0.2465 -4.2760
42 H 0.5828 5.3627 -0.4155
43 H 2.3685 3.6302 0.7893
44 H -0.5202 -0.7841 4.6698
45 H 1.7565 0.3329 3.5663
46 H 3.5359 -2.5135 -1.9197
47 H 3.9585 -0.4883 -0.0852
48 H 2.6188 1.1661 1.3052
49 H -5.5275 -1.7244 0.0568
50 H -5.4387 -0.4210 1.2857
51 H -2.0877 -1.3840 -3.3115
52 H -3.0559 1.0108 -1.8954
53 H -3.3805 -0.5308 -5.2988
54 H -4.4523 -0.9119 -3.9428
55 H -4.0328 0.7820 -4.3004
56 H -0.9857 0.2365 -4.8593
57 H -1.5999 1.5927 -3.8986
58 H -0.4080 0.4758 -3.2040

tpInt4$_{ON}$_triplet
E = -2394.65132270 Hartree
Atom X Y Z
1 C -0.7654 -0.5875 3.0201
2 N -0.4004 -0.3033 1.7643
3 N 0.8113 0.2907 1.8392
4 C 1.2004 0.3868 3.1297
5 C 0.2125 -0.1676 3.9306
6 Cu -1.2270 -0.6440 -0.0708
7 O -3.2377 -0.1290 0.2702
8 C -3.4455 -0.5880 -0.8987
9 O -4.6040 -0.3880 -1.5597
10 C -5.5973 0.4047 -0.9219
11 C -6.7892 -0.4679 -0.4940
12 Cl -6.2766 -1.7027 0.6951
13 B 1.5501 0.7677 0.5599
14 N 0.7086 1.8637 -0.1223
15 N -0.5616 1.6137 -0.5070
16 C -1.0265 2.7717 -0.9846
17 C -0.0576 3.7884 -0.9170
18 C 1.0395 3.1588 -0.3547
19 C -2.4260 2.8899 -1.4818
20 F -2.7995 1.8390 -2.2682
21 C -2.0708 -1.2544 3.3137
22 F -3.1170 -0.3872 3.3385
23 N 0.6547 -1.2259 -0.7306
24 C 1.1390 -2.2111 -1.4969
25 C 2.5221 -2.0750 -1.6711
26 C 2.8413 -0.9385 -0.9427
27 N 1.7092 -0.4522 -0.3878
28 C 0.2484 -3.2769 -2.0470
29 F -0.5903 -3.7875 -1.1087
30 N -2.4622 -1.2517 -1.4656
31 Cl -8.0087 0.6300 0.2542
32 Cl -7.5235 -1.2814 -1.9206
33 F -3.3531 2.9560 -0.4807
34 F -2.5814 4.0197 -2.2278
35 F -0.5328 -2.8386 -3.0767
36 F 0.9915 -4.3082 -2.5358
37 F -2.3718 -2.2162 2.3985
38 F -2.0315 -1.8545 4.5370
39 C -1.6482 -0.1995 -4.4907
40 C -0.3833 0.2848 -3.8638
41 H -0.1516 4.8146 -1.2374
42 H 2.0224 3.5315 -0.1056
43 H 0.1983 -0.2676 5.0048
44 H 2.1488 0.8367 3.3839
45 H 3.1815 -2.7202 -2.2307
46 H 3.7892 -0.4492 -0.7728
47 H 2.6308 1.1958 0.8483
48 H -5.1948 0.9072 -0.0414
49 H -5.9503 1.1383 -1.6496
50 H -2.4553 -1.3580 -2.4765
51 H -2.5560 0.3910 -4.4017
52 H -1.6776 -1.0839 -5.1192
53 C 0.0008 1.7121 -4.3022
54 H -0.4905 0.2871 -2.7689
55 H 0.4386 -0.4055 -4.0889
56 H 0.9050 2.0514 -3.7815
57 H 0.1889 1.7578 -5.3813
58 H -0.8038 2.4206 -4.0746

ipTS3-4$_{ON}$_triplet
E = -2394.63258877 Hartree
Atom X Y Z
1 C 1.2275 -2.2075 -1.4255
2 N 0.7235 -1.1936 -0.7125
3 N 1.7691 -0.4052 -0.3766
4 C 2.9159 -0.9095 -0.8863
5 C 2.6149 -2.0736 -1.5763
6 Cu -1.1739 -0.5984 -0.0542
7 N -2.3471 -1.2467 -1.5055
8 C -3.3799 -0.5859 -0.9694
9 O -4.4683 -0.3451 -1.7264
10 C -5.5184 0.4252 -1.1528
11 C -6.7430 -0.4624 -0.8750
12 Cl -8.0298 0.6069 -0.2026
13 B 1.6004 0.7889 0.5976
14 N 0.7352 1.9026 -0.0297
15 N -0.5562 1.6798 -0.3616
16 C -1.0474 2.8747 -0.7073
17 C -0.0760 3.8855 -0.6101
18 C 1.0511 3.2141 -0.1690
19 C -2.4846 3.0430 -1.0597
20 F -3.3136 2.9780 0.0226
21 C 0.3607 -3.3149 -1.9319
22 F -0.3441 -2.9775 -3.0481
23 N -0.3343 -0.3106 1.8041
24 N 0.8832 0.2721 1.8759
25 C 1.2968 0.3248 3.1619
26 C 0.3190 -0.2464 3.9632
27 C -0.6781 -0.6296 3.0567
28 C -1.9897 -1.2845 3.3450
29 F -2.2996 -2.2390 2.4251
30 O -3.2802 -0.2048 0.2324
31 Cl -7.3345 -1.2162 -2.3970
32 Cl -6.3438 -1.7451 0.3068
33 F -2.7044 4.2521 -1.6460
34 F -2.9347 2.0911 -1.9307
35 F -1.9639 -1.8876 4.5665
36 F -3.0272 -0.4044 3.3650
37 F 1.1279 -4.3905 -2.2731
38 F -0.5375 -3.7354 -1.0064
39 C -1.8784 -0.1895 -3.8524
40 H -0.1901 4.9362 -0.8284

41 H 2.0457 3.5693 0.0583
42 H 0.3223 -0.3783 5.0341
43 H 2.2531 0.7597 3.4129
44 H 3.2882 -2.7400 -2.0929
45 H 3.8593 -0.4137 -0.7104
46 H 2.6784 1.2254 0.8847
47 H -5.2022 0.8930 -0.2194
48 H -5.8019 1.1864 -1.8823
49 C -0.4241 0.2300 -3.8058
50 H -2.1017 -0.7836 -2.7601
51 H -2.5639 0.6589 -3.7805
52 C -2.2718 -1.1741 -4.9321
53 H -0.1789 0.8505 -4.6814
54 H -0.2063 0.8176 -2.9076
55 H 0.2407 -0.6395 -3.8186
56 H -2.1525 -0.7145 -5.9259
57 H -1.6457 -2.0715 -4.9024
58 H -3.3192 -1.4796 -4.8307

tpInt4$_{N}$_triplet
E = -2394.64348069 Hartree
Atom X Y Z
1 N 2.8081 -2.7851 -1.0201
2 N 1.6477 -2.3281 -1.5379
3 C 1.4384 -3.0110 -2.6691
4 C 2.4743 -3.9233 -2.9029
5 C 3.3258 -3.7424 -1.8209
6 Cu 0.5987 -0.9169 -0.4870
7 N -0.7975 0.2921 -0.2549
8 C -0.6937 1.4648 0.4302
9 O -1.8507 2.2102 0.2868
10 C -1.9007 3.4562 0.9499
11 C -3.0047 3.4423 2.0207
12 Cl -2.6749 2.1858 3.2507
13 C 0.2237 -2.7257 -3.4852
14 F -0.9153 -2.7458 -2.7276
15 B 3.3690 -2.1982 0.3086
16 N 3.6377 -0.6961 0.1010
17 N 2.6732 0.1139 -0.3938
18 C 3.2465 1.3080 -0.5493
19 C 4.5942 1.2904 -0.1465
20 C 4.8000 -0.0147 0.2623
21 C 2.5123 2.4617 -1.1483
22 F 3.2414 3.0157 -2.1700
23 N 2.2944 -2.4077 1.4114
24 C 2.4115 -3.0313 2.6050
25 C 1.1787 -3.0004 3.2417
26 C 0.3502 -2.3219 2.3389
27 N 1.0302 -1.9650 1.2437
28 C -1.0993 -1.9932 2.4737
29 F -1.3253 -0.6800 2.7519
30 O 0.2751 1.8493 1.0597
31 F 1.3116 2.1081 -1.6732
32 F 2.2912 3.4747 -0.2666
33 F -1.7964 -2.2680 1.3318
34 F -1.6654 -2.7138 3.4780
35 F 0.2627 -1.4937 -4.0696
36 F 0.0698 -3.6373 -4.4773
37 Cl -3.0259 5.0694 2.7983
38 Cl -4.6121 3.1277 1.2702
39 C -3.7076 -0.5983 -1.0325
40 C -3.7617 0.4289 -2.1148
41 H -4.1697 -0.4098 -0.0665
42 H -1.7735 -0.0087 -0.3757
43 H 5.3026 2.1049 -0.1672
44 H 5.6815 -0.5093 0.6440
45 H 0.9105 -3.4101 4.2032
46 H 3.3568 -3.4539 2.9123
47 H 2.5777 -4.6085 -3.7301
48 H 4.2572 -4.2262 -1.5656
49 H 4.3764 -2.7682 0.6159
50 H -0.9464 3.6803 1.4320
51 H -2.1473 4.2318 0.2196
52 H -3.4466 -1.6286 -1.2666
53 C -2.7232 0.2100 -3.2248
54 H -4.7729 0.4294 -2.5651
55 H -3.6372 1.4284 -1.6764
56 H -2.8438 0.9468 -4.0274
57 H -1.7051 0.3048 -2.8323
58 H -2.8219 -0.7888 -3.6663

ipInt4$_{N}$_triplet
E = -2394.64975074 Hartree
Atom X Y Z
1 C 2.5090 -3.0459 2.4895
2 N 2.3637 -2.4213 1.2996
3 N 1.1051 -1.9485 1.1760
4 C 0.4573 -2.2875 2.2965
5 C 1.3011 -2.9849 3.1701
6 B 3.4075 -2.2311 0.1642
7 N 3.7047 -0.7337 -0.0392
8 N 2.7397 0.1045 -0.4827
9 C 3.3352 1.2859 -0.6531
10 C 4.6986 1.2313 -0.3101
11 C 4.8897 -0.0827 0.0767
12 Cu 0.6397 -0.8813 -0.5365
13 N -0.7547 0.3278 -0.3024
14 C -0.6542 1.5031 0.3776
15 O 0.3181 1.8921 0.9989
16 C 2.6030 2.4638 -1.2063
17 F 1.3886 2.1380 -1.7167
18 C -0.9815 -1.9354 2.4774
19 F -1.7283 -2.2612 1.3804
20 N 1.6274 -2.3044 -1.6274
21 C 1.3669 -2.9691 -2.7586
22 C 2.3717 -3.9046 -3.0330
23 C 3.2607 -3.7555 -1.9762
24 N 2.7913 -2.7945 -1.1502
25 C 0.1233 -2.6554 -3.5186
26 F 0.0917 -1.3627 -3.9550
27 F -1.5106 -2.5998 3.5388
28 F -1.1867 -0.6083 2.6969
29 F 2.4108 3.4553 -0.2939
30 F 3.3197 3.0360 -2.2266
31 F -0.0090 -3.4553 -4.6048
32 F -0.9995 -2.8160 -2.7525
33 O -1.8156 2.2450 0.2430
34 C -1.8453 3.5029 0.8870
35 C -2.9341 3.5219 1.9727
36 Cl -2.8954 5.1499 2.7487
37 Cl -4.5615 3.2591 1.2463
38 Cl -2.6247 2.2567 3.1990
39 C -3.4745 -0.8970 -1.1982
40 C -3.4245 -0.0043 -2.3956
41 C -4.3515 -0.5725 -0.0336
42 H -1.7244 0.0132 -0.4301
43 H 5.4253 2.0285 -0.3546
44 H 5.7746 -0.6026 0.4143
45 H 1.0577 -3.3870 4.1413
46 H 3.4546 -3.4902 2.7634
47 H 2.4335 -4.5823 -3.8705
48 H 4.1882 -4.2631 -1.7555
49 H 4.4102 -2.8269 0.4357
50 H -0.8823 3.7216 1.3538
51 H -2.0908 4.2704 0.1479
52 H -5.4204 -0.7281 -0.2720
53 H -4.1204 -1.1957 0.8371
54 H -4.2521 0.4803 0.2620
55 H -3.1013 -1.9143 -1.3010
56 H -3.3552 1.0532 -2.1052
57 H -2.5721 -0.2388 -3.0437
58 H -4.3372 -0.0999 -3.0124

ipInt4$_{ON}$_triplet
E = -2394.65693733 Hartree
Atom X Y Z
1 C -0.7455 -0.6335 3.0680
2 N -0.3440 -0.3282 1.8284
3 N 0.8643 0.2644 1.9478
4 C 1.2162 0.3388 3.2504
5 C 0.2058 -0.2289 4.0131
6 Cu -1.1258 -0.6281 -0.0350
7 O -3.1299 -0.0892 0.2723
8 C -3.3367 -0.5766 -0.8853
9 O -4.4976 -0.3945 -1.5497
10 C -5.4835 0.4297 -0.9395
11 C -6.6652 -0.4190 -0.4409
12 Cl -6.1331 -1.5789 0.8127
13 B 1.6359 0.7634 0.6958
14 N 0.8144 1.8751 0.0156
15 N -0.4515 1.6404 -0.3925
16 C -0.9072 2.8135 -0.8408
17 C 0.0640 3.8244 -0.7323
18 C 1.1522 3.1750 -0.1754
19 C -2.3086 2.9591 -1.3254
20 F -2.7133 1.9171 -2.1052
21 C -2.0589 -1.3050 3.3114
22 F -3.1041 -0.4364 3.3355
23 N 0.7658 -1.1979 -0.6627
24 C 1.2699 -2.1769 -1.4250
25 C 2.6594 -2.0526 -1.5447
26 C 2.9617 -0.9302 -0.7877
27 N 1.8144 -0.4403 -0.2690
28 C 0.3940 -3.2322 -2.0161
29 F -0.4528 -3.7750 -1.1030
30 N -2.3525 -1.2503 -1.4347
31 Cl -7.8766 0.7199 0.2573
32 Cl -7.4165 -1.3186 -1.8056
33 F -3.2215 3.0468 -0.3120
34 F -2.4501 4.0926 -2.0688
35 F -0.3798 -2.7693 -3.0412
36 F 1.1490 -4.2436 -2.5254
37 F -2.3401 -2.2398 2.3622
38 F -2.0507 -1.9398 4.5175
39 C -1.5174 -0.2725 -4.6191
40 C -0.4042 0.3838 -3.8710
41 C -2.8450 0.3865 -4.8024
42 H -0.0240 4.8607 -1.0204
43 H 2.1327 3.5372 0.0975
44 H 0.1614 -0.3470 5.0847
45 H 2.1569 0.7839 3.5392
46 H 3.3330 -2.6959 -2.0893
47 H 3.9068 -0.4524 -0.5753
48 H 2.7108 1.1812 1.0187
49 H -5.0668 0.9840 -0.0973
50 H -5.8512 1.1177 -1.7034
51 H -1.4181 -1.3246 -4.8755
52 H -2.3556 -1.4224 -2.4340
53 H -0.5366 0.3178 -2.7784
54 H 0.5654 -0.0753 -4.1005
55 H -0.3399 1.4560 -4.1063
56 H -3.4974 0.2750 -3.9187
57 H -2.7389 1.4679 -4.9651
58 H -3.3931 -0.0367 -5.6538

tpInt4$_{ON}$_oss
E = -2394.6513227 Hartree
Atom X Y Z
1 C -0.7641 -0.5850 3.0315
2 N -0.3992 -0.3021 1.7753
3 N 0.8133 0.2904 1.8496
4 C 1.2028 0.3868 3.1400

5 C 0.2145 -0.1659 3.9415
6 Cu -1.2262 -0.6406 -0.0596
7 O -3.2402 -0.1374 0.2744
8 C -3.4372 -0.5711 -0.9064
9 O -4.5938 -0.3654 -1.5694
10 C -5.5974 0.4028 -0.9184
11 C -6.7816 -0.4910 -0.5129
12 Cl -6.2607 -1.7438 0.6536
13 B 1.5542 0.7649 0.5704
14 N 0.7161 1.8624 -0.1142
15 N -0.5550 1.6165 -0.4987
16 C -1.0147 2.7751 -0.9801
17 C -0.0413 3.7877 -0.9159
18 C 1.0529 3.1551 -0.3511
19 C -2.4138 2.8979 -1.4777
20 F -2.7824 1.8621 -2.2864
21 C -2.0701 -1.2507 3.3253
22 F -3.1161 -0.3832 3.3466
23 N 0.6561 -1.2275 -0.7181
24 C 1.1372 -2.2116 -1.4877
25 C 2.5202 -2.0768 -1.6650
26 C 2.8425 -0.9422 -0.9349
27 N 1.7121 -0.4558 -0.3763
28 C 0.2432 -3.2736 -2.0393
29 F -0.6062 -3.7739 -1.1052
30 N -2.4450 -1.2128 -1.4812
31 Cl -8.0159 0.5788 0.2517
32 Cl -7.5011 -1.2838 -1.9586
33 F -3.3428 2.9391 -0.4771
34 F -2.5721 4.0421 -2.2006
35 F -0.5270 -2.8346 -3.0770
36 F 0.9825 -4.3124 -2.5182
37 F -2.3701 -2.2147 2.4121
38 F -2.0324 -1.8477 4.5501
39 C -1.6729 -0.2049 -4.4987
40 C -0.4079 0.2822 -3.8745
41 H -0.1310 4.8133 -1.2391
42 H 2.0373 3.5245 -0.1027
43 H 0.2005 -0.2652 5.0157
44 H 2.1519 0.8356 3.3937
45 H 3.1777 -2.7215 -2.2273
46 H 3.7913 -0.4541 -0.7664
47 H 2.6354 1.1912 0.8598
48 H -5.2033 0.8910 -0.0260
49 H -5.9561 1.1476 -1.6317
50 H -2.4290 -1.3020 -2.4949
51 H -2.5784 0.3908 -4.4196
52 H -1.7004 -1.0868 -5.1309
53 C -0.0196 1.7041 -4.3275
54 H -0.5177 0.2973 -2.7798
55 H 0.4121 -0.4130 -4.0905
56 H 0.8858 2.0450 -3.8099
57 H 0.1689 1.7386 -5.4069
58 H -0.8220 2.4168 -4.1063

tpMECP4-5_singlet

E = -2394.64080273 Hartree

Atom X Y Z
1 N -1.9626 -0.9764 1.4609
2 N -3.3078 -1.0255 1.5794
3 C -3.6511 -1.5220 2.7892
4 C -2.4906 -1.8100 3.4929
5 C -1.4655 -1.4459 2.6106
6 B -4.2288 -0.4459 0.4751
7 N -3.8798 -1.1272 -0.8717
8 N -2.6275 -1.0789 -1.3791
9 C -2.6719 -1.7238 -2.5515
10 C -3.9620 -2.2001 -2.8170
11 C -4.6991 -1.7940 -1.7145
12 Cu -1.1422 -0.1728 -0.2771
13 N 0.5852 0.5474 -0.3792
14 C 1.7279 -0.1857 -0.5286
15 O 2.8489 0.6009 -0.3265
16 C 4.1091 -0.0286 -0.4237
17 C 4.8014 -0.0516 0.9507
18 Cl 3.8479 -1.0028 2.1257
19 C -1.4379 -1.8876 -3.3738
20 F -0.7577 -0.7143 -3.5208
21 C 0.0108 -1.5030 2.8197
22 F 0.6412 -2.2729 1.8933
23 N -2.7384 1.5302 0.0443
24 C -2.8335 2.8619 0.0549
25 C -4.1350 3.2878 0.3714
26 C -4.8283 2.1032 0.5583
27 N -3.9692 1.0732 0.3563
28 C -1.6468 3.6921 -0.2880
29 F -0.5173 3.2939 0.3735
30 F 0.5881 -0.2681 2.7530
31 F 0.3043 -2.0166 4.0452
32 F -0.5612 -2.7773 -2.8346
33 F -1.7468 -2.3372 -4.6200
34 F -1.3329 3.6446 -1.6198
35 F -1.8511 4.9994 0.0186
36 O 1.7847 -1.3648 -0.8252
37 Cl 6.4224 -0.8108 0.7263
38 Cl 5.0157 1.6230 1.5758
39 C 1.1990 1.7409 -2.9886
40 C 2.4026 2.6091 -2.8973
41 H -4.2967 -2.7529 -3.6812
42 H -5.7431 -1.9277 -1.4721
43 H -2.3921 -2.2188 4.4866
44 H -4.6909 -1.6303 3.0607
45 H -4.5022 4.2994 0.4517
46 H -5.8602 1.9195 0.8202
47 H -5.3759 -0.6519 0.7514
48 H 4.0061 -1.0553 -0.7827
49 H 4.7335 0.5535 -1.1068
50 H 0.2094 2.1553 -2.8272
51 H 0.7793 1.5107 -0.1128
52 H 1.2608 0.7200 -3.3502
53 H 2.5447 3.1362 -3.8617
54 C 2.3291 3.6633 -1.7809
55 H 3.3022 1.9912 -2.7720
56 H 3.1997 4.3293 -1.8149
57 H 1.4276 4.2794 -1.8775
58 H 2.3121 3.1857 -0.7961

tpMECP4-5_triplet

E = -2394.64099659 Hartree

Atom X Y Z
1 N -1.9626 -0.9764 1.4609
2 N -3.3078 -1.0255 1.5794
3 C -3.6511 -1.5220 2.7892
4 C -2.4906 -1.8100 3.4929
5 C -1.4655 -1.4459 2.6106
6 B -4.2288 -0.4459 0.4751
7 N -3.8798 -1.1272 -0.8717
8 N -2.6275 -1.0789 -1.3791
9 C -2.6719 -1.7238 -2.5515
10 C -3.9620 -2.2001 -2.8170
11 C -4.6991 -1.7940 -1.7145
12 Cu -1.1422 -0.1728 -0.2771
13 N 0.5852 0.5474 -0.3792
14 C 1.7279 -0.1857 -0.5286
15 O 2.8489 0.6009 -0.3265
16 C 4.1091 -0.0286 -0.4237
17 C 4.8014 -0.0516 0.9507
18 Cl 3.8479 -1.0028 2.1257
19 C -1.4379 -1.8876 -3.3738
20 F -0.7577 -0.7143 -3.5208
21 C 0.0108 -1.5030 2.8197
22 F 0.6412 -2.2729 1.8933
23 N -2.7384 1.5302 0.0443
24 C -2.8335 2.8619 0.0549
25 C -4.1350 3.2878 0.3714
26 C -4.8283 2.1032 0.5583
27 N -3.9692 1.0732 0.3563
28 C -1.6468 3.6921 -0.2880
29 F -0.5173 3.2939 0.3735
30 F 0.5881 -0.2681 2.7530
31 F 0.3043 -2.0166 4.0452
32 F -0.5612 -2.7773 -2.8346
33 F -1.7468 -2.3372 -4.6200
34 F -1.3329 3.6446 -1.6198
35 F -1.8511 4.9994 0.0186
36 O 1.7847 -1.3648 -0.8252
37 Cl 6.4224 -0.8108 0.7263
38 Cl 5.0157 1.6230 1.5758
39 C 1.1990 1.7409 -2.9886
40 C 2.4026 2.6091 -2.8973
41 H -4.2967 -2.7529 -3.6812
42 H -5.7431 -1.9277 -1.4721
43 H -2.3921 -2.2188 4.4866
44 H -4.6909 -1.6303 3.0607
45 H -4.5022 4.2994 0.4517
46 H -5.8602 1.9195 0.8202
47 H -5.3759 -0.6519 0.7514
48 H 4.0061 -1.0553 -0.7827
49 H 4.7335 0.5535 -1.1068
50 H 0.2094 2.1553 -2.8272
51 H 0.7793 1.5107 -0.1128
52 H 1.2608 0.7200 -3.3502
53 H 2.5447 3.1362 -3.8617
54 C 2.3291 3.6633 -1.7809
55 H 3.3022 1.9912 -2.7720
56 H 3.1997 4.3293 -1.8149
57 H 1.4276 4.2794 -1.8775
58 H 2.3121 3.1857 -0.7961

ipMECP4-5_triplet

E = -2394.64914544 Hartree

Atom X Y Z
1 N -1.4383 -1.9563 -0.9560
2 N -2.7279 -2.3136 -1.1469
3 C -2.7964 -3.5723 -1.6329
4 C -1.5058 -4.0626 -1.7684
5 C -0.6977 -3.0081 -1.3262
6 B -3.8878 -1.3791 -0.7186
7 N -3.7122 -0.0132 -1.4286
8 N -2.5840 0.7172 -1.2784
9 C -2.7728 1.8485 -1.9694
10 C -4.0316 1.8593 -2.5826
11 C -4.5955 0.6487 -2.2078
12 Cu -1.0363 -0.0728 -0.1819
13 N 0.5610 0.4409 0.6449
14 C 1.6654 0.8380 -0.0524
15 O 2.7542 0.9996 0.7826
16 C 3.9812 1.3486 0.1759
17 C 4.9655 0.1664 0.2303
18 Cl 4.3193 -1.2374 -0.6677
19 C -1.7056 2.8906 -2.0387
20 F -1.1770 3.1640 -0.8118
21 C 0.7907 -2.9629 -1.2234
22 F 1.3432 -2.0054 -2.0157
23 N -2.7109 -0.6148 1.3611
24 C -2.9306 -0.6029 2.6776
25 C -4.1829 -1.1538 2.9997
26 C -4.7101 -1.5059 1.7676
27 N -3.8104 -1.1701 0.8102
28 C -1.9184 0.0058 3.5840
29 F -0.6492 -0.4357 3.3271
30 F 1.2123 -2.7048 0.0475

31 F 1.3332 -4.1548 -1.5932
32 F -0.6670 2.5314 -2.8382
33 F -2.2053 4.0554 -2.5347
34 F -1.8668 1.3683 3.4730
35 F -2.1839 -0.2724 4.8866
36 O 1.7019 1.0514 -1.2513
37 Cl 6.5076 0.7171 -0.5274
38 Cl 5.2826 -0.3124 1.9359
39 C 0.6168 3.3037 1.9653
40 C 1.4552 4.0306 0.9701
41 H -4.4557 2.6353 -3.2005
42 H -5.5569 0.2131 -2.4368
43 H -1.1897 -5.0294 -2.1283
44 H -3.7532 -4.0260 -1.8446
45 H -4.6248 -1.2773 3.9766
46 H -5.6494 -1.9693 1.5025
47 H -4.9388 -1.8668 -1.0226
48 H 3.8346 1.6301 -0.8691
49 H 4.4191 2.1781 0.7359
50 H -0.4321 3.1377 1.7357
51 H 0.7469 0.3289 1.6400
52 C 1.1292 2.9939 3.3299
53 H 1.3341 5.1271 1.0494
54 H 2.5246 3.8280 1.1179
55 H 1.1904 3.7621 -0.0595
56 H 1.2803 3.9078 3.9348
57 H 0.4460 2.3469 3.8899
58 H 2.1122 2.4991 3.2843

tpTS3-4$_{ON}$_triplet
E = -2394.62647334 Hartree
Atom X Y Z
1 C -0.7807 -0.7419 3.0153
2 N -0.3518 -0.3652 1.8052
3 N 0.8433 0.2365 1.9912
4 C 1.1606 0.2444 3.3052
5 C 0.1389 -0.3772 4.0078
6 Cu -1.0890 -0.5340 -0.1147
7 O -3.1460 -0.0935 0.2290
8 C -3.3122 -0.4793 -0.9659
9 O -4.4299 -0.2078 -1.6677
10 C -5.4267 0.5936 -1.0436
11 C -6.6481 -0.2616 -0.6655
12 Cl -6.1972 -1.5275 0.5156
13 B 1.6427 0.8044 0.7882
14 N 0.8245 1.9382 0.1363
15 N -0.4236 1.7134 -0.3289
16 C -0.8734 2.9034 -0.7385
17 C 0.0818 3.9156 -0.5446
18 C 1.1549 3.2481 0.0204
19 C -2.2593 3.0580 -1.2600
20 F -2.6097 2.0685 -2.1330
21 C -2.0918 -1.4384 3.1876
22 F -3.1471 -0.5812 3.2359
23 N 0.8442 -1.0756 -0.7197
24 C 1.3939 -2.0339 -1.4756
25 C 2.7908 -1.9271 -1.4888
26 C 3.0495 -0.8358 -0.6739
27 N 1.8717 -0.3467 -0.2255
28 C 0.5555 -3.0587 -2.1656
29 F -0.3750 -3.6097 -1.3460
30 N -2.3221 -1.1712 -1.5363
31 Cl -7.8643 0.8507 0.0673
32 Cl -7.3568 -1.0334 -2.1268
33 F -3.2096 3.0399 -0.2773
34 F -2.4062 4.2416 -1.9164
35 F -0.1115 -2.5577 -3.2436
36 F 1.3388 -4.0728 -2.6316
37 F -2.3418 -2.3199 2.1822
38 F -2.1070 -2.1396 4.3570
39 C -2.1088 -0.3923 -4.0129
40 C -2.6281 -1.4980 -4.9039
41 H -0.0061 4.9643 -0.7840
42 H 2.1210 3.6046 0.3468
43 H 0.0675 -0.5545 5.0698
44 H 2.0872 0.6847 3.6431
45 H 3.4947 -2.5615 -2.0047
46 H 3.9818 -0.3789 -0.3761
47 H 2.6994 1.2252 1.1642
48 H -5.0410 1.0756 -0.1443
49 H -5.7423 1.3439 -1.7711
50 H -1.0430 -0.1690 -4.1100
51 H -2.1904 -0.8254 -2.8120
52 H -2.7176 0.5144 -4.0006
53 H -2.5257 -1.1676 -5.9519
54 H -1.9906 -2.3832 -4.7995
55 C -4.0888 -1.8667 -4.6233
56 H -4.4376 -2.6463 -5.3108
57 H -4.2055 -2.2376 -3.5996
58 H -4.7463 -0.9961 -4.7365

tpTS3-4$_{N}$_triplet
E = -2394.62582008 Hartree
Atom X Y Z
1 N 4.0995 -33.0959 4.1595
2 N 2.9595 -32.5354 3.7036
3 C 2.6445 -33.1811 2.5759
4 C 3.5883 -34.1741 2.2821
5 C 4.5003 -34.0814 3.3247
6 Cu 2.0537 -31.0594 4.8525
7 N 0.6440 -29.8652 4.8948
8 C 0.5152 -28.6706 5.5490
9 O -0.6294 -28.0128 5.1597
10 C -0.9606 -26.8199 5.8438
11 C -2.2232 -27.0397 6.6963
12 Cl -1.9368 -28.3140 7.9213
13 C 1.4107 -32.7968 1.8332
14 F 0.2926 -32.8714 2.6196
15 B 4.7576 -32.5869 5.4729
16 N 5.1243 -31.0998 5.2974
17 N 4.1803 -30.1931 4.9575
18 C 4.8285 -29.0378 4.7949
19 C 6.2064 -29.1799 5.0374
20 C 6.3487 -30.5191 5.3544
21 C 4.1123 -27.7898 4.3983
22 F 4.8676 -27.0611 3.5192
23 N 3.7259 -32.7426 6.6232
24 C 3.8612 -33.3996 7.7972
25 C 2.6768 -33.2774 8.5094
26 C 1.8581 -32.5108 7.6698
27 N 2.4964 -32.1900 6.5388
28 C 0.4512 -32.0686 7.8961
29 F 0.3340 -30.7248 8.0751
30 O 1.3157 -28.2212 6.3499
31 F 2.9233 -28.0385 3.7900
32 F 3.8605 -26.9600 5.4485
33 F -0.3622 -32.3853 6.8451
34 F -0.0699 -32.6650 9.0019
35 F 1.4531 -31.5147 1.3695
36 F 1.2061 -33.6069 0.7636
37 Cl -2.6066 -25.4804 7.5127
38 Cl -3.6122 -27.5203 5.6556
39 C -1.6965 -30.8993 4.4321
40 C -2.1171 -30.3078 3.1088
41 H -2.2970 -30.6060 5.2964
42 H -0.5304 -30.3850 4.6894
43 H 6.9714 -28.4207 4.9771
44 H 7.2239 -31.1001 5.6070
45 H 2.4326 -33.6820 9.4794
46 H 4.7836 -33.9070 8.0388
47 H 3.5970 -34.8513 1.4420
48 H 5.3997 -34.6429 3.5307
49 H 5.7363 -33.2328 5.7182
50 H -0.1461 -26.4996 6.4973
51 H -1.1788 -26.0470 5.1028
52 H -1.4734 -31.9692 4.4232
53 H -2.1496 -29.2155 3.1936
54 C -3.4958 -30.8329 2.6683
55 H -4.2681 -30.5772 3.4041
56 H -3.7866 -30.3951 1.7055
57 H -3.4864 -31.9236 2.5553
58 H -1.3698 -30.5527 2.3437

tpInt5$_{N}$_singlet
E = -2394.75930671 Hartree
Atom X Y Z
1 N -1.1704 -1.6870 -0.9986
2 N -2.4471 -2.1304 -1.0148
3 C -2.5234 -3.3378 -1.6249
4 C -1.2500 -3.7062 -2.0268
5 C -0.4487 -2.6317 -1.6100
6 B -3.6086 -1.2732 -0.4482
7 N -3.6827 0.0613 -1.2324
8 N -2.6326 0.9117 -1.2814
9 C -3.0176 1.9203 -2.0695
10 C -4.3283 1.7393 -2.5390
11 C -4.7120 0.5327 -1.9776
12 Cu -0.8973 0.2555 -0.1846
13 N 0.6915 1.3930 0.6156
14 C 1.8757 1.1619 -0.1087
15 O 2.4789 0.0467 0.3916
16 C 3.7119 -0.3431 -0.1911
17 C 4.8551 -0.1322 0.8151
18 Cl 4.5697 -1.0927 2.3063
19 C -2.1033 3.0685 -2.3149
20 F -2.0806 3.9605 -1.2738
21 C 1.0246 -2.4814 -1.7390
22 F 1.3999 -1.2106 -2.0681
23 N -2.2805 -0.3003 1.4704
24 C -2.3786 -0.2419 2.8023
25 C -3.5330 -0.8922 3.2681
26 C -4.1335 -1.3555 2.1088
27 N -3.3649 -0.9872 1.0541
28 C -1.3468 0.4832 3.5880
29 F -0.0701 0.0511 3.3161
30 F 1.6947 -2.7799 -0.5807
31 F 1.5256 -3.3101 -2.6942
32 F -0.8111 2.6782 -2.5010
33 F -2.4805 3.7738 -3.4154
34 F -1.3271 1.8294 3.3295
35 F -1.5376 0.3394 4.9217
36 O 2.2764 1.8286 -1.0311
37 Cl 6.3737 -0.6888 0.0256
38 Cl 5.0034 1.6037 1.2516
39 C 0.2557 2.8176 0.6892
40 C 1.1334 3.6607 1.6134
41 H -4.8949 2.3867 -3.1906
42 H -5.6341 -0.0242 -2.0574
43 H -0.9420 -4.6017 -2.5443
44 H -3.4733 -3.8407 -1.7326
45 H -3.8682 -1.0021 4.2880
46 H -5.0447 -1.9156 1.9579
47 H -4.6412 -1.8698 -0.5776
48 H 3.6500 -1.4061 -0.4240
49 H 3.9185 0.2368 -1.0925
50 H 0.2649 3.2013 -0.3301
51 H 0.7624 0.9699 1.5421
52 H -0.7785 2.8094 1.0393
53 C 0.6253 5.1024 1.6989
54 H 1.1410 3.2070 2.6138
55 H 2.1658 3.6466 1.2420
56 H 1.2602 5.7041 2.3585
57 H 0.6195 5.5786 0.7110

58 H -0.3975 5.1375 2.0936

ipTS3-5$_{ON}$_singlet
E = -2394.63703485 Hartree
Atom X Y Z
1 N 0.8940 0.1787 2.1133
2 N -0.3754 -0.2463 1.9322
3 C -0.8062 -0.6599 3.1288
4 C 0.1858 -0.5003 4.1071
5 C 1.2559 0.0386 3.4092
6 Cu -1.1789 -0.1244 -0.0021
7 O -3.1786 0.0128 0.2726
8 C -3.3559 0.0322 -0.9893
9 O -4.5701 0.1699 -1.5555
10 C -5.6881 0.2303 -0.6751
11 C -6.5103 -1.0676 -0.7580
12 Cl -7.9010 -0.8875 0.3721
13 C -2.1867 -1.2027 3.2968
14 F -2.5035 -2.1176 2.3400
15 B 1.7347 0.7131 0.9260
16 N 1.0720 1.9850 0.3530
17 N -0.1914 1.9455 -0.1191
18 C -0.4520 3.1756 -0.5664
19 C 0.6476 4.0338 -0.3894
20 C 1.6016 3.2247 0.2047
21 C -1.7548 3.4864 -1.2186
22 F -2.8164 2.9024 -0.5969
23 N 0.6511 -0.8815 -0.7215
24 N 1.7815 -0.3810 -0.1755
25 C 2.8684 -0.9742 -0.7213
26 C 2.4384 -1.9001 -1.6586
27 C 1.0399 -1.7993 -1.6125
28 C 0.0460 -2.5538 -2.4292
29 F -0.3996 -1.8458 -3.5101
30 N -2.2486 -0.0652 -1.6919
31 Cl -7.1350 -1.3125 -2.4279
32 Cl -5.5162 -2.4761 -0.2828
33 F 0.6024 -3.6954 -2.9262
34 F -1.0557 -2.9163 -1.7213
35 F -1.9852 4.8305 -1.2313
36 F -1.8054 3.0782 -2.5238
37 F -2.3119 -1.8220 4.5054
38 F -3.1488 -0.2394 3.2513
39 C -2.9040 -0.1241 -4.2037
40 C -3.7426 1.0971 -4.5084
41 H -1.9174 -0.0937 -4.6806
42 H 0.7221 5.0785 -0.6502
43 H 2.6087 3.4377 0.5329
44 H 0.1261 -0.7506 5.1550
45 H 2.2440 0.3260 3.7376
46 H 3.0332 -2.5566 -2.2749
47 H 3.8628 -0.6990 -0.4015
48 H 2.8479 0.9606 1.2951
49 H -5.3694 0.3779 0.3583
50 H -6.3166 1.0616 -1.0007
51 C -3.5866 -1.4653 -4.3571
52 H -2.5405 -0.0513 -3.0507
53 H -3.8554 -1.6273 -5.4119
54 H -2.9320 -2.2850 -4.0469
55 H -4.5089 -1.5086 -3.7693
56 H -3.9941 1.1111 -5.5798
57 H -4.6815 1.0831 -3.9462
58 H -3.2079 2.0227 -4.2749

tpInt5$_{O}$_singlet
E = -2394.75994464 Hartree
Atom X Y Z
1 N -1.9954 -0.6306 1.0710
2 N -0.7630 -0.6440 1.6207
3 C -0.8782 -1.3094 2.7725
4 C -2.1964 -1.7427 2.9875
5 C -2.8755 -1.2838 1.8690
6 Cu 0.7600 0.2955 0.4793
7 O 2.7447 0.1806 0.5303
8 C 3.5763 0.5776 1.3567
9 N 3.3060 0.9332 2.6157
10 C 4.2467 1.5614 3.5466
11 C 4.4328 3.0604 3.2842
12 C 0.3207 -1.4620 3.6381
13 F 0.0413 -2.1730 4.7600
14 B -2.2632 0.1126 -0.2680
15 N -1.8539 1.6015 -0.0965
16 N -0.5871 1.9437 0.2244
17 C -0.5879 3.2722 0.3600
18 C -1.8615 3.8147 0.1201
19 C -2.6394 2.7045 -0.1666
20 C 0.6756 3.9824 0.6973
21 F 0.4270 5.2201 1.2064
22 N -1.4267 -0.5277 -1.4020
23 N -0.0809 -0.5844 -1.3184
24 C 0.3354 -1.1526 -2.4518
25 C -0.7433 -1.4760 -3.2926
26 C -1.8528 -1.0555 -2.5768
27 C 1.7924 -1.3682 -2.6746
28 F 2.0221 -1.9230 -3.8978
29 O 4.8979 0.6498 1.0547
30 C 5.2640 0.4487 -0.3051
31 C 5.6314 1.7916 -0.9607
32 Cl 4.2329 2.9035 -0.9458
33 Cl 7.0116 2.5596 -0.0902
34 Cl 6.1213 1.4389 -2.6554
35 F 1.4241 3.3086 1.6216
36 F 1.4935 4.1674 -0.3841
37 F 2.5166 -0.2101 -2.6223
38 F 2.3559 -2.2006 -1.7503
39 F 1.3623 -2.0787 3.0127
40 F 0.8182 -0.2456 4.0572
41 H -2.1603 4.8511 0.1575
42 H -3.6887 2.6185 -0.4091
43 H -0.7103 -1.9417 -4.2656
44 H -2.9059 -1.0918 -2.8153
45 H -2.5850 -2.2998 3.8262
46 H -3.9144 -1.3722 1.5862
47 H -3.4303 0.0447 -0.5372
48 H 6.1459 -0.1945 -0.3142
49 H 4.4493 -0.0070 -0.8707
50 H 5.2042 1.0377 3.4763
51 H 2.3233 0.9075 2.8585
52 H 3.8524 1.3885 4.5531
53 C 5.4318 3.6873 4.2589
54 H 4.7791 3.1936 2.2528
55 H 3.4591 3.5600 3.3571
56 H 5.5531 4.7578 4.0607
57 H 5.1011 3.5761 5.2996
58 H 6.4200 3.2179 4.1708

Butane amidation ---

iH(CH$_3$)$_3$
E = -158.467606919 Hartree
Atom X Y Z
1 C -3.7017 -2.0621 -3.0463
2 C -2.7460 -3.1137 -3.6263
3 H -3.3074 -1.6313 -2.1175
4 H -3.8519 -1.2384 -3.7575
5 H -4.6859 -2.4936 -2.8251
6 C -3.3269 -3.7380 -4.9023
7 C -1.3613 -2.5082 -3.8934
8 H -2.6284 -3.9139 -2.8800
9 H -2.6631 -4.5118 -5.3079
10 H -4.3044 -4.1993 -4.7137
11 H -3.4637 -2.9756 -5.6813
12 H -0.6631 -3.2603 -4.2813
13 H -1.4262 -1.7007 -4.6354
14 H -0.9259 -2.0853 -2.9795

tbInt4$_{N}$_triplet
E = -2433.96138286 Hartree
Atom X Y Z
1 C 2.6874 -2.9700 2.6182
2 N 2.5249 -2.3697 1.4180
3 N 1.2539 -1.9326 1.2877
4 C 0.6163 -2.2691 2.4145
5 C 1.4788 -2.9289 3.2991
6 B 3.5634 -2.1695 0.2797
7 N 3.8241 -0.6684 0.0550
8 N 2.8364 0.1402 -0.3925
9 C 3.4035 1.3309 -0.5913
10 C 4.7714 1.3125 -0.2630
11 C 4.9957 0.0102 0.1454
12 Cu 0.7619 -0.8985 -0.4388
13 N -0.6630 0.2720 -0.1963
14 C -0.5896 1.4650 0.4591
15 O 0.3688 1.8929 1.0753
16 C 2.6380 2.4819 -1.1559
17 F 1.4294 2.1168 -1.6546
18 C -0.8283 -1.9426 2.5980
19 F -1.5694 -2.2557 1.4946
20 N 1.7850 -2.3115 -1.5103
21 C 1.5338 -3.0098 -2.6233
22 C 2.5574 -3.9299 -2.8796
23 C 3.4466 -3.7370 -1.8304
24 N 2.9607 -2.7660 -1.0261
25 C 0.2864 -2.7368 -3.3932
26 F 0.2879 -1.5025 -3.9736
27 F -1.3490 -2.6350 3.6461
28 F -1.0510 -0.6227 2.8458
29 F 2.4243 3.4800 -0.2559
30 F 3.3334 3.0585 -2.1880
31 F 0.1068 -3.6478 -4.3817
32 F -0.8230 -2.7738 -2.5935
33 O -1.7684 2.1710 0.3013
34 C -1.8735 3.4142 0.9625
35 C -3.0299 3.3677 1.9751
36 Cl -3.1307 4.9865 2.7619
37 Cl -4.5889 3.0203 1.1415
38 Cl -2.7306 2.1079 3.2107
39 C -3.5735 -0.7360 -0.7352
40 C -3.7981 0.2766 -1.8153
41 C -5.2798 0.2596 -2.2647
42 C -2.8592 0.0544 -3.0118
43 H -1.6312 -0.0656 -0.2865
44 H 5.4798 2.1244 -0.3298
45 H 5.8954 -0.4841 0.4823
46 H 1.2466 -3.3209 4.2772
47 H 3.6442 -3.3852 2.8984
48 H 2.6295 -4.6271 -3.7000
49 H 4.3844 -4.2212 -1.6002
50 H 4.5805 -2.7371 0.5583
51 H -0.9487 3.6545 1.4922
52 H -2.0985 4.1896 0.2250
53 H -3.5975 1.2719 -1.3947
54 H -3.9501 -0.5577 0.2696
55 H -3.3117 -1.7613 -0.9909
56 H -3.0514 0.7897 -3.8024
57 H -1.8088 0.1472 -2.7157
58 H -3.0019 -0.9456 -3.4413
59 H -5.9536 0.4441 -1.4196
60 H -5.4607 1.0349 -3.0206
61 H -5.5439 -0.7110 -2.7029

tbTS3-4$_{ON}$_triplet
E = -2433.94642377 Hartree
Atom X Y Z
1 C -0.7422 -0.7627 3.1053
2 N -0.3237 -0.4349 1.8782
3 N 0.9001 0.1203 2.0240
4 C 1.2442 0.1468 3.3311
5 C 0.2123 -0.4130 4.0698
6 Cu -1.0889 -0.6190 -0.0255
7 O -3.1766 -0.1344 0.2432
8 C -3.2860 -0.4913 -0.9658
9 O -4.3696 -0.2070 -1.7153
10 C -5.3906 0.5892 -1.1257
11 C -6.6404 -0.2612 -0.8424
12 Cl -6.2715 -1.5696 0.3202
13 B 1.6998 0.6269 0.7927
14 N 0.9090 1.7775 0.1329
15 N -0.3831 1.6156 -0.2285
16 C -0.8017 2.8246 -0.6165
17 C 0.2206 3.7840 -0.5196
18 C 1.2989 3.0656 -0.0323
19 C -2.2223 3.0633 -0.9960
20 F -2.7170 2.1177 -1.8490
21 C -2.0803 -1.3937 3.3144
22 F -3.0952 -0.4879 3.3361
23 N 0.8124 -1.2923 -0.6010
24 C 1.3098 -2.3052 -1.3201
25 C 2.7071 -2.2290 -1.3952
26 C 3.0211 -1.1001 -0.6540
27 N 1.8721 -0.5611 -0.1879
28 C 0.4216 -3.3435 -1.9240
29 F -0.5448 -3.7621 -1.0686
30 N -2.2737 -1.1752 -1.5060
31 Cl -7.8834 0.8429 -0.1437
32 Cl -7.2741 -0.9775 -2.3658
33 F -3.0671 3.0734 0.0760
34 F -2.3646 4.2677 -1.6154
35 F -0.2054 -2.9149 -3.0557
36 F 1.1529 -4.4386 -2.2800
37 F -2.3773 -2.2988 2.3427
38 F -2.1171 -2.0498 4.5086
39 C -2.0140 -0.5257 -4.0221
40 C -1.0791 0.6449 -4.2538
41 H 0.1682 4.8338 -0.7644
42 H 2.3048 3.3734 0.2132
43 H 0.1554 -0.5599 5.1373
44 H 2.1952 0.5557 3.6395
45 H 3.3772 -2.9079 -1.8993
46 H 3.9741 -0.6515 -0.4149
47 H 2.7773 1.0200 1.1383
48 H -5.0514 1.0400 -0.1920
49 H -5.6578 1.3641 -1.8469
50 H -2.0922 -0.8379 -2.7774
51 H -3.0596 -0.3373 -4.2808
52 H -1.6612 -1.4765 -4.4320
53 C -0.9492 0.8968 -5.7706
54 H -1.5277 1.5373 -3.8032
55 C 0.2979 0.4215 -3.6151
56 H -0.3159 1.7725 -5.9625
57 H -0.4925 0.0339 -6.2718
58 H -1.9263 1.0779 -6.2337
59 H 0.9611 1.2740 -3.8076
60 H 0.2112 0.2995 -2.5324
61 H 0.7770 -0.4788 -4.0194

tbInt4$_{ON}$_oss
E = -2433.9691218 Hartree
Atom X Y Z
1 C -0.7704 -0.7287 3.1796
2 N -0.3522 -0.4264 1.9452
3 N 0.8688 0.1389 2.0784
4 C 1.2108 0.1976 3.3846
5 C 0.1815 -0.3511 4.1354
6 Cu -1.1324 -0.6456 0.0705
7 O -3.1521 -0.1458 0.3713
8 C -3.3090 -0.4720 -0.8502
9 O -4.4476 -0.2124 -1.5274
10 C -5.4774 0.4825 -0.8383
11 C -6.6562 -0.4593 -0.5383
12 Cl -6.1425 -1.8033 0.5248
13 B 1.6727 0.6202 0.8395
14 N 0.9014 1.7666 0.1552
15 N -0.3842 1.6004 -0.2229
16 C -0.7747 2.7856 -0.7000
17 C 0.2606 3.7352 -0.6430
18 C 1.3170 3.0351 -0.0854
19 C -2.1749 3.0025 -1.1618
20 F -2.6131 2.0322 -2.0158
21 C -2.1002 -1.3727 3.4066
22 F -3.1274 -0.4829 3.4149
23 N 0.7458 -1.2915 -0.5408
24 C 1.2153 -2.2882 -1.3008
25 C 2.6109 -2.2246 -1.4028
26 C 2.9525 -1.1193 -0.6374
27 N 1.8204 -0.5821 -0.1312
28 C 0.2967 -3.2911 -1.9177
29 F -0.6338 -3.7513 -1.0421
30 N -2.2976 -1.0560 -1.4490
31 Cl -7.9235 0.5220 0.2883
32 Cl -7.3316 -1.1314 -2.0649
33 F -3.0788 3.0340 -0.1390
34 F -2.2892 4.1904 -1.8196
35 F -0.3843 -2.7972 -2.9922
36 F 1.0020 -4.3661 -2.3675
37 F -2.3862 -2.3040 2.4561
38 F -2.1210 -2.0044 4.6147
39 C -1.9495 -0.4434 -4.5919
40 C -1.0149 0.7258 -4.5508
41 H 0.2305 4.7666 -0.9588
42 H 2.3231 3.3428 0.1597
43 H 0.1253 -0.4745 5.2058
44 H 2.1592 0.6187 3.6841
45 H 3.2631 -2.8946 -1.9413
46 H 3.9148 -0.6847 -0.4100
47 H 2.7577 0.9982 1.1779
48 H -5.1124 0.8985 0.1020
49 H -5.8332 1.2797 -1.4945
50 H -2.2526 -1.0609 -2.4695
51 H -3.0127 -0.2973 -4.7667
52 H -1.5526 -1.4437 -4.7515
53 C -0.5216 1.0623 -5.9796
54 H -1.5661 1.5970 -4.1762
55 C 0.1815 0.4742 -3.6156
56 H 0.1331 1.9437 -5.9646
57 H 0.0478 0.2242 -6.4008
58 H -1.3600 1.2732 -6.6534
59 H 0.8564 1.3396 -3.6056
60 H -0.1483 0.2927 -2.5899
61 H 0.7576 -0.3989 -3.9466

ibInt4$_{N}$_triplet
E = -2433.97492785 Hartree
Atom X Y Z
1 N 0.0165 -0.0406 0.0799
2 N 0.0131 -0.0255 1.4316
3 C 1.2936 0.0587 1.8131
4 C 2.1470 0.1006 0.7039
5 C 1.2845 0.0319 -0.3810
6 Cu -1.7934 -0.1459 2.3939
7 N -2.9503 -0.3281 3.8455
8 C -4.1490 -0.9639 3.7431
9 O -4.7402 -1.0942 4.9881
10 C -5.9669 -1.7924 5.0420
11 C -7.0954 -0.8590 5.5106
12 Cl -7.2965 0.5106 4.3775
13 C 1.6428 0.1643 3.2587
14 F 1.5039 1.4372 3.7366
15 B -1.3051 -0.1996 -0.7258
16 N -1.9245 -1.5652 -0.3792
17 N -2.1000 -1.9303 0.9109
18 C -2.5829 -3.1729 0.8730
19 C -2.7347 -3.6288 -0.4493
20 C -2.3006 -2.5639 -1.2176
21 C -2.8489 -3.9519 2.1179
22 F -2.2000 -5.1602 2.0802
23 N -2.2635 0.9493 -0.3077
24 C -2.8942 1.8537 -1.0896
25 C -3.6299 2.7034 -0.2757
26 C -3.3811 2.2289 1.0182
27 N -2.5622 1.1711 0.9897
28 C -3.8768 2.7692 2.3172
29 F -4.8159 1.9829 2.9092
30 O -4.6476 -1.4032 2.7215
31 F -2.4260 -3.3169 3.2400
32 F -4.1665 -4.2515 2.2917
33 F -2.8647 2.9060 3.2272
34 F -4.4363 3.9962 2.1486
35 F 0.8457 -0.6218 4.0399
36 F 2.9299 -0.2012 3.4839
37 Cl -8.6133 -1.8326 5.5687
38 Cl -6.7471 -0.2183 7.1578
39 C -2.4534 1.3195 6.4238
40 C -3.5683 2.3176 6.4982
41 C -2.5295 0.1041 7.2982
42 C -1.1075 1.7610 5.9322
43 H -2.8917 0.2747 4.6785
44 H -3.0942 -4.5911 -0.7815
45 H -2.2225 -2.4452 -2.2886
46 H -4.2434 3.5430 -0.5637
47 H -2.7708 1.8315 -2.1623
48 H 3.2240 0.1675 0.7007
49 H 1.4825 0.0248 -1.4428
50 H -1.0804 -0.1254 -1.9000
51 H -6.2296 -2.1935 4.0605
52 H -5.8755 -2.6029 5.7703
53 H -0.5467 2.3056 6.7152
54 H -0.4838 0.9076 5.6410
55 H -1.1885 2.4410 5.0755
56 H -2.2330 0.3385 8.3380
57 H -3.5468 -0.3013 7.3363
58 H -1.8574 -0.6903 6.9491
59 H -3.5319 2.8919 7.4435
60 H -3.5213 3.0451 5.6807
61 H -4.5491 1.8271 6.4689

tbInt5$_{N}$_singlet
E = -2434.07856916 Hartree
Atom X Y Z
1 N -1.6311 -1.0546 1.2699
2 N -2.9720 -1.1548 1.4076
3 C -3.2852 -1.9881 2.4291
4 C -2.1076 -2.4565 2.9879
5 C -1.1091 -1.8400 2.2178
6 B -3.9427 -0.4332 0.4379
7 N -3.6978 -0.9479 -1.0025
8 N -2.4900 -0.8366 -1.6006
9 C -2.6228 -1.4198 -2.7964
10 C -3.9225 -1.9130 -2.9915
11 C -4.5743 -1.5888 -1.8134
12 Cu -0.9814 0.1248 -0.3874
13 N 0.8388 1.0665 -0.9447
14 C 1.9381 0.2697 -0.5779

15 O 2.2973 0.5853 0.6989
16 C 3.4135 -0.0959 1.2479
17 C 4.6016 0.8698 1.3929
18 Cl 4.1631 2.2562 2.4489
19 C -1.4787 -1.4525 -3.7471
20 F -1.2770 -0.2514 -4.3767
21 C 0.3646 -1.9418 2.3812
22 F 1.0248 -2.0315 1.1896
23 N -2.5190 1.6340 0.1435
24 C -2.6543 2.9522 0.3200
25 C -3.9347 3.2857 0.7905
26 C -4.5723 2.0600 0.8901
27 N -3.7046 1.0950 0.4974
28 C -1.5338 3.8640 -0.0261
29 F -0.3412 3.4770 0.5359
30 F 0.9051 -0.8594 3.0267
31 F 0.7076 -3.0344 3.1152
32 F -0.2986 -1.7574 -3.1371
33 F -1.6798 -2.3720 -4.7292
34 F -1.2938 3.9221 -1.3741
35 F -1.7726 5.1330 0.3858
36 O 2.4630 -0.5647 -1.2735
37 Cl 5.9516 -0.0528 2.1451
38 Cl 5.1175 1.4914 -0.2103
39 C 0.7439 1.3871 -2.3988
40 C 1.8926 2.2532 -2.9379
41 H -4.3126 -2.4296 -3.8550
42 H -5.5895 -1.7707 -1.4920
43 H -1.9811 -3.1389 3.8143
44 H -4.3169 -2.1891 2.6782
45 H -4.3240 4.2659 1.0193
46 H -5.5725 1.8053 1.2091
47 H -5.0733 -0.6729 0.7589
48 H 3.1325 -0.4465 2.2409
49 H 3.7104 -0.9329 0.6135
50 H -0.2162 1.8920 -2.5336
51 H 0.8004 1.9026 -0.3623
52 H 0.7037 0.4345 -2.9234
53 C 1.6762 2.4668 -4.4423
54 C 2.0151 3.5937 -2.2007
55 H 2.8249 1.6891 -2.8005
56 H 2.8288 4.1918 -2.6271
57 H 1.0891 4.1751 -2.2840
58 H 2.2390 3.4665 -1.1346
59 H 2.5062 3.0345 -4.8782
60 H 1.6018 1.5124 -4.9771
61 H 0.7522 3.0295 -4.6290

tbInt5$_O$_singlet
E = -2434.07922514 Hartree
Atom X Y Z
1 N -1.9302 -0.5517 1.1250
2 N -0.6760 -0.5563 1.6240
3 C -0.7479 -1.1843 2.8004
4 C -2.0588 -1.6001 3.0833
5 C -2.7804 -1.1723 1.9793
6 Cu 0.8050 0.3216 0.3882
7 O 2.7911 0.2828 0.3423
8 C 3.6512 0.6426 1.1579
9 N 3.4007 1.0159 2.4152
10 C 4.3494 1.5020 3.4168
11 C 4.2831 3.0268 3.6200
12 C 0.4841 -1.3196 3.6210
13 F 0.2535 -2.0198 4.7605
14 B -2.2486 0.1367 -0.2330
15 N -1.8258 1.6280 -0.1418
16 N -0.5463 1.9705 0.1204
17 C -0.5303 3.3031 0.2007
18 C -1.8066 3.8483 -0.0172
19 C -2.6040 2.7350 -0.2306
20 C 0.7524 4.0049 0.4775
21 F 0.5397 5.2927 0.8618
22 N -1.4599 -0.5592 -1.3689
23 N -0.1123 -0.6231 -1.3331
24 C 0.2572 -1.2547 -2.4488
25 C -0.8546 -1.6124 -3.2306
26 C -1.9334 -1.1450 -2.4970
27 C 1.7042 -1.4891 -2.7154
28 F 1.8818 -2.1659 -3.8847
29 O 4.9686 0.6555 0.8331
30 C 5.3061 0.3254 -0.5094
31 C 5.6892 1.5876 -1.3022
32 Cl 4.3144 2.7226 -1.3971
33 Cl 7.0945 2.4123 -0.5317
34 Cl 6.1554 1.0468 -2.9560
35 F 1.4718 3.4005 1.4723
36 F 1.5851 4.0545 -0.6062
37 F 2.4233 -0.3316 -2.8148
38 F 2.3136 -2.2211 -1.7363
39 F 1.5050 -1.9365 2.9618
40 F 0.9900 -0.0961 4.0077
41 H -2.0948 4.8884 -0.0125
42 H -3.6617 2.6499 -0.4334
43 H -0.8615 -2.1299 -4.1775
44 H -2.9950 -1.1855 -2.6936
45 H -2.4157 -2.1273 3.9548
46 H -3.8304 -1.2626 1.7420
47 H -3.4257 0.0637 -0.4526
48 H 6.1754 -0.3336 -0.4703
49 H 4.4734 -0.1649 -1.0162
50 H 5.3542 1.2016 3.1109
51 H 2.4176 1.0142 2.6606
52 H 4.1193 0.9842 4.3550
53 C 5.1701 3.4203 4.8082
54 C 4.6813 3.7845 2.3489
55 H 3.2407 3.2810 3.8630
56 H 5.1164 4.4990 4.9932
57 H 4.8679 2.9061 5.7292
58 H 6.2211 3.1697 4.6121
59 H 4.5885 4.8664 2.4988
60 H 5.7217 3.5703 2.0744
61 H 4.0469 3.5137 1.5009

ibMECP4-5_triplet
E = -2433.97273675 Hartree
Atom X Y Z
1 N 2.0595 -0.0406 1.9140
2 N 3.4094 -0.0283 1.8454
3 C 3.9376 -0.1987 3.0778
4 C 2.8995 -0.3236 3.9900
5 C 1.7460 -0.2188 3.2032
6 B 4.1419 0.0850 0.4786
7 N 3.6653 1.3813 -0.2199
8 N 2.3517 1.6622 -0.3770
9 C 2.2925 2.8998 -0.8859
10 C 3.5751 3.4292 -1.0774
11 C 4.4201 2.4215 -0.6388
12 Cu 0.9745 0.2081 0.1952
13 N -0.7245 -0.0709 -0.5294
14 C -1.8164 0.6993 -0.2684
15 O -2.9166 0.2459 -0.9808
16 C -4.1622 0.8384 -0.6873
17 C -5.0572 -0.1529 0.0800
18 Cl -4.3033 -0.6096 1.6394
19 C 0.9862 3.5943 -1.0826
20 F 0.0624 2.8117 -1.7031
21 C 0.3177 -0.2456 3.6390
22 F -0.2636 0.9851 3.6004
23 N 2.5091 -1.4469 -0.6711
24 C 2.5486 -2.6506 -1.2503
25 C 3.8613 -3.1468 -1.3392
26 C 4.6254 -2.1526 -0.7565
27 N 3.7931 -1.1534 -0.3677
28 C 1.3163 -3.3300 -1.7356
29 F 0.1776 -2.7645 -1.2544
30 F -0.4454 -1.0584 2.8558
31 F 0.2146 -0.6972 4.9181
32 F 0.4317 3.9973 0.0922
33 F 1.1499 4.7109 -1.8456
34 F 1.2126 -3.3107 -3.1033
35 F 1.2980 -4.6460 -1.3830
36 O -1.8571 1.6582 0.4815
37 Cl -6.6370 0.6630 0.3811
38 Cl -5.3394 -1.6376 -0.8990
39 C -1.0121 -0.0942 -3.6974
40 C 0.4757 0.0538 -3.6992
41 H 3.8344 4.3998 -1.4707
42 H 5.4979 2.3679 -0.5925
43 H 2.9603 -0.4707 5.0572
44 H 5.0083 -0.2223 3.2176
45 H 4.1863 -4.0880 -1.7560
46 H 5.6898 -2.0848 -0.5838
47 H 5.3256 0.1390 0.6569
48 H -4.0356 1.7376 -0.0796
49 H -4.6573 1.0832 -1.6307
50 H -0.9117 -0.7709 -1.2448
51 C -1.8426 1.1358 -3.8743
52 C -1.6534 -1.4272 -3.9116
53 H 0.8469 0.3823 -4.6887
54 H 0.8036 0.8174 -2.9823
55 H 0.9875 -0.8829 -3.4625
56 H -1.8861 -1.6005 -4.9798
57 H -1.0142 -2.2553 -3.5915
58 H -2.6104 -1.4988 -3.3766
59 H -1.7984 1.4986 -4.9189
60 H -2.8974 0.9521 -3.6435
61 H -1.4903 1.9624 -3.2471

tbMECP4-5_singlet
E = -2433.95921359 Hartree
Atom X Y Z
1 N -1.3896 -1.8269 -0.9198
2 N -2.6829 -2.1897 -1.0641
3 C -2.7631 -3.4587 -1.5218
4 C -1.4756 -3.9500 -1.6846
5 C -0.6573 -2.8837 -1.2917
6 B -3.8295 -1.2423 -0.6232
7 N -3.6714 0.1080 -1.3640
8 N -2.5410 0.8421 -1.2541
9 C -2.7350 1.9440 -1.9898
10 C -4.0011 1.9337 -2.5888
11 C -4.5627 0.7406 -2.1589
12 Cu -0.9691 0.0712 -0.1708
13 N 0.7137 0.4174 0.5795
14 C 1.8029 0.7635 -0.1644
15 O 2.9585 0.7309 0.5945
16 C 4.1671 1.0739 -0.0544
17 C 5.1592 -0.0981 0.0289
18 Cl 4.5123 -1.5386 -0.8110
19 C -1.6598 2.9711 -2.1198
20 F -1.0916 3.2732 -0.9169
21 C 0.8328 -2.8269 -1.2340
22 F 1.3415 -1.7955 -1.9574
23 N -2.6121 -0.4379 1.4279
24 C -2.8010 -0.4418 2.7498
25 C -4.0372 -1.0122 3.0973
26 C -4.5893 -1.3618 1.8762
27 N -3.7180 -1.0063 0.8997
28 C -1.7824 0.1597 3.6538
29 F -0.5055 -0.1883 3.3106
30 F 1.2971 -2.6741 0.0403
31 F 1.3786 -3.9743 -1.7208

32 F -0.6484 2.5761 -2.9370
33 F -2.1585 4.1284 -2.6334
34 F -1.8117 1.5259 3.6522
35 F -1.9737 -0.2312 4.9420
36 O 1.7838 1.1019 -1.3359
37 Cl 6.6902 0.4335 -0.7659
38 Cl 5.5050 -0.5147 1.7453
39 C 0.2852 3.0251 1.8796
40 C 1.3132 3.8627 1.1994
41 H -4.4330 2.6836 -3.2335
42 H -5.5280 0.2986 -2.3582
43 H -1.1677 -4.9232 -2.0344
44 H -3.7249 -3.9182 -1.6962
45 H -4.4511 -1.1467 4.0850
46 H -5.5277 -1.8379 1.6320
47 H -4.8888 -1.7342 -0.8909
48 H 3.9899 1.3150 -1.1048
49 H 4.6120 1.9310 0.4583
50 H -0.7089 2.9231 1.4597
51 H 0.9536 0.1643 1.5362
52 H 0.4701 2.5790 2.8516
53 C 0.8071 5.3146 1.0238
54 C 2.6545 3.8264 1.9455
55 H 1.4645 3.4669 0.1800
56 H 1.5451 5.9187 0.4811
57 H -0.1322 5.3373 0.4611
58 H 0.6322 5.7841 1.9991
59 H 3.4220 4.3997 1.4125
60 H 2.5508 4.2600 2.9482
61 H 3.0095 2.7969 2.0616

ibMECP4-5_singlet
E = -2433.97278832 Hartree
Atom X Y Z
1 N 2.0595 -0.0406 1.9140
2 N 3.4094 -0.0283 1.8454
3 C 3.9376 -0.1987 3.0778
4 C 2.8995 -0.3236 3.9900
5 C 1.7460 -0.2188 3.2032
6 B 4.1419 0.0850 0.4786
7 N 3.6653 1.3813 -0.2199
8 N 2.3517 1.6622 -0.3770
9 C 2.2925 2.8998 -0.8859
10 C 3.5751 3.4292 -1.0774
11 C 4.4201 2.4215 -0.6388
12 Cu 0.9745 0.2081 0.1952
13 N -0.7245 -0.0709 -0.5294
14 C -1.8164 0.6993 -0.2684
15 O -2.9166 0.2459 -0.9808
16 C -4.1622 0.8384 -0.6873
17 C -5.0572 -0.1529 0.0800
18 Cl -4.3033 -0.6096 1.6394
19 C 0.9862 3.5943 -1.0826
20 F 0.0624 2.8117 -1.7031
21 C 0.3177 -0.2456 3.6390
22 F -0.2636 0.9851 3.6004
23 N 2.5091 -1.4469 -0.6711
24 C 2.5486 -2.6506 -1.2503
25 C 3.8613 -3.1468 -1.3392
26 C 4.6254 -2.1526 -0.7565
27 N 3.7931 -1.1534 -0.3677
28 C 1.3163 -3.3300 -1.7356
29 F 0.1776 -2.7645 -1.2544
30 F -0.4454 -1.0584 2.8558
31 F 0.2146 -0.6972 4.9181
32 F 0.4317 3.9973 0.0922
33 F 1.1499 4.7109 -1.8456
34 F 1.2126 -3.3107 -3.1033
35 F 1.2980 -4.6460 -1.3830
36 O -1.8571 1.6582 0.4815
37 Cl -6.6370 0.6630 0.3811
38 Cl -5.3394 -1.6376 -0.8990
39 C -1.0121 -0.0942 -3.6974
40 C 0.4757 0.0538 -3.6992
41 H 3.8344 4.3998 -1.4707
42 H 5.4979 2.3679 -0.5925
43 H 2.9603 -0.4707 5.0572
44 H 5.0083 -0.2223 3.2176
45 H 4.1863 -4.0880 -1.7560
46 H 5.6898 -2.0848 -0.5838
47 H 5.3256 0.1390 0.6569
48 H -4.0356 1.7376 -0.0796
49 H -4.6573 1.0832 -1.6307
50 H -0.9117 -0.7709 -1.2448
51 C -1.8426 1.1358 -3.8743
52 C -1.6534 -1.4272 -3.9116
53 H 0.8469 0.3823 -4.6887
54 H 0.8036 0.8174 -2.9823
55 H 0.9875 -0.8829 -3.4625
56 H -1.8861 -1.6005 -4.9798
57 H -1.0142 -2.2553 -3.5915
58 H -2.6104 -1.4988 -3.3766
59 H -1.7984 1.4986 -4.9189
60 H -2.8974 0.9521 -3.6435
61 H -1.4903 1.9624 -3.2471

tbMECP4-5_triplet
E = -2433.95939602 Hartree
Atom X Y Z
1 N -1.3896 -1.8269 -0.9198
2 N -2.6829 -2.1897 -1.0641
3 C -2.7631 -3.4587 -1.5218
4 C -1.4756 -3.9500 -1.6846
5 C -0.6573 -2.8837 -1.2917
6 B -3.8295 -1.2423 -0.6232
7 N -3.6714 0.1080 -1.3640
8 N -2.5410 0.8421 -1.2541
9 C -2.7350 1.9440 -1.9898
10 C -4.0011 1.9337 -2.5888
11 C -4.5627 0.7406 -2.1589
12 Cu -0.9691 0.0712 -0.1708
13 N 0.7137 0.4174 0.5795
14 C 1.8029 0.7635 -0.1644
15 O 2.9585 0.7309 0.5945
16 C 4.1671 1.0739 -0.0544
17 C 5.1592 -0.0981 0.0289
18 Cl 4.5123 -1.5386 -0.8110
19 C -1.6598 2.9711 -2.1198
20 F -1.0916 3.2732 -0.9169
21 C 0.8328 -2.8269 -1.2340
22 F 1.3415 -1.7955 -1.9574
23 N -2.6121 -0.4379 1.4279
24 C -2.8010 -0.4418 2.7498
25 C -4.0372 -1.0122 3.0973
26 C -4.5893 -1.3618 1.8762
27 N -3.7180 -1.0063 0.8997
28 C -1.7824 0.1597 3.6538
29 F -0.5055 -0.1883 3.3106
30 F 1.2971 -2.6741 0.0403
31 F 1.3786 -3.9743 -1.7208
32 F -0.6484 2.5761 -2.9370
33 F -2.1585 4.1284 -2.6334
34 F -1.8117 1.5259 3.6522
35 F -1.9737 -0.2312 4.9420
36 O 1.7838 1.1019 -1.3359
37 Cl 6.6902 0.4335 -0.7659
38 Cl 5.5050 -0.5147 1.7453
39 C 0.2852 3.0251 1.8796
40 C 1.3132 3.8627 1.1994
41 H -4.4330 2.6836 -3.2335
42 H -5.5280 0.2986 -2.3582
43 H -1.1677 -4.9232 -2.0344
44 H -3.7249 -3.9182 -1.6962
45 H -4.4511 -1.1467 4.0850
46 H -5.5277 -1.8379 1.6320
47 H -4.8888 -1.7342 -0.8909
48 H 3.9899 1.3150 -1.1048
49 H 4.6120 1.9310 0.4583
50 H -0.7089 2.9231 1.4597
51 H 0.9536 0.1643 1.5362
52 H 0.4701 2.5790 2.8516
53 C 0.8071 5.3146 1.0238
54 C 2.6545 3.8264 1.9455
55 H 1.4645 3.4669 0.1800
56 H 1.5451 5.9187 0.4811
57 H -0.1322 5.3373 0.4611
58 H 0.6322 5.7841 1.9991
59 H 3.4220 4.3997 1.4125
60 H 2.5508 4.2600 2.9482
61 H 3.0095 2.7969 2.0616

ibInt5$_O$_singlet
E = -2434.08121344 Hartree
Atom X Y Z
1 N -1.9625 -0.5888 1.0920
2 N -0.7219 -0.6297 1.6215
3 C -0.8289 -1.3141 2.7629
4 C -2.1500 -1.7318 2.9914
5 C -2.8398 -1.2434 1.8922
6 Cu 0.7989 0.3066 0.4617
7 O 2.7790 0.1347 0.5182
8 C 3.6180 0.5826 1.3140
9 N 3.3362 1.0150 2.5452
10 C 4.2124 1.6718 3.5552
11 C 5.3105 0.6996 4.0149
12 C 0.3806 -1.5018 3.6065
13 F 0.1058 -2.2265 4.7213
14 B -2.2373 0.1700 -0.2362
15 N -1.8009 1.6508 -0.0614
16 N -0.5244 1.9730 0.2426
17 C -0.5034 3.3016 0.3794
18 C -1.7717 3.8635 0.1579
19 C -2.5703 2.7657 -0.1194
20 C 0.7743 3.9945 0.6982
21 F 0.5503 5.2482 1.1784
22 N -1.4287 -0.4775 -1.3864
23 N -0.0829 -0.5561 -1.3236
24 C 0.3064 -1.1338 -2.4617
25 C -0.7903 -1.4417 -3.2848
26 C -1.8816 -1.0013 -2.5529
27 C 1.7558 -1.3774 -2.7044
28 F 1.9601 -1.9186 -3.9381
29 O 4.9343 0.6339 0.9853
30 C 5.2716 0.3432 -0.3670
31 C 5.6359 1.6359 -1.1177
32 Cl 4.2598 2.7745 -1.1163
33 Cl 7.0657 2.4267 -0.3528
34 Cl 6.0526 1.1701 -2.8049
35 F 1.5121 3.3289 1.6377
36 F 1.5939 4.1373 -0.3879
37 F 2.5053 -0.2366 -2.6417
38 F 2.3111 -2.2364 -1.7990
39 F 1.4012 -2.1272 2.9556
40 F 0.9058 -0.3021 4.0368
41 H -2.0538 4.9043 0.2004
42 H -3.6238 2.6960 -0.3478
43 H -0.7798 -1.9097 -4.2571
44 H -2.9386 -1.0210 -2.7751
45 H -2.5335 -2.2980 3.8264
46 H -3.8840 -1.3120 1.6242
47 H -3.4091 0.1240 -0.4889
48 H 6.1472 -0.3084 -0.3528
49 H 4.4411 -0.1391 -0.8844

50 H 2.3527 0.9348 2.7747
51 C 3.2860 2.0109 4.7335
52 C 4.8132 2.9629 2.9758
53 H 5.9065 1.1625 4.8093
54 H 4.8679 -0.2225 4.4078
55 H 5.9814 0.4404 3.1921
56 H 3.8603 2.4990 5.5267
57 H 2.4885 2.6956 4.4211
58 H 2.8267 1.1072 5.1495
59 H 5.3815 3.4864 3.7529
60 H 5.4893 2.7512 2.1449
61 H 4.0200 3.6273 2.6177

ibTS3-4$_{N}$_triplet
E = -2433.95484148 Hartree
Atom X Y Z
1 C 2.8390 -33.3343 2.4925
2 N 2.9539 -32.6879 3.6584
3 N 4.1267 -33.0879 4.1986
4 C 4.7389 -33.9746 3.3817
5 C 3.9410 -34.1689 2.2642
6 Cu 1.8341 -31.2883 4.7118
7 N 3.9085 -30.1730 4.8275
8 N 4.8948 -30.9643 5.3038
9 C 6.0416 -30.2586 5.4735
10 C 5.8024 -28.9516 5.0896
11 C 4.4524 -28.9614 4.6928
12 B 4.6578 -32.4720 5.5218
13 N 3.5986 -32.6897 6.6338
14 N 2.3152 -32.3000 6.4740
15 C 1.6849 -32.6084 7.6135
16 C 2.5605 -33.2028 8.5312
17 C 3.7738 -33.2323 7.8598
18 C 0.2252 -32.3516 7.7680
19 F -0.2362 -32.8672 8.9379
20 C 3.6779 -27.8055 4.1559
21 F 3.3448 -26.8883 5.1112
22 C 1.6207 -33.1628 1.6525
23 F 1.8626 -33.4960 0.3580
24 N 0.4998 -30.0081 4.8265
25 C 0.5743 -28.8972 5.6202
26 O -0.4338 -28.0031 5.3455
27 C -0.4026 -26.7716 6.0458
28 C -1.5810 -26.6774 7.0295
29 Cl -3.1494 -26.7621 6.1482
30 O 1.4519 -28.6907 6.4432
31 Cl -1.5087 -27.9990 8.2333
32 Cl -1.4602 -25.0835 7.8630
33 F -0.0999 -31.0304 7.7583
34 F -0.5019 -32.9270 6.7606
35 F 4.4122 -27.1180 3.2270
36 F 2.5213 -28.1791 3.5491
37 F 0.5813 -33.9472 2.0710
38 F 1.1573 -31.8799 1.6684
39 C -1.9843 -30.7291 4.2993
40 C -1.7410 -32.1078 3.7099
41 C -2.8153 -30.7171 5.5689
42 C -2.3885 -29.6804 3.2772
43 H -0.8347 -30.3717 4.6301
44 H 6.4899 -28.1193 5.0815
45 H 6.9360 -30.7387 5.8431
46 H 2.3336 -33.5595 9.5238
47 H 4.7424 -33.6017 8.1630
48 H 4.1228 -34.8071 1.4132
49 H 5.6961 -34.3970 3.6501
50 H 5.6816 -33.0093 5.8369
51 H 0.5284 -26.6643 6.6058
52 H -0.5028 -25.9640 5.3165
53 H -2.6930 -32.5485 3.3766
54 H -1.0786 -32.0569 2.8409
55 H -1.3023 -32.7892 4.4466
56 H -3.3726 -29.9327 2.8530
57 H -2.4587 -28.6883 3.7326
58 H -1.6704 -29.6331 2.4504
59 H -3.8593 -30.9734 5.3298
60 H -2.4518 -31.4469 6.2975
61 H -2.8170 -29.7277 6.0357

ibTS3-4$_{ON}$_triplet
E = -2433.95172595 Hartree
Atom X Y Z
1 Cu -1.6398 -0.2099 0.0944
2 C 1.2190 -1.3797 -0.7435
3 N 0.4129 -0.4144 -0.2876
4 N 1.2058 0.6362 0.0144
5 C 2.4969 0.3428 -0.2641
6 C 2.5540 -0.9500 -0.7610
7 N -2.3597 -1.1668 -1.4729
8 C -3.6094 -0.9051 -1.0737
9 O -4.6141 -1.0052 -1.9678
10 C -5.9286 -0.6809 -1.5318
11 C -6.8023 -1.9455 -1.5060
12 Cl -6.1463 -3.1422 -0.3479
13 B 0.6403 1.8672 0.7652
14 N -0.3829 2.6425 -0.0951
15 N -1.5977 2.1199 -0.3838
16 C -2.2936 3.1190 -0.9374
17 C -1.5317 4.2974 -1.0270
18 C -0.3153 3.9440 -0.4715
19 C -3.7464 2.9890 -1.2435
20 F -4.1097 1.7388 -1.6355
21 C 0.6934 -2.7231 -1.1350
22 F -0.2855 -3.1610 -0.3048
23 N -1.1012 0.4675 1.9670
24 N -0.0847 1.3568 2.0435
25 C 0.1441 1.6880 3.3342
26 C -0.7491 0.9906 4.1342
27 C -1.5018 0.2393 3.2220
28 C -2.6300 -0.6997 3.4994
29 F -3.8498 -0.1429 3.2655
30 O -3.7880 -0.5702 0.1318
31 Cl -8.4547 -1.4403 -0.9918
32 Cl -6.8920 -2.6849 -3.1431
33 F -2.5575 -1.8251 2.7378
34 F -2.6194 -1.0883 4.8051
35 F -4.5320 3.2916 -0.1672
36 F -4.1162 3.8451 -2.2392
37 F 0.1899 -2.7567 -2.4008
38 F 1.6993 -3.6454 -1.1011
39 C -1.7644 -0.1767 -3.8383
40 C -2.7550 0.8974 -4.2539
41 C -0.3501 0.3469 -3.6371
42 C -1.8532 -1.4372 -4.6850
43 H -1.8353 5.2530 -1.4265
44 H 0.5844 4.5191 -0.3077
45 H -0.8422 1.0090 5.2090
46 H 0.9253 2.3887 3.5898
47 H 3.4222 -1.5111 -1.0705
48 H 3.2776 1.0650 -0.0757
49 H 1.5420 2.5940 1.0716
50 H -5.9168 -0.2392 -0.5344
51 H -6.3536 0.0200 -2.2529
52 H -2.0947 -0.5870 -2.7202
53 H -3.7892 0.5508 -4.1604
54 H -2.6404 1.8095 -3.6625
55 H -2.5853 1.1643 -5.3084
56 H -2.8837 -1.8069 -4.7361
57 H -1.5212 -1.2244 -5.7128
58 H -1.2219 -2.2343 -4.2825
59 H 0.0256 0.7829 -4.5755
60 H -0.3209 1.1298 -2.8702
61 H 0.3344 -0.4521 -3.3412

ibInt4$_{ON}$_triplet
E = -2433.97990162 Hartree
Atom X Y Z
1 N 2.2020 1.6399 2.7590
2 N 1.2644 0.7061 2.4858
3 C 0.7486 0.3266 3.6609
4 C 1.3476 1.0256 4.7169
5 C 2.2696 1.8523 4.0919
6 Cu 0.9532 0.1441 0.5386
7 O -1.1362 0.0291 0.5200
8 C -0.9991 -0.5032 -0.6300
9 O -2.0456 -0.7093 -1.4574
10 C -3.3261 -0.2722 -1.0263
11 C -4.2220 -1.4787 -0.6987
12 Cl -5.8356 -0.8300 -0.2217
13 C -0.3259 -0.7103 3.7370
14 F -0.1393 -1.7087 2.8312
15 B 3.0203 2.3068 1.6197
16 N 2.0575 3.1000 0.7149
17 N 1.0189 2.4823 0.1124
18 C 0.3324 3.4498 -0.5015
19 C 0.9222 4.7109 -0.3047
20 C 2.0265 4.4363 0.4834
21 C -0.9357 3.1501 -1.2237
22 F -2.0007 2.9670 -0.3881
23 N 3.0169 0.1375 0.3042
24 N 3.7149 1.1794 0.8092
25 C 5.0326 1.0237 0.5525
26 C 5.2089 -0.1636 -0.1426
27 C 3.9122 -0.6787 -0.2652
28 C 3.4933 -1.9652 -0.8963
29 F 2.8285 -1.7883 -2.0745
30 N 0.2168 -0.8238 -1.0040
31 Cl -4.4125 -2.5338 -2.1442
32 Cl -3.5317 -2.4349 0.6468
33 F 4.5819 -2.7337 -1.1758
34 F 2.6752 -2.6940 -0.0931
35 F -1.2730 4.1716 -2.0598
36 F -0.8587 2.0197 -1.9840
37 F -0.3433 -1.2829 4.9746
38 F -1.5702 -0.2065 3.5252
39 C 0.5139 -0.6001 -4.2654
40 C 1.0211 -1.9073 -4.7939
41 C -0.9185 -0.2458 -4.5303
42 C 1.4901 0.5150 -4.0338
43 H 0.5887 5.6643 -0.6848
44 H 2.7860 5.0861 0.8934
45 H 1.1422 0.9280 5.7718
46 H 2.9690 2.5684 4.4977
47 H 6.1281 -0.6037 -0.4967
48 H 5.7471 1.7606 0.8888
49 H 3.8479 3.0369 2.0852
50 H -3.2508 0.3616 -0.1415
51 H -3.7807 0.2796 -1.8519
52 H 0.4031 -0.9864 -1.9927
53 H 1.0913 1.2600 -3.3352
54 H 2.4424 0.1459 -3.6367
55 H 1.7196 1.0521 -4.9742
56 H -1.2544 0.5921 -3.9112
57 H -1.0642 0.0537 -5.5861
58 H -1.5885 -1.0953 -4.3484
59 H 1.1773 -1.8649 -5.8893
60 H 1.9811 -2.1875 -4.3467
61 H 0.3070 -2.7216 -4.6145

tbInt4$_{ON}$_triplet
E = -2433.96912181 Hartree
Atom X Y Z
1 C -0.7527 -0.7141 3.1803
2 N -0.3564 -0.4214 1.9364

3 N 0.8639 0.1508 2.0438
4 C 1.2272 0.2234 3.3435
5 C 0.2131 -0.3232 4.1163
6 Cu -1.1634 -0.6664 0.0795
7 O -3.1839 -0.1655 0.4126
8 C -3.3589 -0.5104 -0.7999
9 O -4.5033 -0.2547 -1.4676
10 C -5.5242 0.4526 -0.7771
11 C -6.7072 -0.4796 -0.4651
12 Cl -6.1946 -1.8203 0.6033
13 B 1.6450 0.6220 0.7871
14 N 0.8552 1.7549 0.1007
15 N -0.4348 1.5757 -0.2559
16 C -0.8371 2.7489 -0.7526
17 C 0.1946 3.7036 -0.7297
18 C 1.2618 3.0197 -0.1722
19 C -2.2417 2.9449 -1.2106
20 F -2.6694 1.9621 -2.0557
21 C -2.0754 -1.3620 3.4363
22 F -3.1064 -0.4767 3.4548
23 N 0.7067 -1.3112 -0.5547
24 C 1.1672 -2.3066 -1.3219
25 C 2.5595 -2.2316 -1.4552
26 C 2.9088 -1.1204 -0.7017
27 N 1.7840 -0.5907 -0.1722
28 C 0.2422 -3.3140 -1.9219
29 F -0.6633 -3.7868 -1.0271
30 N -2.3596 -1.1135 -1.4042
31 Cl -7.9631 0.5145 0.3634
32 Cl -7.3958 -1.1568 -1.9831
33 F -3.1429 2.9721 -0.1850
34 F -2.3731 4.1258 -1.8772
35 F -0.4681 -2.8188 -2.9769
36 F 0.9452 -4.3803 -2.3949
37 F -2.3735 -2.3030 2.4991
38 F -2.0718 -1.9829 4.6501
39 C -1.8751 -0.3097 -4.5916
40 C -0.8745 0.8021 -4.5254
41 H 0.1558 4.7279 -1.0670
42 H 2.2697 3.3368 0.0525
43 H 0.1751 -0.4368 5.1886
44 H 2.1785 0.6516 3.6231
45 H 3.2050 -2.8977 -2.0064
46 H 3.8723 -0.6764 -0.4984
47 H 2.7329 1.0107 1.1030
48 H -5.1505 0.8716 0.1583
49 H -5.8777 1.2484 -1.4362
50 H -2.3256 -1.1173 -2.4217
51 H -2.9306 -0.1034 -4.7500
52 H -1.5350 -1.3305 -4.7533
53 C -0.3226 1.1182 -5.9365
54 H -1.3818 1.7027 -4.1575
55 C 0.2769 0.4702 -3.5596
56 H 0.3864 1.9559 -5.8972
57 H 0.2033 0.2487 -6.3507
58 H -1.1278 1.3867 -6.6299
59 H 0.9974 1.2967 -3.5090
60 H -0.0990 0.2854 -2.5508
61 H 0.8156 -0.4267 -3.8901

ibInt5$_{N}$_singlet
E = -2434.08096968 Hartree
Atom X Y Z
1 N 0.9223 0.6805 1.3251
2 N 2.0307 0.1307 1.8688
3 C 2.0383 0.3001 3.2150
4 C 0.8941 0.9907 3.5730
5 C 0.2420 1.2093 2.3481
6 B 3.1942 -0.3917 0.9922
7 N 3.6604 0.7517 0.0495
8 N 2.8291 1.3317 -0.8478
9 C 3.5503 2.2858 -1.4466
10 C 4.8613 2.3342 -0.9486
11 C 4.8831 1.3337 0.0088
12 Cu 0.8464 0.5423 -0.8434
13 N -1.0081 0.0848 -1.8673
14 C -1.9417 -0.2189 -0.8571
15 O -1.5906 -1.4008 -0.2846
16 C -2.3818 -1.8001 0.8286
17 C -1.6768 -2.9837 1.4980
18 Cl -0.0444 -2.5139 2.0609
19 C 2.9537 3.1165 -2.5269
20 F 2.8208 2.4375 -3.7086
21 C -1.0412 1.9325 2.1329
22 F -1.1542 2.4286 0.8751
23 N 1.8946 -1.5104 -0.8679
24 C 1.8949 -2.7067 -1.4669
25 C 2.7715 -3.6067 -0.8376
26 C 3.3176 -2.8603 0.1920
27 N 2.7781 -1.6158 0.1465
28 C 1.1017 -2.9360 -2.7027
29 F -0.2551 -2.7937 -2.5222
30 F -2.1403 1.1399 2.3456
31 F -1.1675 2.9782 2.9987
32 F 1.7043 3.5688 -2.2147
33 F 3.7237 4.2058 -2.7913
34 F 1.4161 -2.0535 -3.7007
35 F 1.2955 -4.1851 -3.1941
36 O -2.8922 0.4442 -0.5152
37 Cl -2.6876 -3.4644 2.9124
38 Cl -1.5433 -4.3729 0.3674
39 C -1.4076 1.0447 -2.9685
40 C -0.2731 0.9691 -3.9981
41 H 5.6572 3.0010 -1.2423
42 H 5.6733 0.9977 0.6640
43 H 0.5802 1.3053 4.5567
44 H 2.8607 -0.0673 3.8113
45 H 2.9747 -4.6320 -1.1056
46 H 4.0524 -3.1161 0.9411
47 H 4.1045 -0.7055 1.7079
48 H -2.4758 -0.9867 1.5496
49 H -3.3769 -2.1179 0.5035
50 H -0.6819 -0.7928 -2.2730
51 C -1.5139 2.4596 -2.3910
52 C -2.7293 0.5954 -3.6106
53 H -0.4691 1.6675 -4.8176
54 H 0.6883 1.2361 -3.5494
55 H -0.1827 -0.0373 -4.4237
56 H -2.9650 1.2435 -4.4615
57 H -2.6521 -0.4336 -3.9832
58 H -3.5582 0.6513 -2.9008
59 H -1.7313 3.1670 -3.1987
60 H -2.3056 2.5249 -1.6433
61 H -0.5684 2.7554 -1.9259

tbTS3-5$_{ON}$_singlet
E = -2433.94927616 Hartree
Atom X Y Z
1 N 5.5114 -2.9178 -8.3032
2 N 4.1663 -2.9687 -8.3839
3 C 3.8873 -3.9371 -9.2589
4 C 5.0588 -4.5234 -9.7699
5 C 6.0753 -3.8383 -9.1250
6 Cu 3.0694 -1.4440 -7.1220
7 N 1.7256 -0.8306 -8.4665
8 C 0.6708 -1.0249 -7.6887
9 O -0.5676 -0.6823 -8.1383
10 C -1.6773 -1.0864 -7.3520
11 C -2.4998 -2.1385 -8.1158
12 Cl -3.0997 -1.4580 -9.6749
13 C 2.4737 -4.2795 -9.5804
14 F 2.3772 -5.5546 -10.0548
15 B 6.2068 -1.9651 -7.3020
16 N 5.9153 -0.4889 -7.6734
17 N 4.6565 -0.0062 -7.6453
18 C 4.7520 1.2945 -7.9338
19 C 6.0857 1.6713 -8.1649
20 C 6.7950 0.4947 -7.9835
21 C 3.5319 2.1469 -7.9899
22 F 3.8570 3.4669 -7.8919
23 N 4.3435 -2.0804 -5.5794
24 C 4.2175 -2.3873 -4.2848
25 C 5.4496 -2.7537 -3.7237
26 C 6.3384 -2.6432 -4.7815
27 N 5.6537 -2.2395 -5.8774
28 C 2.8816 -2.3190 -3.6242
29 F 2.9951 -2.5609 -2.2864
30 O 0.8881 -1.4924 -6.5457
31 Cl -1.5051 -3.5858 -8.4482
32 Cl -3.9079 -2.5789 -7.0847
33 F 2.8387 2.0150 -9.1669
34 F 2.6458 1.8712 -6.9973
35 F 2.2933 -1.0983 -3.7617
36 F 2.0009 -3.2329 -4.1179
37 F 1.6522 -4.1811 -8.5047
38 F 1.9274 -3.4780 -10.5529
39 C 3.3457 -0.6458 -11.4100
40 C 2.0438 0.1517 -11.5867
41 C 0.9187 -0.3625 -10.7423
42 H 6.4648 2.6516 -8.4103
43 H 7.8537 0.2882 -8.0435
44 H 5.6508 -3.0482 -2.7054
45 H 7.4027 -2.8205 -4.8336
46 H 5.1413 -5.3307 -10.4816
47 H 7.1489 -3.9444 -9.1805
48 H 7.3908 -2.1555 -7.3186
49 H -1.3512 -1.5145 -6.4014
50 H -2.3104 -0.2136 -7.1757
51 H 0.6698 -1.4182 -10.8243
52 H 0.0685 0.2900 -10.5480
53 H 1.5337 -0.3167 -9.4393
54 C 1.5538 0.0946 -13.0606
55 H 2.2264 1.2010 -11.3324
56 H 4.1302 -0.2262 -12.0499
57 H 3.6955 -0.6146 -10.3733
58 H 3.2021 -1.6955 -11.6845
59 H 0.6385 0.6776 -13.2094
60 H 2.3332 0.5088 -13.7120
61 H 1.3619 -0.9394 -13.3685

ibTS3-5$_{ON}$_singlet_MIN
E = -2433.95907259 Hartree
Atom X Y Z
1 N 1.7336 -0.4064 -0.1231
2 N 0.5893 -0.9647 -0.5783
3 C 0.9464 -1.9105 -1.4551
4 C 2.3401 -1.9677 -1.5848
5 C 2.7992 -0.9899 -0.7152
6 Cu -1.1806 -0.2531 0.1533
7 N -2.2075 -0.2423 -1.4744
8 C -3.2938 -0.0195 -0.7933
9 O -4.4947 0.1632 -1.3472
10 C -5.6016 0.3582 -0.4708
11 C -6.4747 -0.9090 -0.4213
12 Cl -7.0796 -1.3176 -2.0623
13 C -0.0665 -2.7611 -2.1507
14 F 0.5055 -3.9285 -2.5645
15 B 1.7220 0.7214 0.9475
16 N 1.0341 1.9700 0.3636
17 N -0.2426 1.9011 -0.0683
18 C -0.5294 3.1143 -0.5450
19 C 0.5655 3.9892 -0.4277
20 C 1.5464 3.2098 0.1614

21 C -1.8527 3.3982 -1.1694
22 F -2.0938 4.7394 -1.2093
23 N -0.3437 -0.2537 2.0324
24 C -0.7476 -0.6307 3.2513
25 C 0.2555 -0.4061 4.2028
26 C 1.3012 0.1304 3.4655
27 N 0.9178 0.2089 2.1720
28 C -2.1103 -1.2050 3.4631
29 F -2.1844 -1.8229 4.6745
30 O -3.0816 0.0372 0.4821
31 Cl -5.5447 -2.2908 0.2329
32 Cl -7.8708 -0.5463 0.6578
33 F -1.9374 2.9540 -2.4587
34 F -2.8925 2.8235 -0.5011
35 F -3.0942 -0.2644 3.4419
36 F -2.4293 -2.1291 2.5149
37 F -1.1107 -3.0865 -1.3445
38 F -0.5864 -2.1741 -3.2625
39 C -2.9120 0.0022 -4.3535
40 C -1.6177 0.5140 -4.9911
41 C -4.0293 1.0452 -4.4206
42 C -3.3516 -1.3429 -4.9358
43 H 0.6198 5.0257 -0.7236
44 H 2.5606 3.4430 0.4517
45 H 0.2198 -0.6160 5.2606
46 H 2.2866 0.4551 3.7661
47 H 2.9160 -2.6346 -2.2075
48 H 3.8026 -0.6736 -0.4708
49 H 2.8451 0.9783 1.2753
50 H -5.2751 0.6027 0.5419
51 H -6.1951 1.1754 -0.8842
52 H -2.6683 -0.1716 -3.2749
53 H -4.2744 1.2756 -5.4675
54 H -4.9414 0.6827 -3.9342
55 H -3.7307 1.9785 -3.9310
56 H -3.5976 -1.2401 -6.0026
57 H -2.5602 -2.0944 -4.8424
58 H -4.2433 -1.7242 -4.4229
59 H -1.7648 0.7119 -6.0628
60 H -1.2899 1.4465 -4.5174
61 H -0.8113 -0.2210 -4.8909

tbTS3-4$_{N}$_triplet
E = -2433.94620205 Hartree
Atom X Y Z
1 C 3.8553 -33.3103 7.8299
2 N 3.7021 -32.6779 6.6446
3 N 2.4447 -32.1988 6.5329
4 C 1.8065 -32.5417 7.6572
5 C 2.6535 -33.2483 8.5207
6 B 4.7386 -32.4872 5.5037
7 N 5.0421 -30.9889 5.3095
8 N 4.0730 -30.1346 4.9097
9 C 4.6737 -28.9528 4.7560
10 C 6.0444 -29.0249 5.0637
11 C 6.2344 -30.3498 5.4125
12 Cu 1.9695 -31.1186 4.8229
13 N 0.5975 -29.8830 4.9522
14 C 0.5919 -28.7266 5.6830
15 O 1.4666 -28.3868 6.4611
16 C 3.9286 -27.7478 4.2870
17 F 2.7317 -28.0547 3.7228
18 C 0.3689 -32.1912 7.8451
19 F -0.3953 -32.5798 6.7805
20 N 2.9575 -32.5534 3.7108
21 C 2.6984 -33.2248 2.5837
22 C 3.7016 -34.1642 2.3134
23 C 4.5896 -34.0118 3.3696
24 N 4.1207 -33.0449 4.1900
25 C 1.4624 -32.9113 1.8123
26 F 1.4757 -31.6530 1.2864
27 F -0.1377 -32.8021 8.9491
28 F 0.1552 -30.8553 7.9955
29 F 3.6850 -26.8465 5.2798
30 F 4.6529 -27.0705 3.3421
31 F 1.2909 -33.7771 0.7812
32 F 0.3410 -32.9799 2.5936
33 O -0.5149 -27.9609 5.3974
34 C -0.6666 -26.7490 6.1124
35 C -1.8815 -26.8360 7.0527
36 Cl -2.0252 -25.2522 7.9006
37 Cl -3.3884 -27.1409 6.1145
38 Cl -1.6572 -28.1480 8.2491
39 C -1.8281 -30.7798 4.6601
40 C -2.4143 -29.9822 3.5150
41 C -3.8755 -30.4175 3.2797
42 C -1.5757 -30.1315 2.2384
43 H -0.6183 -30.3353 4.8249
44 H 6.7753 -28.2314 5.0231
45 H 7.1230 -30.8843 5.7158
46 H 2.4164 -33.6544 9.4918
47 H 4.8013 -33.7600 8.0935
48 H 3.7617 -34.8467 1.4799
49 H 5.5153 -34.5208 3.5948
50 H 5.7423 -33.0854 5.7690
51 H 0.2231 -26.5303 6.7070
52 H -0.8489 -25.9467 5.3931
53 H -2.4195 -28.9259 3.8113
54 H -2.2919 -30.6263 5.6373
55 H -1.6460 -31.8384 4.4494
56 H -2.0000 -29.5386 1.4192
57 H -0.5455 -29.7960 2.4019
58 H -1.5395 -31.1786 1.9135
59 H -4.4842 -30.2831 4.1820
60 H -4.3258 -29.8236 2.4742
61 H -3.9281 -31.4745 2.9891

ibInt4$_{ON}$_oss
E = -2433.979881 Hartree
Atom X Y Z
1 N 2.1384 1.6473 2.8929
2 N 1.2116 0.7277 2.5404
3 C 0.6661 0.2842 3.6795
4 C 1.2327 0.9251 4.7894
5 C 2.1689 1.7858 4.2371
6 Cu 0.9349 0.3227 0.5410
7 O -1.1408 -0.0481 0.5238
8 C -0.9936 -0.2889 -0.7169
9 O -2.0551 -0.5239 -1.5312
10 C -3.3396 -0.5689 -0.9354
11 C -3.8744 -2.0120 -0.9262
12 Cl -5.5214 -1.9714 -0.1947
13 C -0.3978 -0.7663 3.6805
14 F -0.1564 -1.7447 2.7682
15 B 3.0272 2.3476 1.8313
16 N 2.1460 3.2010 0.8974
17 N 1.1510 2.6287 0.1885
18 C 0.6088 3.6120 -0.5339
19 C 1.2509 4.8410 -0.3000
20 C 2.2308 4.5273 0.6268
21 C -0.5029 3.3325 -1.4860
22 F -1.5038 2.5874 -0.9429
23 N 3.0411 0.2731 0.3797
24 N 3.7425 1.2443 1.0039
25 C 5.0690 1.0563 0.8171
26 C 5.2469 -0.0817 0.0447
27 C 3.9409 -0.5317 -0.1949
28 C 3.5070 -1.7329 -0.9648
29 F 2.9987 -1.4205 -2.1947
30 N 0.2214 -0.2783 -1.2004
31 Cl -3.9930 -2.6526 -2.6065
32 Cl -2.8080 -3.0774 0.0370
33 F 4.5596 -2.5687 -1.1841
34 F 2.5466 -2.4468 -0.3214
35 F -1.0625 4.4923 -1.9326
36 F -0.0866 2.6522 -2.5998
37 F -0.4636 -1.3646 4.9064
38 F -1.6388 -0.2744 3.4256
39 C 0.1991 -0.5651 -4.2630
40 C 0.3325 -2.0551 -4.2193
41 C -1.1438 0.0277 -4.5513
42 C 1.4086 0.2698 -4.5497
43 H 1.0247 5.8008 -0.7383
44 H 2.9745 5.1437 1.1108
45 H 0.9991 0.7676 5.8309
46 H 2.8563 2.4778 4.7009
47 H 6.1708 -0.5300 -0.2866
48 H 5.7860 1.7389 1.2492
49 H 3.8419 3.0374 2.3754
50 H -3.3118 -0.1998 0.0916
51 H -4.0133 0.0408 -1.5419
52 H 0.3675 -0.3369 -2.2144
53 H 1.2811 1.2989 -4.1938
54 H 2.3148 -0.1485 -4.1010
55 H 1.5852 0.3310 -5.6401
56 H -1.2129 1.0627 -4.1981
57 H -1.3269 0.0478 -5.6420
58 H -1.9516 -0.5514 -4.0947
59 H 0.3329 -2.4764 -5.2423
60 H 1.2673 -2.3682 -3.7427
61 H -0.5052 -2.5202 -3.6865

Methanol amidation_solvent TCE---
--

CH3OH_TCE
E= -115.717695916 Hartree
Atom X Y Z
1 C -3.6522 -2.0875 -3.0913
2 H -2.9646 -2.8507 -3.4710
3 H -3.7738 -1.3191 -3.8713
4 H -4.6310 -2.5638 -2.9215
5 O -3.0824 -1.5680 -1.8962
6 H -3.6893 -0.8897 -1.5574

NCMe_TCE
Energy = -132.760464430 Hartree
Atom X Y Z
1 N -3.3332 0.0040 0.0934
2 C -3.4736 0.1039 -1.0545
3 C -3.6503 0.2297 -2.4962
4 H -2.6924 0.0806 -3.0052
5 H -4.3627 -0.5212 -2.8533
6 H -4.0317 1.2264 -2.7414

N2_TCE
Energy = -109.515073888 Hartree
Atom X Y Z
1 N -3.0732 -1.0933 -2.5021
2 N -3.1186 -1.7953 -3.3540

N3Troc_TCE
Energy = -475.039957758 Hartree
Atom X Y Z
1 N -2.8642 -0.3416 -1.5052
2 C -3.9936 0.3369 -1.0197
3 O -3.9524 1.0046 -0.0174
4 N -3.0253 -1.0295 -2.5395
5 N -3.0200 -1.6879 -3.4578
6 O -5.0783 0.1513 -1.8190
7 C -6.3151 0.7218 -1.4000
8 C -7.2766 -0.3919 -0.9537
9 H -6.1656 1.4201 -0.5747

10 H -6.7476 1.2355 -2.2604
11 Cl -6.6136 -1.2652 0.4700
12 Cl -8.8373 0.3835 -0.5145
13 Cl -7.5467 -1.5674 -2.2876

HOR_Int0_TCE
Energy = -2042.74603682 Hartree
Atom X Y Z
1 C 0.8631 0.3470 2.9312
2 N 0.7114 0.3594 1.5838
3 N -0.4655 -0.2012 1.2352
4 C -1.0521 -0.5658 2.3780
5 C -0.2590 -0.2454 3.4918
6 Cu -0.9570 -0.2427 -0.8769
7 N -0.2122 1.7976 -0.9311
8 N 0.9427 2.0487 -0.2800
9 C 1.3090 3.3440 -0.4441
10 C 0.3572 3.9702 -1.2354
11 C -0.5675 2.9487 -1.5073
12 B 1.6697 0.9223 0.5016
13 N 2.0414 -0.2171 -0.4845
14 N 1.0839 -0.8586 -1.1866
15 C 1.7188 -1.7708 -1.9269
16 C 3.1064 -1.7338 -1.7150
17 C 3.2650 -0.7204 -0.7809
18 C 0.9190 -2.6485 -2.8208
19 H 3.8676 -2.3483 -2.1714
20 H 4.1533 -0.3249 -0.3097
21 C -1.8155 2.9903 -2.3133
22 H 0.3318 4.9981 -1.5642
23 H 2.2125 3.7241 0.0106
24 C -2.3861 -1.2179 2.3188
25 H -0.4723 -0.4200 4.5356
26 H 1.7509 0.7572 3.3906
27 H 2.6598 1.3564 1.0170
28 F -2.8447 -1.5375 3.5598
29 F -3.3395 -0.4258 1.7361
30 F -2.3792 -2.3755 1.5874
31 F -2.0386 4.2301 -2.8293
32 F -1.7953 2.1196 -3.3701
33 F -2.9283 2.6565 -1.5884
34 F 1.7072 -3.5294 -3.4954
35 F -0.0133 -3.3881 -2.1436
36 F 0.2127 -1.9472 -3.7615
37 N -2.5802 -0.8920 -1.7284
38 C -3.5561 -1.2659 -2.2296
39 C -4.7805 -1.7335 -2.8588
40 H -5.6382 -1.1941 -2.4439
41 H -4.7317 -1.5572 -3.9384
42 H -4.9051 -2.8055 -2.6734

HOR_Int1_singlet_TCE
Energy = -1909.95044680 Hartree
Atom X Y Z
1 C 0.9327 0.3372 2.9473
2 N 0.7092 0.3667 1.6100
3 N -0.5224 -0.1137 1.3274
4 C -1.0678 -0.4423 2.5019
5 C -0.1925 -0.1826 3.5685
6 Cu -0.9729 -0.0455 -0.7585
7 N -0.2200 1.9426 -0.8678
8 N 0.9714 2.0808 -0.2421
9 C 1.4351 3.3472 -0.3828
10 C 0.5165 4.0707 -1.1277
11 C -0.4939 3.1358 -1.4034
12 B 1.6498 0.8891 0.4902
13 N 1.9363 -0.2504 -0.5272
14 N 0.9404 -0.8401 -1.2265
15 C 1.5286 -1.7483 -2.0106
16 C 2.9203 -1.7644 -1.8279
17 C 3.1344 -0.7860 -0.8690
18 C 0.6838 -2.6003 -2.8874
19 H 3.6500 -2.3886 -2.3215
20 H 4.0467 -0.4322 -0.4110
21 C -1.7728 3.3256 -2.1362
22 H 0.5668 5.1061 -1.4291
23 H 2.3801 3.6393 0.0519
24 C -2.4253 -1.0465 2.5264
25 H -0.3598 -0.3462 4.6224
26 H 1.8681 0.6891 3.3577
27 H 2.6808 1.2565 0.9754
28 F -2.9807 -0.9870 3.7669
29 F -3.2900 -0.4296 1.6655
30 F -2.4284 -2.3690 2.1639
31 F -1.7431 4.4368 -2.9210
32 F -2.0754 2.2662 -2.9456
33 F -2.8512 3.4696 -1.3016
34 F 1.4215 -3.2293 -3.8413
35 F 0.0205 -3.5830 -2.1981
36 F -0.2887 -1.8872 -3.5322

HOR_Int2O_singlet_TCE
Energy = -2385.01877116 Hartree
Atom X Y Z
1 C 0.9228 -0.1254 3.5054
2 N 0.7255 0.2512 2.2172
3 N -0.5908 0.2459 1.9170
4 C -1.2185 -0.1381 3.0318
5 C -0.3129 -0.3887 4.0765
6 Cu -1.0421 0.8110 -0.1659
7 O -3.0506 0.6423 -0.4616
8 C -3.7233 0.8017 -1.4670
9 N -3.2144 1.4507 -2.5821
10 N -3.9671 1.5139 -3.5855
11 N -4.5179 1.6470 -4.5607
12 B 1.7872 0.6151 1.1457
13 N 1.5208 2.0495 0.6124
14 N 0.3684 2.3566 -0.0222
15 C 0.4586 3.6499 -0.3470
16 C 1.6786 4.2024 0.0737
17 C 2.3250 3.1384 0.6850
18 C -0.6496 4.2863 -1.1068
19 H 2.0286 5.2163 -0.0491
20 H 3.2919 3.0788 1.1634
21 C -2.7005 -0.2566 3.0273
22 H -0.5297 -0.7091 5.0844
23 H 1.9196 -0.1786 3.9188
24 N 0.5476 -0.5412 -0.7345
25 N 1.6908 -0.3949 -0.0300
26 C 2.6500 -1.2303 -0.5012
27 C 2.1185 -1.9546 -1.5565
28 C 0.8011 -1.4760 -1.6550
29 C -0.2808 -1.9243 -2.5704
30 H 2.6016 -2.7064 -2.1625
31 H 3.6347 -1.2462 -0.0568
32 O -4.9987 0.3986 -1.5928
33 C -5.5597 -0.3746 -0.5229
34 C -5.6631 -1.8481 -0.9475
35 Cl -6.7085 -2.0141 -2.4043
36 Cl -4.0401 -2.5131 -1.3094
37 Cl -6.4024 -2.7438 0.4238
38 H 2.8816 0.5656 1.6298
39 H -4.9449 -0.3014 0.3748
40 H -6.5594 0.0195 -0.3379
41 F -3.1760 -0.6110 4.2532
42 F -3.3297 0.9079 2.6806
43 F -3.1603 -1.1969 2.1425
44 F -0.5091 5.6393 -1.1561
45 F -0.7207 3.8543 -2.4058
46 F -1.8800 4.0298 -0.5686
47 F 0.2197 -2.4438 -3.7261
48 F -1.0667 -2.9109 -2.0234
49 F -1.1367 -0.9199 -2.9153

HOR_Int2N_singlet_TCE
Energy = -2385.02231303 Hartree
Atom X Y Z
1 C 1.3278 0.5328 2.8651
2 N 0.9074 0.4707 1.5777
3 N -0.2521 -0.2216 1.4900
4 C -0.5485 -0.5959 2.7387
5 C 0.4139 -0.1437 3.6563
6 Cu -0.9893 -0.4780 -0.5053
7 N -2.8874 -0.8788 -1.2222
8 N -2.9994 -1.2056 -2.4379
9 N -2.9808 -1.5389 -3.5146
10 B 1.6238 1.0271 0.3192
11 N 0.6982 2.0323 -0.4148
12 N -0.5073 1.6490 -0.8847
13 C -1.0165 2.7202 -1.5005
14 C -0.1453 3.8199 -1.4338
15 C 0.9430 3.3291 -0.7285
16 C -2.3572 2.6236 -2.1334
17 H -0.2889 4.8104 -1.8386
18 H 1.8668 3.8047 -0.4322
19 C -1.7899 -1.3647 3.0271
20 H 0.4372 -0.2962 4.7249
21 H 2.2435 1.0457 3.1218
22 N 0.9722 -0.9356 -1.1333
23 N 1.9535 -0.1507 -0.6401
24 C 3.1597 -0.5514 -1.1107
25 C 2.9647 -1.6422 -1.9460
26 C 1.5748 -1.8333 -1.9183
27 C 0.7376 -2.8408 -2.6188
28 H 3.7053 -2.2102 -2.4885
29 H 4.0637 -0.0357 -0.8205
30 C -4.0474 -0.3154 -0.6247
31 O -5.0493 -0.2012 -1.5128
32 C -6.2351 0.4399 -1.0292
33 C -7.0853 0.7990 -2.2509
34 Cl -6.2044 1.9330 -3.3273
35 O -4.0725 0.0075 0.5335
36 Cl -7.5226 -0.6781 -3.1783
37 Cl -8.5828 1.5865 -1.6432
38 H 2.6374 1.5757 0.6438
39 H -6.7949 -0.2411 -0.3829
40 H -5.9814 1.3511 -0.4830
41 F 1.4884 -3.6979 -3.3580
42 F -0.0084 -3.6009 -1.7577
43 F -0.1706 -2.2682 -3.4729
44 F -1.6198 -2.1870 4.1058
45 F -2.8584 -0.5672 3.3319
46 F -2.1820 -2.1505 1.9857
47 F -2.6645 3.7405 -2.8425
48 F -2.4600 1.5617 -2.9960
49 F -3.3686 2.4449 -1.2232

HOR_TS2_3singlet_TCE
Energy = -2384.98552281 Hartree
Atom X Y Z
1 C 1.0890 0.5413 2.8812
2 N 0.7832 0.4263 1.5673
3 N -0.3295 -0.3271 1.4057
4 C -0.7130 -0.6876 2.6362
5 C 0.1473 -0.1635 3.6129
6 Cu -0.9681 -0.6124 -0.5959
7 N -2.7887 -0.9181 -0.9173
8 N -3.2149 -1.6726 -2.4359
9 N -2.8701 -2.3871 -3.2152
10 B 1.5515 1.0273 0.3619
11 N 0.6438 2.0287 -0.3939
12 N -0.5205 1.6241 -0.9437
13 C -1.0095 2.6897 -1.5847

14 C -0.1680 3.8072 -1.4497
15 C 0.8819 3.3334 -0.6790
16 C -2.3022 2.5926 -2.3147
17 H -0.3056 4.7973 -1.8575
18 H 1.7753 3.8239 -0.3203
19 C -1.9229 -1.5261 2.8682
20 H 0.0898 -0.2908 4.6835
21 H 1.9524 1.1085 3.1975
22 N 0.9880 -0.9007 -1.1674
23 N 1.9392 -0.1219 -0.6116
24 C 3.1616 -0.4797 -1.0690
25 C 3.0076 -1.5334 -1.9603
26 C 1.6232 -1.7524 -1.9791
27 C 0.8230 -2.7070 -2.7930
28 H 3.7726 -2.0610 -2.5093
29 H 4.0484 0.0366 -0.7311
30 C -3.8477 -0.1554 -0.5276
31 O -4.9325 -0.1370 -1.3289
32 C -5.9922 0.7022 -0.8713
33 C -7.0284 0.7785 -1.9954
34 Cl -6.3121 1.4940 -3.4787
35 O -3.7489 0.4441 0.5356
36 Cl -7.6766 -0.8550 -2.3799
37 Cl -8.3707 1.8269 -1.4150
38 H 2.5387 1.5815 0.7485
39 H -6.4638 0.2797 0.0196
40 H -5.6258 1.7083 -0.6558
41 F 1.6022 -3.6570 -3.3686
42 F -0.1340 -3.3538 -2.0580
43 F 0.1411 -2.0854 -3.8076
44 F -1.7120 -2.4157 3.8861
45 F -3.0171 -0.7966 3.2361
46 F -2.2858 -2.2513 1.7769
47 F -2.4490 3.6107 -3.2065
48 F -2.4261 1.4247 -3.0132
49 F -3.3969 2.6478 -1.4898

HOR_Int3N_singlet_TCE
Energy = -2275.48259182 Hartree
Atom X Y Z
1 C -0.8758 -1.9478 -2.2439
2 N -0.8263 -1.1282 -1.1703
3 N 0.1864 -1.4963 -0.3548
4 C 0.7685 -2.5613 -0.9205
5 C 0.1371 -2.8891 -2.1266
6 Cu 0.4684 -0.3345 1.3269
7 N 2.2517 -0.2371 1.4767
8 C 3.1411 0.2778 0.6435
9 O 4.2871 0.6791 1.2653
10 C 5.2594 1.3270 0.4575
11 C 6.5023 0.4349 0.2971
12 Cl 7.2291 0.0613 1.9010
13 B -1.7887 0.0315 -0.7928
14 N -1.0129 1.3666 -0.7474
15 N -0.0425 1.5622 0.1704
16 C 0.3763 2.8197 0.0007
17 C -0.3172 3.4539 -1.0437
18 C -1.1989 2.4837 -1.4923
19 C 1.4431 3.3942 0.8669
20 H -0.1992 4.4662 -1.4000
21 H -1.9452 2.5091 -2.2733
22 C 1.9086 -3.2628 -0.2613
23 H 0.3772 -3.6994 -2.7978
24 H -1.6298 -1.8096 -3.0052
25 N -1.5127 -0.4261 1.6812
26 N -2.3466 -0.2578 0.6314
27 C -3.6302 -0.3539 1.0421
28 C -3.6409 -0.5952 2.4103
29 C -2.2854 -0.6310 2.7548
30 C -1.6493 -0.8528 4.0826
31 H -4.4931 -0.7274 3.0591
32 H -4.4414 -0.2453 0.3369
33 O 2.9177 0.3672 -0.5685
34 Cl 6.0839 -1.1000 -0.5276
35 Cl 7.6991 1.3498 -0.6945
36 H -2.6818 0.0921 -1.5837
37 H 4.8657 1.5547 -0.5347
38 H 5.5565 2.2471 0.9648
39 F -2.5734 -1.0900 5.0459
40 F -0.7894 -1.9168 4.0734
41 F -0.9052 0.2198 4.4912
42 F 1.3027 4.7446 0.9911
43 F 1.4345 2.8695 2.1243
44 F 2.7094 3.1935 0.3826
45 F 2.0495 -4.5232 -0.7591
46 F 3.1090 -2.6428 -0.4371
47 F 1.7301 -3.3805 1.0860

HOR_Int3N_triplet_TCE
Energy = -2275.50135299 Hartree
Atom X Y Z
1 C 2.3521 0.2913 2.3923
2 N 1.2833 0.2745 1.5658
3 N 0.1949 -0.1822 2.2241
4 C 0.5934 -0.4541 3.4734
5 C 1.9548 -0.1737 3.6388
6 Cu -1.5662 -0.3209 1.1984
7 O -5.2062 0.0568 -0.1017
8 C -4.0490 -0.2496 -0.3540
9 O -3.4233 0.0021 -1.5451
10 C -4.1787 0.6409 -2.5627
11 C -4.3562 -0.3035 -3.7610
12 Cl -2.7599 -0.8012 -4.4242
13 B 1.1987 0.6933 0.0702
14 N 0.1326 1.8121 -0.0497
15 N -1.1248 1.6473 0.4208
16 C -1.7428 2.8217 0.2414
17 C -0.8920 3.7587 -0.3611
18 C 0.2981 3.0677 -0.5257
19 C -3.1641 3.0193 0.6372
20 H -1.1133 4.7827 -0.6216
21 H 1.2475 3.3791 -0.9365
22 C -0.3632 -1.0412 4.4521
23 H 2.5514 -0.2891 4.5309
24 H 3.3134 0.6346 2.0389
25 N -0.3244 -1.2473 -0.4264
26 N 0.7747 -0.5384 -0.7622
27 C 1.4087 -1.1130 -1.8125
28 C 0.7006 -2.2481 -2.1786
29 C -0.3703 -2.2817 -1.2711
30 C -1.4443 -3.3078 -1.1478
31 H 0.9272 -2.9533 -2.9638
32 H 2.3154 -0.6829 -2.2126
33 N -3.2339 -0.8685 0.5410
34 Cl -5.2841 -1.7629 -3.2873
35 Cl -5.2661 0.6005 -5.0304
36 H 2.2582 1.1001 -0.3033
37 H -5.1639 0.9379 -2.1994
38 H -3.6207 1.5192 -2.8943
39 F 0.0326 -0.8200 5.7321
40 F -1.6223 -0.5288 4.3187
41 F -0.4944 -2.3959 4.3117
42 F -3.3897 4.2924 1.0640
43 F -4.0338 2.8095 -0.3999
44 F -3.5487 2.1804 1.6384
45 F -1.1253 -4.4327 -1.8486
46 F -1.6464 -3.6885 0.1494
47 F -2.6573 -2.8952 -1.6120

HOR_MECP3N_singlet
Energy = -2275.47898401 Hartree
Atom X Y Z
1 C -2.2765 -1.9720 -3.1237
2 N -2.2536 -1.0639 -2.1248
3 N -1.3291 -1.4186 -1.2052
4 C -0.7782 -2.5653 -1.6279
5 C -1.3404 -2.9589 -2.8469
6 Cu -1.1157 -0.2357 0.4107
7 N 0.6012 -0.2344 0.8303
8 C 1.6392 0.2394 0.2150
9 O 2.6849 0.6564 0.9641
10 C 3.8537 1.0363 0.2438
11 C 4.9802 0.0211 0.5010
12 Cl 5.4079 -0.0228 2.2485
13 B -3.1686 0.1790 -1.9314
14 N -2.3181 1.4620 -1.9539
15 N -1.3692 1.6660 -1.0149
16 C -0.8524 2.8682 -1.2801
17 C -1.4591 3.4571 -2.4048
18 C -2.3942 2.5179 -2.8035
19 C 0.2272 3.4474 -0.4346
20 H -1.2500 4.4219 -2.8429
21 H -3.1077 2.5246 -3.6150
22 C 0.2577 -3.2848 -0.8284
23 H -1.1079 -3.8405 -3.4242
24 H -2.9606 -1.8558 -3.9513
25 N -3.1008 -0.0853 0.5856
26 N -3.8433 0.0496 -0.5347
27 C -5.1555 0.0840 -0.2157
28 C -5.2798 -0.0347 1.1630
29 C -3.9586 -0.1404 1.6123
30 C -3.4372 -0.2660 3.0022
31 H -6.1828 -0.0417 1.7541
32 H -5.9047 0.1905 -0.9868
33 O 1.5483 0.2803 -1.0279
34 Cl 4.4824 -1.6190 -0.0236
35 Cl 6.4180 0.5535 -0.4472
36 H -3.9987 0.2050 -2.7904
37 H 3.6612 1.0867 -0.8297
38 H 4.1701 2.0153 0.6084
39 F -4.4414 -0.4370 3.8976
40 F -2.5796 -1.3216 3.1462
41 F -2.7323 0.8386 3.3931
42 F 0.0660 4.7971 -0.2845
43 F 0.2633 2.9088 0.8147
44 F 1.4814 3.2841 -0.9588
45 F 0.3733 -4.5745 -1.2520
46 F 1.4984 -2.7343 -0.9220
47 F -0.0503 -3.3259 0.4996

HOR_MECP3N_triplet
Energy = -2275.47898548 Hartree
Atom X Y Z
1 C -2.2765 -1.9720 -3.1237
2 N -2.2536 -1.0639 -2.1248
3 N -1.3291 -1.4186 -1.2052
4 C -0.7782 -2.5653 -1.6279
5 C -1.3404 -2.9589 -2.8469
6 Cu -1.1157 -0.2357 0.4107
7 N 0.6012 -0.2344 0.8303
8 C 1.6392 0.2394 0.2150
9 O 2.6849 0.6564 0.9641
10 C 3.8537 1.0363 0.2438
11 C 4.9802 0.0211 0.5010
12 Cl 5.4079 -0.0228 2.2485
13 B -3.1686 0.1790 -1.9314
14 N -2.3181 1.4620 -1.9539
15 N -1.3692 1.6660 -1.0149
16 C -0.8524 2.8682 -1.2801
17 C -1.4591 3.4571 -2.4048
18 C -2.3942 2.5179 -2.8035
19 C 0.2272 3.4474 -0.4346
20 H -1.2500 4.4219 -2.8429

21 H -3.1077 2.5246 -3.6150
22 C 0.2577 -3.2848 -0.8284
23 H -1.1079 -3.8405 -3.4242
24 H -2.9606 -1.8558 -3.9513
25 N -3.1008 -0.0853 0.5856
26 N -3.8433 0.0496 -0.5347
27 C -5.1555 0.0840 -0.2157
28 C -5.2798 -0.0347 1.1630
29 C -3.9586 -0.1404 1.6123
30 C -3.4372 -0.2660 3.0022
31 H -6.1828 -0.0417 1.7541
32 H -5.9047 0.1905 -0.9868
33 O 1.5483 0.2803 -1.0279
34 Cl 4.4824 -1.6190 -0.0236
35 Cl 6.4180 0.5535 -0.4472
36 H -3.9987 0.2050 -2.7904
37 H 3.6612 1.0867 -0.8297
38 H 4.1701 2.0153 0.6084
39 F -4.4414 -0.4370 3.8976
40 F -2.5796 -1.3216 3.1462
41 F -2.7323 0.8386 3.3931
42 F 0.0660 4.7971 -0.2845
43 F 0.2633 2.9088 0.8147
44 F 1.4814 3.2841 -0.9588
45 F 0.3733 -4.5745 -1.2520
46 F 1.4984 -2.7343 -0.9220
47 F -0.0503 -3.3259 0.4996

HOR_TS3_4N_triplet_TCE
Energy = -2391.21682253 Hartree
Atom X Y Z
1 C 2.8666 -33.5630 2.5687
2 N 2.9475 -32.7368 3.6199
3 N 4.0966 -33.0436 4.2615
4 C 4.7287 -34.0518 3.6221
5 C 3.9690 -34.4230 2.5201
6 Cu 1.8133 -31.2197 4.3752
7 N 3.7556 -30.1162 4.4551
8 N 4.7381 -30.7687 5.1086
9 C 5.8178 -29.9650 5.2679
10 C 5.5323 -28.7371 4.6909
11 C 4.2279 -28.8948 4.1949
12 B 4.5487 -32.2476 5.5186
13 N 3.4302 -32.3685 6.5878
14 N 2.1348 -32.1103 6.2967
15 C 1.4467 -32.3421 7.4217
16 C 2.2959 -32.7467 8.4606
17 C 3.5546 -32.7500 7.8795
18 C -0.0316 -32.1638 7.4735
19 F -0.5813 -32.9190 8.4643
20 C 3.3946 -27.9023 3.4606
21 F 2.5482 -27.1910 4.2649
22 C 1.6887 -33.4911 1.6620
23 F 0.5516 -34.0000 2.2300
24 N 0.6731 -29.7589 4.7490
25 C 0.7958 -29.0241 5.8912
26 O -0.3359 -28.3055 6.1550
27 C -0.2794 -27.5152 7.3313
28 C -1.6631 -26.8932 7.5353
29 Cl -2.1075 -25.8508 6.1385
30 O 1.7993 -28.9412 6.5895
31 Cl -2.9139 -28.1635 7.7461
32 Cl -1.5880 -25.8815 9.0265
33 F -0.4124 -30.8738 7.7236
34 F -0.6394 -32.5200 6.3076
35 F 4.1756 -26.9805 2.8276
36 F 2.6105 -28.4856 2.5081
37 F 1.3843 -32.2016 1.3174
38 F 1.8970 -34.1803 0.5138
39 H -0.4867 -29.8373 4.3454
40 H 6.1676 -27.8669 4.6225
41 H 6.7063 -30.3255 5.7658
42 H 2.0254 -33.0097 9.4720
43 H 4.5224 -33.0044 8.2869
44 H 4.1779 -35.1921 1.7923
45 H 5.6710 -34.4297 3.9911
46 H 5.5708 -32.6941 5.9467
47 H -0.0340 -28.1239 8.2060
48 H 0.4558 -26.7109 7.2317
49 O -1.5654 -30.3111 4.2177
50 C -1.4242 -31.4467 3.4099
51 H -0.5040 -31.4104 2.8019
52 H -1.4384 -32.3818 3.9870
53 H -2.2695 -31.4704 2.7025

HOR_TS3_4ON_triplet_TCE
Energy = -2391.21392658 Hartree
Atom X Y Z
1 C 1.2057 0.1813 3.3017
2 N 0.8815 0.2010 1.9902
3 N -0.3337 -0.3594 1.8034
4 C -0.7680 -0.7376 3.0128
5 C 0.1674 -0.4159 4.0037
6 Cu -1.0649 -0.4994 -0.1080
7 O -3.1919 -0.1154 0.1472
8 C -3.2558 -0.3672 -1.0876
9 O -4.3258 -0.0762 -1.8453
10 C -5.3903 0.6660 -1.2527
11 C -6.6203 -0.2367 -1.0692
12 Cl -7.1584 -0.9021 -2.6520
13 B 1.6894 0.7800 0.7948
14 N 0.8805 1.9386 0.1727
15 N -0.3891 1.7536 -0.2524
16 C -0.8105 2.9582 -0.6539
17 C 0.1833 3.9377 -0.4941
18 C 1.2512 3.2355 0.0399
19 C -2.2063 3.1536 -1.1288
20 H 0.1239 4.9890 -0.7315
21 H 2.2387 3.5605 0.3342
22 C -2.1076 -1.3712 3.1771
23 H 0.0968 -0.5993 5.0650
24 H 2.1471 0.5884 3.6408
25 N 0.8501 -1.0703 -0.7219
26 N 1.8926 -0.3548 -0.2431
27 C 3.0558 -0.8414 -0.7300
28 C 2.7703 -1.9195 -1.5552
29 C 1.3742 -2.0204 -1.5075
30 C 0.5094 -3.0358 -2.1741
31 H 3.4601 -2.5471 -2.0983
32 H 3.9985 -0.3940 -0.4502
33 N -2.2013 -0.9657 -1.6541
34 Cl -6.2539 -1.5951 0.0438
35 Cl -7.9332 0.7789 -0.3732
36 H 2.7522 1.1755 1.1730
37 H -5.1008 1.0722 -0.2828
38 H -5.6472 1.4728 -1.9415
39 H -1.9084 -0.7353 -2.8750
40 F -2.1803 -2.0647 4.3466
41 F -3.1257 -0.4616 3.2031
42 F -2.3993 -2.2443 2.1711
43 F -2.3717 4.3819 -1.6917
44 F -2.5900 2.2335 -2.0616
45 F -3.1314 3.0619 -0.1210
46 F 1.2551 -4.0908 -2.6088
47 F -0.4413 -3.5377 -1.3341
48 F -0.1532 -2.5563 -3.2638
49 O -1.6621 -0.0859 -3.7827
50 C -2.8246 0.2144 -4.5120
51 H -3.3648 1.0765 -4.0968
52 H -2.4894 0.4970 -5.5225
53 H -3.5083 -0.6418 -4.5969

HOR_Int4N_triplet_TCE
Energy = -2391.23480649 Hartree
Atom X Y Z
1 C 2.8399 -33.4620 2.5243
2 N 2.9379 -32.6534 3.5885
3 N 4.0808 -32.9931 4.2240
4 C 4.6930 -34.0032 3.5684
5 C 3.9258 -34.3423 2.4617
6 Cu 1.8103 -31.1462 4.3733
7 N 3.7895 -30.0713 4.4839
8 N 4.7497 -30.7531 5.1419
9 C 5.8345 -29.9669 5.3503
10 C 5.5768 -28.7209 4.8011
11 C 4.2824 -28.8500 4.2696
12 B 4.5397 -32.2400 5.5055
13 N 3.4158 -32.3800 6.5668
14 N 2.1296 -32.0846 6.2767
15 C 1.4286 -32.3295 7.3906
16 C 2.2624 -32.7824 8.4226
17 C 3.5244 -32.7988 7.8485
18 C -0.0453 -32.1164 7.4436
19 F -0.6181 -32.9077 8.3937
20 C 3.4868 -27.8278 3.5337
21 F 2.6845 -27.0650 4.3321
22 C 1.6547 -33.3812 1.6272
23 F 0.5636 -34.0411 2.1308
24 N 0.5873 -29.7836 4.8394
25 C 0.8008 -28.9696 5.8927
26 O -0.3350 -28.2304 6.1886
27 C -0.2067 -27.3991 7.3234
28 C -1.5355 -26.6652 7.5168
29 Cl -1.9064 -25.6240 6.0969
30 O 1.8302 -28.8290 6.5421
31 Cl -2.8860 -27.8270 7.7635
32 Cl -1.3797 -25.6255 8.9835
33 F -0.3984 -30.8336 7.7560
34 F -0.6586 -32.4022 6.2596
35 F 4.3069 -26.9511 2.8808
36 F 2.6696 -28.3837 2.5926
37 F 1.2454 -32.0933 1.4239
38 F 1.9086 -33.9260 0.4116
39 H -0.4057 -29.9552 4.6613
40 H 6.2237 -27.8571 4.7710
41 H 6.7069 -30.3523 5.8581
42 H 1.9802 -33.0657 9.4253
43 H 4.4838 -33.0853 8.2542
44 H 4.1191 -35.1037 1.7216
45 H 5.6279 -34.4050 3.9306
46 H 5.5537 -32.7150 5.9230
47 H -0.0006 -27.9833 8.2254
48 H 0.5837 -26.6537 7.1918
49 O -2.0729 -30.9483 4.0680
50 C -1.5237 -32.0048 3.3937
51 H -0.4473 -32.1433 3.5753
52 H -2.0778 -32.9430 3.5653
53 H -1.6525 -31.7738 2.3137

HOR_Int4ON_triplet_TCE
Energy = -2391.23638897 Hartree
Atom X Y Z
1 C 1.0298 0.2119 3.4681
2 N 0.7943 0.2074 2.1379
3 N -0.3898 -0.3900 1.8767
4 C -0.8926 -0.7683 3.0601
5 C -0.0352 -0.4078 4.1063
6 Cu -1.0283 -0.5301 -0.0565
7 O -3.0830 -0.1022 0.2309
8 C -3.1758 -0.2690 -1.0278
9 O -4.2924 0.0729 -1.7189
10 C -5.3758 0.6342 -0.9931
11 C -6.5195 -0.3859 -0.8581

12 Cl -7.0997 -0.9094 -2.4810
13 B 1.6879 0.7675 0.9990
14 N 0.9522 1.9259 0.2917
15 N -0.2565 1.7278 -0.2761
16 C -0.5866 2.8977 -0.8330
17 C 0.4078 3.8702 -0.6301
18 C 1.3772 3.1990 0.0974
19 C -1.8644 3.0433 -1.5816
20 H 0.4118 4.8968 -0.9635
21 H 2.3291 3.5301 0.4869
22 C -2.2089 -1.4634 3.1589
23 H -0.1711 -0.5845 5.1625
24 H 1.9353 0.6484 3.8638
25 N 0.9183 -1.0426 -0.6007
26 N 1.9413 -0.3746 -0.0229
27 C 3.1225 -0.8409 -0.4856
28 C 2.8685 -1.8545 -1.3990
29 C 1.4710 -1.9384 -1.4287
30 C 0.6195 -2.8553 -2.2391
31 H 3.5790 -2.4477 -1.9540
32 H 4.0540 -0.4228 -0.1330
33 N -2.1397 -0.7629 -1.6592
34 Cl -5.9864 -1.8287 0.0643
35 Cl -7.8683 0.4254 0.0189
36 H 2.7280 1.1495 1.4482
37 H -5.0660 0.9447 0.0056
38 H -5.7452 1.4916 -1.5596
39 H -2.0750 -0.6691 -2.6738
40 F -2.2851 -2.1899 4.3105
41 F -3.2694 -0.6059 3.1789
42 F -2.4219 -2.3255 2.1263
43 F -2.0254 4.3146 -2.0421
44 F -1.9396 2.2200 -2.6732
45 F -2.9696 2.7556 -0.8282
46 F 1.3732 -3.8275 -2.8227
47 F -0.3345 -3.4828 -1.4915
48 F -0.0432 -2.2175 -3.2474
49 O -2.1237 -0.1878 -4.5836
50 C -3.3705 0.2970 -4.8847
51 H -3.3791 1.3414 -4.5038
52 H -3.5674 0.3422 -5.9673
53 H -4.1743 -0.2219 -4.3392

HOR_Int4ON_oss'__TCE
Energy = -2391.23544322 Hartree
Atom X Y Z
1 C -3.1433 -1.4763 -1.7837
2 N -2.6763 -0.6746 -0.8170
3 N -3.7237 -0.4054 -0.0061
4 C -4.8326 -1.0345 -0.4536
5 C -4.5057 -1.7411 -1.6027
6 Cu -0.8222 0.1329 -0.3387
7 N 0.5106 -1.2257 -0.8403
8 C 1.4690 -0.5316 -0.2621
9 O 2.6774 -1.0938 -0.0481
10 C 3.6965 -0.2782 0.5207
11 C 4.7266 0.1047 -0.5566
12 Cl 5.9856 1.1234 0.2346
13 B -3.5696 0.4869 1.2561
14 N -2.6472 -0.2186 2.2709
15 N -1.3845 -0.5574 1.9333
16 C -0.8725 -1.1485 3.0179
17 C -1.7996 -1.1969 4.0734
18 C -2.9261 -0.5871 3.5461
19 C 0.5211 -1.6700 3.0052
20 F 1.4629 -0.6937 2.8208
21 C -2.2466 -1.9633 -2.8688
22 F -1.3478 -2.9019 -2.4421
23 N -1.7425 1.8640 0.1650
24 N -2.9211 1.8294 0.8254
25 C -3.3733 3.0846 1.0340
26 C -2.4631 3.9783 0.4871
27 C -1.4636 3.1585 -0.0483
28 C -0.2203 3.5799 -0.7581
29 F 0.1088 2.7385 -1.7758
30 O 1.1942 0.6580 0.0869
31 Cl 5.5069 -1.3746 -1.2348
32 Cl 3.9432 1.0079 -1.8851
33 F 0.8276 -2.2881 4.1789
34 F 0.7385 -2.5835 2.0100
35 F -0.3719 4.8232 -1.2966
36 F 0.8674 3.6477 0.0623
37 F -2.9613 -2.5396 -3.8714
38 F -1.5097 -0.9587 -3.4253
39 O 1.6489 1.1185 -3.9512
40 C 1.5397 -0.2417 -3.9477
41 H -1.6635 -1.6142 5.0596
42 H -3.8968 -0.3920 3.9786
43 H -2.5144 5.0562 0.4666
44 H -4.3070 3.2523 1.5509
45 H -5.1502 -2.3463 -2.2218
46 H -5.7710 -0.9326 0.0717
47 H -4.6427 0.6913 1.7413
48 H 3.2783 0.6342 0.9478
49 H 4.1970 -0.8649 1.2929
50 H 0.5996 -2.2330 -0.9133
51 H 1.0283 -0.5064 -2.9920
52 H 0.8895 -0.6260 -4.7522
53 H 2.5106 -0.7664 -3.9287

HOR_Int4ON_oss_TCE
Energy = -2391.236316Hartree
Atom X Y Z
1 C 1.0336 0.2102 3.4661
2 N 0.7967 0.2037 2.1362
3 N -0.3899 -0.3900 1.8776
4 C -0.8928 -0.7639 3.0624
5 C -0.0329 -0.4043 4.1068
6 Cu -1.0324 -0.5304 -0.0539
7 O -3.0862 -0.0943 0.2343
8 C -3.1795 -0.2624 -1.0241
9 O -4.2939 0.0848 -1.7161
10 C -5.3753 0.6505 -0.9908
11 C -6.5233 -0.3650 -0.8569
12 Cl -7.1060 -0.8843 -2.4804
13 B 1.6911 0.7581 0.9952
14 N 0.9593 1.9184 0.2869
15 N -0.2513 1.7244 -0.2784
16 C -0.5772 2.8947 -0.8370
17 C 0.4218 3.8632 -0.6378
18 C 1.3896 3.1891 0.0893
19 C -1.8556 3.0443 -1.5838
20 H 0.4298 4.8890 -0.9734
21 H 2.3436 3.5168 0.4764
22 C -2.2113 -1.4543 3.1642
23 H -0.1683 -0.5782 5.1635
24 H 1.9411 0.6442 3.8598
25 N 0.9116 -1.0511 -0.6003
26 N 1.9384 -0.3867 -0.0250
27 C 3.1168 -0.8592 -0.4884
28 C 2.8571 -1.8734 -1.3995
29 C 1.4591 -1.9511 -1.4272
30 C 0.6028 -2.8664 -2.2342
31 H 3.5641 -2.4711 -1.9542
32 H 4.0507 -0.4446 -0.1378
33 N -2.1459 -0.7630 -1.6546
34 Cl -5.9961 -1.8111 0.0637
35 Cl -7.8686 0.4509 0.0212
36 H 2.7334 1.1365 1.4423
37 H -5.0649 0.9592 0.0082
38 H -5.7408 1.5097 -1.5571
39 H -2.0789 -0.6680 -2.6688
40 F -2.2888 -2.1776 4.3177
41 F -3.2689 -0.5932 3.1829
42 F -2.4281 -2.3183 2.1339
43 F -2.0119 4.3153 -2.0466
44 F -1.9361 2.2190 -2.6735
45 F -2.9609 2.7629 -0.8281
46 F 1.3518 -3.8426 -2.8172
47 F -0.3522 -3.4888 -1.4835
48 F -0.0594 -2.2282 -3.2426
49 O -2.1156 -0.1819 -4.5820
50 C -3.3630 0.2996 -4.8856
51 H -3.3731 1.3458 -4.5099
52 H -3.5603 0.3390 -5.9684
53 H -4.1661 -0.2177 -4.3373

HOR_MECP4-5_singlet
Energy = -2391.22132166 Hartree
Atom X Y Z
1 N -1.0845 -0.4172 1.9726
2 N -2.2928 -0.3535 2.5753
3 C -2.1564 -0.4599 3.9170
4 C -0.8080 -0.6002 4.2116
5 C -0.1881 -0.5671 2.9557
6 B -3.5930 -0.1115 1.7649
7 N -3.7110 -1.2065 0.6742
8 N -2.7514 -1.3773 -0.2624
9 C -3.1572 -2.3990 -1.0311
10 C -4.3903 -2.8990 -0.5973
11 C -4.7049 -2.1038 0.4959
12 Cu -1.0452 -0.2224 -0.1740
13 N 0.5975 0.6039 -0.7150
14 C 1.6663 -0.2148 -1.0121
15 O 2.8689 0.3930 -0.7997
16 C 4.0177 -0.3053 -1.2613
17 C 5.1791 -0.0475 -0.2953
18 Cl 4.8123 -0.7025 1.3344
19 C -2.3044 -2.8934 -2.1473
20 F -1.8417 -1.8833 -2.9414
21 C 1.2638 -0.6274 2.6354
22 F 1.5462 -1.5405 1.6601
23 N -2.5148 1.5732 0.2285
24 C -2.7088 2.8513 -0.1145
25 C -3.8390 3.3945 0.5178
26 C -4.3216 2.3442 1.2820
27 N -3.5129 1.2741 1.0893
28 C -1.7708 3.5274 -1.0519
29 F -0.4719 3.4650 -0.6309
30 F 1.7482 0.5698 2.1843
31 F 2.0032 -0.9627 3.7263
32 F -1.1950 -3.5608 -1.7070
33 F -2.9887 -3.7534 -2.9464
34 F -1.7933 2.9938 -2.3076
35 F -2.0758 4.8491 -1.1845
36 O 1.5471 -1.3499 -1.4467
37 Cl 6.6304 -0.8870 -0.9615
38 Cl 5.5281 1.7127 -0.1601
39 H -4.9599 -3.7129 -1.0196
40 H -5.5611 -2.1096 1.1548
41 H -0.3407 -0.7120 5.1781
42 H -3.0227 -0.4260 4.5618
43 H -4.2312 4.3965 0.4317
44 H -5.1712 2.2798 1.9463
45 H -4.5392 -0.1609 2.4952
46 H 3.8328 -1.3802 -1.3082
47 H 4.2966 0.0663 -2.2528
48 H 0.8779 1.5003 -0.3191
49 O 0.7520 1.4344 -2.6396
50 C -0.0337 0.6330 -3.4460
51 H -1.1116 0.7381 -3.2525
52 H 0.1482 0.9748 -4.4839
53 H 0.2582 -0.4290 -3.4072

HOR_MECP4-5_triplet
Energy = -2391.22140414 Hartree
Atom X Y Z
1 N -1.0845 -0.4172 1.9726
2 N -2.2928 -0.3535 2.5753
3 C -2.1564 -0.4599 3.9170
4 C -0.8080 -0.6002 4.2116
5 C -0.1881 -0.5671 2.9557
6 B -3.5930 -0.1115 1.7649
7 N -3.7110 -1.2065 0.6742
8 N -2.7514 -1.3773 -0.2624
9 C -3.1572 -2.3990 -1.0311
10 C -4.3903 -2.8990 -0.5973
11 C -4.7049 -2.1038 0.4959
12 Cu -1.0452 -0.2224 -0.1740
13 N 0.5975 0.6039 -0.7150
14 C 1.6663 -0.2148 -1.0121
15 O 2.8689 0.3930 -0.7997
16 C 4.0177 -0.3053 -1.2613
17 C 5.1791 -0.0475 -0.2953
18 Cl 4.8123 -0.7025 1.3344
19 C -2.3044 -2.8934 -2.1473
20 F -1.8417 -1.8833 -2.9414
21 C 1.2638 -0.6274 2.6354
22 F 1.5462 -1.5405 1.6601
23 N -2.5148 1.5732 0.2285
24 C -2.7088 2.8513 -0.1145
25 C -3.8390 3.3945 0.5178
26 C -4.3216 2.3442 1.2820
27 N -3.5129 1.2741 1.0893
28 C -1.7708 3.5274 -1.0519
29 F -0.4719 3.4650 -0.6309
30 F 1.7482 0.5698 2.1843
31 F 2.0032 -0.9627 3.7263
32 F -1.1950 -3.5608 -1.7070
33 F -2.9887 -3.7534 -2.9464
34 F -1.7933 2.9938 -2.3076
35 F -2.0758 4.8491 -1.1845
36 O 1.5471 -1.3499 -1.4467
37 Cl 6.6304 -0.8870 -0.9615
38 Cl 5.5281 1.7127 -0.1601
39 H -4.9599 -3.7129 -1.0196
40 H -5.5611 -2.1096 1.1548
41 H -0.3407 -0.7120 5.1781
42 H -3.0227 -0.4260 4.5618
43 H -4.2312 4.3965 0.4317
44 H -5.1712 2.2798 1.9463
45 H -4.5392 -0.1609 2.4952
46 H 3.8328 -1.3802 -1.3082
47 H 4.2966 0.0663 -2.2528
48 H 0.8779 1.5003 -0.3191
49 O 0.7520 1.4344 -2.6396
50 C -0.0337 0.6330 -3.4460
51 H -1.1116 0.7381 -3.2525
52 H 0.1482 0.9748 -4.4839
53 H 0.2582 -0.4290 -3.4072

product_OMe_TCE
Energy = -481.292295753 Hartree
Atom X Y Z
1 O -5.0184 -1.3697 -0.6569
2 C -4.2280 -0.6820 -1.2652
3 O -4.3677 0.6608 -1.5172
4 C -5.5841 1.2643 -1.1131
5 C -6.4378 1.6119 -2.3456
6 Cl -5.5595 2.7507 -3.4292
7 N -2.9996 -1.0736 -1.7372
8 Cl -6.8494 0.1283 -3.2717
9 Cl -7.9483 2.3962 -1.7616
10 H -6.1581 0.5980 -0.4656
11 H -5.3460 2.1913 -0.5862
12 H -2.6083 -0.5617 -2.5235
13 O -2.8463 -2.4535 -1.9011
14 C -2.0903 -2.9890 -0.8053
15 H -2.6178 -2.8407 0.1437
16 H -2.0047 -4.0583 -1.0173
17 H -1.0923 -2.5366 -0.7549

H2O_hydrolysis---

H2O
Energy = -76.4102736159 Hartree
Atom X Y Z
1 O -3.6618 -2.1289 -3.1065
2 H -2.6936 -2.0793 -3.1065
3 H -3.9382 -1.1997 -3.1065

H2O_TS3_4ON_triplet
Energy = -2351.88954635 Hartree
Atom X Y Z
1 C 1.2591 0.2657 3.2271
2 N 0.8752 0.2420 1.9320
3 N -0.3314 -0.3564 1.8189
4 C -0.6988 -0.7149 3.0549
5 C 0.2724 -0.3409 3.9916
6 Cu -1.1367 -0.5636 -0.0415
7 O -3.2593 -0.2076 0.2785
8 C -3.3523 -0.4664 -0.9514
9 O -4.4207 -0.1500 -1.7007
10 C -5.4848 0.5680 -1.0771
11 C -6.7147 -0.3394 -0.9133
12 Cl -7.2640 -0.9486 -2.5113
13 B 1.6192 0.7869 0.6808
14 N 0.7686 1.9089 0.0486
15 N -0.5158 1.6929 -0.3117
16 C -0.9670 2.8732 -0.7498
17 C 0.0231 3.8673 -0.6812
18 C 1.1197 3.2010 -0.1620
19 C -2.3812 3.0450 -1.1819
20 H -0.0602 4.9048 -0.9657
21 H 2.1153 3.5476 0.0736
22 C -2.0041 -1.4011 3.2967
23 H 0.2560 -0.5022 5.0583
24 H 2.2038 0.7060 3.5102
25 N 0.7429 -1.1281 -0.7215
26 N 1.7967 -0.3825 -0.3204
27 C 2.9385 -0.8662 -0.8584
28 C 2.6247 -1.9712 -1.6355
29 C 1.2349 -2.0920 -1.5084
30 C 0.3480 -3.1415 -2.0971
31 H 3.2911 -2.6063 -2.1984
32 H 3.8873 -0.3967 -0.6440
33 N -2.3087 -1.0802 -1.5243
34 Cl -6.3406 -1.7246 0.1567
35 Cl -8.0145 0.6677 -0.1765
36 H 2.6919 1.2137 0.9984
37 H -5.1852 0.9434 -0.0980
38 H -5.7467 1.3954 -1.7389
39 H -2.1806 -0.9696 -2.7038
40 F -1.9848 -2.0594 4.4882
41 F -3.0545 -0.5378 3.3426
42 F -2.2867 -2.3158 2.3282
43 F -2.5966 4.2957 -1.6660
44 F -2.7552 2.1699 -2.1678
45 F -3.2780 2.8522 -0.1688
46 F 1.0940 -4.1919 -2.5423
47 F -0.5357 -3.6310 -1.1840
48 F -0.3801 -2.7012 -3.1550
49 O -1.9596 -0.2412 -3.6599
50 H -2.6702 0.4266 -3.6114

H2O_TS3_4N_triplet
Energy = -2351.88726417 Hartree
Atom X Y Z
1 C 2.9185 -33.4746 2.5432
2 N 3.0118 -32.6609 3.6014
3 N 4.1336 -33.0152 4.2669
4 C 4.7372 -34.0444 3.6335
5 C 3.9871 -34.3780 2.5139
6 Cu 1.9644 -31.0807 4.3175
7 N 3.9097 -30.0808 4.4504
8 N 4.8391 -30.7710 5.1445
9 C 5.9384 -30.0058 5.3477
10 C 5.7215 -28.7695 4.7604
11 C 4.4340 -28.8778 4.2110
12 B 4.5803 -32.2408 5.5410
13 N 3.4288 -32.3335 6.5773
14 N 2.1544 -32.0200 6.2509
15 C 1.4139 -32.2946 7.3306
16 C 2.2072 -32.7803 8.3789
17 C 3.4882 -32.7888 7.8497
18 C -0.0681 -32.1312 7.3078
19 F -0.6314 -32.6852 8.4133
20 C 3.6718 -27.8533 3.4366
21 F 2.8295 -27.1064 4.2036
22 C 1.7501 -33.3728 1.6202
23 F 0.6832 -34.1055 2.0478
24 N 0.8576 -29.5832 4.5917
25 C 0.9031 -28.8743 5.7599
26 O -0.2812 -28.2324 6.0049
27 C -0.3366 -27.5316 7.2354
28 C -1.7737 -27.0352 7.4203
29 Cl -2.2370 -25.9101 6.1032
30 O 1.8708 -28.7711 6.4975
31 Cl -2.9226 -28.4175 7.4550
32 Cl -1.8411 -26.1681 8.9991
33 F -0.4672 -30.8200 7.2855
34 F -0.6360 -32.7150 6.2204
35 F 4.5265 -26.9741 2.8389
36 F 2.9132 -28.4136 2.4554
37 F 1.3113 -32.0857 1.5032
38 F 2.0628 -33.8183 0.3775
39 H -0.2081 -29.7276 4.1861
40 H 6.3898 -27.9233 4.7159
41 H 6.7922 -30.3979 5.8808
42 H 1.8851 -33.0907 9.3608
43 H 4.4307 -33.0937 8.2800
44 H 4.1799 -35.1494 1.7844
45 H 5.6553 -34.4618 4.0197
46 H 5.5737 -32.7305 5.9946
47 H -0.0794 -28.1880 8.0713
48 H 0.3374 -26.6698 7.2313
49 O -1.2854 -30.4348 4.2843
50 H -1.5883 -30.2297 5.1909

H2O_TS3_5ON_singlet
Energy = -2351.87167503 Hartree
Atom X Y Z
1 C 1.3008 0.0509 3.3452
2 N 0.9127 0.2094 2.0588
3 N -0.3614 -0.2113 1.8990
4 C -0.7679 -0.6400 3.0984
5 C 0.2440 -0.4954 4.0580
6 Cu -1.1732 -0.0854 -0.0226
7 O -3.2463 -0.0136 0.1918
8 C -3.3985 0.0612 -1.0485
9 O -4.5558 0.2752 -1.6575
10 C -5.7350 0.2839 -0.8491
11 C -6.5303 -1.0146 -1.0677
12 Cl -6.9761 -1.2004 -2.7935
13 B 1.7308 0.7495 0.8576

14 N 1.0347 2.0076 0.2865
15 N -0.2296 1.9478 -0.1835
16 C -0.5252 3.1835 -0.5965
17 C 0.5503 4.0645 -0.4000
18 C 1.5276 3.2647 0.1703
19 C -1.8447 3.4635 -1.2267
20 H 0.5971 5.1166 -0.6357
21 H 2.5296 3.4964 0.5009
22 C -2.1496 -1.1748 3.2793
23 H 0.2051 -0.7579 5.1039
24 H 2.2960 0.3320 3.6572
25 N 0.6585 -0.8759 -0.7610
26 N 1.7873 -0.3510 -0.2359
27 C 2.8774 -0.9478 -0.7728
28 C 2.4507 -1.9017 -1.6833
29 C 1.0515 -1.8129 -1.6298
30 C 0.0546 -2.6022 -2.4074
31 H 3.0484 -2.5678 -2.2863
32 H 3.8711 -0.6561 -0.4658
33 N -2.2484 -0.0877 -1.7513
34 Cl -5.5690 -2.4329 -0.5420
35 Cl -8.0230 -0.8861 -0.0678
36 H 2.8416 1.0243 1.2131
37 H -5.4921 0.3817 0.2104
38 H -6.3395 1.1284 -1.1833
39 H -2.1028 0.5628 -2.6225
40 F -2.2698 -1.7987 4.4842
41 F -3.1034 -0.2018 3.2439
42 F -2.4827 -2.0803 2.3181
43 F -2.0739 4.7979 -1.3256
44 F -1.9415 2.9518 -2.4938
45 F -2.8871 2.9201 -0.5303
46 F 0.6142 -3.7391 -2.9002
47 F -1.0271 -2.9620 -1.6715
48 F -0.4296 -1.9160 -3.4966
49 O -2.7493 -0.0200 -3.7039
50 H -2.1732 -0.7892 -3.8772

H2O_Int4NO_triplet
Energy = -2351.90100468 Hartree
Atom X Y Z
1 C 1.1723 0.2776 3.0485
2 N 0.7526 0.2243 1.7660
3 N -0.4747 -0.3398 1.7049
4 C -0.8170 -0.6497 2.9624
5 C 0.1909 -0.2759 3.8586
6 Cu -1.3290 -0.6754 -0.0952
7 O -3.3122 -0.1366 0.2868
8 C -3.5267 -0.4352 -0.9347
9 O -4.6724 -0.1188 -1.5662
10 C -5.6788 0.5531 -0.8178
11 C -6.8556 -0.3943 -0.5281
12 Cl -7.5746 -0.9986 -2.0607
13 B 1.4721 0.7356 0.4853
14 N 0.6298 1.8740 -0.1316
15 N -0.5692 1.6205 -0.7051
16 C -1.0864 2.8210 -0.9989
17 C -0.2299 3.8641 -0.6166
18 C 0.8576 3.2074 -0.0617
19 C -2.4192 2.9222 -1.6608
20 H -0.3792 4.9254 -0.7442
21 H 1.7719 3.5899 0.3686
22 C -2.1282 -1.2991 3.2723
23 H 0.1994 -0.4039 4.9298
24 H 2.1356 0.7010 3.2923
25 N 0.5273 -1.2111 -0.8360
26 N 1.5939 -0.4451 -0.5090
27 C 2.7041 -0.9156 -1.1198
28 C 2.3577 -2.0306 -1.8684
29 C 0.9824 -2.1720 -1.6516
30 C 0.0705 -3.2338 -2.1799
31 H 2.9956 -2.6561 -2.4730
32 H 3.6578 -0.4306 -0.9724
33 N -2.5548 -1.0479 -1.5715
34 Cl -6.3212 -1.7838 0.4660
35 Cl -8.0924 0.5598 0.3732
36 H 2.5604 1.1415 0.7759
37 H -5.2842 0.9249 0.1284
38 H -6.0435 1.3803 -1.4294
39 H -2.5173 -1.0434 -2.5847
40 F -2.0809 -1.9001 4.4936
41 F -3.1611 -0.4174 3.3092
42 F -2.4521 -2.2563 2.3596
43 F 0.7969 -4.2744 -2.6732
44 F -0.7448 -3.7307 -1.2089
45 F -0.7352 -2.8047 -3.1866
46 F -2.5848 4.1363 -2.2495
47 F -2.5850 1.9719 -2.6247
48 F -3.4652 2.7663 -0.7934
49 O -0.3326 0.1001 -3.3250
50 H -0.5227 0.5542 -2.4708

H2O_Int4N_triplet
Energy = -2351.89480780 Hartree
Atom X Y Z
1 C 1.7205 -3.1233 -2.8391
2 N 1.7329 -2.3500 -1.7469
3 N 2.8028 -2.7316 -1.0140
4 C 3.4573 -3.7326 -1.6423
5 C 2.7956 -4.0200 -2.8281
6 Cu 0.5212 -0.9062 -0.9689
7 N 2.4903 0.1842 -0.6828
8 N 3.3909 -0.5297 0.0318
9 C 4.4777 0.2285 0.3208
10 C 4.2864 1.4838 -0.2287
11 C 3.0266 1.3935 -0.8461
12 B 3.1459 -2.0204 0.3290
13 N 1.9282 -2.1957 1.2798
14 N 0.6822 -1.8726 0.8738
15 C -0.1454 -2.2020 1.8721
16 C 0.5668 -2.7386 2.9524
17 C 1.8878 -2.7154 2.5270
18 C -1.6210 -2.0279 1.7249
19 F -2.2887 -2.7731 2.6424
20 C 2.3543 2.4477 -1.6627
21 F 2.0689 3.5716 -0.9515
22 C 0.6868 -2.9181 -3.8957
23 F 0.4307 -4.0689 -4.5682
24 N -0.9078 0.2871 -0.7306
25 C -0.7936 1.3951 0.0343
26 O -2.0497 1.9952 0.2358
27 C -2.0606 3.2518 0.8851
28 C -2.6549 3.1491 2.3016
29 Cl -4.3179 2.4533 2.2283
30 O 0.2218 1.8790 0.4997
31 Cl -1.6400 2.1312 3.3634
32 Cl -2.7443 4.8205 2.9699
33 F -2.0339 -0.7373 1.8921
34 F -2.0532 -2.4089 0.4894
35 F 3.1734 2.8574 -2.6855
36 F 1.2026 2.0192 -2.2365
37 F -0.4931 -2.4714 -3.3733
38 F 1.0676 -1.9949 -4.8243
39 H -1.8681 -0.0591 -0.7910
40 H 4.9521 2.3333 -0.2033
41 H 5.3027 -0.1818 0.8850
42 H 0.1706 -3.1015 3.8882
43 H 2.7946 -3.0385 3.0168
44 H 3.0434 -4.7641 -3.5694
45 H 4.3420 -4.1689 -1.2023
46 H 4.1159 -2.5182 0.8230
47 H -1.0482 3.6536 0.9635
48 H -2.6970 3.9251 0.3050
49 O -3.9278 -0.1199 -0.2389
50 H -3.5655 0.6701 0.2305

19. References

(*1*) Lu, H.; Subbarayan, V.; Tao, J.; Zhang, X. P. Cobalt(II)-Catalyzed Intermolecular Benzylic C−H Amination with 2,2,2-Trichloroethoxycarbonyl Azide ($TrocN_3$). *Organometallics* **2010**, *29*, 389–393.

(*2*) Jin, W.; Kim, H.-J.; Lu, H.-L.; Rasika-Dias, H. V. Polyfluorinated Tris(pyrazolyl)borates. Syntheses and Spectroscopic and Structural Characterization of Group 1 and Group 11 Metal Complexes of [HB(3,5-$(CF_3)_2Pz)_3$]- and [HB(3-$(CF_3)Pz)_3$]. *Inorg. Chem.* **1996**, *35*, 2317–2328.

(*3*) Gómez-Emeterio, B. P.; Urbano, J.; Díaz-Requejo, M. M.; Pérez, P. J. Easy Alkane Catalytic Functionalization. *Organometallics* **2008**, *27*, 4126–4130.

(*4*) Urbano, J.; Belderraín, T. R.; Nicasio, M. C.; Trofimenko. S.; Díaz-Requejo, M. M.; Pérez, P. J. Functionalization of Primary Carbon-Hydrogen Bonds of Alkanes by Carbene Insertion with a Silver-Based Catalyst. *Organometallics* **2005**, *24*, 1528–1532.

(*5*) Schneider, J. L.; Carrier, S. M.; Ruggiero, C. E.; Young, V. G. Jr.; Tolman, W. B. Influences of Ligand Environment on the Spectroscopic Properties and Disproportionation Reactivity of Copper−Nitrosyl Complexes. *J. Am. Chem. Soc.* **1998**, *120*, 11408–11418.

(*6*) Kämpfe, A.; Brendler, E.; Kroke, E.; Wagler, J. Tp*Cu(I)–CN–SiL_2–NC–Cu(I)Tp* – a hexacoordinate Si-complex as connector for redox active metals via π-conjugated ligands. *Dalton Trans.* **2015**, *44*, 4744–4750.

(*7*) Mairena, M. A.; Urbano, J.; Carbajo, J.; Maraver, J.; Alvarez, E.; Díaz-Requejo, M. M.; Pérez, P. J. Effects of the Substituents in the Tp^xCu Activation of Dioxygen: An Experimental Study. *Inorg. Chem.* **2007**, *46*, 7428−7435.

(*8*) Caballero, A.; Díaz-Requejo, M. M.; Belderraín, T. R.; Nicasio, M. C.; Trofimenko, S.; Pérez, P. J. Highly regioselective functionalization of aliphatic carbon-hydrogen bonds with a perbromohomoscorpionate copper(I) catalyst. *J. Am. Chem. Soc.* **2003**,*125*, 1446–1447.

(*9*) Gava, R.; Olmos, A.; Noverges, B.; Varea, T.; Álvarez, E. Belderrain, T. R.; Caballero, A.; Asensio, G.; Pérez, P. J. Discovering Copper for Methane C–H Bond Functionalization. *ACS Catal.* **2015**, *5*, 3726–3730.

(*10*) Lebel, H.; Huard, K. De Novo Synthesis of Troc-Protected Amines: Intermolecular Rhodium-Catalyzed C−H Amination with N-Tosyloxycarbamates.

Org. Lett. **2007**, *9*, 639–642.

(*11*) Lee, J.; Jin, S.; Kim, D.; Hong, S. H.; Chang, S. Cobalt-Catalyzed Intermolecular C–H Amidation of Unactivated Alkanes. *J. Am. Chem. Soc.* **2021**, *143*, 5191−5200.

(*12*) Zhang, Y.; Ge, X.; Lu, H.; Li, G. Catalytic Decarboxylative C−N Formation to Generate Alkyl, Alkenyl, and Aryl Amines. *Angew. Chem. Int. Ed.* **2021**, *60*, 1845–1852.

(*13*) Tierney, M. M.; Crespi, S.; Ravelli, D.; Alexanian, E. J. Identifying Amidyl Radicals for Intermolecular C–H Functionalizations. *J. Org. Chem.* **2019**, *84*, 12983−12991.

(*14*) *Gaussian 16, Revision C.01*, M. J. Frisch, G. W. Trucks, H. B. Schlegel, G. E. Scuseria, M. A. Robb, J. R. Cheeseman, G. Scalmani, V. Barone, G. A. Petersson, H. Nakatsuji, X. Li, M. Caricato, A. V. Marenich, J. Bloino, B. G. Janesko, R. Gomperts, B. Mennucci, H. P. Hratchian, J. V. Ortiz, A. F. Izmaylov, J. L. Sonnenberg, D. Williams-Young, F. Ding, F. Lipparini, F. Egidi, J. Goings, B. Peng, A. Petrone, T. Henderson, D. Ranasinghe, V. G. Zakrzewski, J. Gao, N. Rega, G. Zheng, W. Liang, M. Hada, M. Ehara, K. Toyota, R. Fukuda, J. Hasegawa, M. Ishida, T. Nakajima, Y. Honda, O. Kitao, H. Nakai, T. Vreven, K. Throssell, J. A., Jr. Montgomery, J. E. Peralta, F. Ogliaro, M. J. Bearpark, J. J. Heyd, E. N. Brothers, K. N. Kudin, V. N. Staroverov, T. A. Keith, R. Kobayashi, J. Normand, K. Raghavachari, A. P. Rendell, J. C. Burant, S. S. Iyengar, J. Tomasi, M. Cossi, J. M. Millam, M. Klene, C. Adamo, R. Cammi, J. W. Ochterski, R. L. Martin, K. Morokuma, O. Farkas, J. B. Foresman, D. J. Fox, *Gaussian, Inc., Wallingford CT*, **2016**.

(*15*) Becke, A. D. Density-functional thermochemistry. III. The role of exact exchange. *J. Chem. Phys*. **1993**, *98*, 5648–5652.

(*16*) Grimme, S.; Jens, A.; Stephan, E.; Helge, K. A consistent and accurate ab initio parametrization of density functional dispersion correction (DFT-D) for the 94 elements H-Pu. *J. Chem. Phys.* **2010**, *132*, 154104.

(*17*) Rassolov, V. A.; Pople, J. A.; Ratner, M. A.; Windus, T. L. 6-31G* basis set for atoms K through Zn. *J. Chem. Phys*. **1998**, *109*, 1223–1229.

(*18*) Chiodo, S.; Russo, N.; Sicilia, E. LANL2DZ basis sets recontracted in the framework of density functional theory. *J. Chem. Phys*. **2006**, *125*, 104107.

(*19*) Check, C. E.; Faust, T. O.; Bailey, J. M.; Wright, B. J.; Gilbert, T. M.; Sunderlin, L. S. Addition of Polarization and Diffuse Functions to the LANL2DZ Basis Set for P-Block Elements. *J. Phys. Chem. A* **2001**, *105*, 8111–8116.

(*20*) Pritchard, B. P.; Altarawy, D.; Didier, B.; Gibson, T. D.; Windus, T. L. New Basis Set Exchange: An Open, Up-to-Date Resource for the Molecular Sciences Community. *J. Chem. Inf. Model*. ***2019***, *59*, 4814–4820.

(*21*) Marenich, A. V.; Cramer, C. J.; Truhlar, D. G. Universal Solvation Model Based on Solute Electron Density and on a Continuum Model of the Solvent Defined by the Bulk Dielectric Constant and Atomic Surface Tensions. *J. Phys. Chem. B* **2009**, *113*, 6378–6396.

(*22*) Harvey, J.; Aschi, M.; Schwarz, H. The singlet and triplet states of phenyl cation. A hybrid approach for locating minimum energy crossing points between non-interacting potential energy surfaces. *Theor. Chem. Acc*. **1998**, *99*, 95–99.

(*23*) Rodríguez-Guerra Pedregal, J.; Funes-Ardoiz, I.; Maseras, F. EasyMECP: Quick Setup of MECP Calculations with Gaussian, **2018**. Available online: https://github.com/jaimergp/easymecp.

(*24*) Luchini, G.; Alegre-Requena, J. V.; Funes-Ardoiz, I.; Paton, R. S. GoodVibes: automated thermochemistry for heterogeneous computational chemistry data. *F1000Research*, **2020**, *9*, 291–305.

(*25*) Ribeiro, R. F.; Marenich, A. V.; Cramer, C. J.; Truhlar, D. G. Use of Solution-Phase Vibrational Frequencies in Continuum Models for the Free Energy of Solvation. *J. Phys. Chem. B*, **2011**, *115*, 14556–14562.

(*26*) Grimme, S. Supramolecular Binding Thermodynamics by Dispersion-Corrected Density Functional Theory. *Chem. Eur. J.*, **2012**, *18*, 9955–9964.

(*27*) Weigenda, F.; Ahlrichs, R. Balanced basis sets of split valence, triple zeta valence and quadruple zeta valence quality for H to Rn: Design and assessment of accuracy. *Phys. Chem. Chem. Phys*., **2005**, *7*, 3297–3305.

(*28*) Weigend, F.; Furche, F.; Ahlrichs, R. Gaussian basis sets of quadruple zeta valence quality for atoms H–Kr. *J. Chem. Phys*., **2003**, *119*, 12753–12762.

(*29*) Pracht, P.; Bohle, F.; Grimme, S. Automated exploration of the low-energy chemical space with fast quantum chemical methods. *Phys. Chem. Chem. Phys.*, **2020**, *22*, 7169–7192.

(*30*) Alvarez-Moreno, M.; de Graaf, C.; Lopez, N.; Maseras, F.; Poblet, J. M.; Bo, C. Managing the Computational Chemistry Big Data Problem: The ioChem-BD Platform. *J. Chem. Inf. Model.* **2015**, *55*, 95–103.

(*31*) Pérez-Soto, R.; Besora, M.; Maseras, F. The Challenge of Reproducing with Calculations Raw Experimental Kinetic Data for an Organic Reaction. *ACS Catal.* **2023**, *13*, 8250–8260.

(*32*) Rush, L.E., Pringle, P.G.; Harvey, J.N. (2014), Computational Kinetics of Cobalt-Catalyzed Alkene Hydroformylation. *Angew. Chem. Int. Ed.*, **2014**, *53*: 8672-8676.

(*33*) Besora, M.; Maseras F. Microkinetic modeling in homogeneous catalysis. *WIREs Comput Mol Sci.* **2018**; *8*:e1372.

(*34*) Hoops S., Sahle S., Gauges R., Lee C., Pahle J., Simus N., Singhal M., Xu L., Mendes P.; Kummer U. COPASI: a COmplex PAthway SImulator. *Bioinformatics.* **2006**, *22*, 3067-74..

(*35*) Mendes, P., Hoops, S., Sahle, S., Gauges, R., Dada, J.O.; Kummer, U. Computational Modeling of Biochemical Networks Using COPASI. *Methods in Molecular Biology*, *Humana Press*. **2009**, *500*, 17-59.

(*36*) Meeus, E.J.; Derks, M.T.G.M.; van Leest, N.P.; Verhoef, C.J.; Roithová, J.; Reek, J.N.H.; de Bruin, B. Styrene aziridination with [CoIII(TAMLred)]– in water: Understanding and preventing epoxidation via nitrene hydrolysis. *Chem Catalysis*, **2023**, *3*, 100700.

(*37*) Álvarez, M.; Pérez, P.J. The catalytic nitrene-oxo transfer battle in water as solvent. *Chem Catalysis*, **2023**, *3*, 100712
